# Changes in the Control of the Hypothalamic-Pituitary Gonadal Axis Across Three Differentially Selected Strains of Laying Hens (*Gallus gallus domesticus*)

**DOI:** 10.3389/fphys.2021.651491

**Published:** 2021-03-25

**Authors:** Charlene Hanlon, Kayo Takeshima, Grégoy Y. Bédécarrats

**Affiliations:** Department of Animal Biosciences, University of Guelph, Guelph, ON, Canada

**Keywords:** laying hen, HPG axis, estradiol, reproduction, sexual maturation, deep brain photoreceptors

## Abstract

Genetic selection for earlier sexual maturation and extended production cycles in laying hens has significantly improved reproductive efficiency. While limited emphasis has been placed on the underlying physiological changes, we hypothesize that modifications in the control of the hypothalamic-pituitary gonadal (HPG) axis have occurred. Thus, three strains of White leghorn derivatives were followed from hatch to 100 weeks of age (woa), including Lohmann LSL-lite (*n* = 120) as current commercial hens, heritage Shaver White leghorns (*n* = 100) as 2000s commercial equivalents, and Smoky Joe hens (*n* = 68) as 1960s commercial equivalents. Body weight (BW) and egg production were monitored, and blood samples were collected throughout to monitor estradiol (E_2_) concentrations. Tissue samples were collected at 12, 17, 20, 25, 45, 60, 75, and 100 woa to capture changes in mRNA levels of key genes involved in the HPG axis and monitor ovarian follicular pools. All hens, regardless of strain, age or photoperiod laid their first egg within a 64-gram BW window and, as E_2_ levels increased prior to photostimulation (PS) in Lohmann and Shaver hens, a metabolic trigger likely induced sexual maturation. However, increased levels of Opsin 5 (OPN5) were observed during the maturation period. Although an elevation in gonadotrophin-releasing hormone I (GnRH-I) mRNA levels was associated with early maturation, no changes in gonadotrophin-inhibitory hormone (GnIH) mRNA levels were observed. Nonetheless, a significant shift in pituitary sensitivity to GnRH was associated with maturation. Throughout the trial, Lohmann, Shaver, and Smoky Joe hens laid 515, 417, and 257 eggs, respectively (*p* < 0.0001). Results show that the extended laying persistency in Lohmann hens was supported by sustained pituitary sensitivity to GnRH-I, recurrent elevations in follicle-stimulating hormone (FSH) mRNA levels, and five cyclical elevations in E_2_ levels. This was also associated with a consistently higher pool of small white ovarian follicles. In summary, our results demonstrate first that, regardless of photoperiodic cues, meeting a specific narrow body weight threshold is sufficient to initiate sexual maturation in Leghorn chicken derivatives. Furthermore, recurrent increases in E_2_ and FSH may be the key to sustain extended laying period, allowing modern layers to double their reproductive capacity compared to their 1960s-counterparts.

## Introduction

Genetic selection, combined with environmental and nutritional advancements, has been instrumental in maximizing the reproductive capacity of commercial layers. With the development and implementation of easily measurable phenotypic traits, breeding programs for layers have resulted in significant improvements in egg production by advancing sexual maturity, improving peak production, and reducing the ovulation interval (Anang et al., 2001, 2002; Wolc et al., 2011, 2014). Age at first egg (AFE) and cumulative egg number have been of particular focus as they are moderately to highly heritable (*h*^2^) in modern White Leghorns (*h*^2^ = 0.55 ± 0.02 and 0.34 ± 0.02, respectively; Wolc et al., 2018). However, intensive selection for these few traits has led to the erosion of genetic variability in commercial strains and newer phenotypes need to be developed for the continued improvement of breeding programs (Wolc et al., 2018). Furthermore, as the biological limit of the initiation of lay and interval between ovulations may have been reached, efforts have shifted to extend the duration of the laying cycle to 100 weeks of age (woa; van Sambeek, 2010). Interestingly, when using egg number after the peak of lay (~23 woa) for selection rather than cumulative egg number, the *h*^2^ becomes moderate to low, thus reducing its effectiveness as a phenotype (Liu et al., 2019). However, average clutch length and number of clutches have proven to be much more effective (*h*^2^ = 0.34 ± 0.02 and 0.41 ± 0.02, respectively; Wolc et al., 2018). As estimations show that an increase of 10 weeks in the laying cycle could result in a potential savings of 1 g of nitrogen per dozen eggs (Bain et al., 2016), improving late-stage production and laying persistency would not only increase profitability, but help to meet the demands of the growing population and reduce the environmental impact of the industry. Unfortunately, limited emphasis has been placed on the underlying physiological processes which make this possible. In fact, selection for these traits has led to hens entering lay prior to photostimulation (PS), while still continuing to produce at a high-level through to the end of the laying cycle (Baxter and Bédécarrats, 2019). As these birds continue to be pushed to their biological limits, the physiological implications of this intensive selection must be considered.

It has been well-established that in order to trigger sexual maturation, the hypothalamic-pituitary gonadal (HPG) axis must be activated (Dunn and Sharp, 1990; Bédécarrats et al., 2009). Prior to sexual maturation, pullets are reared under short days, which results in an increased production of melatonin (MEL), a hormone released by the pineal gland and the retina of the eye that has been shown to stimulate gonadotropin-inhibitory hormone (GnIH; Ubuka et al., 2005). This neuropeptide, GnIH, will in turn inhibit gonadotropin-releasing hormone (GnRH) production (Bentley et al., 2003, 2008) and release (Tsutsui et al., 2000), while directly suppressing the synthesis and release of gonadotropins (luteinizing hormone; LH and follicle-stimulating hormone; FSH) *via* binding to its receptor (Gonadotrophin-Inhibiting Hormone Receptor, GnIH-R) on the anterior pituitary (Ciccone et al., 2004; Ikemoto and Park, 2005; Ubuka et al., 2006; Maddineni et al., 2008). However, once the hen is placed on a photoperiod above 12 h of light, referred to as PS, MEL synthesis will decline, resulting in a downregulation of GnIH expression, thereby allowing for the stimulation of GnRH (Ikemoto and Park, 2005; Maddineni et al., 2008). In addition to a decline in MEL, direct stimulation of deep brain photoreceptors (DBPs) by environmental light results in further increases in GnRH production. Although the exact DBPs involved have not yet been established, the downstream activity of PS has been well documented (Bernal, 2002). Presently, two proposed candidates, neuropsin (Opsin 5; OPN5) and vertebrate ancient (VA)-opsin, have been considered as the primary “breeding opsins” involved in the integration of the response between photoperiod and the reproductive axis (Nakane et al., 2014; García-Fernández et al., 2015). The current working model suggests that long day (LD) photoperiods stimulate thyrotrope cells in the pars tuberalis of the pituitary to release thyroid stimulating hormone (TSH) leading to the release of GnRH in the median eminence (Prevot et al., 1999; Yoshimura et al., 2003; Yamamura et al., 2004, 2006; Nakao et al., 2008). However, expression levels of these DBPs have yet to be considered in relation to the HPG axis activation and throughout the production cycle of the laying hen.

This hypothalamic switch from inhibitory to stimulatory leads to the subsequent elevation in FSH and LH, stimulating the ovary to undergo maturation. As FSH is primarily involved in follicular recruitment and maturation (Palmer and Bahr, 1992), it is linked to the initial increase in circulating estradiol (E_2_) produced by the thousands of viable small white follicles (SWF; Robinson and Etches, 1986). In turn, E_2_ plays a key role during the maturation of the oviduct and the maintenance of the hierarchy (Shahabi et al., 1975; Shodono et al., 1975). On the other hand, in the mature hen, LH is responsible for the increase in progesterone (P_4_) production by preovulatory follicles (Yang et al., 1997), which triggers the pre-ovulatory surge in LH *via* positive feedback (Robinson et al., 1988, 2003). Additionally, E_2_ is known to influence critical physiological processes associated with egg production, such as liver metabolism, intestinal absorption, and skeletal development (Simkiss, 1967; Ohashi et al., 1991; Ohashi and Kusuhara, 1993; Turner et al., 1993; Flouriot et al., 1996; Walzem, 1996; Li et al., 2014), with elevated levels during the recruitment of SWFs that slowly decline through the remainder of the laying cycle (Senior, 1974).

We hypothesize that decades of genetic selection in commercial layer hens have altered the activation and function of the HPG axis to support the significant increase in production. Therefore, this study intends to characterize and compare the reproductive parameters in three strains of differentially selected laying hens with a particular emphasis placed on the period around sexual maturation and the persistency of lay up to 100 woa.

## Materials and Methods

### Animals

Three differentially selected strains of laying hens were housed from hatch at the Arkell Poultry Research Station (University of Guelph, Guelph, ON). The modern commercial line (Lohmann LSL lite) was developed by Lohmann-Tierzucht (2015) and heavily selected for traits related to production and egg quality. The heritage Shaver White Leghorn was chosen as a mid-point selection strain, equivalent to a commercial line corresponding to the early 2000s, as this strain was donated to the University of Guelph by Dr. Donald McQueen Shaver in 2002 (Smith, 2010) and has since been maintained in accordance with its original breeding program. Meanwhile, the white-leghorn derived Smoky Joe line (Salter et al., 1997) was used as a control strain, as breeding emphasis was placed on maintaining genetic diversity rather than production performance, thus displaying levels comparable to a 1960s commercial strain (Len et al., 1964; Baxter et al., 2014). As this line carries a recessive genetic mutation causing retinal degeneration (Salter et al., 1997), only unaffected sighted females were chosen for this trial to avoid confounding variables.

### Experimental Design and Housing

This experiment was approved by the Animal Care Committee at the University of Guelph and all procedures and management were in accordance with the guidelines from the Canadian Council for Animal Care (CCAC, 2009). Fertile eggs for the Lohmann LSL-lite were acquired from Archer’s Hatchery (Brighton, Ontario, Canada), while fertile eggs for the Shaver and Smoky Joe strains were obtained from the University of Guelph colonies. A total of 300 eggs per strain were placed in a single incubator set at 99.5°F (37.5°C) and 55% humidity, rotating once per hour for the entire period. Smoky Joe eggs were set 1 day earlier than the Lohmann and Shaver, as they require 22 days to hatch, therefore these eggs were transferred to the hatcher on embryonic day 19 (E19), while the Lohmann and Shaver eggs were moved on E18. The hatcher was maintained at 37.5°C with 90% humidity. Upon hatch, all chicks were individually wing tagged, weighed and all were vaccinated according to management guidelines (Lohmann-Tierzucht, 2015).

From the time of placement (1 day of age; doa), birds were randomly distributed into brooding cages (*n* = 12 cages) with five cages for Lohmann chicks (*n* = 120 chicks; 24 per cage), four cages for Shaver chicks (*n* = 100 chicks; 25 per cage), and three cages for Smoky Joe chicks (*n* = 68 chicks; 22–23 per cage). These were further divided into 24 cages at 6 woa to maintain space allocation (*n* = 10 for Lohmann, 8 for Shaver, and 6 for Smoky Joe). At 12 woa, all pullets were transferred into two identical rooms, each equipped with 80 standard cages (18”x10”x18”; Ford Dickison Inc., Mitchell, ON, Canada), with two birds of the same strain randomly allocated into each cage and strains randomly placed throughout both rooms. Chicks were provided with 24 h of light for the first 2 days, followed by a step down to 16 h for the remainder of the first week. Photoperiod was then decreased by 1 h per week until 10 h of light was reached at 6 woa, with an intensity of 6 lux. All pullets were photostimulated at 18 woa, using a step-up lighting program beginning with 12 h of light at 10 lux for the first week, followed by a 1 h increase per week until 16 h of light was reached (22 woa). The photoperiod was maintained at 16 h for the remainder of the laying cycle (to 100 woa). All lights in the trial were white light emitting-diode bulbs. Chicks were fed a starter crumble diet from hatch to 6 woa, followed by a pullet grower crumble diet until 18 woa and a layer breeder diet thereafter. All diets were obtained from Floredale Feed Mill and were formulated to meet or exceed NRC requirements (National Research Council, 1994). Both feed and water were provided *ad libitum* throughout the trial.

### Growth and Production Performance

Individual body weights (BWs) were recorded for all hens on a weekly basis and used to determine the BW at AFE and at the time of photostimulation. Egg production was recorded on a daily basis per cage (*n* = 2 hens) and calculated on a hen-day production percentage weekly. From this, cumulative egg production was determined on a hen-housed basis, defining the cage as the unit. Furthermore, feather loss and re-growth were monitored after the initiation of lay through to 100 woa during body weight measurements on all hens to be used as an indicator of molting behavior.

### Estradiol Analyses

Repeated blood samples were collected from 50 focal individuals per strain by venipuncture of the brachial vein. Samples were collected biweekly between 12 and 56 woa, monthly between 60 and 76 woa, and biweekly between 78 and 100 woa. Additional samples were also taken at 17 woa for the Lohmann, 21 woa for the Smoky Joe, and 19, 45, 47, and 49 woa for all three strains. To ensure consistency throughout the trial, all samples were collected during the morning approximately 2 h after lights were turned on and were completed within a 4 h period. Approximately 2 ml of blood was collected and placed in 4 ml sodium heparin tubes. The samples were then centrifuged (Centrifuge J6-MI, Beckman Coulter, Inc., United States) for 15 min at 900 × *g* at 4°C to recover plasma and stored at −20°C until extractions and assays were completed.

Prior to conducting the ELISA, fat was extracted from the plasma samples using the cold ethanol extraction procedure outlined by Baxter et al. (2014). Estradiol concentrations were measured using the DetectX commercial estradiol ELISA following the manufacturer’s protocol (DetectX 17β-estradiol enzyme immunoassay #K030-H5, Arbor Assays, Ann Arbor, Michigan). All samples were measured in duplicates and optical densities were measured at 450 nm using a microplate spectrophotometer (Model 550, Bio Rad, CA, United States). Data were analyzed with the MyAssays software^1^ using a four-parameter logistic curve. The intra-assay and inter-assay coefficient of variance (CV) were <15%.

### Tissue Collection

Specific time points representative of the different life stages of a laying hen were selected for hypothalamus, pituitary gland, and ovary collection. These times points included an immature baseline (12 woa), a pre-lay point (17 woa), the initiation of lay (20 woa), peak of lay (25 woa), throughout the laying cycle (45, 60, and 75 woa) and at the end of the trial (100 woa). At each time point, four hens, excluding the focal blood sampling birds, were randomly selected from each strain. The birds were weighed, and blood was collected prior to euthanasia *via* cervical dislocation. Hypothalamic samples were dissected following the methods outline in Lal et al. (1990), followed by the removal of the sphenoid bone to expose the pituitary gland located in the sella turcica. Samples were stored in RNAlater solution (Invitrogen, Thermo Fisher Scientific, Cat No. AM7021, Carlsbad, CA) at −80°C until total RNA extraction. Additionally, the ovaries collected from each hen were weighed prior to the follicles being separated into categories (<1, 1–2, 3–5, and 6–8 mm) and counted. Specifically, the <1 and 1–2 mm follicles were distinguished using a 5X magnifying mirror. Finally, the follicular hierarchy (F1–F6) was collected, with each of the large yellow follicles (LYFs) weighed and measured.

### RNA Extractions and Semi-Quantitative PCR

Pituitary gland total RNA was extracted using the RNeasy Mini kit (Qiagen; Cat No. 74104) according to the manufacturer’s instructions with individual pituitary glands homogenized by sonication in 600 μl of RLT buffer containing β-Mercaptoethanol (Sigma-Aldrich, Cat No. M6250, St. Louis, MO) and the total RNA eluted with 50 μl of Rnase free water. Due to the high lipid content in brain tissues, total RNA from hypothalamic samples were extracted with TRIzol Reagent (Invitrogen, Thermo Fisher Scientific, Cat No. 15596018, Carlsbad, CA) following a modified protocol. Briefly, individual hypothalami were homogenized in 3 ml of TRIzol reagent using a TissueRuptor II (Qiagen, Cat No. 9002755, Hilden, Germany). Each homogenate was then split into four 1.5 ml Eppendorf tubes, with an additional 0.25 ml of TRIzol added to each tube. Then, phase separation and total RNA recovery proceeded as per manufacturer’s instructions for each tube. The concentration and purity of these RNA samples was determined with a NanoDrop 8000 spectrophotometer (ND-8000; Thermo Fisher Scientific, Waltham, MA). All RNA extracted samples were stored at −80°C until used. Reverse transcription was performed on 1 μg of total RNA samples using the Superscript III RT (Thermo Fisher Scientific, Cat No. 18080093, Waltham, MA) according to the manufacturer’s protocol. The cDNA samples were then stored at −20°C until analysis.

The genes analyzed include chicken GnRH-I, GnIH, OPN5, and VA-Opsin in the hypothalamus, and chicken LHβ, FSHβ, Gonadotrophin-releasing hormone receptor III (GnRH-RIII), Gonadotrophin-Releasing Hormone Receptor I (GnRH-RI), and GnIH-R in the pituitary gland. Gene specific primers used are listed in Table 1, while the quantitative PCR (qPCR) conditions are outlined in Table 2. All primers and PCR conditions have been optimized in our laboratory, with primer efficiencies ranging between 0.85 and 1.15. The LinReg software (Dr. J. M. Ruijter, Academic Medical Centre, Amsterdam, Netherlands^2^) was used to determine the Ct values. Data were normalized to the geometric mean of the housekeeping genes, GAPDH and ACTB, and the 2^-ΔΔCt^ method was used to analyze the mRNA expression of each gene relative to the 12 woa samples of the Smoky Joe.

**Table 1:** Primer list, including hypothalamic genes; GnRH-I, Gonadotrophin inhibitory hormone (GnIH), OPN5, and VA-Opsin, and pituitary genes; FSH beta-subunit, LH beta-subunit, GnRH-RI, GnRH-RIII, and GnIH-R, along with reference genes, glyceraldehyde 3-phosphate dehydrogenase (GAPDH) and beta-actin (ACTB).

Gene	Direction	Primer sequence (5'-->3')	Accession no.	Reference
***Hypothalamus***				
GnRH-I	F^1^	CTCCTGTTCACCGCATCT	X69491.1	Avital-Cohen et al., 2013
	R^2^	CTGCCCTTCTCCTAGACTTTC		
GnIH	F	CGAGTGCTTATTTGCCCTTTG	AB120325.1	Ahmed et al., 2014
	R	CTCCTGGACACCTTGAGATAGA		
OPN5	F	CAGACGGTTTGGGTTTGACT	NM_001323223.1	N/A
	R	TTCAGTGTCTCACGCACCAA		
VA-Opsin	F	GAAAGAAGGCTTCAGGAGCTGAG	XM_015288991.2	N/A
	R	CTGCCCACACATGGGAAGAC		
***Pituitary gland***				
FSH beta-subunit	F	CCACGTGGTGCTCAGGATACT	NM_204257	Avital-Cohen et al., 2013
	R	AGGTACATATTTGCTGAACAGATGAGA		
LH beta-subunit	F	GTGTTGGTGCTGATGACCCT	HQ872606.1	Baxter, 2015
	R	ACCGCCACCGTTACGTTTAT		
GnRH-RI	F	AATGTGACGGTGCAGTGGTA	AJ304414	Sparling, 2016
	R	GTGTGCACGTGGAAGAGAAA		
GnRH-RIII	F	ATGTACGCCTCCGCCTTCGT	NM_001012609	Joseph et al., 2009
	R	GCAGGGTGACGGTGTGGAAG		
GnIH-R	F	CACTGATGCTGCTGACAGACTA	AB193127.1	Maddineni et al., 2008
	R	CTCATTGAAGTAGCCGTAGATGAT		
***Reference genes***				
GAPDH	F	AATGGGCACGCCATCACTAT	NM_204305.1	Baxter, 2015
	R	AGCTGAGGGAGCTGAGATGA		
ACTB	F	GTATGGAGTCCTGTGGTAT	NM_205518.1	Shimizu and Bedecarrats, 2006
	R	CACATCTGCTGGAAGGTGG		

1 Forward primer.

2 Reverse primer.

**Table 2:** Cycling conditions for RT-quantitative PCR (qPCR).

Gene	Initial denaturation		Cycling conditions^1^			Initial melt temp (°C)	No. of cycles
	Temp (°C)	Time (min)	Denaturation	Annealing	Extension		
***Hypothalamus***							
GnRH-I	95	10	95°C for 20 s	62°C for 20 s	72°C for 20 s	62	50
GnIH	95	10	95°C for 20 s	60°C for 30 s	72°C for 20 s	60	40
OPN5	95	10	95°C for 15 s	59°C for 30 s	72°C for 20 s	59	45
VA-Opsin	95	10	95°C for 15 s	60°C for 30 s	72°C for 20 s	60	45
***Pituitary gland***							
FSH beta-subunit	95	10	95°C for 15 s	60°C for 20 s	72°C for 20 s	60	45
LH beta-subunit	94	10	94°C for 20 s	60°C for 20 s	72°C for 20 s	60	40
GnRH-RI	95	10	95°C for 10 s	57°C for 15 s	72°C for 25 s	57	50
GnRH-RIII	95	10	95°C for 15 s	58°C for 15 s	72°C for 30 s	58	50
GnIH-R	95	10	95°C for 10 s	57°C for 15 s	72°C for 15 s	57	50
***Reference genes***							
ACTB	95	10	95°C for 15 s	57°C for 15 s	72°C for 30 s	57	40
GAPDH	95	10	95°C for 10 s	56°C for 30 s	72°C for 30 s	56	40

Targeted genes include hypothalamic genes; GnRH-I, GnIH, OPN5, and VA-Opsin, and pituitary genes; FSH beta-subunit, LH beta-subunit, GnRH-RI, GGnRH-RIII, and GnIH-R, along with reference genes, GAPDH and ACTB.

1 Time is in seconds (s).

### Statistical Analyses

The software SAS v9.4 (SAS Institute, Cary, NC) was used to analyze all data presented. For production and growth parameters, the data were analyzed with an ANOVA (PROC MIXED), with strain and age included as main effects, along with interactions between these variables. Meanwhile, the random effects included cage, room, tier (top and bottom) and location within the room (right, middle right, middle left, and left). Means were separated using Tukey’s multiple comparisons, with differences between the least squared means (LSMEANS statement) determined using the test of least significant differences (PDIFF statement). This determined pairwise differences, reported as an overall significance at the *p* < 0.05 level. A repeated statement was used when appropriate to reflect the repeated measures on individual hens or cages over time *via* ID or cage number, respectively. In order to analyze the normalized mRNA expression, datasets were log_2_ transformed prior to ANOVA, with only age as the main effect, as the strains were analyzed separately. For the E_2_ profiles, curves were first smoothed using the LOESS procedure in SAS with a smoothing parameter of 0.20 and a CI of 95% for each strain. In order to determine the number of peaks on average for each breed, an ANOVA (PROC MIXED) was conducted on these predicted values, with age, strain and the interaction included in the model, as outlined above.

## Results

### Body Weight

**Figure 1:**
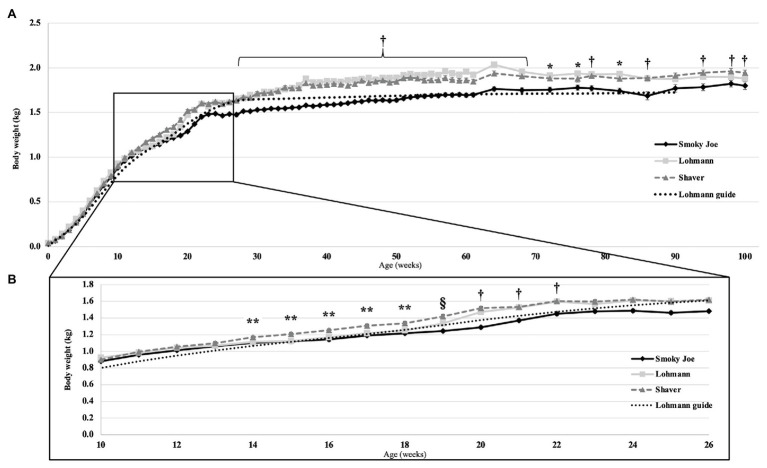
**(A)** Body weight (BW) of the Lohmann, Shaver, and Smoky Joe hens, along with the Lohmann guidelines predicted production (Lohmann-Tierzucht, 2015) from 0 to 100 weeks of age (woa). *p*-values for the sources of variation: Age, *p* < 0.0001; Strain, *p* < 0.0001; Interaction, *p* < 0.0001. **(B)** BW emphasized from 10 to 26 woa in these same strains. *Lohmann > Smoky Joe, **Shaver > Lohmann and Smoky Joe, ^†^Shaver and Lohmann > Smoky Joe, and ^§^Shaver > Lohmann > Smoky Joe.

Body weight was monitored from hatch to 100 woa and shown in Figure 1A, presented along with the recommended BW from the Lohmann LSL-lite guidelines (Lohmann-Tierzucht, 2015). At the time of hatch, Lohmann and Shaver chicks did not differ from each other, weighing 37.66 and 37.98-g, respectively, yet both were heavier (*p* < 0.0001) than the Smoky Joe chicks (29.91-g). This difference in hatch weight was most likely the result of differences in egg weight at the time of incubation, with the Lohmann and Shaver eggs (61.35 and 61.81-g, respectively) being heavier than the Smoky Joe eggs (49.84-g; *p* < 0.0001). However, these differences dissipated throughout the early growth phase, with no differences in weight present up to 13 woa (Figure 1B). From 14 to 17 woa, Shaver hens were significantly heavier compared to Lohmann and Smoky Joe hens (*p* < 0.05). Immediately prior to PS (18 woa), these differences persisted (*p* < 0.0001). However, 1-week post-PS, while Shaver hens remained the heaviest, Lohmann hens were significantly heavier (*p* < 0.0001) than the Smoky Joe hens (Table 3). From 20 to 22 woa, the BW of Lohmann hens no longer differed from that of Shaver hens, while both strains were heavier than the Smoky Joe (*p* < 0.0001). From 23 to 26 woa, all three strains demonstrated similar BW, yet for the remainder of the study (Figure 1A), although Lohmann and Shaver hens did not differ, they remained heavier than Smoky Joe hens up to 90 and 68 woa, respectively (*p* < 0.01).

**Table 3:** Body weight of the hens at the time of photostimulation (PS), 1-week post-PS, the age of first egg (AFE) and the peak in estradiol (E_2_) in Lohmann, Shaver, and Smoky Joe hens.

Strain	Body weight (kg)			
	at PS^1^ ± SEM^4^	1-week post-PS ± SEM	at AFE^2^ ± SEM	at E_2_ peak^3^ ± SEM
Lohmann	1.257 ± 0.013^B^	1.336 ± 0.013^B^	1.442 ± 0.015	1.37 ± 0.018^B^
Shaver	1.336 ± 0.016^A^	1.419 ± 0.018^A^	1.496 ± 0.0.059	1.458 ± 0.018^A^
Smoky Joe	1.216 ± 0.009^B^	1.242 ± 0.010^C^	1.428 ± 0.034	1.338 ± 0.017^B^

1 PS, photostimulation occurred at 18 woa.

2 AFE, age of first egg.

3 E_2_ peak: estradiol functional peak.

4 SEM.

A,B,C LSMeans of the strains within each trait lacking a common superscript are different (*p* < 0.05).

### Estradiol

**Figure 2:**
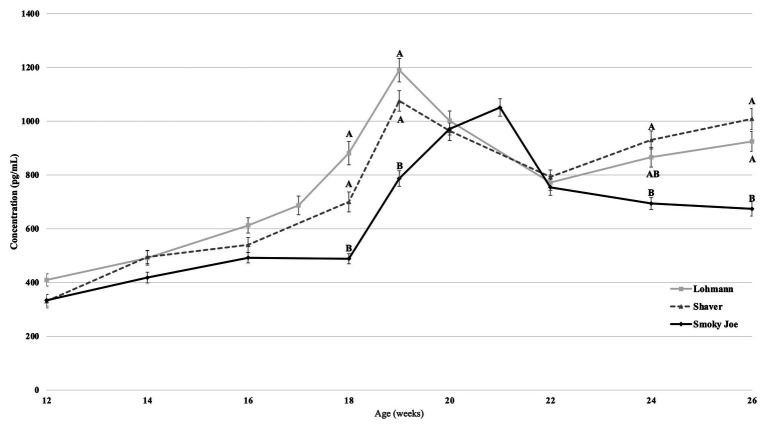
Estradiol (E_2_) concentration in Lohmann, Shaver, and Smoky Joe hens from 12 to 26 woa ± SEM. *p*-values for the sources of variation: Age, *p* < 0.0001; Strain, *p* < 0.0001; Age*Strain, *p* < 0.0001. ^A,B^represent the significance level between strains at each age. Strains at each timepoint lacking a common subscript demonstrate a significance of *p* < 0.05.

**Figure 3:**
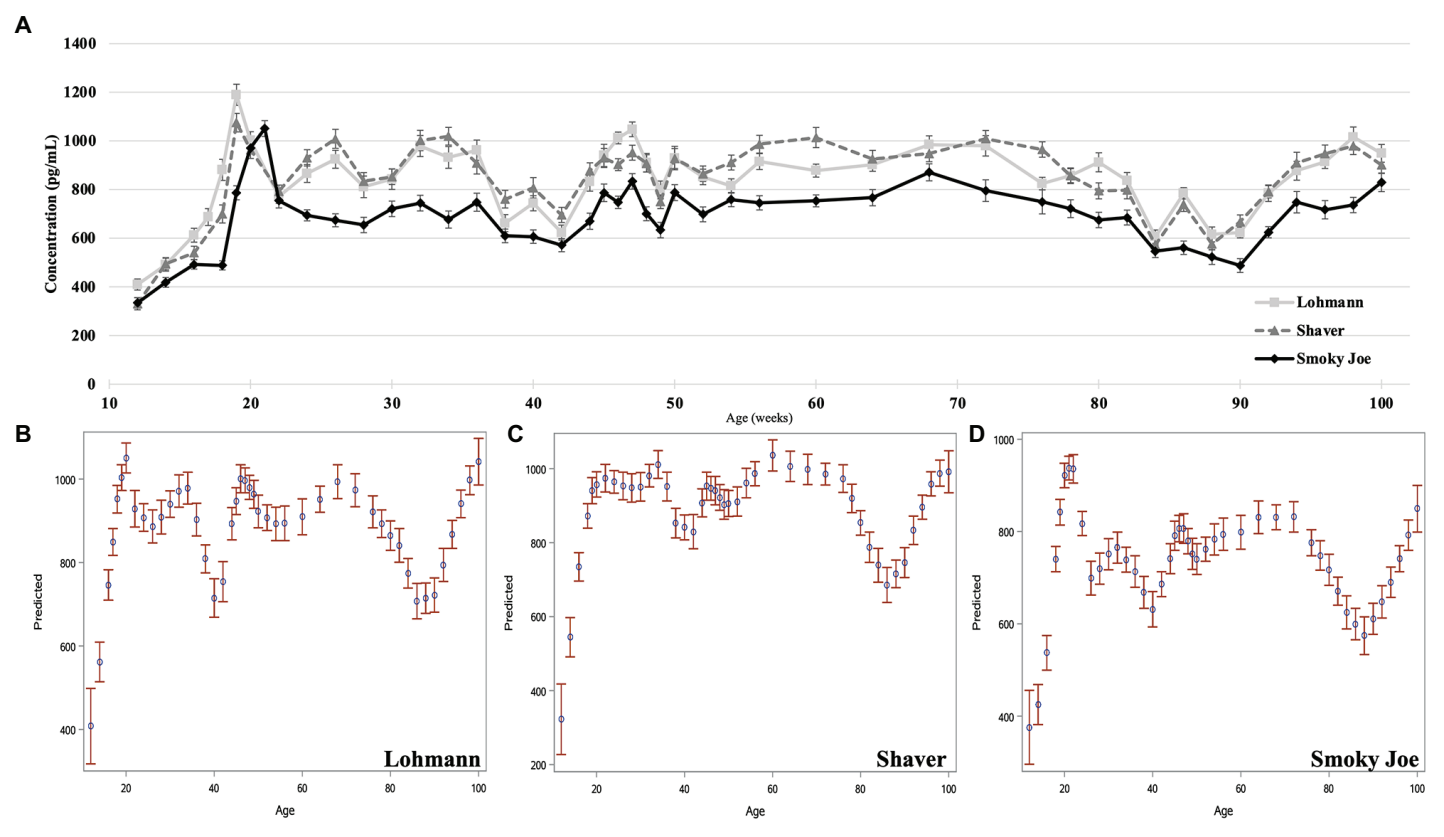
Estradiol concentration of Lohmann, Shaver, and Smoky Joe hens from 12 to 100 woa. **(A)** Average E_2_ concentration per strain ± SEM. Predicted smoothed curve for Lohmann **(B)**, Shaver **(C)**, and Smoky Joe **(D)** hens ±95% CI.

Plasma concentrations in E_2_ were used as an indirect measure of the activation of the reproductive axis and profiles from 12 to 26 woa are displayed in Figure 2. There were no differences between strains from 12 to 16 woa. At 18 and 19 woa, corresponding to and following PS, Smoky Joe hens had significantly lower E_2_ concentrations compared to both Lohmann (*p* < 0.0001) and Shaver (*p* < 0.05) hens. The timing of the functional peak, defined as the highest concentration, occurred at 19.20 ± 0.15 woa for Lohmann hens, significantly earlier than for Shaver hens (19.65 ± 0.15 woa; *p* = 0.0224). Of importance, this occurred 1-week subsequent to PS, suggesting that the initial increase in E_2_ occurred prior to the change in photoperiod. Conversely, the functional peak for Smoky Joe hens occurred significantly later (20.67 ± 0.14 woa; *p* < 0.0001), approximately 2–3 weeks post-PS, thus in line with the expected response to an increased photoperiod. Interestingly, at the time of this E_2_ peak, Lohmann and Smoky Joe hens remained lighter than the Shaver hens (*p* < 0.001; Table 3). As shown in Figure 3A, E_2_ profiles up to 100 woa reveal the occurrence of recurrent elevations similar to the functional peak, although with a less defined shape as inter-individual variations increased over time with less synchronization between sampled hens. Therefore, the curves were smoothed in order to reduce the noise caused by this variation (Figures 3B–D). On average, Lohmann hens displayed five significant peaks throughout the laying cycle, from 18 to 22, 32 to 34, 46 to 49, 64 to 72, and 96 to 100 woa (*p* < 0.0001; Figure 3B). In comparison, Shaver hens demonstrated an average of three significant peaks, occurring over longer intervals from 19 to 36, 44 to 78, and 94 to 100 woa (*p* < 0.0001; Figure 3C). Meanwhile, Smoky Joe hens also displayed three peaks, occurring from 20 to 22, 46 to 76, and 96 to 100 woa (*p* < 0.0001; Figure 3D). It is interesting to note that the initial peak appeared to be more synchronized in the Smoky Joe, compared to the Shaver, while the second peak was less synchronized occurring over a longer period of 25 weeks. The individual profiles used to create these smoothed curves have been provided in Supplementary Figure S1 and illustrate the differences in inter-individual variations.

### Egg Production

#### Sexual Maturation and Peak Production

**Figure 4:**
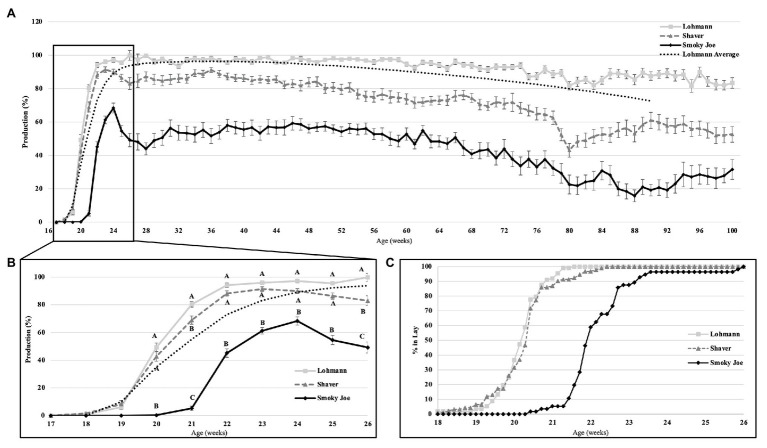
The rate of egg production in Lohmann, Shaver, and Smoky Joe hens up to 100 woa **(A)**, along with an emphasis on production from 17 to 26 woa **(B)**, and the associated percentage of hens in each strain, which have initiated lay per week **(C)**. *p*-values for the sources of variation: Age, *p* < 0.0001; Strain, *p* < 0.0001; and Age*Strain, *p* < 0.0001. ^A,B,C^Data points lacking the common superscript in **(B)** differ significantly within the ages of each strain, while no superscript indicates no difference from any other strain at that time point.

Age at first egg and cumulative egg production at 26 and 100 woa are displayed in Table 4. Lohmann and Shaver hens initiated lay significantly earlier (*p* < 0.0001) than Smoky Joe hens by approximately 12 days. Interestingly, comparing the average BW at the time of first egg revealed a 64-g window in which all hens, regardless of strain, age or photoperiod, initiated lay (Table 3). Additionally, regardless of strain, the first egg was laid 1 week after the functional peak in E_2_. As displayed in Figure 4C, all of the birds had initiated lay by 26 woa. At that time, Lohmann hens produced 4.6 eggs more than Shaver hens on average (*p* < 0.0001), with Shaver hens producing double that of Smoky Joe hens (*p* < 0.0001). By the end of the trial, this difference was further exacerbated with Lohmann hens producing almost 100 eggs more than Shaver hens (*p* < 0.0001) and approximately 250 eggs more than Smoky Joe hens (*p* < 0.0001).

**Table 4:** The AFE and cumulative egg number at 26 and 100 woa within each strain.

Strain	AFE	Cumulative egg number	
	Days of age ± SEM^1^	26 woa ± SEM	100 woa ± SEM
Lohmann	139.85 ± 0.416^A^	43.9 ± 0.9^A^	515.5 ± 6.2^A^
Shaver	140.05 ± 0.433^A^	39.3 ± 1.2^B^	417.2 ± 11.2^B^
Smoky Joe	152.64 ± 0.486^B^	19.9 ± 1.1^C^	257.9 ± 10.3^C^

1 SEM.

A,B,C LSMeans of the strains within each trait lacking a common superscript are different (*p* < 0.05).

#### Laying Cycle

Overall production rates are shown on Figure 4A, including the Lohmann predicted laying rate listed in the guidelines (Lohmann-Tierzucht, 2015). At 20 woa, both the Lohmann and Shaver hens had a significantly higher production rate (*p* < 0.0001) than the Smoky Joe. By 21 woa, the Lohmann hens increased production at a rapid rate to levels significantly higher than the Shaver hens (*p* = 0.0079), which were also higher than the Smoky Joe hens (*p* < 0.0001). From 22 to 25 woa, the production rates remained similar between both Lohmann and Shaver hens, yet significantly higher than that of Smoky Joe hens (*p* < 0.0001). By 26 woa, egg production was highest in Lohmann hens and lowest in Smoky Joe hens, with all three strains significantly differing (*p* < 0.0001; Figure 4B). The remainder of the laying cycle is displayed in Figure 4A. From 50 to 100 woa, Lohmann hens maintained a significantly higher production rate than the other strains, while it slowly declined for Shaver hens to reach a level similar to Smoky Joe hens, especially toward the end of the experiment (93–100 woa). Overall, the production rate of Smoky Joe hens remained significantly lower than that of Lohmann hens from 27 woa onward (*p* < 0.0001).

#### Signs of Spontaneous Molting

Feather loss and re-growth was recorded during body weight measurements. This data was compiled with the daily production records and used to identify hens undergoing molt, as defined by Berry (2003). By 100 woa, 83% of Smoky Joe hens and 50% of the Shaver hens underwent a spontaneous molt, while none of Lohmann hens did. In birds selected for tissue sampling, three out of four Smoky Joe hens were identified as molting at 75 woa, while this was observed in only two out of four Smoky Joe hens at 100 woa. None of the sampled Lohmann or Shaver hens displayed any sign of molt.

### Ovarian Growth and Development

#### Ovarian Weight

**Figure 5:**
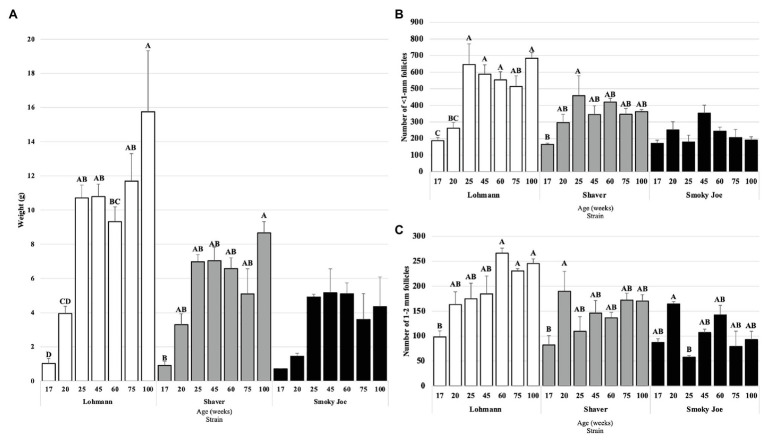
Ovary weight **(A)**, as well as the number of <1-mm **(B)** and 1–2 mm follicles **(C)** in Lohmann, Shaver, and Smoky Joe hens at 17, 20, 25, 45, 60, 75, and 100 woa. **(A)** *p*-values for the sources of variation: Age, *p* < 0.0001; Strain, *p* < 0.0001; and Age*Strain, *p* = 0.0071 **(B)** Age, *p* < 0.0001; Strain, *p* < 0.0001; and Age*Strain, *p* = 0.0003. **(C)** Age, *p* < 0.0001; Strain, *p* < 0.0001; and Age*Strain, *p* = 0.0007. ^A,B,C,D^Data points lacking the common superscript differ significantly within the ages of each strain, while no superscript indicates no difference from any other time point in that strain.

The ovary weight was recorded from 17 to 100 woa and displayed in Figure 5A. Lohmann ovaries significantly increased in weight between 20 and 25 woa (*p* = 0.01), with the highest weight observed at 100 woa. In Shaver hens, ovary weight was only found to significantly differ between 17 and 100 woa (*p* = 0.0029), with no other differences observed between sampling times, while Smoky Joe ovaries did not display any significant differences in weight throughout. This resulted in Lohmann ovaries being significantly heavier than Shaver and Smoky Joe hens at 75 and 100 woa (*p* < 0.05).

#### Follicular Recruitment

As shown in Figure 5B, ovaries from Lohmann hens were found to have accumulated a significantly higher number of <1 mm follicles at 25 woa, compared to 20 woa (*p* < 0.001), remaining consistently elevated through to 100 woa. In Shaver hens, the only significant increase in number of <1 mm follicles occurred between 17 and 25 woa (*p* = 0.0307). No differences were observed for the Smoky Joe hens throughout the sampling period. When comparing strains, ovaries from Lohmann hens had a higher number of this subset of follicles compared to Smoky Joe hens at 25 woa (*p* < 0.0001) and from 60 to 100 woa (*p* < 0.05) and compared to Shaver hens at 100 woa (*p* = 0.0103). However, this follicle category did not differ between Shaver and Smoky Joe hens at any time.

In the 1–2 mm follicle subset (Figure 5C), Lohmann hens demonstrated a steady increase in the recruitment of this particular follicular pool, with a significant increase between 17 and 60 woa (*p* < 0.01), which remained elevated through the remainder of the study. However, while a significant increase was observed earlier in Shaver hens between 17 and 20 woa (*p* = 0.0314), this did not persist as numbers of 1–2 mm follicles from 25 to 100 woa were similar to that at 17 woa. In Smoky Joe hens, there was a significant decline in the number of 1–2 mm follicles between 20 and 25 woa (*p* = 0.0367), yet no further differences were observed from 45 to 100 woa. This resulted in a significantly greater number of 1–2 mm follicles in ovaries from Lohmann hens compared to Smoky Joe hens at 25 woa and from 60 to 100 woa (*p* < 0.01) and compared to Shaver hens at 60 woa (*p* = 0.0026). No differences were observed in the 3–5 and 6–8 mm follicle categories (not shown).

The follicular hierarchy (F1–F6) was assessed in terms of diameter and weight from 25 to 100 woa (Supplementary Table S1). While there was a significant overall strain effect demonstrating that Smoky Joe follicles remained smaller and lighter than Lohmann and Shaver follicles (*p* < 0.05), there was no interaction due to the small sample size and lower number of follicles present in aging Smoky Joe hens.

### Levels of mRNA From Genes Involved in the HPG Axis

#### Deep Brain Photoreceptors

**Figure 6:**
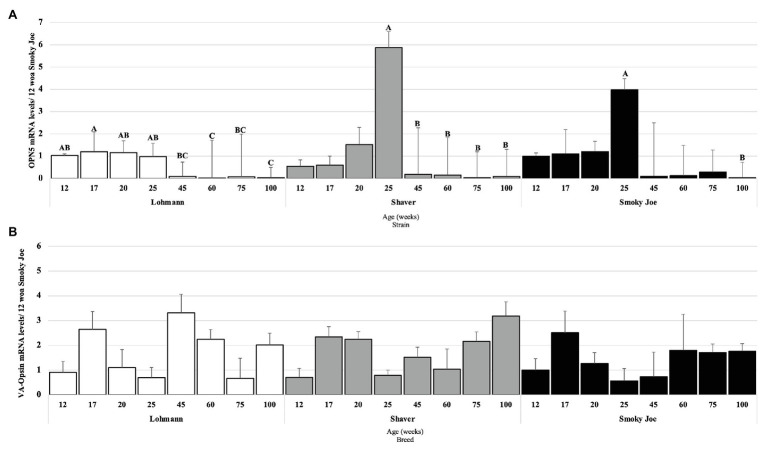
Opsin 5 (OPN5; **A**) and vertebrate ancient (VA)-Opsin **(B)** mRNA expression levels in Lohmann, Shaver, and Smoky Joe hens at 12, 17, 20, 25, 45, 60, 75, and 100 woa. *p*-values for the sources of variation of age by strain: **(A)** Lohmann, *p* < 0.0001; Shaver, *p* = 0.0029; and Smoky Joe, *p* = 0.0143. **(B)** Lohmann, *p* = 0.0502; Shaver, *p* = 0.00396; and Smoky Joe, *p* = 0.6476. ^A,B,C^Data points lacking the common superscript differ significantly within the ages of each strain, while no superscript did not differ from any other time point in that strain.

The mRNA levels of two primary photoreceptor candidates, OPN5 and VA-Opsin, were measured to assess any effect or relation with the reproductive status, age, and strain. Strains were individually analyzed, and OPN5 mRNA levels in Lohmann hens were found to be elevated from 12 to 25 woa and significantly downregulated at 60 and 100 woa (*p* < 0.01), as displayed in Figure 6A. In Shaver hens, OPN5 mRNA levels remained elevated throughout maturation, with the highest mRNA levels observed at 25 woa, significantly declining at 45 woa (*p* = 0.0465) and remaining downregulated for the duration of the extended laying cycle (*p* < 0.05). Similarly, levels of OPN5 mRNA in Smoky Joe hens remained unchanged during maturation, with the highest mRNA levels present at 25 woa, yet a significant decline was not observed until 100 woa (*p* = 0.0082). Surprisingly, no significant changes in VA-Opsin mRNA levels were observed (Figure 6B).

#### Hypothalamic Neuropeptides

**Figure 7:**
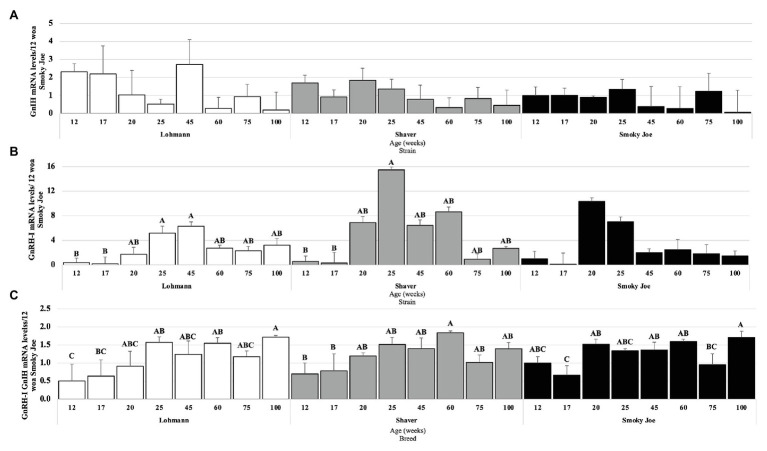
Gonadotrophin-Inhibiting Hormone (GnIH; **A**) and Gonadotrophin-Releasing Hormone I (GnRH-I; **B**) mRNA expression levels in Lohmann, Shaver, and Smoky Joe hens at 12, 17, 20, 25, 45, 60, 75, and 100 woa. **(C)** Ratio between GnRH-I and GnIH mRNA levels. *p*-values for the sources of variation of age by strain: **(A)** Lohmann, *p* = 0.0766; Shaver, *p* = 0.1713; and Smoky Joe, *p* = 0.0941. **(B)** Lohmann, *p* = 0.0155; Shaver, *p* = 0.0026; and Smoky Joe, *p* = 0.0684. **(C)** Lohmann, *p* = 0.0025; Shaver, *p* = 0.0037; and Smoky Joe, *p* = 0.0006. ^A,B,C^Data points lacking the common superscript differ significantly within the ages of each strain, while no superscript did not differ from any other time point in that strain.

As presented in Figure 7A, no age effect was found on the levels of GnIH mRNA within any of the strains. However, levels of GnRH-I mRNA were significantly higher in Lohmann hens at 25 to 45 woa, compared to 17 woa (*p* < 0.05), with intermediate levels occurring during the remainder of the study (Figure 7B). For Shaver hens, GnRH-I mRNA levels also significantly increased between 17 and 25 woa (*p* < 0.05), before decreasing to intermediate levels, yet in Smoky Joe hens, these levels were not found to significantly change over time. When the ratio of stimulatory (GnRH-I) to inhibitory (GnIH) neuropeptides was considered (Figure 7C), Lohmann hens displayed a significant increase between 12 and 25 woa (*p* < 0.01), favoring the stimulatory neuropeptides for the remainder of the sampling period. In Shaver hens, this ratio significantly shifted from inhibitory to stimulatory between 17 and 60 woa (*p* = 0.0197), again with higher GnRH-I mRNA levels remaining through to 100 woa. Interestingly, this switch was highly synchronized with the onset of lay in the Smoky Joe hens, with a significant switch to primarily GnRH-I mRNA between 17 and 20 woa (*p* = 0.0215), with only hens at 75 woa demonstrating a lower ratio, favoring GnIH mRNA expression at this time.

#### Pituitary Receptors to Hypothalamic Neuropeptides

**Figure 8:**
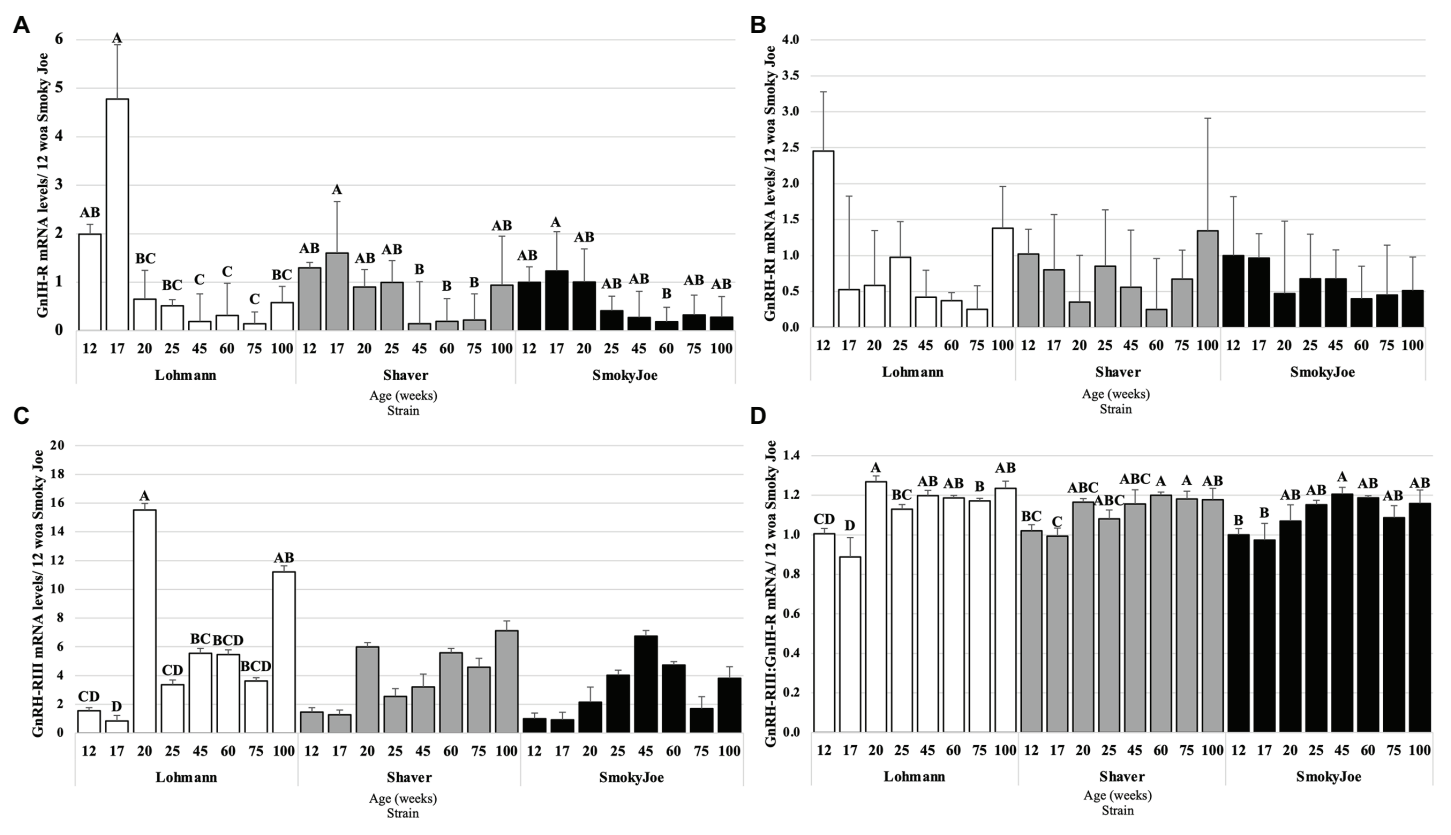
Gonadotrophin-Inhibiting Hormone Receptor (GnIH-R; **A**), Gonadotrophin-Releasing Hormone Receptor I (GnRH-RI; **B**), and Gonadotrophin-releasing hormone receptor III (GnRH-RIII; **C**) mRNA expression levels in Lohmann, Shaver, and Smoky Joe hens at 12, 17, 20, 25, 45, 60, 75, and 100 woa. **(D)** Ratio between GnRH-RIII and GnIH-R mRNA levels. *p*-values for the sources of variation of age by strain: **(A)** Lohmann, *p* < 0.0001; Shaver, *p* = 0.0034; and Smoky Joe, *p* = 0.0061. **(B)** Lohmann, *p* = 0.0506; Shaver, *p* = 0.1885; and Smoky Joe, *p* = 0.8869. **(C)** Lohmann, *p* < 0.0001; Shaver, *p* = 0.0258; and Smoky Joe, *p* = 0.0997. **(D)** Lohmann, *p* < 0.0001; Shaver, *p* = 0.0012; and Smoky Joe, *p* = 0.0086. ^A,B,C,D^Data points lacking the common superscript differ significantly within the ages of each strain, while no superscript did not differ from any other time point in that strain.

In considering the pituitary responsiveness to hypothalamic neuropeptides, GnIH-R, GnRH-RI, and GnRH-RIII mRNA levels were determined and presented in Figure 8. Levels of GnIH-R mRNA were found to significantly decline from 17 to 20 woa in Lohmann hens (*p* = 0.0065), remaining downregulated for the remainder of the trial. For Shaver hens, this significant decline in GnIH-R mRNA levels occurred between 17 and 45 woa (*p* = 0.0249) and levels remained low until 100 woa. This decline was further delayed and short-lived in Smoky Joe hens, with levels only significantly declining between 17 and 60 woa (*p* = 0.0203). As shown in Figure 8B, levels of GnRH-RI mRNA did not change statistically over the course of the experiment in any of the strains. Levels of GnRH-RIII mRNA did not differ throughout the trial in Shaver or Smoky Joe hens (Figure 8C), yet in Lohmann hens, these levels significantly increased between 17 and 20 woa (*p* < 0.001). Interestingly in this strain, GnRH-RIII mRNA levels at 100 woa did not differ from 20 woa. The ratio of GnRH-RIII to GnIH-R was also considered as an indicator of the sensitivity of the pituitary gland throughout the laying cycle (Shimizu and Bédécarrats, 2010). Interestingly, a switch in sensitivity from inhibitory to stimulatory was observed between 17 and 20 woa in Lohmann hens (*p* < 0.0001), while this occurred between 17 and 60 woa in Shaver hens (*p* = 0.008), and between 17 and 45 woa in Smoky Joe hens (*p* = 0.0155; Figure 8D).

#### Gonadotropins

**Figure 9:**
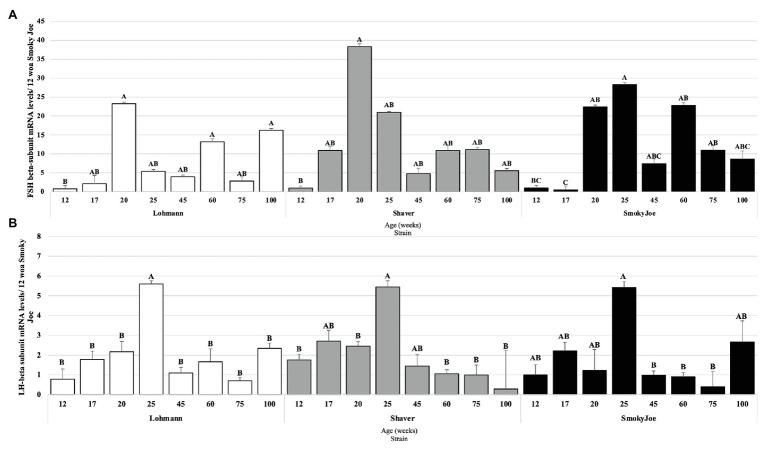
Follicle-Stimulating Hormone (FSH; **A**) and Luteinizing Hormone (LH; **B**) mRNA expression levels in Lohmann, Shaver, and Smoky Joe hens at 12, 17, 20, 25, 45, 60, 75, and 100 woa. *p*-values for the sources of variation of age by strain: **(A)** Lohmann, *p* = 0.0013; Shaver, *p* = 0.0282; and Smoky Joe, *p* = 0.0153. **(B)** Lohmann, *p* = 0.0002; Shaver, *p* = 0.0055; and Smoky Joe, *p* = 0.0077. ^A,B,C^Data points lacking the common superscript differ significantly within the ages of each strain, while no superscript did not differ from any other time point in that strain.

The pituitary mRNA levels of the gonadotropins’ beta subunits were also analyzed and are presented in Figure 9. As shown in Figure 9A, when compared to the immature state (12 woa), FSHβ mRNA levels were significantly higher at 20, 60, and 100 woa in Lohmann hens (*p* < 0.05). A similar increase between 12 and 20 woa was also observed for Shaver hens (*p* = 0.0256), yet no additional significant differences were detected. Interestingly, the initial increase in FSHβ mRNA levels in Lohmann and Shaver hens was progressive, initiating prior to PS. Conversely, in Smoky Joe hens, a significant increase occurred immediately following PS, between 17 and 20 woa (*p* = 0.0117), with levels at 60 and 75 woa also significantly higher than that of 17 woa (*p* < 0.05). For LHβ, a significant increase in mRNA levels was observed between 20 and 25 woa in Lohmann (*p* = 0.0419) and Shaver (*p* = 0.0363) hens and, although a similar increase could be seen for the Smoky Joe hens, it did not reach significance. Nonetheless, a significant decline in mRNA levels was observed between 25 and 45 woa for the Lohmann (*p* < 0.0001) and Smoky Joe hens (*p* = 0.0042), as well as between 25 and 60 woa for the Shaver hens (*p* = 0.0023). Thereafter, levels remained low in all strains (Figure 9B).

#### Relationship Between Hypothalamic Neuropeptides and Photoreceptors

Gonadotrophin-inhibitory hormone mRNA and OPN5 mRNA levels were moderately correlated in Lohmann (*r*^2^ = 0.575; *p* = 0.0005), Shaver (*r*^2^ = 0.690; *p* < 0.0001), and Smoky Joe hens (*r*^2^ = 0.762; *p* < 0.0001) throughout the study. Meanwhile, a positive correlation was observed between GnIH mRNA and VA-Opsin mRNA levels in Lohmann hens only (*r*^2^ = 0.390; *p* = 0.0226). In the Shaver hens, a negative correlation was found between OPN5 and VA-Opsin mRNA (*r*^2^ = −0.410; *p* = 0.0198), although this was not observed in the other two strains. No correlation between GnRH-I and GnIH mRNA levels were determined (Table 5).

**Table 5:** Correlations between GnRH-I, GnIH, OPN5, and VA-Opsin in Lohmann, Shaver, and Smoky Joe hens.

Trait	Lohmann				Shaver				Smoky Joe			
	GnRH-I	GnIH	OPN5	VA-Opsin	GnRH-I	GnIH	OPN5	VA-Opsin	GnRH-I	GnIH	OPN5	VA-Opsin
GnRH-I	1	0.00	−0.24	0.10	1	0.03	0.31	−0.21	1	0.28	0.25	−0.08
GnIH		1	0.58^***^	0.39^*^		1	0.69^***^	−0.14		1	0.76^***^	0.01
OPN5			1	−0.16			1	−0.41^*^			1	−0.01
VA-Opsin				1				1				1

* *p* < 0.05;

*** *p* < 0.001.

## Discussion

As breeding programs continue to emphasize selection based on reproductive capacity in laying hens, it is becoming increasingly important to determine the resulting underlying physiological modifications and anticipate any consequences of these changes. This is especially relevant as sexual maturation and extension of the laying period have been the focus of intensive selection pressure recently (van Sambeek, 2010). In the present study, through the comparison of three differentially selected strains, we identified several alterations of the reproductive axis that may have significant practical implications. These include shifting the primary trigger of sexual maturation from photoperiodic cues to possibly metabolic cues, resulting in an earlier maturation of modern commercial hens associated with a significant switch in pituitary receptor expression from inhibitory to stimulatory. Additionally, this is the first study to report the occurrence of cyclical increases in E_2_, associated to and possibly responsible for extended laying persistency.

The importance of photostimulation of DBPs on the activation of the HPG axis, and thus reproductive success, has been well-established in avian species (Sharp, 2005; Bédécarrats, 2015; Bedecarrats et al., 2016). OPN5 and VA-Opsin have been considered two of the primary candidates to act as the “breeding opsin” linked to the trigger of sexual maturation (Tarttelin et al., 2003; Nakane et al., 2014; García-Fernández et al., 2015), due to their localization in the photosensitive regions of the brain (Foster et al., 1985; Davies et al., 2012). Interestingly, in the present study, OPN5 mRNA levels were found to be highest in Lohmann hens 1 week prior to PS, although they remained relatively constant throughout the immature period, suggesting that an increase in daylength is not necessary for stimulating the expression of this opsin. This was also the case in Shaver and Smoky Joe hens, yet in both, levels were found to be highest during the peak of lay. In accordance with our results, a study in boarder canaries demonstrated that OPN5 mRNA levels remain elevated throughout the photoinducible period during exposure to longer daylength (Stevenson and Ball, 2012), indicating that increasing daylength will not impact the mRNA levels of this opsin. In the present study, mRNA levels of VA-Opsin were found to remain unchanged throughout the period of maturation in all strains. Levels of OPN5 and VA-Opsin mRNA have been shown to increase from 3 to 16 woa leading up to the period of sexual maturation in male quail (Banerjee et al., 2018). Unfortunately, in our study, the first tissue sampling occurred at 12 woa and such an increase in DBP mRNA levels may have been missed. Nonetheless, despite the maintained expression levels of both DBPs regardless of photostimulation, their importance during the activation of the HPG axis cannot be excluded.

Interestingly, the present study demonstrates that regardless of age, strain, and photoperiod, the body weight at first egg was consistently within a 64-g window for all hens. The influence of body weight on the timing of sexual maturation is not a new concept. This is in line with data previously reported in Shaver 288 hens where all birds initiated lay within 60 g of each other, despite various photostimulatory daylengths (Lewis et al., 1997). While the impact of body weight on maturation was initially recorded in meat birds (Dunnington et al., 1983) and Japanese quail (Zelenka et al., 1984), this was also reported in laying hens despite variations in AFE (Dunnington and Siegel, 1984). Furthermore, when hens were age-matched, layers were heavier than non-layers (Brody et al., 1984), suggesting chronological age may not be the best indicator of maturity. Thus, rather than absolute body weight and chronological age, body composition may be more important, as the onset of lay has been reported to be associated with lean mass, adiposity, and skeletal size (Brody et al., 1980, 1984; Krapu, 1981; Dunnington et al., 1983; Leeson and Summers, 1983; Dunnington and Siegel, 1984; Zelenka et al., 1984). In fact, evidence shows that hens that enter lay are significantly fatter than non-layers (Zelenka et al., 1986), while total body fat was found to decline as energy is diverted to the reproductive process (Zelenka et al., 1984). These studies support the hypothesis that a metabolic threshold or compositional requirement exists within a population for initiation of lay (Brody et al., 1984), regardless of the age or photoperiod of the hen. Intriguingly, despite the small window of body weight between strains at AFE, Smoky Joe hens continued to lag behind in weight at the time of and 1-week post-PS, suggesting that PS had an influence on the body weight increase of these hens. This is similar to studies in both broiler breeder and turkey hens, which showed that while body weight differed at the various ages of photostimulation, overall, there was no impact on the body weight of these hens at maturation (Robinson et al., 1996; Applegate and Lilburn, 1998). Therefore, photostimulation was shown to only influence the age of the maturation process, potentially allowing these hens to reach their optimal body weight threshold at an earlier age. This is likely an indirect impact of photostimulation on the activation of the HPG axis, thereby triggering the associated weight gain from the activation of physiological processes, combined with the increase daylength for the hens to access feed and higher energy requirements to prepare for lay, resulting in a higher feed intake during this period.

As the control of reproduction involves the hypothalamus and anterior pituitary gland, it is reasonable to assume that along with modified triggers of maturation, intensive genetic selection for increased egg production may have altered the neuroendocrine responses. As this is the first study conducted in which GnIH mRNA levels were followed from the period of growth in immature pullets through to sexual maturation and an extended laying cycle, we were surprised that no changes were observed. However, this is consistent with a lack of changes in GnIH mRNA levels between laying and out-of-lay hens previously reported in broiler breeders (Ciccone et al., 2004, 2005). In fact, the size of GnIH-containing neurons were found to be unaffected during the photosensitive to photostimulatory phases in song sparrows, while the number of these neurons did not differ based on the sensitivity or length of the photoperiod (Bentley et al., 2003). Likewise in Tree Sparrows, levels of GnIH mRNA remained consistently low during growth and development, only elevating with the regression of the reproductive tract during the photorefractory state (Dixit et al., 2017). Conversely, we did observe an elevation in GnRH-I mRNA levels in the Lohmann and Shaver hens, although in Smoky Joe hens, this elevation was not found to be significant with greater inter-individual variation. These findings are consistent with a study in broiler breeders, which demonstrated the highest GnRH-I mRNA content in younger laying hens, with a higher ratio of GnRH-I to GnIH during this early laying phase (Ciccone et al., 2005). In fact, while Ni et al. (2013) showed no difference in GnRH-I mRNA levels between exposure to SD and LD at the same age in ISA brown layers, there was an elevation under LD between 112 and 136 doa. Using a similar stimulatory photoperiod, our study reports similar effects. However, future studies should consider the effect of non-photostimulatory daylengths during the period of maturation to avoid any confounding factors between photostimulatory and metabolic inputs on the HPG axis.

At the level of the anterior pituitary gland, the decline in GnIH-R mRNA levels over time in all strains was in line with previous results from our laboratory, with receptor levels shown to be highest in immature hens and declining at the time of peak lay (Shimizu and Bédécarrats, 2010). A similar decline in GnIH-R mRNA levels between immature and mature hens was also reported in broiler breeders (Maddineni et al., 2008). Interestingly, this decline occurred much more rapidly in Lohmann hens, resulting in a significant switch in pituitary sensitivity to these neuropeptides from inhibitory to stimulatory during the period of maturation leading to the first egg. This was not the case in Shaver and Smoky Joe hens, as no statistical differences in GnRH-RIII mRNA levels were observed. In fact, we did not observe any significant alteration in the levels of GnIH-R or GnRH-RIII mRNA in the pituitary gland of Shaver or Smoky Joe hens between the immature state and entry into lay. This indicates that efforts to advance genetic selection through phenotypic selection may have resulted in changing the pituitary sensitivity to hypothalamic factors. It is important to note that this study is limited to mRNA levels at specific intervals and more frequent sampling would be required to determine the precise timing of this switch in receptor mRNA levels.

Despite the different mRNA levels in hypothalamic neuropeptide receptors between strains, all hens demonstrated a single significant elevation in LH-β mRNA levels at the time of peak lay. This is consistent with Ciccone et al. (2005) who showed that LH-β mRNA levels were higher in younger than older laying hens or those hens which had dropped out of lay. Nonetheless, levels of FSH-β mRNA were found to significantly increase at 20 woa in all three strains, confirming the activation of the pituitary gland. This initial elevation in FSH mRNA levels is consistent with results reported by Ni et al. (2013), but contrast with Ciccone et al. (2005) who reported that FSH-β mRNA levels were highest in hens that were out of lay, while no differences in levels were observed between young and old layers. However, in the same study, pituitary mRNA levels did not correspond to plasma FSH levels, which were higher in old compared to young laying hens (Ciccone et al., 2005). Unlike Smoky Joe hens, a slight non-significant increase was observed the week prior to PS in Lohmann and Shaver hens suggesting a possible activation at an earlier age. Unfortunately, due to the timing of tissue sample collection this study was unable to capture precisely the time of FSH stimulation, in particular between 18 and 19 woa.

The resulting activation of SWFs, acting as the follicular pool for the remainder of the laying cycle, produces the surge in E_2_ observed at this time (Robinson and Etches, 1986). Thus, we opted to use this initial functional peak in E_2_ as a marker of sexual maturation. In fact, this elevation in plasma E_2_ is key to the activation of the liver to initiate the production and accumulation of yolk proteins (Tanabe et al., 1981; Flouriot et al., 1996; Walzem, 1996; Li et al., 2014), diverting calcium from structural development to a medullary bone source for the formation of the shell (Simkiss, 1967; Ohashi et al., 1991; Ohashi and Kusuhara, 1993; Turner et al., 1993), and stimulating the development of the oviduct (Etches, 1996). In the present study, the functional peak occurred significantly earlier in Lohmann hens than in the other strains, while it was significantly delayed in Smoky Joe hens. This indicates that the maturation of the ovary occurred later in Shaver and Smoky Joe hens compared to Lohmann hens. Interestingly, the period between the E_2_ functional peak and the timing of the first egg was shortest for Lohmann and Shaver hens, which may initially suggest these hens have the ability to rapidly prepare for the laying cycle. However, due to the time required for a small white follicle to develop into an ovulatory follicle (Johnson, 2014), it is likely a threshold plasma concentration of E_2_ is required to be produced by these small white follicles to trigger follicular selection. Thereby, the functional peak in E_2_ would occur subsequent to achieving ovarian maturation. If so, this threshold level would have been reached at an earlier age in Lohmann and Shaver hens, prior to PS. While there is a considerable amount of literature highlighting the roles of E_2_ in the various physiological processes outlined above, few studies have investigated the relationship between the peak in E_2_ and the AFE. However, our results are in line with those previously reported for white leghorns (Tanabe et al., 1981), but much shorter than the 2–3 weeks reported by Senior (1974) for Sussex white and Rhode Island Red crosses. Thus, although genetic selection for a faster maturation is most likely in part responsible, it may also be strain dependent. In the ovary, we hypothesize that the strong rapid peak in E_2_ observed in the Lohmann hens was the result of the significantly higher number of small white follicles (<1-mm) observed in this modern commercial strain. As anticipated, this resulted in an overall peak production of 68.4% for the Smoky Joe hens, while Shaver hens peaked at 91.4% and Lohmann hens achieved the highest peak at 99.9%. In fact, Smoky Joe hens were comparable to reports from the 1960s, with a low peak production combined with a shortened laying cycle (Len et al., 1964). In the case of Lohmann and Shaver hens, the improved early production rates observed were to be expected as this has been the focus of genetic selection for many years prior to the 2000s (Acharya et al., 1969; Wolc et al., 2018). Therefore, our experimental strains are indeed representative of decades of intensive genetic selection.

More recently, focus has been placed on extending the laying cycle in an effort to achieve 500 eggs by 100 woa (van Sambeek, 2010). Although commercial flocks have been reported to meet this milestone as early as 2017 (Hendrix-Genetics, 2017), this is the first research study characterizing this achievement in a modern strain. When comparing the persistency of lay, Shaver hens displayed a progressive decline in production at a faster rate than Lohmann hens during the standard laying period of 52-weeks. Succeeding this period, this strain rapidly dropped out of lay, followed by an apparent improvement in production rate, attributed to a spontaneous molt at approximately 80 woa. This spontaneous molt also explains the inconsistent increases and decreases in production in Smoky Joe hens, while a rapid decline occurred following peak productivity. Zakaria et al. (1983) suggested that lower production in the later phases of lay resulted from a decline in growing follicle numbers. In fact, age has been demonstrated to slow the follicular growth rate during this depletion, slowing the rate of production (Williams and Sharp, 1978; Tanabe et al., 1981; Etches et al., 1983; Moudgal and Razdan, 1985; Johnson et al., 1986; Joyner et al., 1987). While this was found to be true for Smoky Joe hens with 4–5 hierarchical follicles present during late-lay, Shaver hens maintained six hierarchical follicles throughout the study despite a decline in production. Interestingly, Lohmann hens demonstrated an increased number of small follicles throughout the production cycle. This suggests that the follicular pool, rather than the number of growing hierarchical follicles, may be responsible for the extended laying persistency. As a result, the average cumulative egg production for Lohmann hens was 515 eggs per hen with 38 non-consecutive pause days throughout 100 woa. This is consistent with the current breeding programs aiming at reducing the number of pause days, combined with an increase in clutch length, in order to improve persistency (Reddy et al., 2016; Wolc et al., 2018).

To the best of our knowledge, no study has previously investigated the possible role of DBPs on egg laying persistency. Here, we report that the decline in OPN5 mRNA levels by the end of the study was not associated with laying status in any strain; therefore, this photoreceptor does not appear to be linked to photorefractoriness. Furthermore, as the mRNA levels of VA-Opsin remained relatively constant throughout the life of these hens, it is possible that this DBP plays a critical role in the prevention of photorefractoriness, or rather the maintenance of photosensitivity, allowing for the extension of the laying cycle in the presence of stimulatory photoperiods. In the case of Smoky Joe hens, despite undergoing a spontaneous molt, the initiation of the secondary laying cycle occurred without a decrease in daylength, generally required to dissipate photorefractoriness (Dawson and Goldsmith, 1989). Thereby, it is possible that these maintained mRNA levels of VA-Opsin act in support of continued HPG axis stimulation in Lohmann hens, while supporting re-entry into lay and renewed HPG axis stimulation in Smoky Joe hens. It has previously been suggested that reduced levels of GnRH-I mRNA are associated with a decreased laying persistency (Nicholls et al., 1988). While a decline in GnRH-I mRNA was hypothesized to mediate the decline in ovarian function associated with reduced production rates at end of lay, this was only found to be true in incubating hens, while aging hens did not demonstrate any changes (Sharp et al., 1992; Dunn et al., 1996; Ciccone et al., 2005), consistent with our results. As there were also no differences observed in GnIH mRNA levels during the prolonged laying period in any of the strains, it is unlikely that this hypothalamic input contributed to the persistency. Interestingly, at the level of pituitary gland, GnIH-R mRNA levels tended to elevate toward the end of the study in Shaver and Smoky Joe hens, for which production was declining and spontaneous molt was observed. However, this was not the case for Lohmann hens, with GnIH-R mRNA levels remaining significantly lower than those immediately prior to maturation. Thus, maintaining a low pituitary sensitivity to GnIH rather than higher hypothalamic GnIH gene expression may be critical to laying persistency. Interestingly, rather than declining toward the end of lay as previously reported (Shimizu and Bedecarrats, 2006), levels of GnRH-RIII mRNA in Lohmann hens were found to increase by the end of the study, while they remained unchanged in Shaver and Smoky Joe hens. This finding further reinforces the hypothesized importance of the pituitary sensitivity to these hypothalamic neuropeptides (Bédécarrats et al., 2009).

These alterations in pituitary sensitivity are hypothesized to have resulted in the fluctuations in FSH mRNA levels seen in Lohmann hens with elevations at 20, 60, and 100 woa. Interestingly, it has been hypothesized that inadequate production of FSH in aging hens reduces the ovarian function and follicular growth, leading to an overall decline in production (Palmer and Bahr, 1992; Ciccone et al., 2005). Consistent with this hypothesis, Shaver and Smoky Joe hens displayed only one elevation in FSH mRNA at the time of sexual maturation, with a decline thereafter, concomitant with a rapid decline in production. As FSH controls the maturation of the ovary, individual E_2_ profiles from each strain were analyzed. Surprisingly, numerous recurrent increases in plasma E_2_ were observed in Lohmann hens, with a greater magnitude than those typically reported for daily ovulatory cycle fluctuations (Ogawa, 1977; Johnson and van Tienhoven, 1980; Tanabe et al., 1981). As a matter of fact, we hypothesize that the elevations in FSH mRNA seen in Lohmann hens may be responsible for the recurrent increases in E_2_ observed throughout the laying cycle for this strain. Although previous studies have found that reduced circulating E_2_ levels increase LH-β mRNA expression (Terada et al., 1997) and that a decline in mRNA levels was associated with a decrease in egg production (Sharp et al., 1992), this was not observed in the present study. Taken together, our results suggest that while LH-β mRNA levels are critical to the maturation process, there is no apparent input of this gonadotrophin during the extended laying period.

As noted above, the re-occurrence of elevations in E_2_ observed in these strains varied significantly. While Tanabe et al. (1981) previously observed a second elevation in E_2_ at 1 year of age, such late elevations have typically only been reported in hens after undergoing molt and immediately prior to the initiation of the second laying cycle (Braw-Tal et al., 2004). Nonetheless, it was recently reported that a secondary elevation in E_2_ with an amplitude similar to the initial functional peak can be observed at 52 woa in the Lohmann LSL-lite, the same strain as used the present study (Baxter and Bédécarrats, 2019). Since blood samples in the aforementioned study were collected monthly, no recurrent increases could be detected, whereas the biweekly sampling paradigm in the present study allowed us to capture a cyclical pattern of circulating E_2_ concentrations. Although individual hen profiles were not synchronized, introducing individual variability within this dataset, smoothed curves were able to predict a higher frequency in these recurrent elevations of E_2_ in Lohmann hens compared to the other strains, with five peaks in total. Interestingly, individual variability was found to be the lowest for Lohmann hens, with peaks occurring over a period of 3–5 weeks. In contrast, while both Shaver and Smoky Joe hens displayed three peaks during the study, individual variability was greater. It is also important to note that these recurrent increases in E_2_ observed in Smoky Joe hens were linked to spontaneous molting. In addition to frequency, the amplitude of the peaks in E_2_ was also higher for Lohmann hens, especially during the initiation of lay. As peak E_2_ concentrations have been positively correlated with overall egg production (Onagbesan et al., 2006), this could additionally contribute to the extended laying rate. In fact, the larger persistent number of small white follicles observed in Lohmann hens is indicative of either a larger pool of follicles initially recruited, resulting in a significantly higher peak E_2_ concentration, or additional successive recruitment waves throughout the cycle, possibly mediated by FSH.

Overall, this study suggests that our initial models describing the HPG axis (Bédécarrats, 2015; Bedecarrats et al., 2016) may not be fully applicable to modern commercial layer strains. While sexual maturation has been previously identified to be dependent on photo-schedule, the “64-g window” reported in this study strongly suggests a link between body weight and the AFE, likely *via* a metabolic trigger (Hanlon et al., 2020). This is further reinforced by evidence of a de-synchronization in OPN5 mRNA levels between the strains, with Lohmann hens demonstrating an earlier elevation in levels during the immature pullet phase. Additionally, increased selection pressure for early production in Lohmann hens is associated with higher GnRH-I mRNA levels during the maturation process, along with a synchronized switch in pituitary sensitivity to hypothalamic neuropeptides. In fact, Lohmann hens demonstrated the earliest functional peak of E_2_ resulting in an earlier AFE and higher peak production. Interestingly, the initiation of the E_2_ elevation occurred prior to PS for both the Lohmann and Shaver hens. With 515 eggs throughout the 100-week duration of the experiment, Lohmann hens significantly outperformed the other strains, further highlighting the gains achieved through genetic selection. While no changes in hypothalamic neuropeptides were determined in any strain during the extended laying period, Lohmann hens demonstrated a simultaneous decline in GnIH-R and elevation in GnRH-RIII, suggesting an improved pituitary sensitivity to stimulatory input. We hypothesize this resulted in additional elevations in FSH mRNA levels, which in turn would support recurrent follicular recruitment and recurrent elevations in E_2_, as observed in Lohmann hens.

## Data Availability Statement

The raw data supporting the conclusions of this article will be made available by the authors, without undue reservation.

## Ethics Statement

The animal study was reviewed and approved by Animal Care Committee at the University of Guelph.

## Author Contributions

CH and GB designed the study. CH and KT carried out this study. CH was the primary writer and conducted the analyses with the support of KT and GB. GB participated in the redaction of the manuscript and supported CH throughout the project as supervisor. All authors contributed to the article and approved the submitted version.

### Conflict of Interest

The authors declare that the research was conducted in the absence of any commercial or financial relationships that could be construed as a potential conflict of interest.

## Footnotes

^1^www.myassays.com/arbor-assays-estradiol-eia-kit.assay

^2^https://www.medischebiologie.nl/files/
