# Supplementary material for: Changes in the Control of the Hypothalamic-Pituitary Gonadal Axis Across Three Differentially Selected Strains of Laying Hens (Gallus gallus domesticus)
Source: Front Physiol. 2021 Mar 25;12:651491. doi: 10.3389/fphys.2021.651491 (PMC8027345; doi:10.3389/fphys.2021.651491)
Supplement: Supplementary file 1 [file Table_1.docx]

**Supplemental Table 1.** Diameter and Weight of the Hierarchical Follicles in Lohmann, Shaver, and Smoky Joe hens at 25, 45, 60, 75, and 100 weeks of age (woa).

|  | Strain | Age | Follicle Diameter (mm) | | | | | |  | Follicle Weight (g) | | | | | |
| --- | --- | --- | --- | --- | --- | --- | --- | --- | --- | --- | --- | --- | --- | --- | --- |
|  |  |  | F1^2^ | F2 | F3 | F4 | F5 | F6 |  | F1 | F2 | F3 | F4 | F5 | F6 |
| Strain | Lohmann |  | 32.78^a^ | 29.75^a^ | 26.77^a^ | 22.32^a^ | 17.04^a^ | 13.27 |  | 14.76^a^ | 12.05^a^ | 8.43^a^ | 5.44^a^ | 2.61^a^ | 1.33 |
|  | Shaver |  | 32.33^a^ | 28.85^a^ | 26.11^a^ | 21.59^a^ | 16.08^a^ | 11.99 |  | 14.21^a^ | 10.76^a^ | 7.31^ab^ | 4.47^a^ | 2.06^a^ | 0.81 |
|  | Smoky Joe |  | 29.93^b^ | 26.34^b^ | 20.65^b^ | 15.86^b^ | 13.05^b^ | . |  | 11.73^b^ | 7.94^b^ | 5.35^b^ | 2.02^b^ | 1.04^b^ | . |
| Age^1^ |  | 25 | 29.32^b^ | 26.56^b^ | 23.78 | 21.49 | 16.97 | 12.54 |  | 10.85^b^ | 8.81^b^ | 6.36 | 4 | 2.21 | 0.95 |
|  |  | 45 | 31.19^ab^ | 28.54^ab^ | 23.48 | 20.04 | 15.98 | . |  | 13.03^ab^ | 9.27^ab^ | 7.88 | 3.91 | 1.91 | . |
|  |  | 60 | 33.31^a^ | 30.24^a^ | 27.23 | 21.5 | 16.4 | . |  | 15.22^a^ | 11.54^a^ | 8.6 | 4.66 | 2.14 | . |
|  |  | 75 | 32.21^ab^ | 28.12^ab^ | 23.04 | 18.11 | . | . |  | 14.59^a^ | 10.80^ab^ | 5.55 | 3.5 | . | . |
|  |  | 100 | 31.80^ab^ | 28.10^ab^ | 25.02 | 18.47 | 13 | . |  | 14.14^a^ | 10.80^ab^ | 6.76 | 3.82 | 1.4 | . |
| Strain x Age | Lohmann | 25 | 30.68 | 28.14 | 24.9 | 22.85 | 18.51 | 14.53 |  | 12.72 | 10.41 | 7.67 | 5.31 | 2.99 | 1.75 |
|  |  | 45 | 33.12 | 30.23 | 27.59 | 24.78 | 17.88 | 11.99 |  | 15.28 | 11.72 | 8.18 | 6.42 | 2.41 | 0.79 |
|  |  | 60 | 35.34 | 31.06 | 29.43 | 22.04 | 18.29 | 12.91 |  | 17.38 | 13.38 | 10.43 | 6.08 | 3.05 | 1.23 |
|  |  | 75 | 32.04 | 27.78 | 24.37 | 20.24 | 15.12 | 13.79 |  | 14.65 | 10.79 | 6.84 | 4.43 | 2.59 | 1.6 |
|  |  | 100 | 32.72 | 31.53 | 27.57 | 19.71 | 15.4 | 13.15 |  | 15.59 | 13.94 | 9.06 | 4.99 | 1.99 | 1.27 |
|  | Shaver | 25 | 29.84 | 26.3 | 23.87 | 22.8 | 17.28 | 11.61 |  | 10.89 | 8.6 | 5.88 | 4.67 | 2.21 | 0.75 |
|  |  | 45 | 33.48 | 30.52 | 28.96 | 21.75 | 17.54 | 12.48 |  | 15.2 | 10.82 | 8.92 | 3.96 | 2.44 | 0.77 |
|  |  | 60 | 32.61 | 31.24 | 28.26 | 22.64 | 17.04 | 12.29 |  | 14.43 | 11.83 | 9.28 | 5.17 | 2.23 | 1 |
|  |  | 75 | 33.68 | 30.16 | 25.78 | 22.17 | 15.62 | 11.81 |  | 16.26 | 12.59 | 6.05 | 5.27 | 1.94 | 0.8 |
|  |  | 100 | 32.06 | 26.03 | 23.7 | 18.61 | 12.9 | 11.75 |  | 1.27 | 9.94 | 6.43 | 3.31 | 1.5 | 0.74 |
|  | Smoky Joe | 25 | 27.44 | 25.24 | 22.56 | 18.83 | 15.12 | 11.47 |  | 8.96 | 7.42 | 5.54 | 2.03 | 1.43 | 0.35 |
|  |  | 45 | 28.65 | 24.86 | 13.91 | 13.6 | 12.52 | . |  | 10.43 | 5.29 | 6.53 | 1.36 | 0.86 | . |
|  |  | 60 | 31.99 | 28.43 | 23.99 | 17.83 | 13.87 | . |  | 13.85 | 9.43 | 6.1 | 2.73 | 1.15 | . |
|  |  | 75 | 30.94 | 26.43 | 18.98 | 11.92 | . | . |  | 12.85 | 9.01 | 3.77 | 0.79 | . | . |
|  |  | 100 | 30.64 | 26.75 | 23.81 | 17.11 | 10.67 | . |  | 12.57 | 8.53 | 4.79 | 3.17 | 0.71 | . |
| Sources of variation | | | *P-value .* | | | | | | | | | | | | |
| Age | | | 0.0086 | 0.024 | 0.0629 | 0.1407 | 0.055 | 0.996 |  | 0.0002 | 0.0272 | 0.0834 | 0.7796 | 0.6734 | 0.9256 |
| Strain | | | 0.0234 | 0.0047 | 0.0001 | <0.0001 | 0.0129 | 0.5158 |  | 0.0021 | <0.0001 | 0.0093 | 0.003 | 0.0228 | 0.1263 |
| Age x Strain | | | 0.6874 | 0.1536 | 0.0201 | 0.4727 | 0.9839 | 0.9372 |  | 0.4144 | 0.2419 | 0.8965 | 0.6868 | 0.9967 | 0.8551 |

^1^ Age in weeks

^2^ F1-F6 = Follicle 1 to Follicle 6

^a,b^ LSMeans of the strains within each trait lacking a common superscript are different (P<0.05).
