# Supplementary material for: Changes in the Control of the Hypothalamic-Pituitary Gonadal Axis Across Three Differentially Selected Strains of Laying Hens (Gallus gallus domesticus)
Source: Front Physiol. 2021 Mar 25;12:651491. doi: 10.3389/fphys.2021.651491 (PMC8027345; doi:10.3389/fphys.2021.651491)
Supplement: Supplementary file 2 [file Data_Sheet_1.pdf]

Supplemental Figure 1. Individual Estradiol (E_2) profiles from 12 to 100 weeks of age (woa) presented as a plasma concentration (pg/µL). Each figure is labelled with the strain and ID number of the hen.

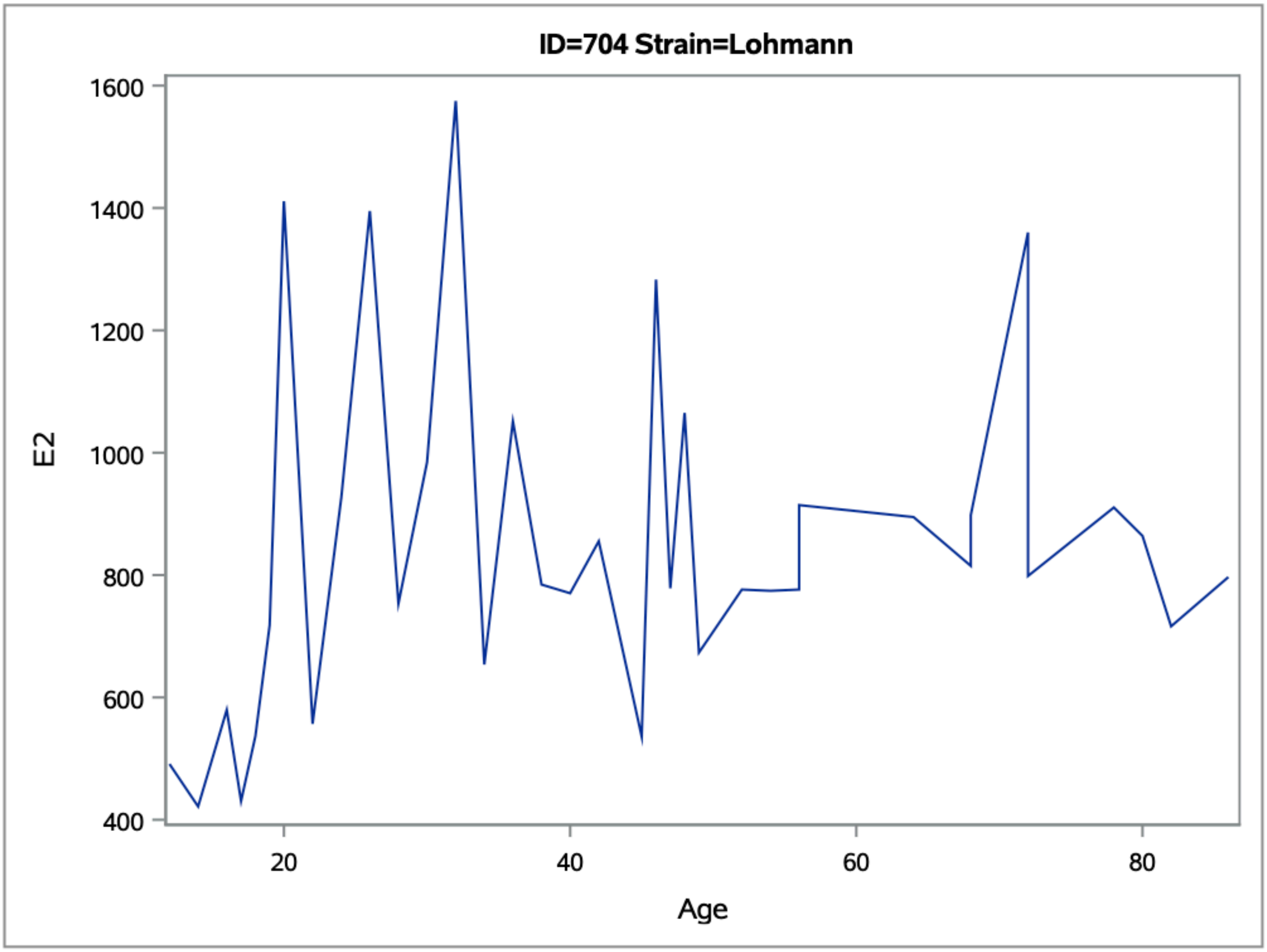
ID=704 Strain=Lohmann
E2
1600
1400
1200
1000
800
600
400
20
40
60
80
Age

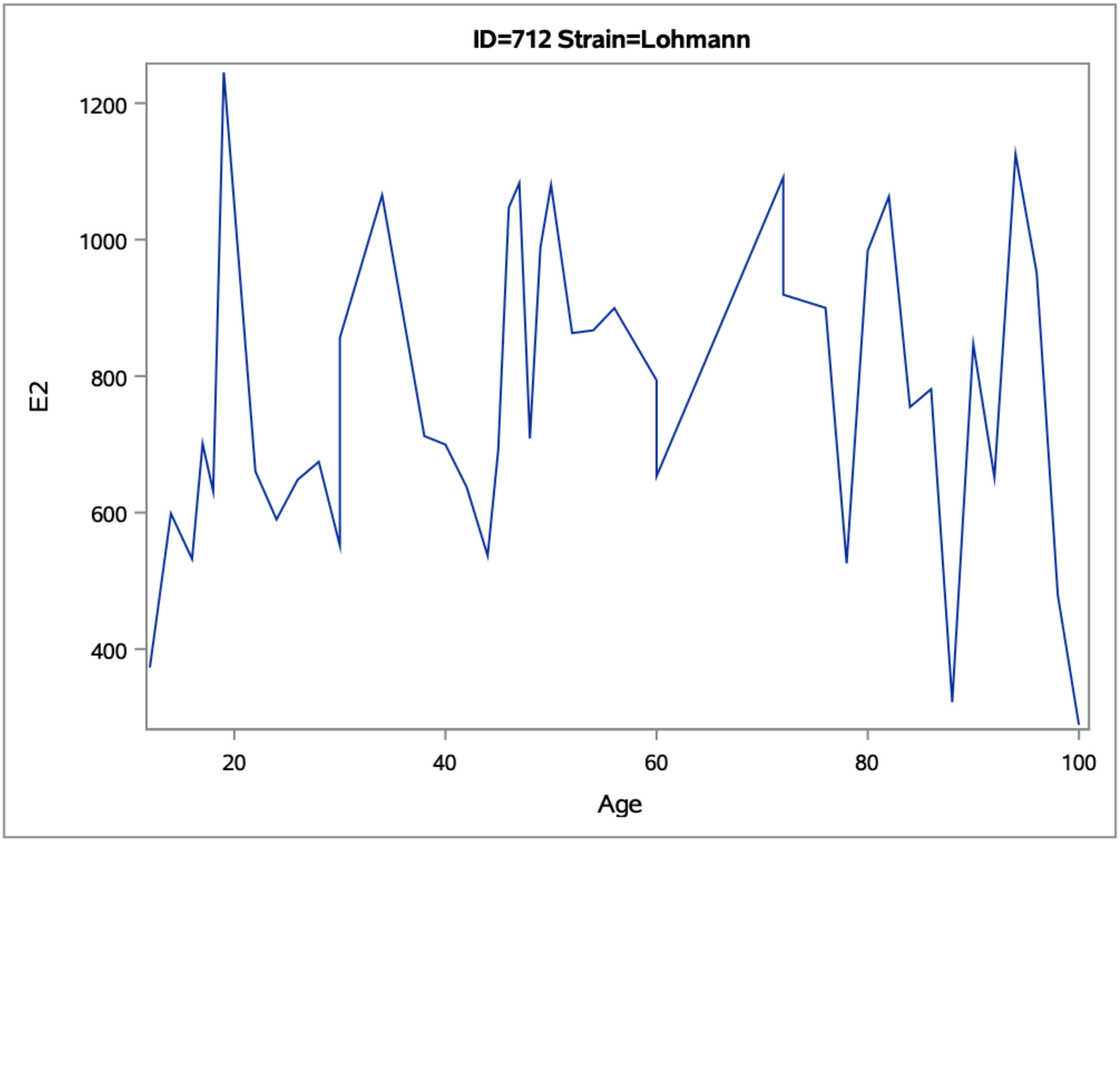
ID=712 Strain=Lohmann
E2
1200
1000
800
600
400
20
40
60
80
100
Age

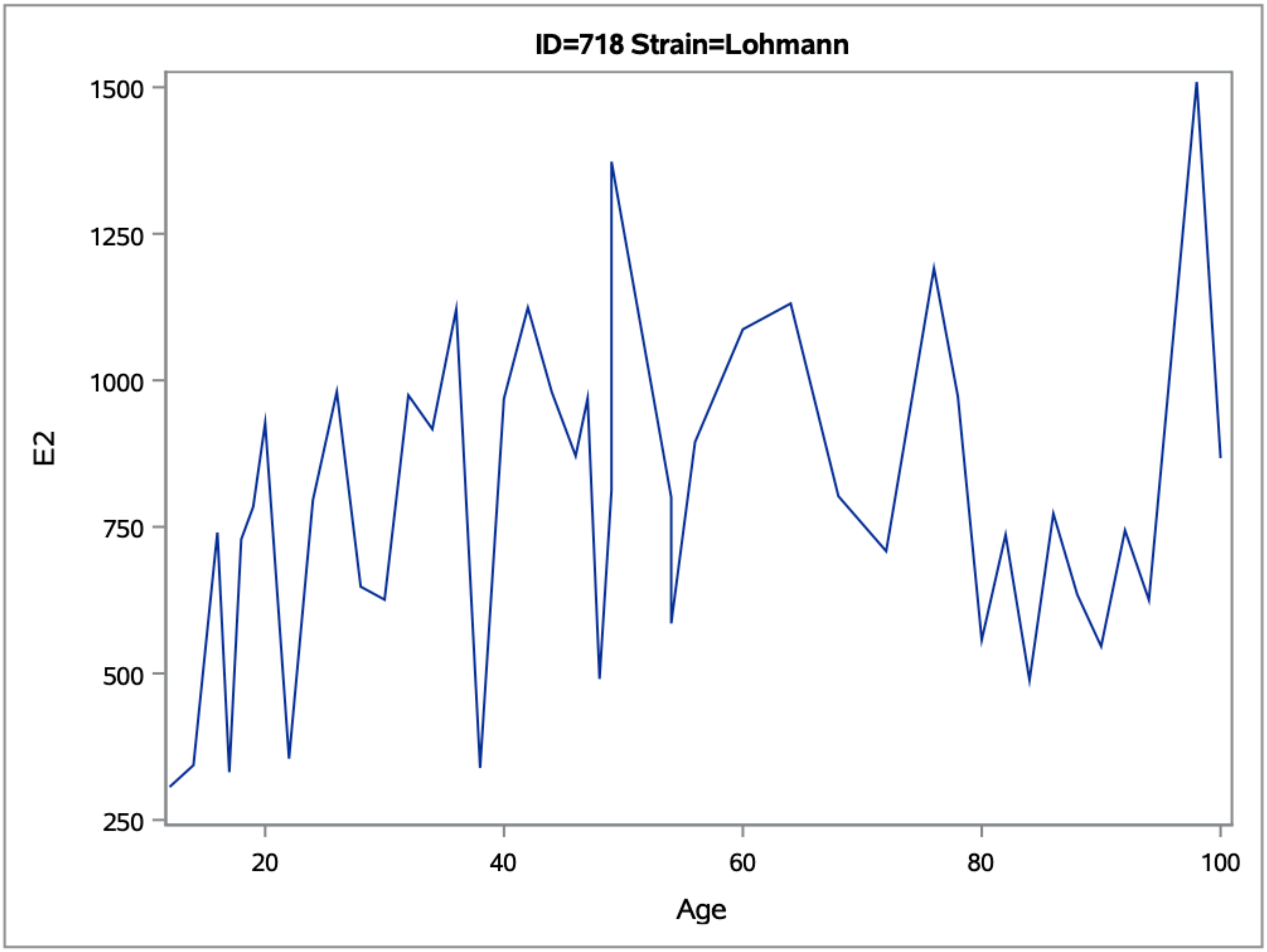
ID=718 Strain=Lohmann
1500
1250
1000
750
500
250
E2
20
40
60
80
100
Age

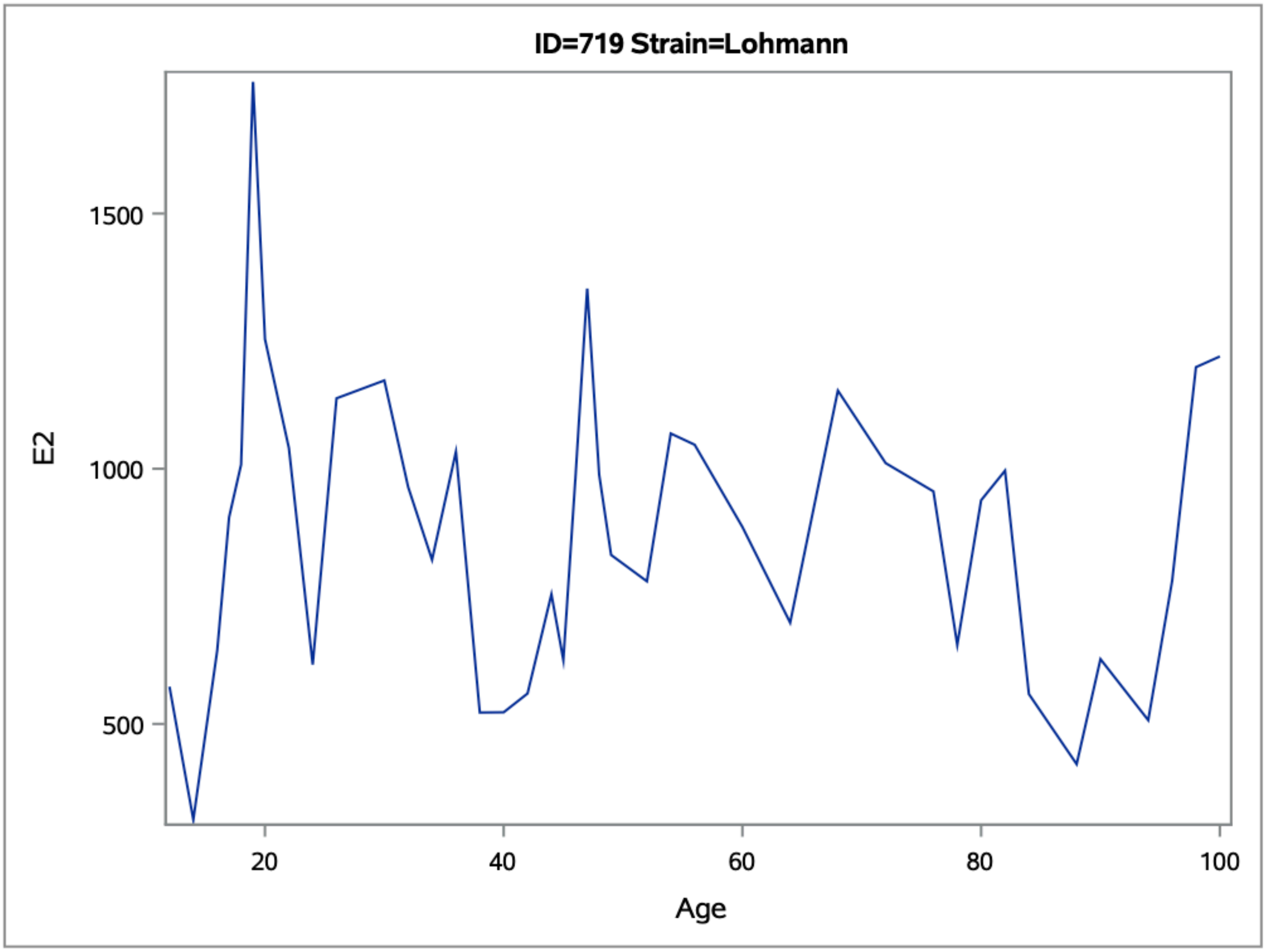
ID=719 Strain=Lohmann
E2
1500
1000
500
20
40
60
80
100
Age

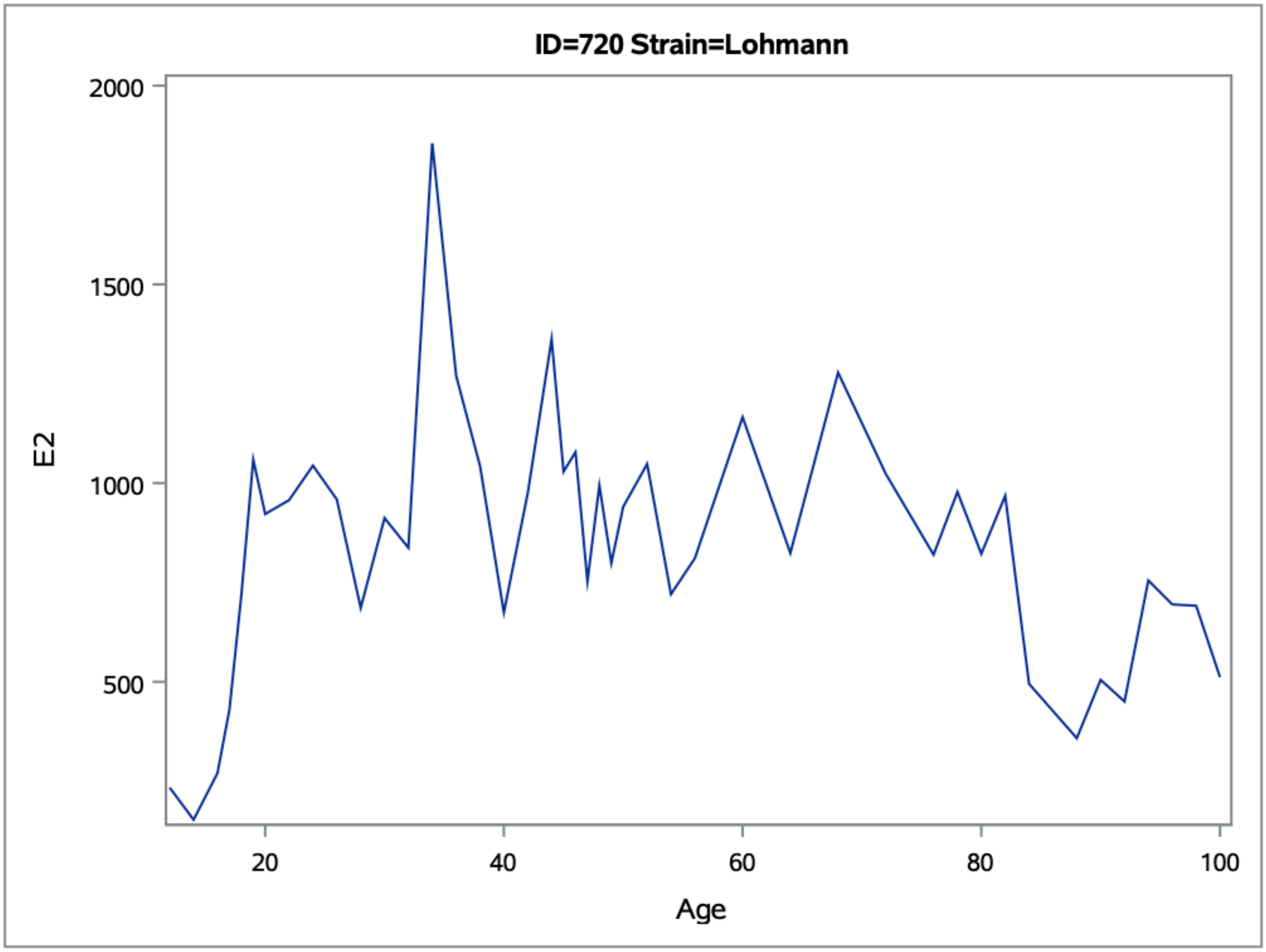
ID=720 Strain=Lohmann
2000
1500
1000
500
E2
20
40
60
80
100
Age

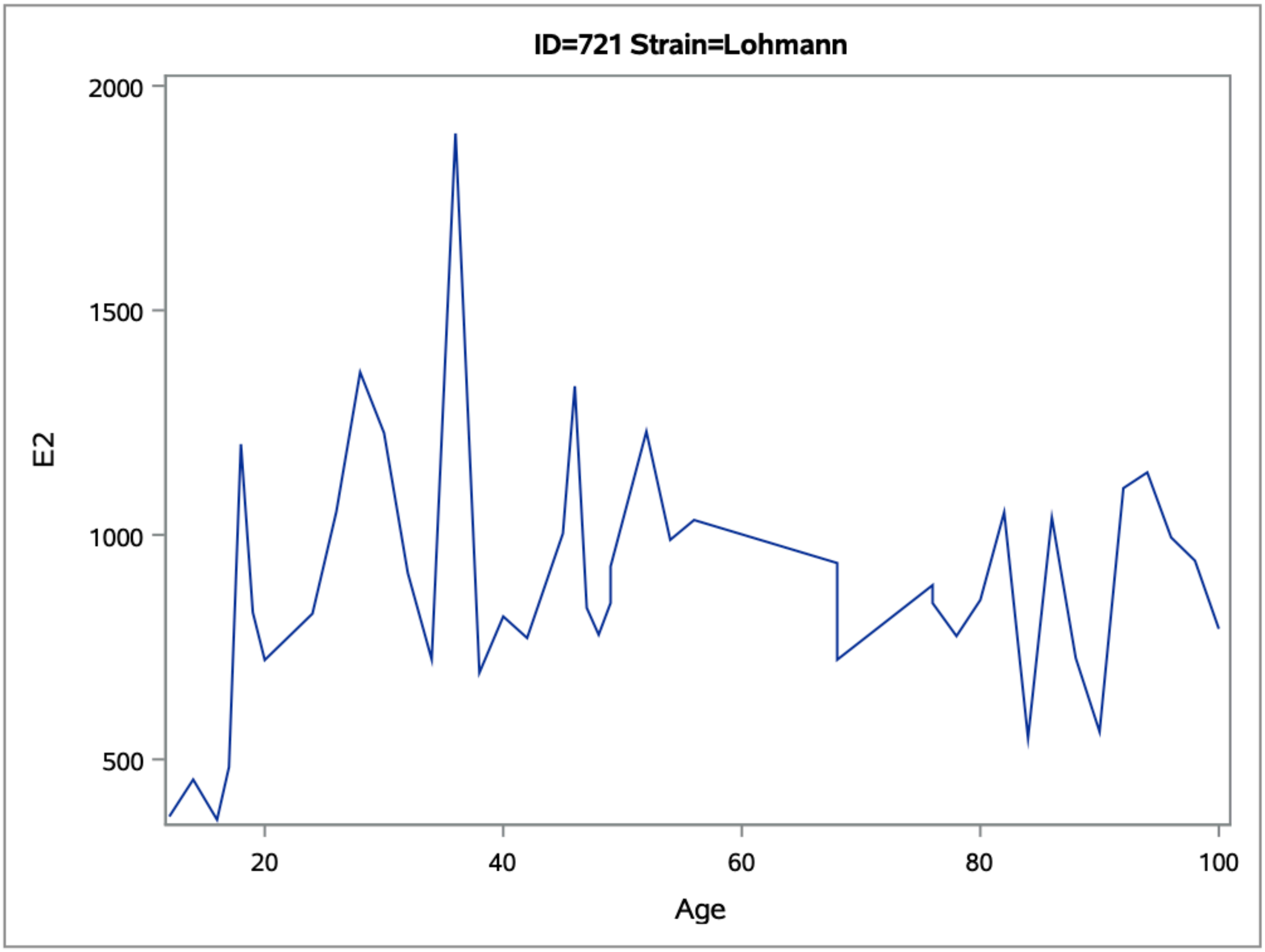
ID=721 Strain=Lohmann
2000
1500
1000
500
E2
20
40
60
80
100
Age

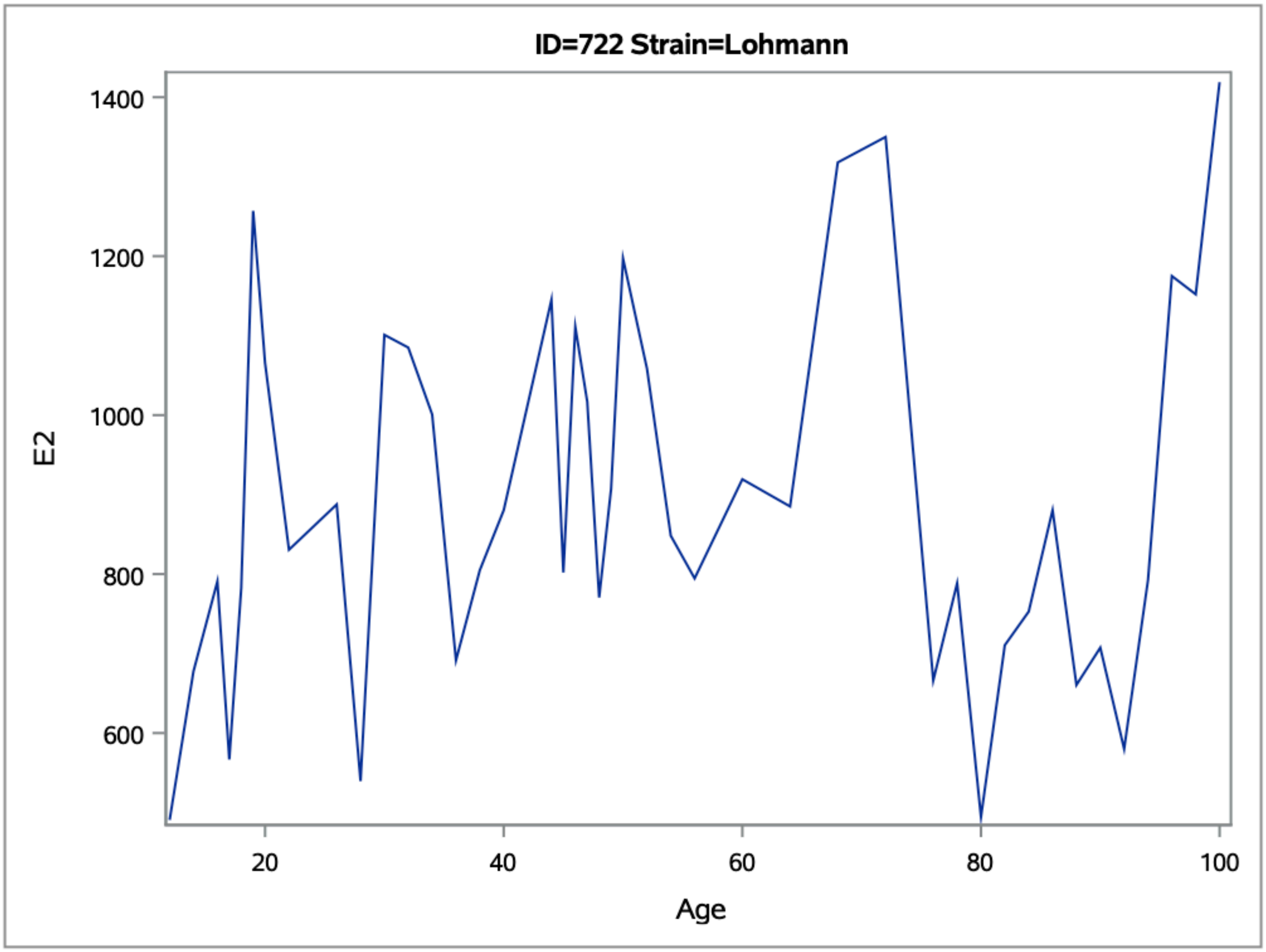

ID=722 Strain=Lohmann
1400
1200
1000
800
600
E2
20
40
60
80
100
Age

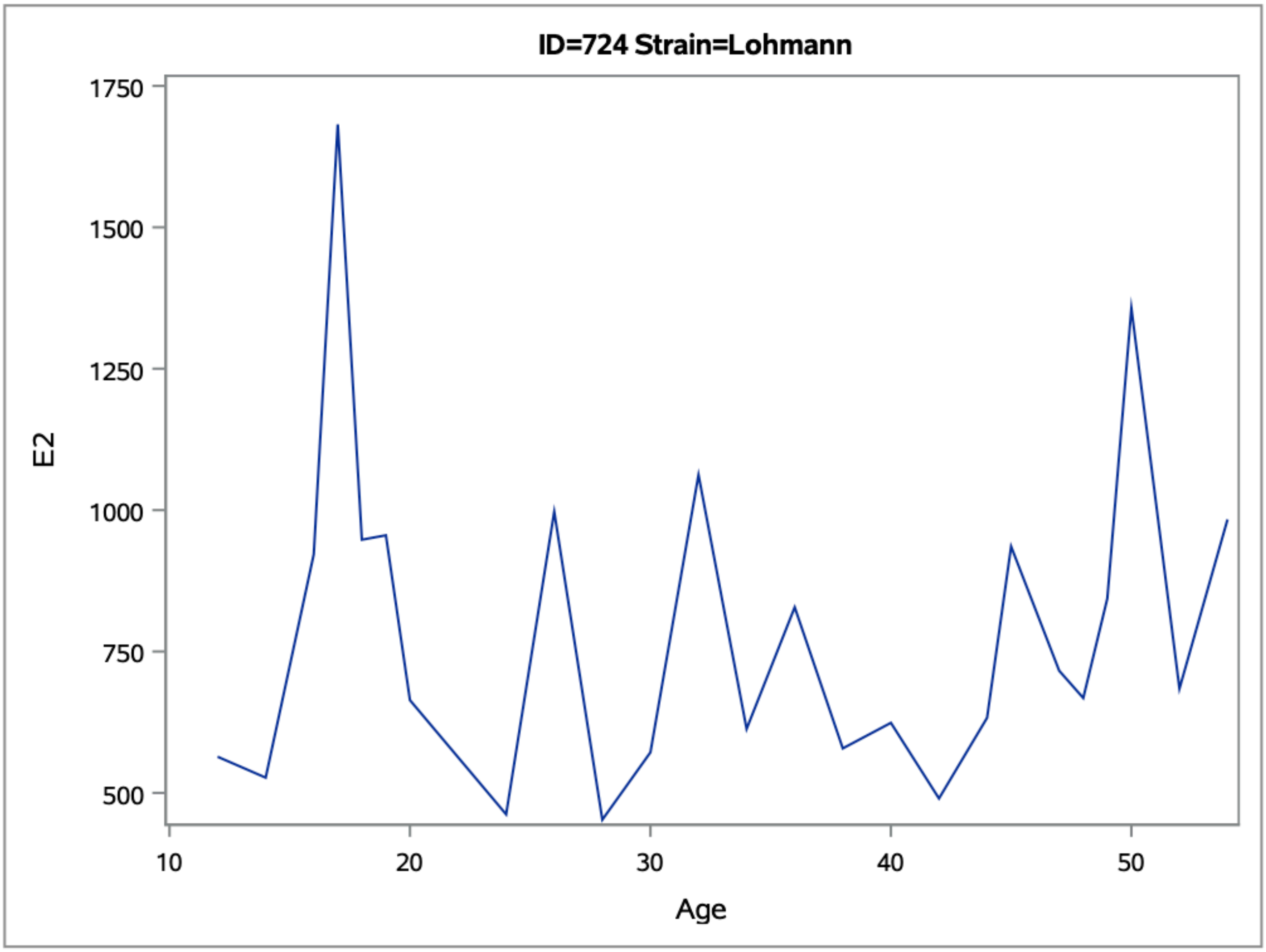
ID=724 Strain=Lohmann
1750
1500
1250
1000
750
500
E2
10
20
30
40
50
Age

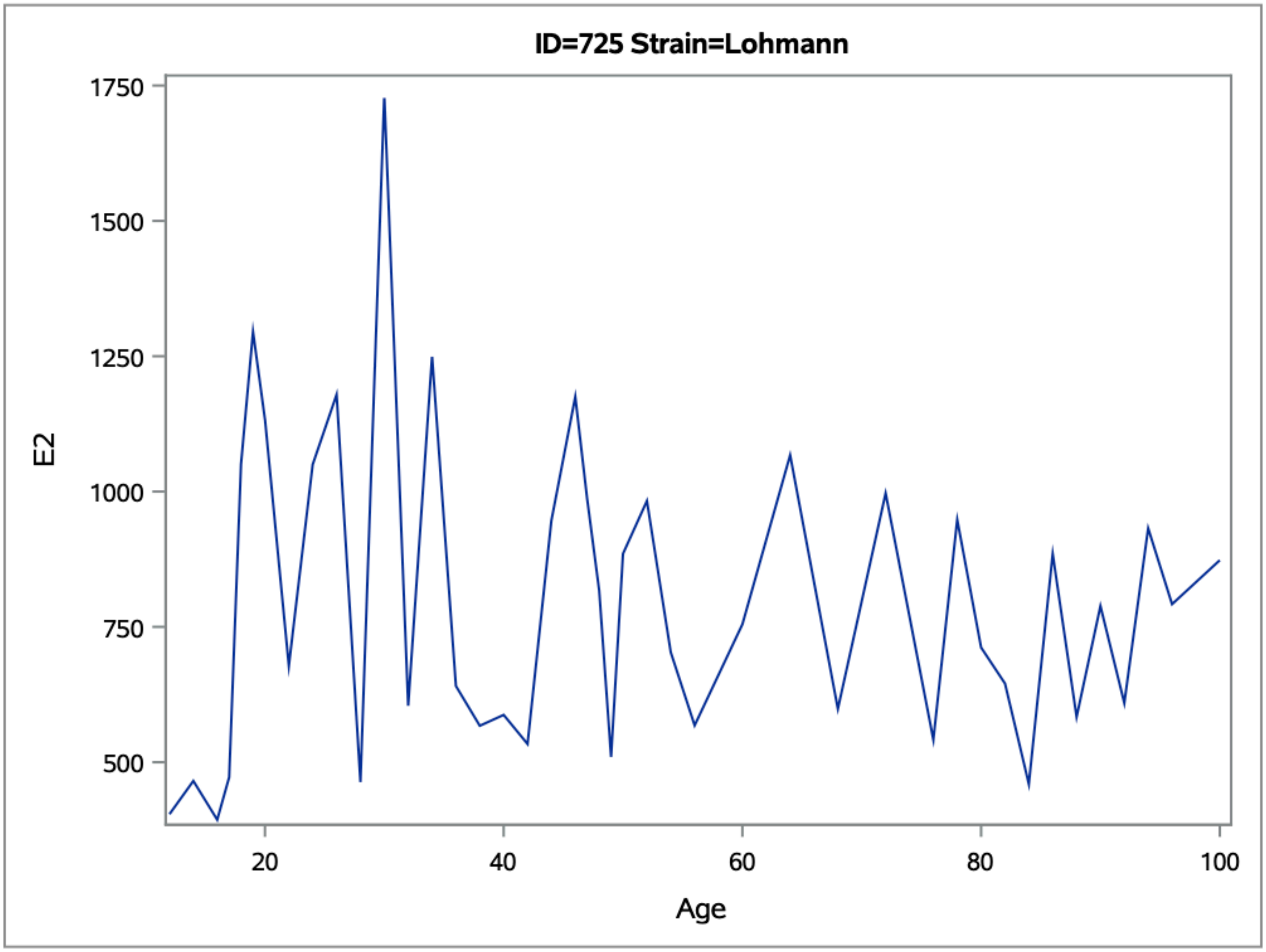

ID=725 Strain=Lohmann
1750
1500
1250
1000
750
500
E2
20
40
60
80
100
Age

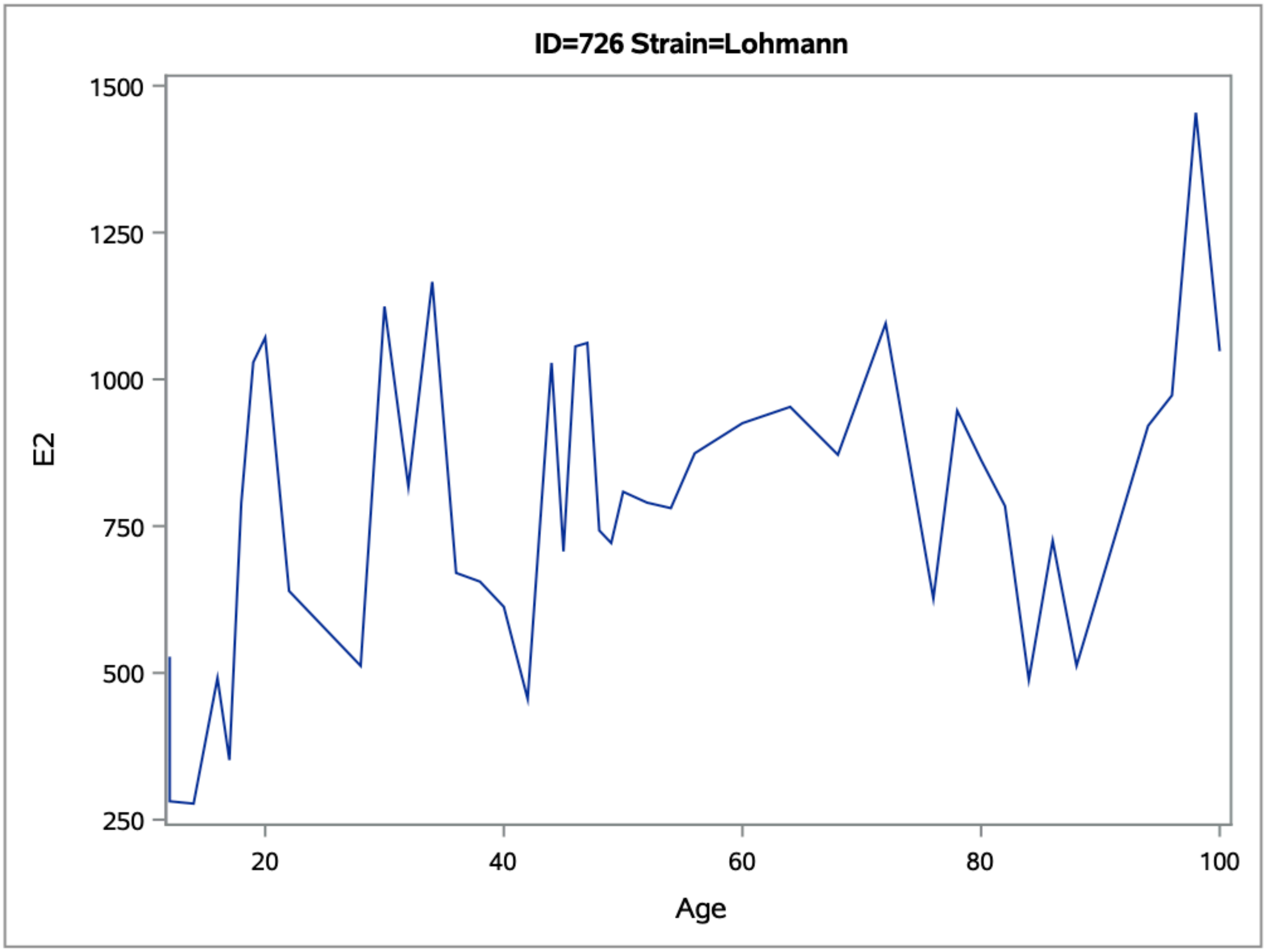
ID=726 Strain=Lohmann
1500
1250
1000
750
500
250
E2
20
40
60
80
100
Age

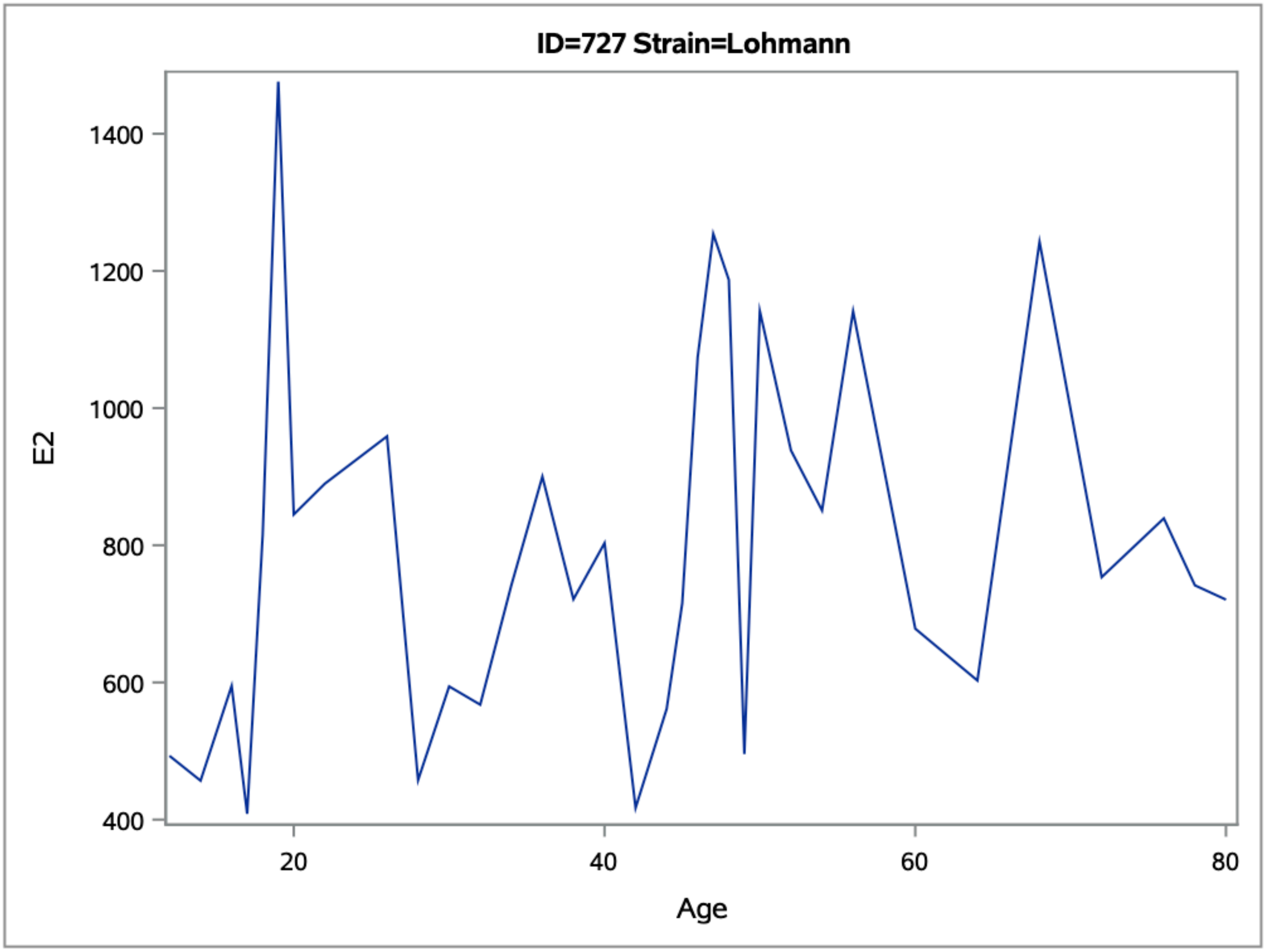
ID=727 Strain=Lohmann
E2
1400
1200
1000
800
600
400
20
40
60
80
Age

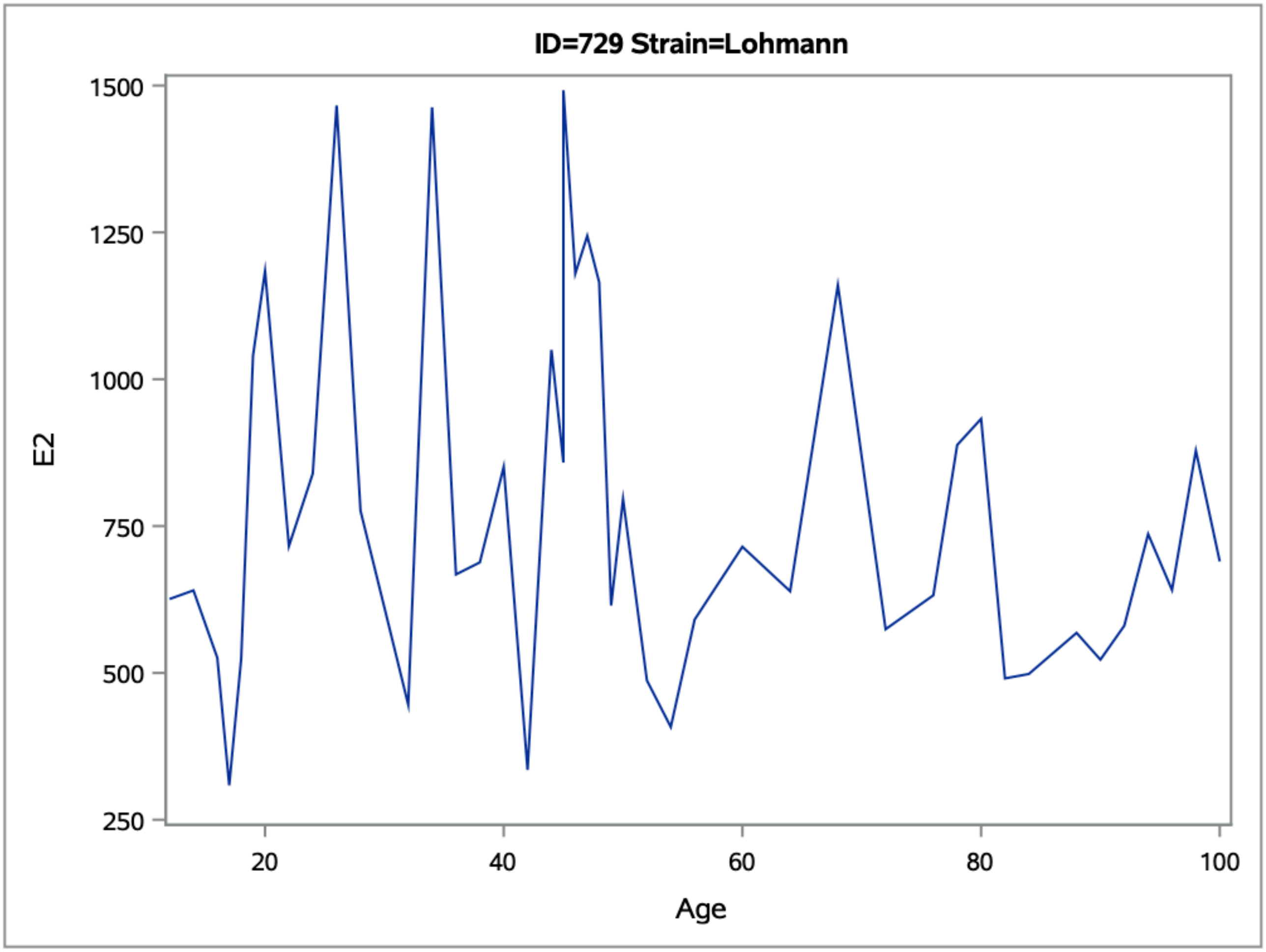

ID=729 Strain=Lohmann
E2
1500
1250
1000
750
500
250
20
40
60
80
100
Age

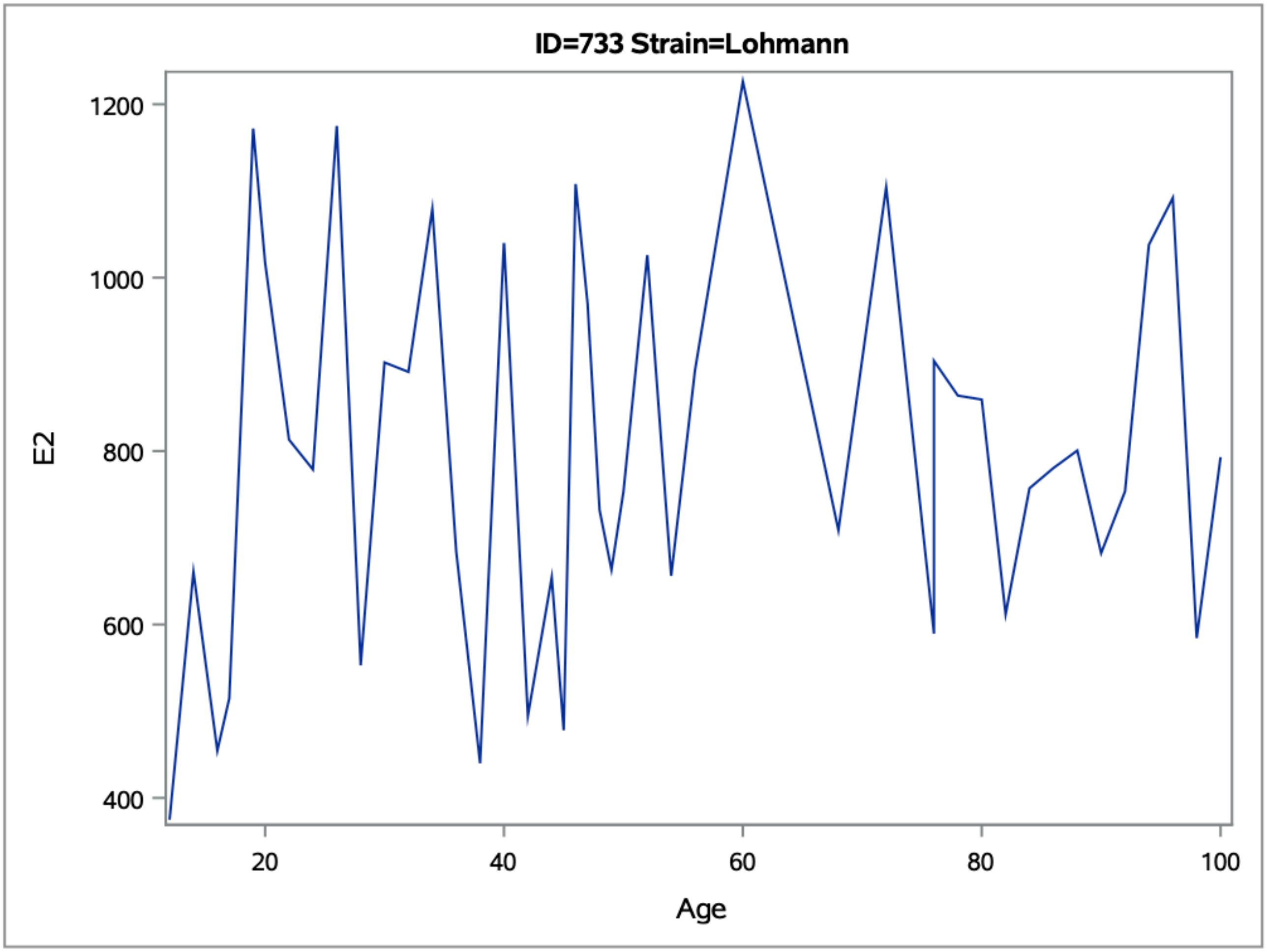
ID=733 Strain=Lohmann
E2
1200
1000
800
600
400
20
40
60
80
100
Age

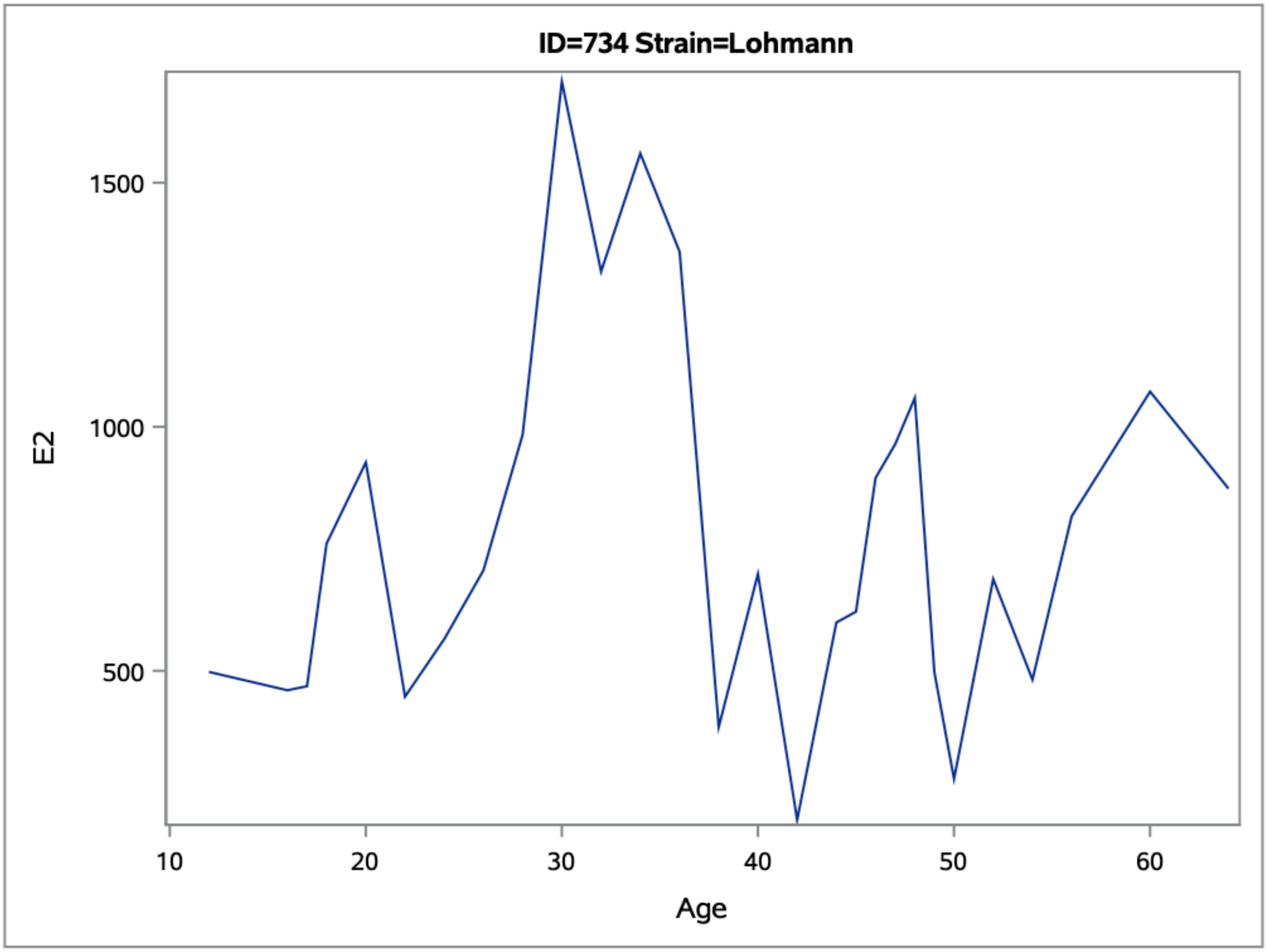
ID=734 Strain=Lohmann
E2
1500
1000
500
10
20
30
40
50
60
Age

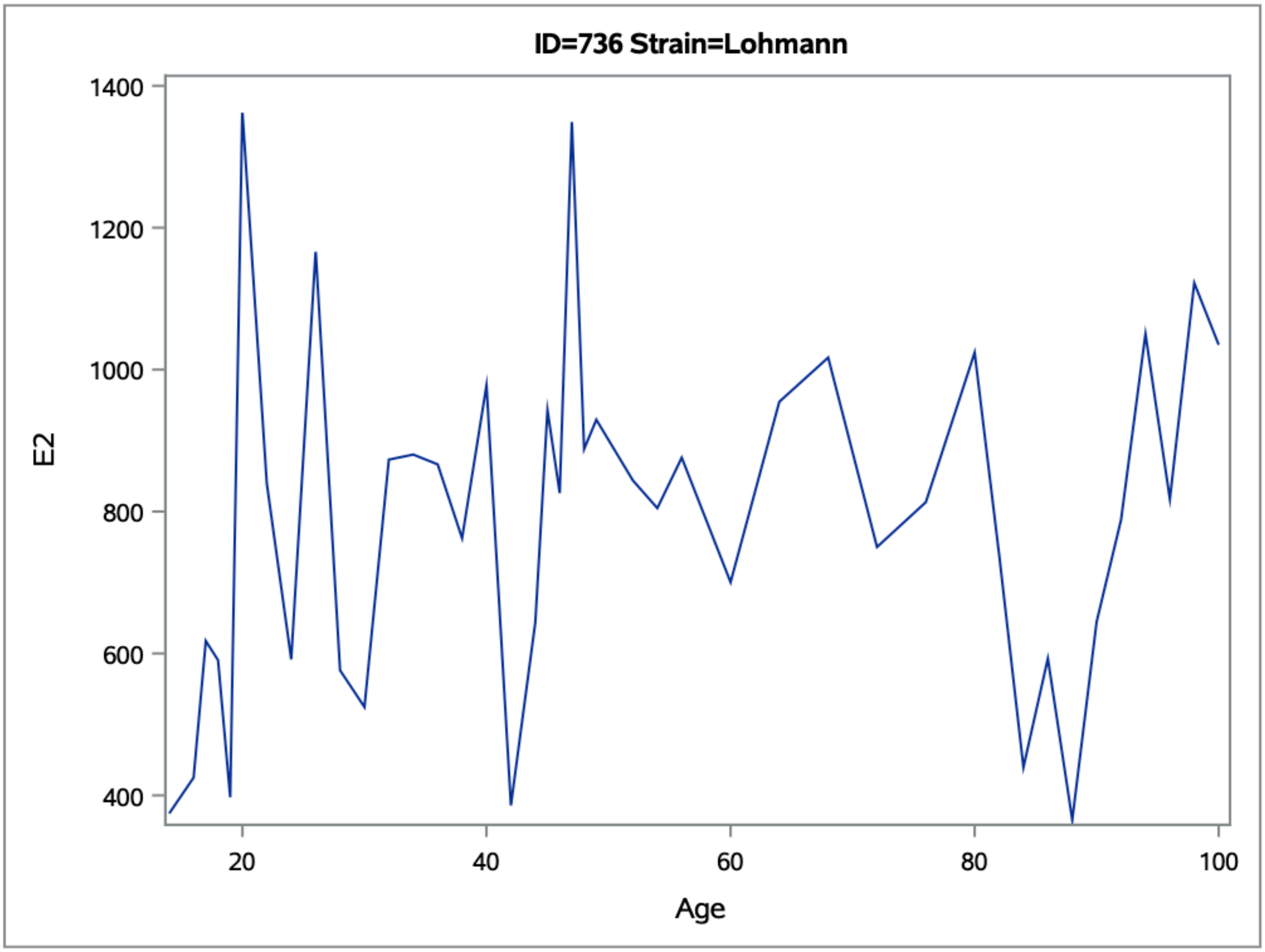

ID=736 Strain=Lohmann
1400
1200
1000
800
600
400
E2
20
40
60
80
100
Age

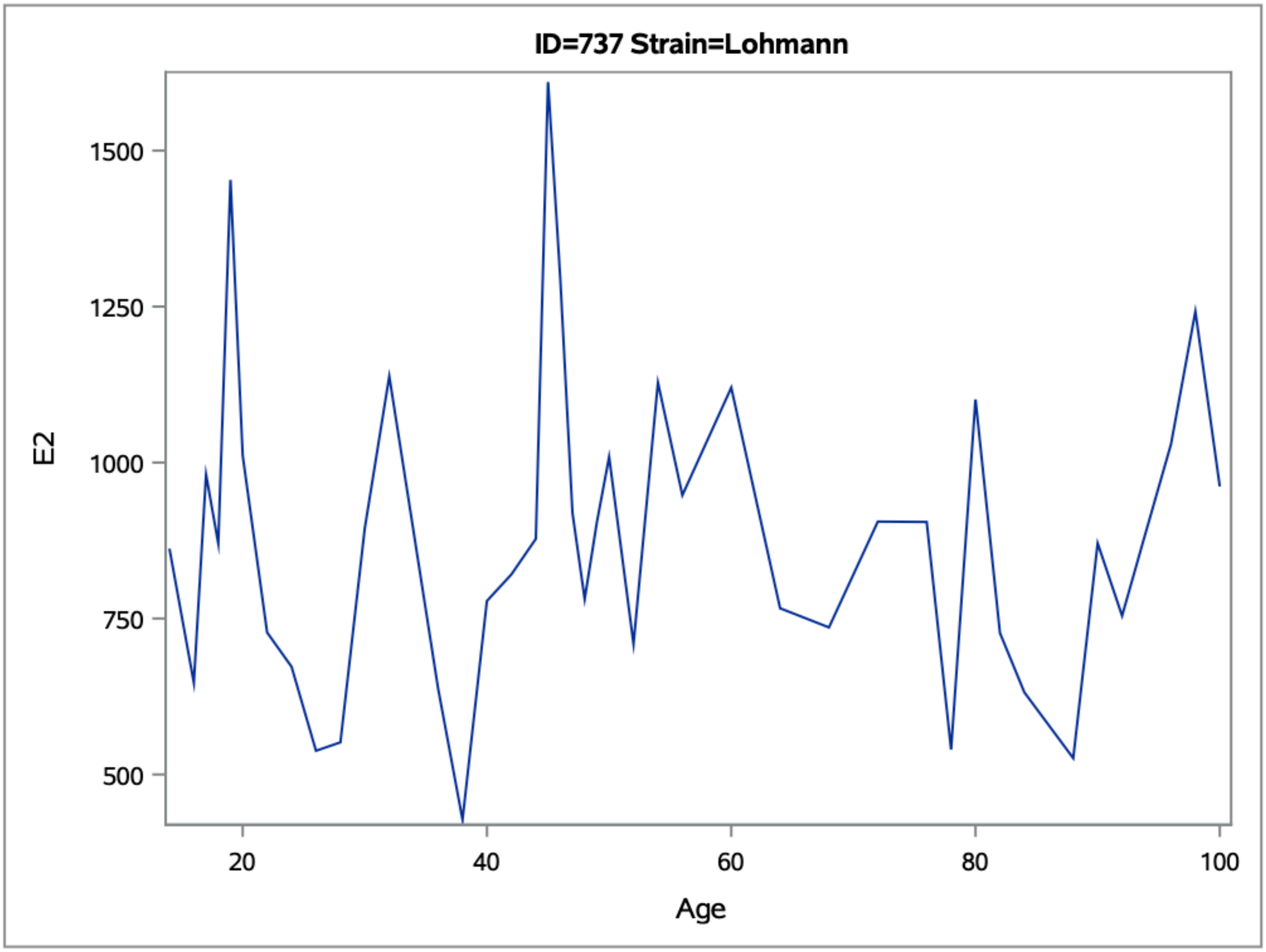
ID=737 Strain=Lohmann
E2
1500
1250
1000
750
500
20
40
60
80
100
Age

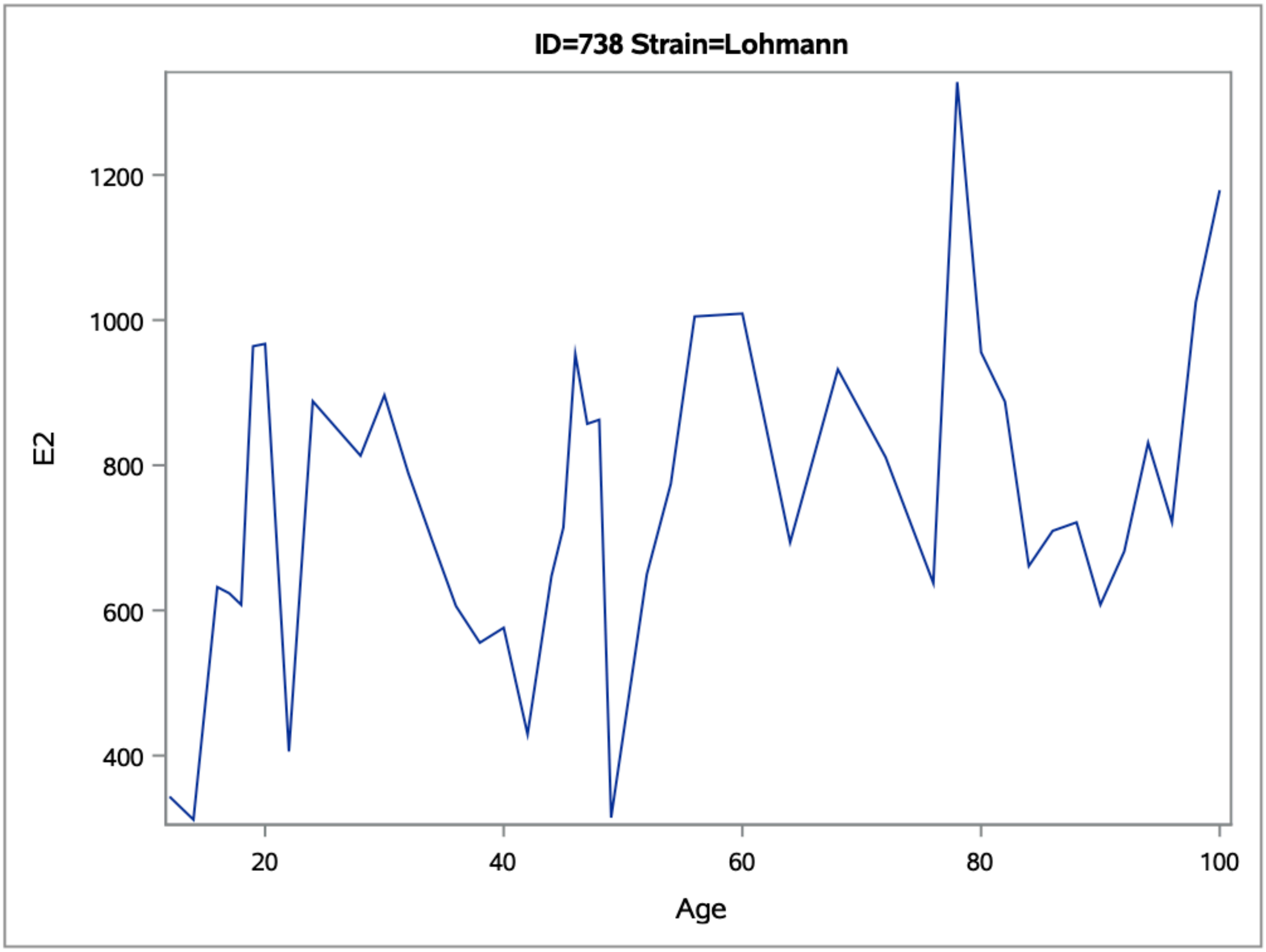
ID=738 Strain=Lohmann
E2
1200
1000
800
600
400
20
40
60
80
100
Age

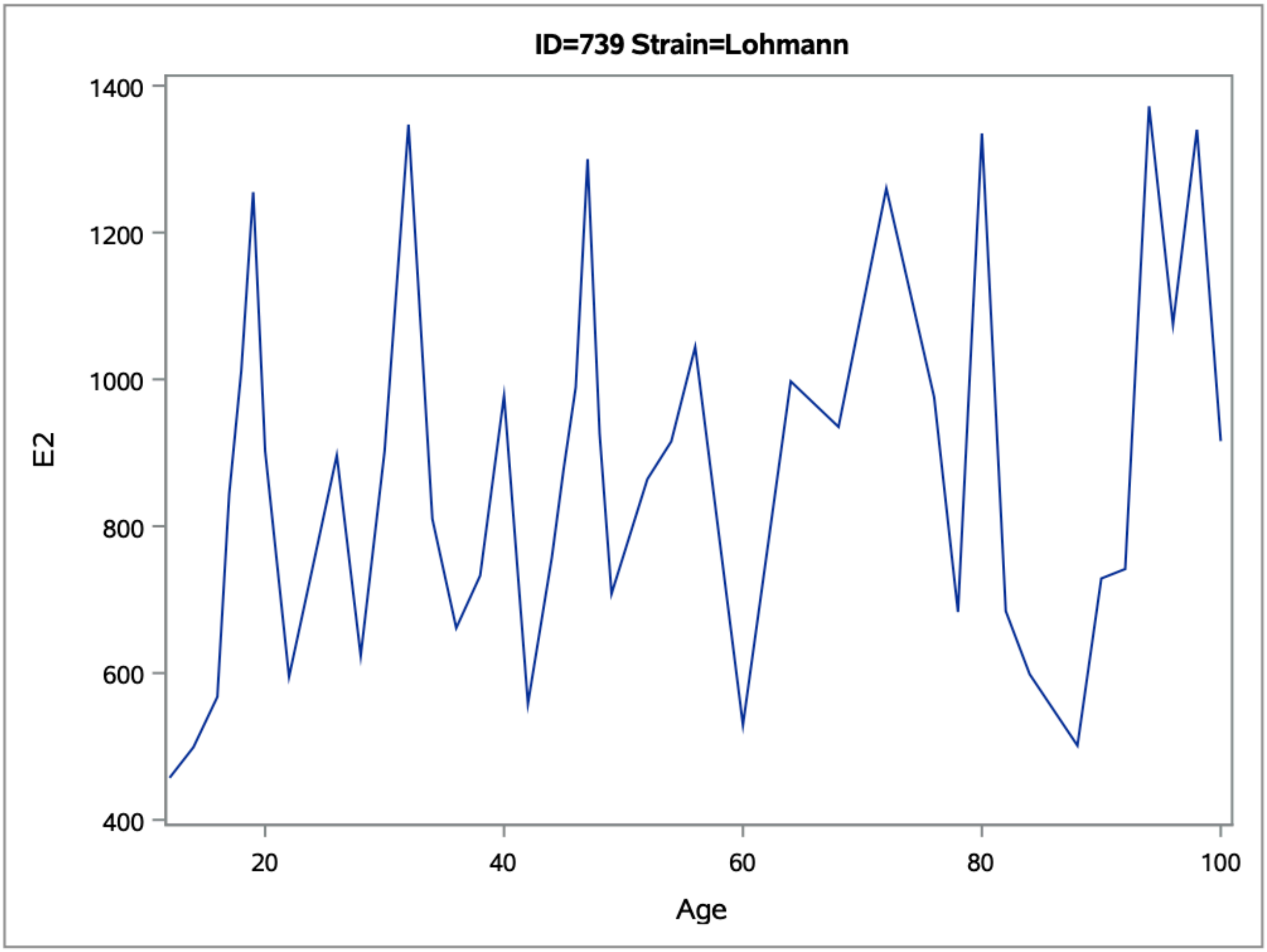
ID=739 Strain=Lohmann
1400
1200
1000
800
600
400
E2
20
40
60
80
100
Age

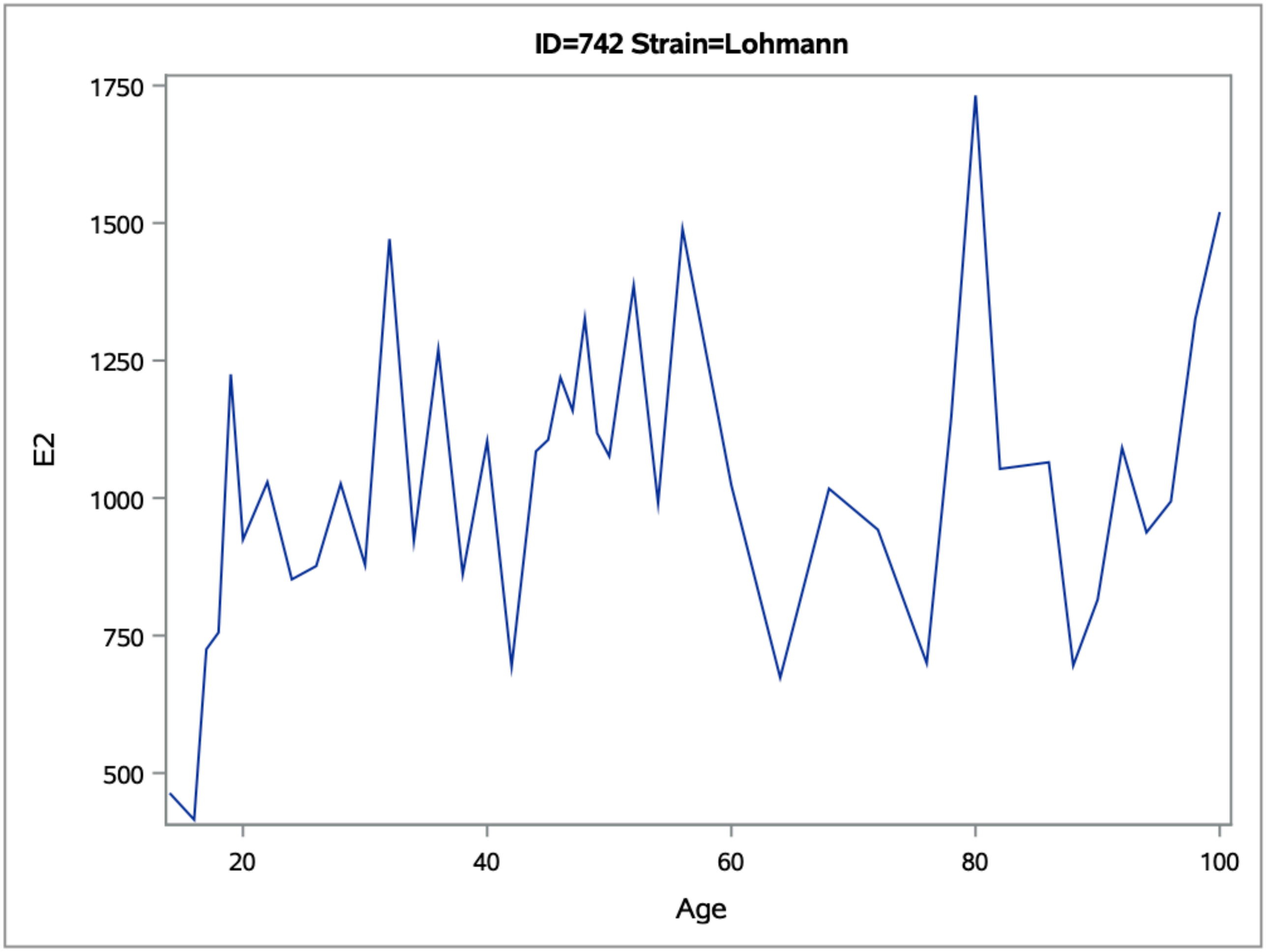
ID=742 Strain=Lohmann
1750
1500
1250
1000
750
500
E2
20
40
60
80
100
Age

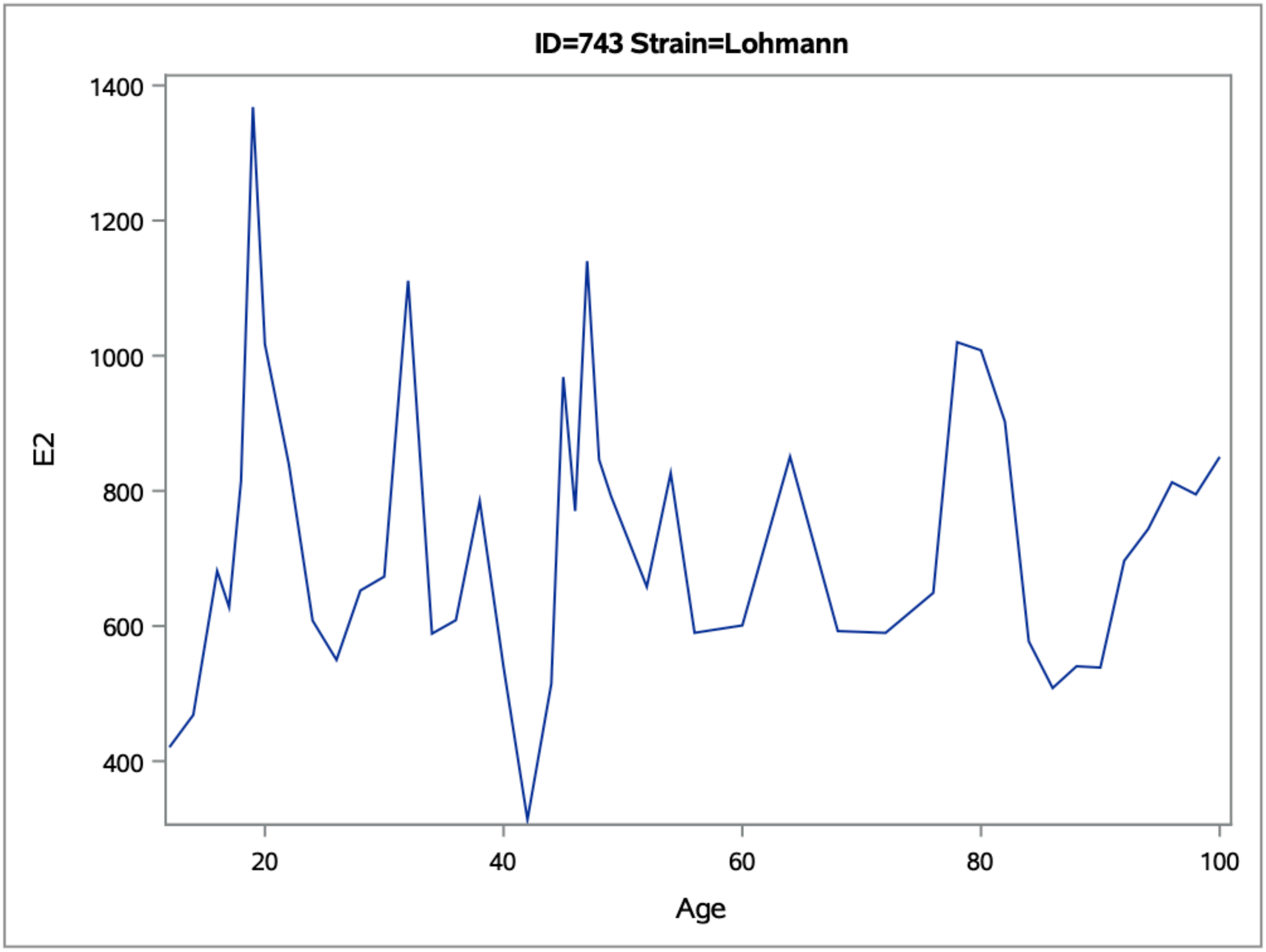

ID=743 Strain=Lohmann
1400
1200
1000
800
600
400
E2
20
40
60
80
100
Age

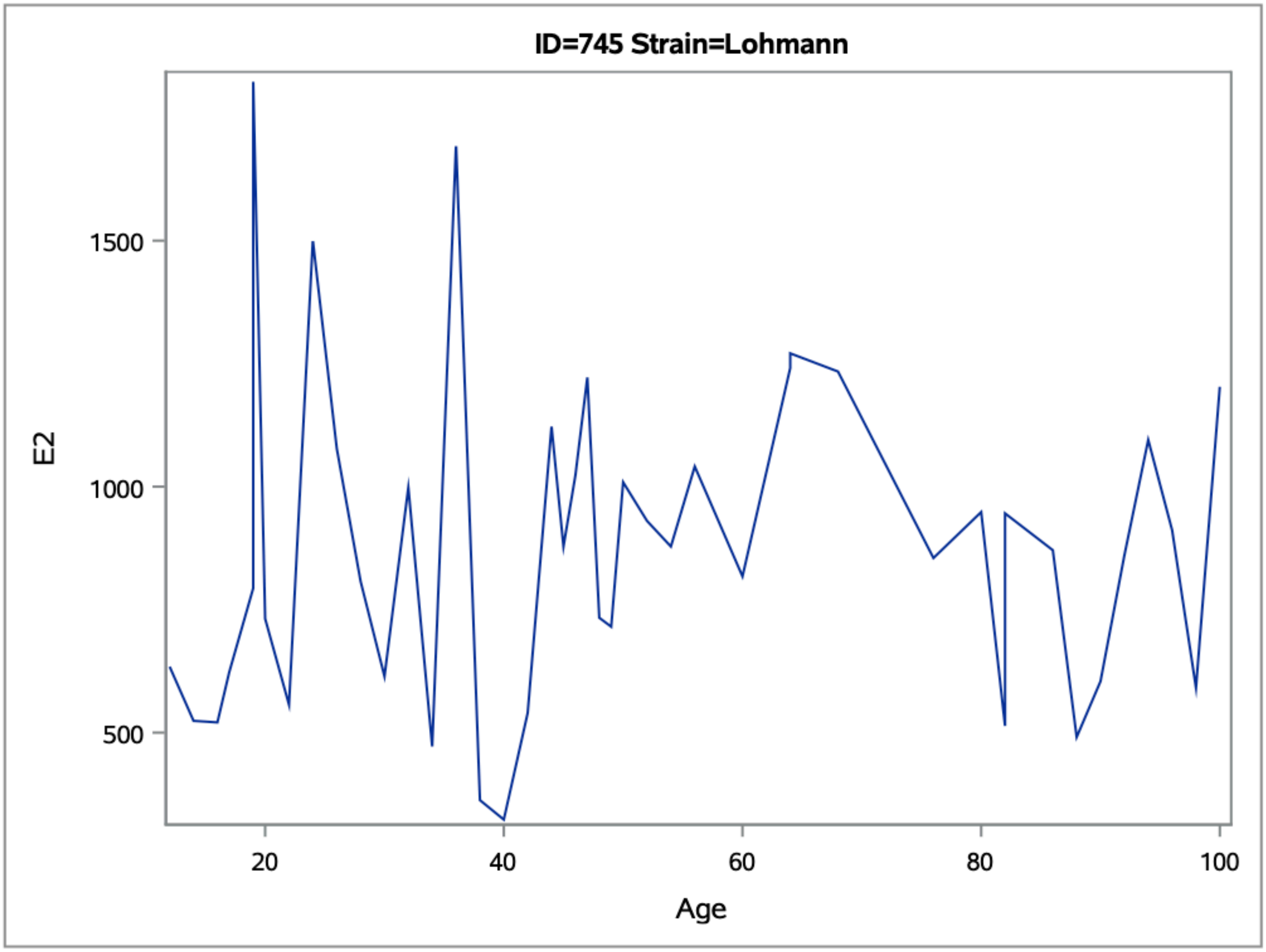
ID=745 Strain=Lohmann
E2
1500
1000
500
20
40
60
80
100
Age

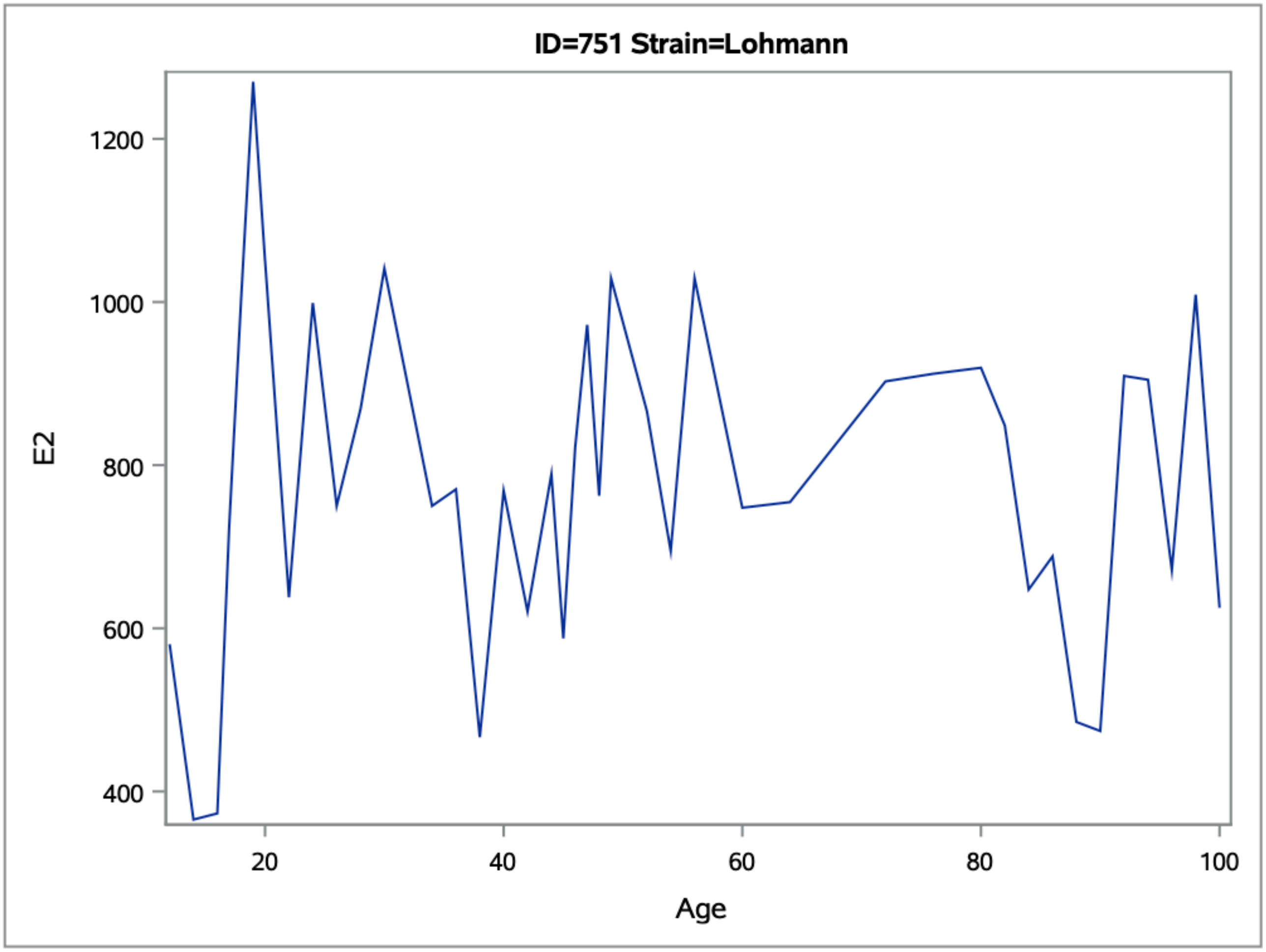
ID=751 Strain=Lohmann
E2
1200
1000
800
600
400
20
40
60
80
100
Age

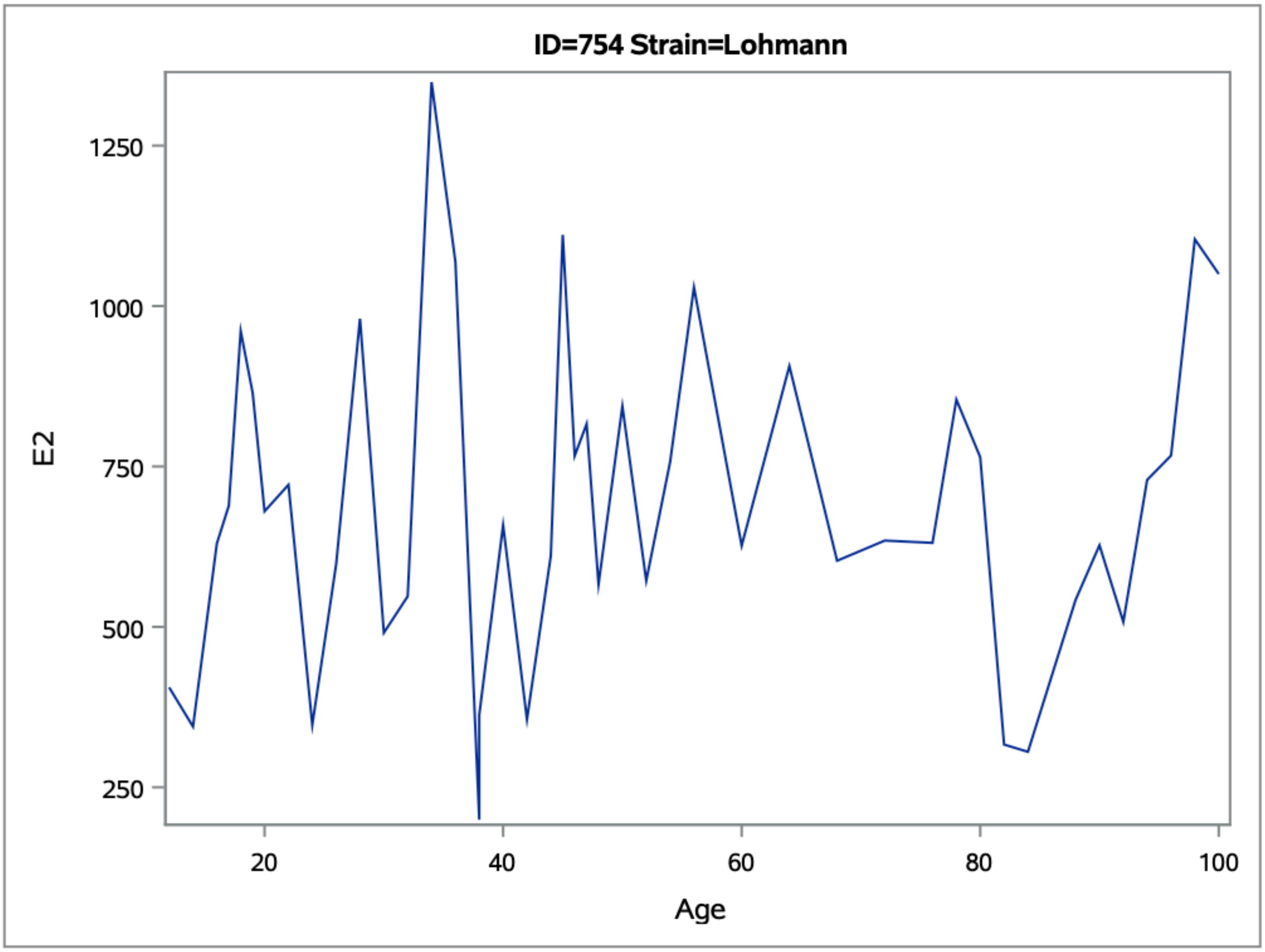
ID=754 Strain=Lohmann
E2
1250
1000
750
500
250
20
40
60
80
100
Age

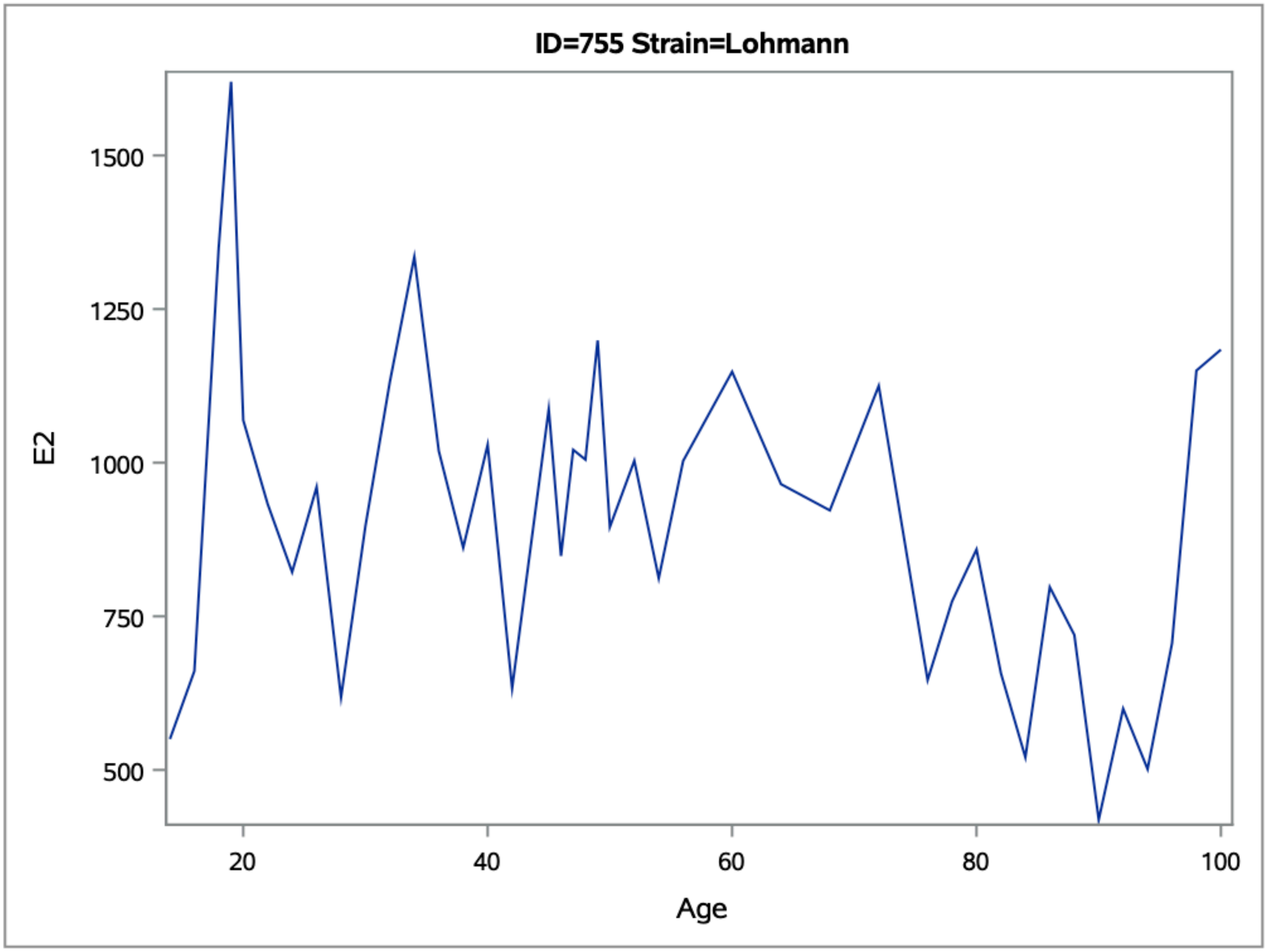

ID=755 Strain=Lohmann
1500
1250
1000
750
500
E2
20
40
60
80
100
Age

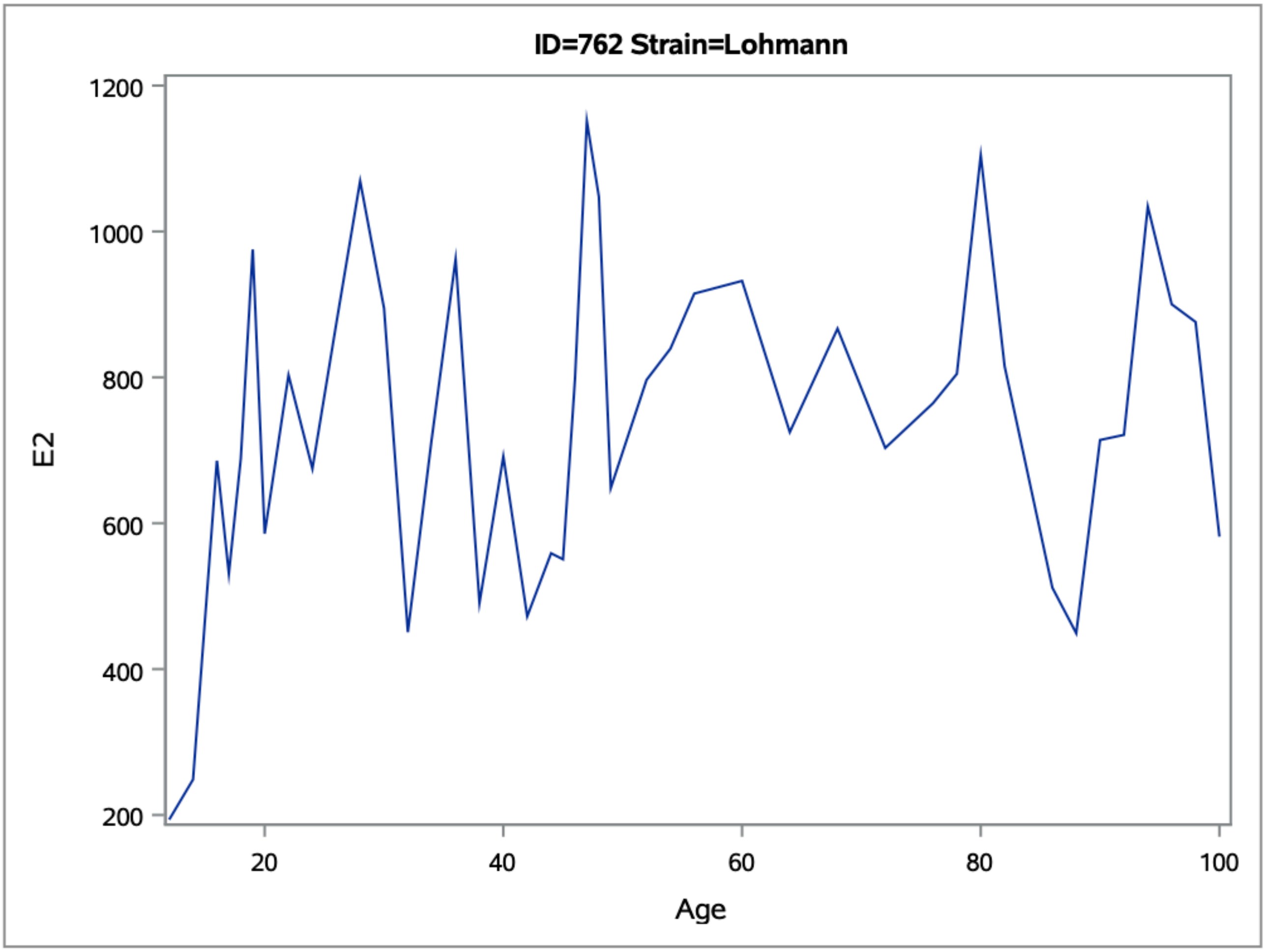
ID=762 Strain=Lohmann
E2
1200
1000
800
600
400
200
20
40
60
80
100
Age

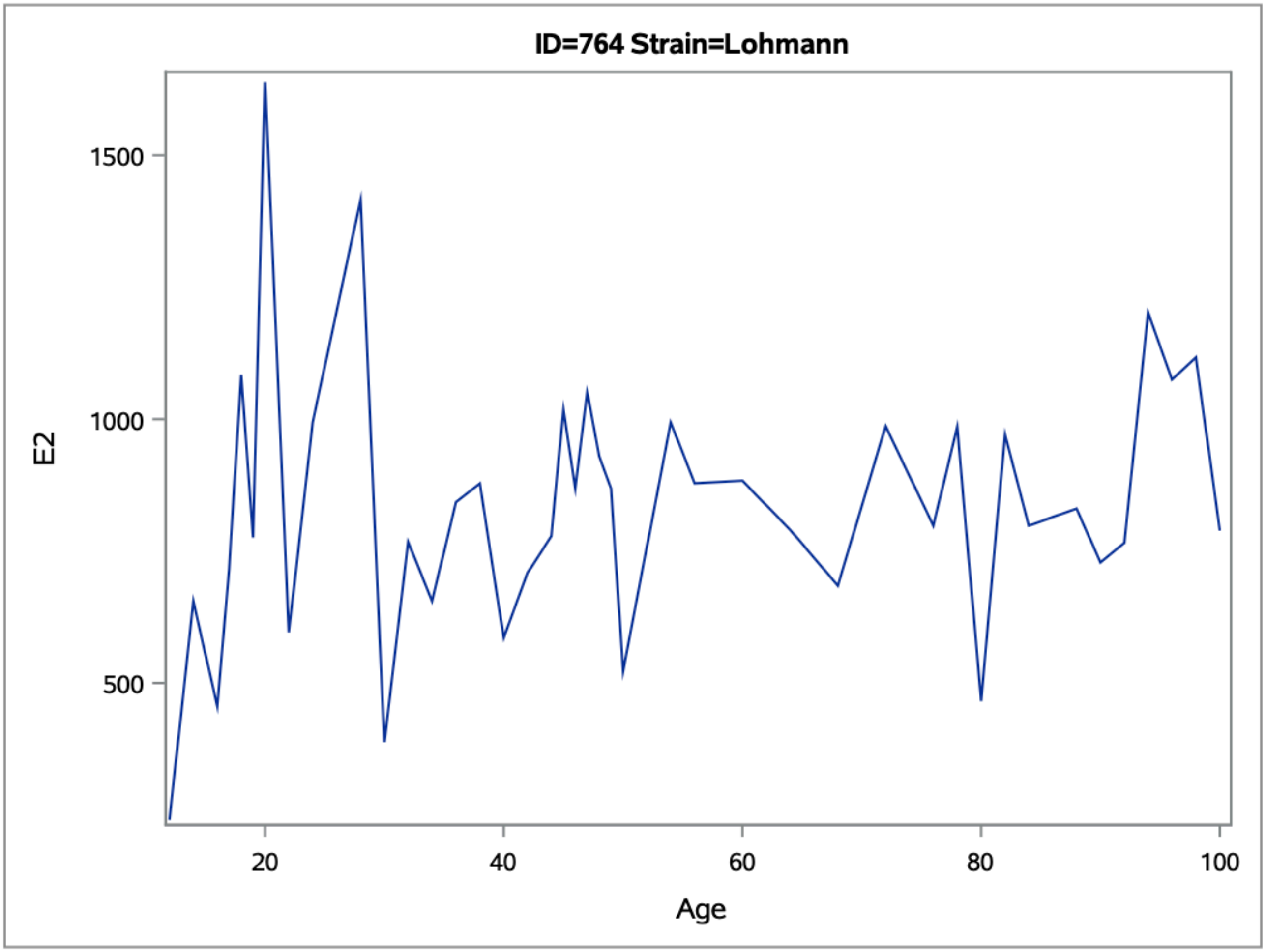
ID=764 Strain=Lohmann
1500
1000
500
E2
20
40
60
80
100
Age

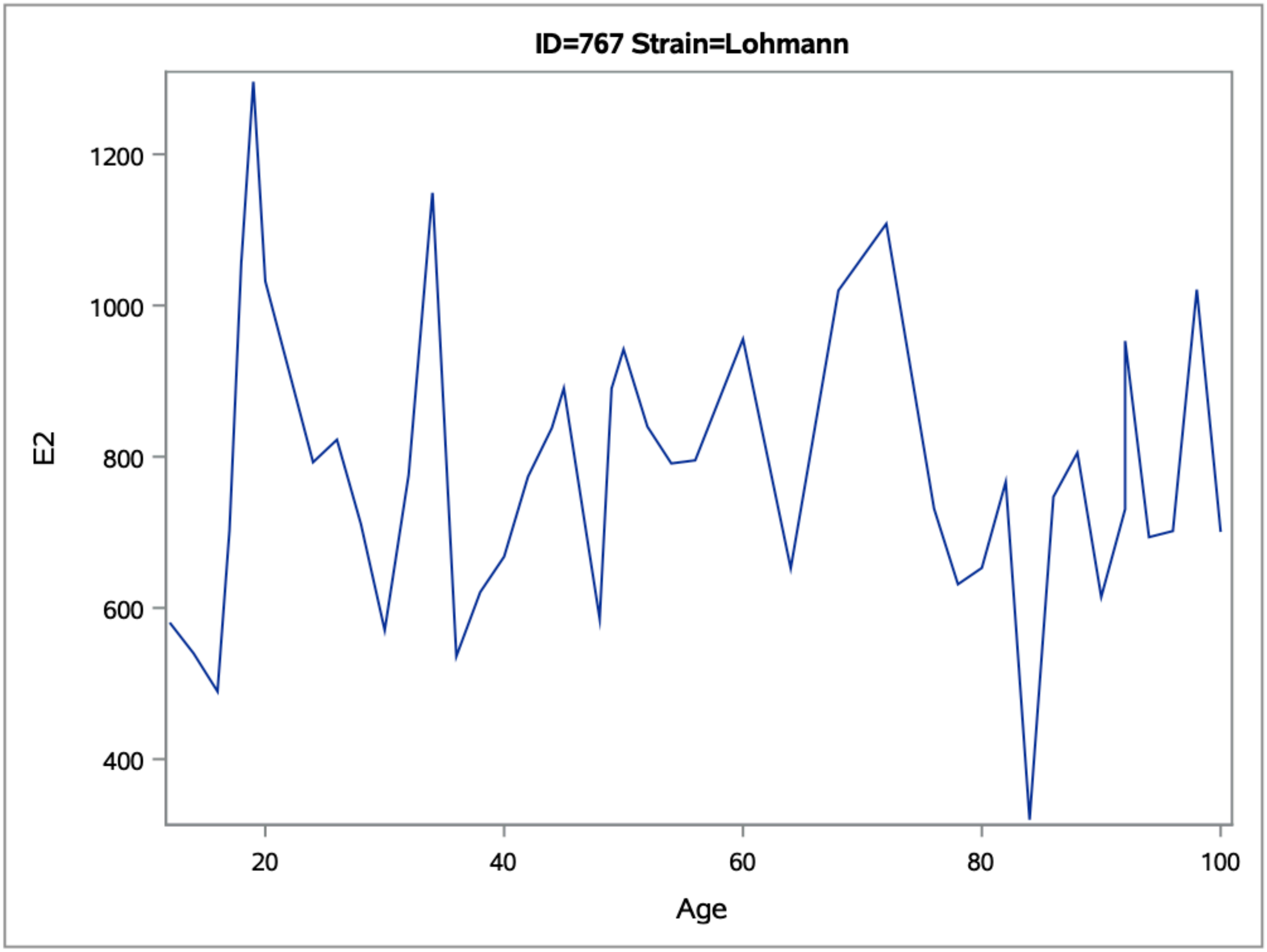

ID=767 Strain=Lohmann
E2
1200
1000
800
600
400
20
40
60
80
100
Age

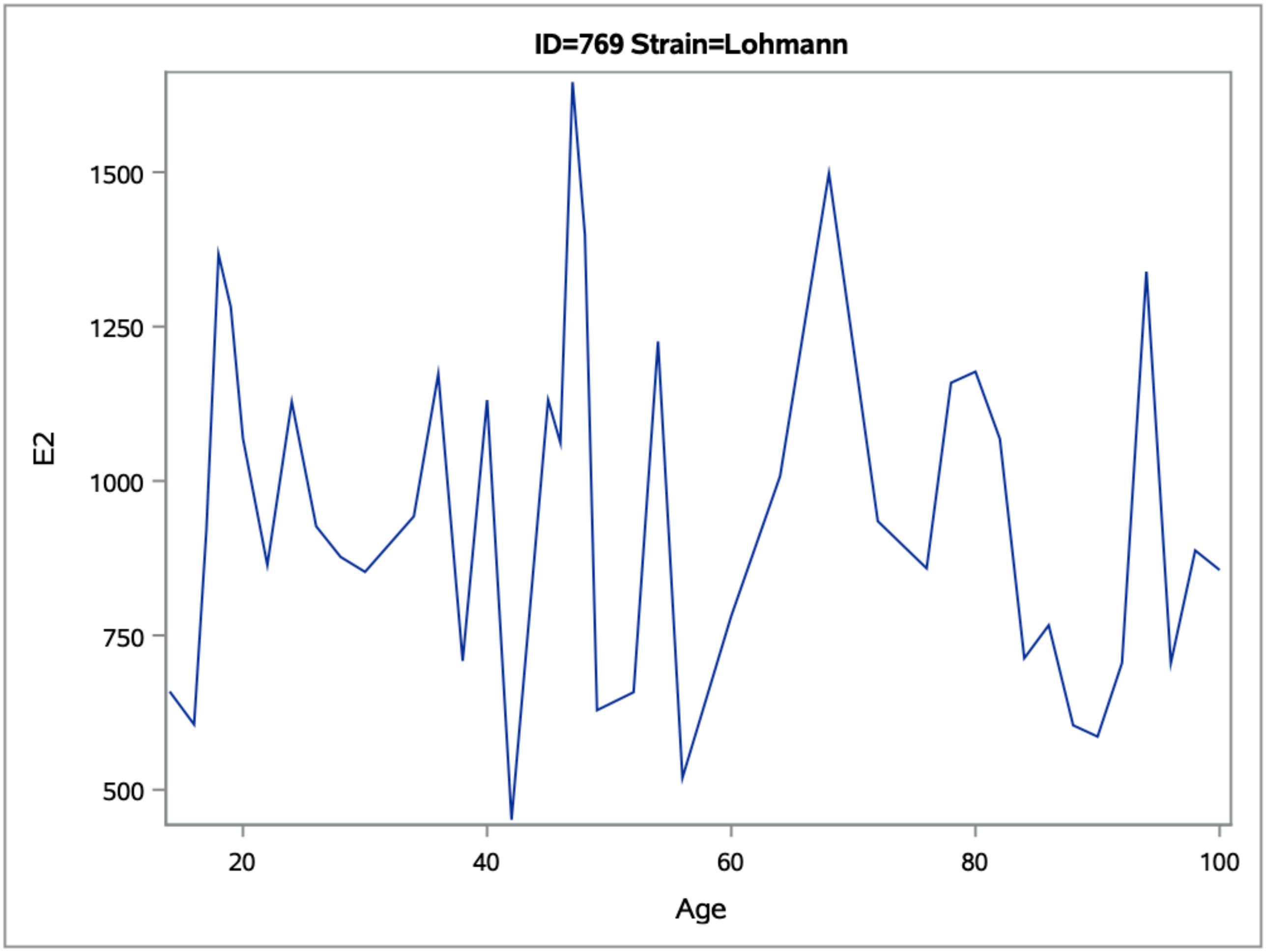
ID=769 Strain=Lohmann
E2
1500
1250
1000
750
500
20
40
60
80
100
Age

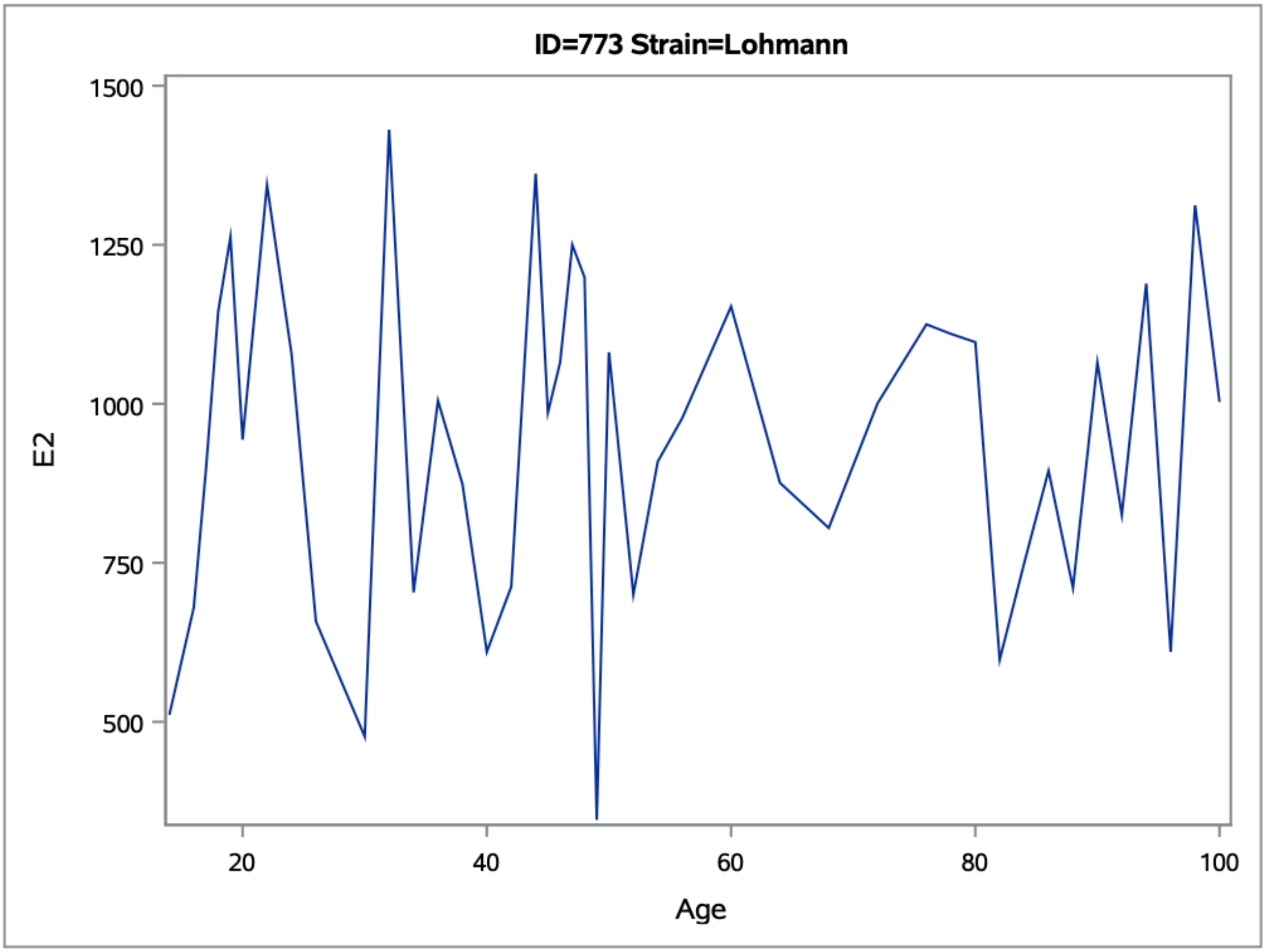

ID=773 Strain=Lohmann
1500
1250
1000
750
500
E2
20
40
60
80
100
Age

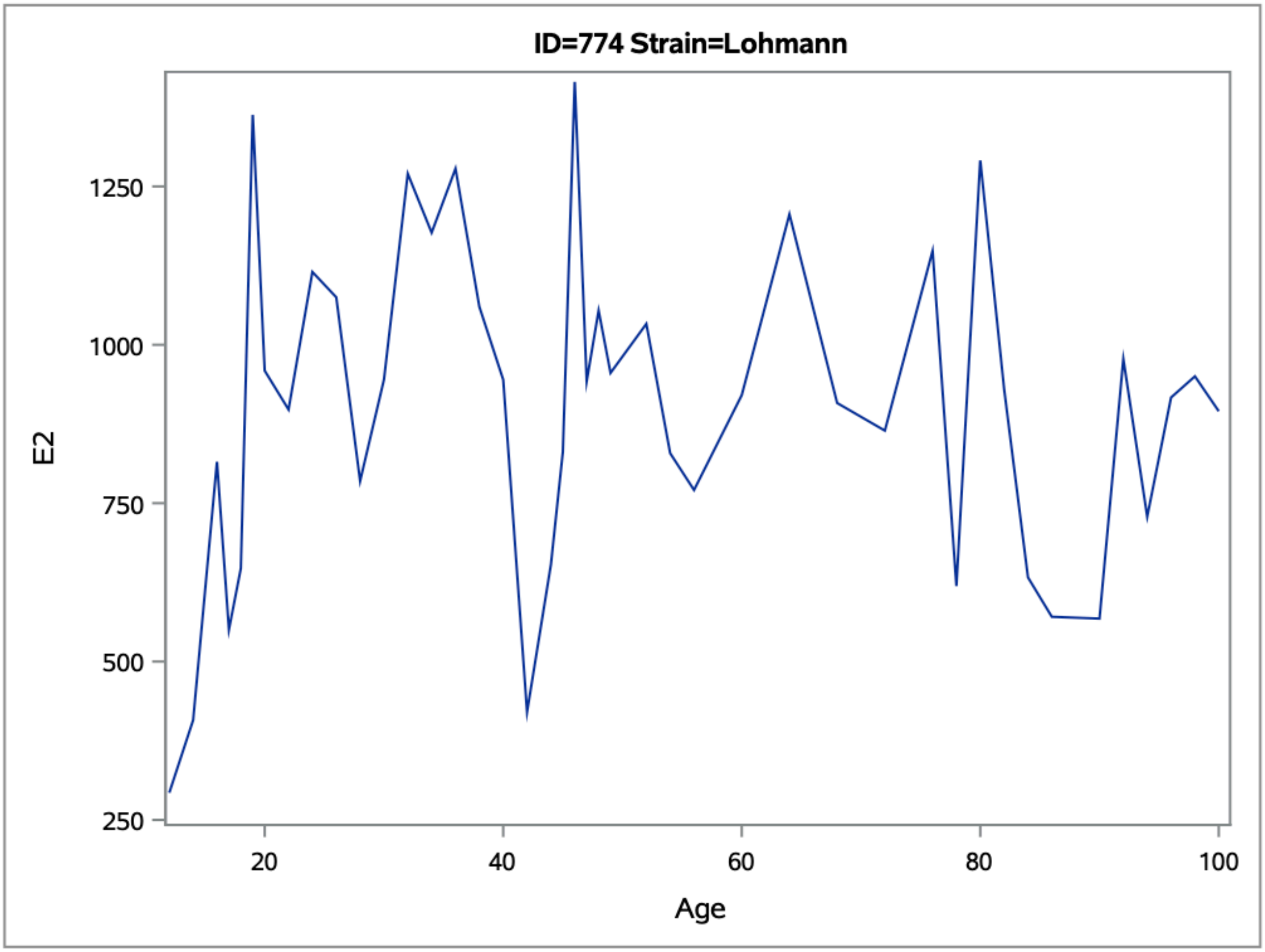
ID=774 Strain=Lohmann
E2
1250
1000
750
500
250
20
40
60
80
100
Age

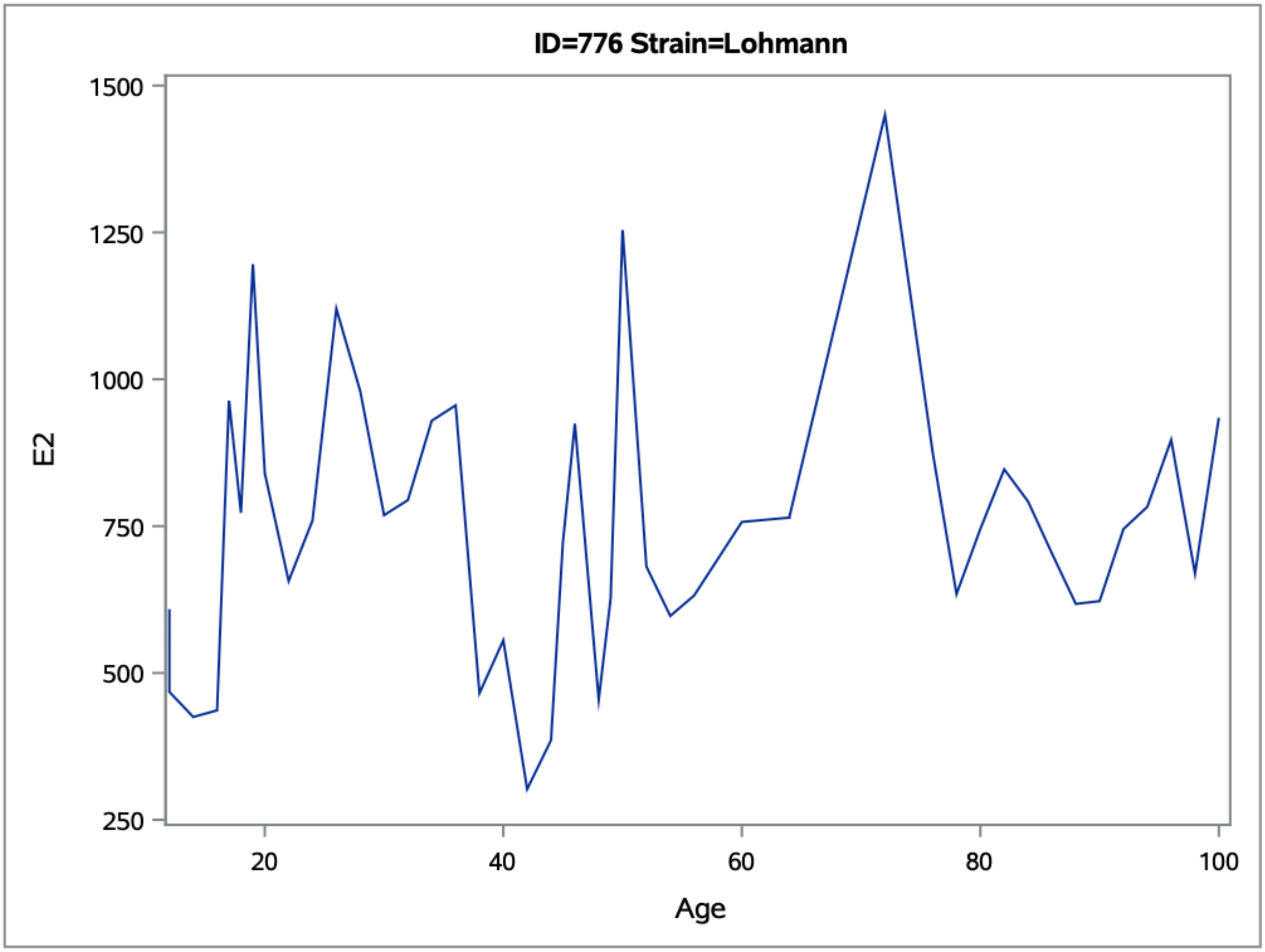

ID=776 Strain=Lohmann
1500
1250
1000
750
500
250
E2
20
40
60
80
100
Age

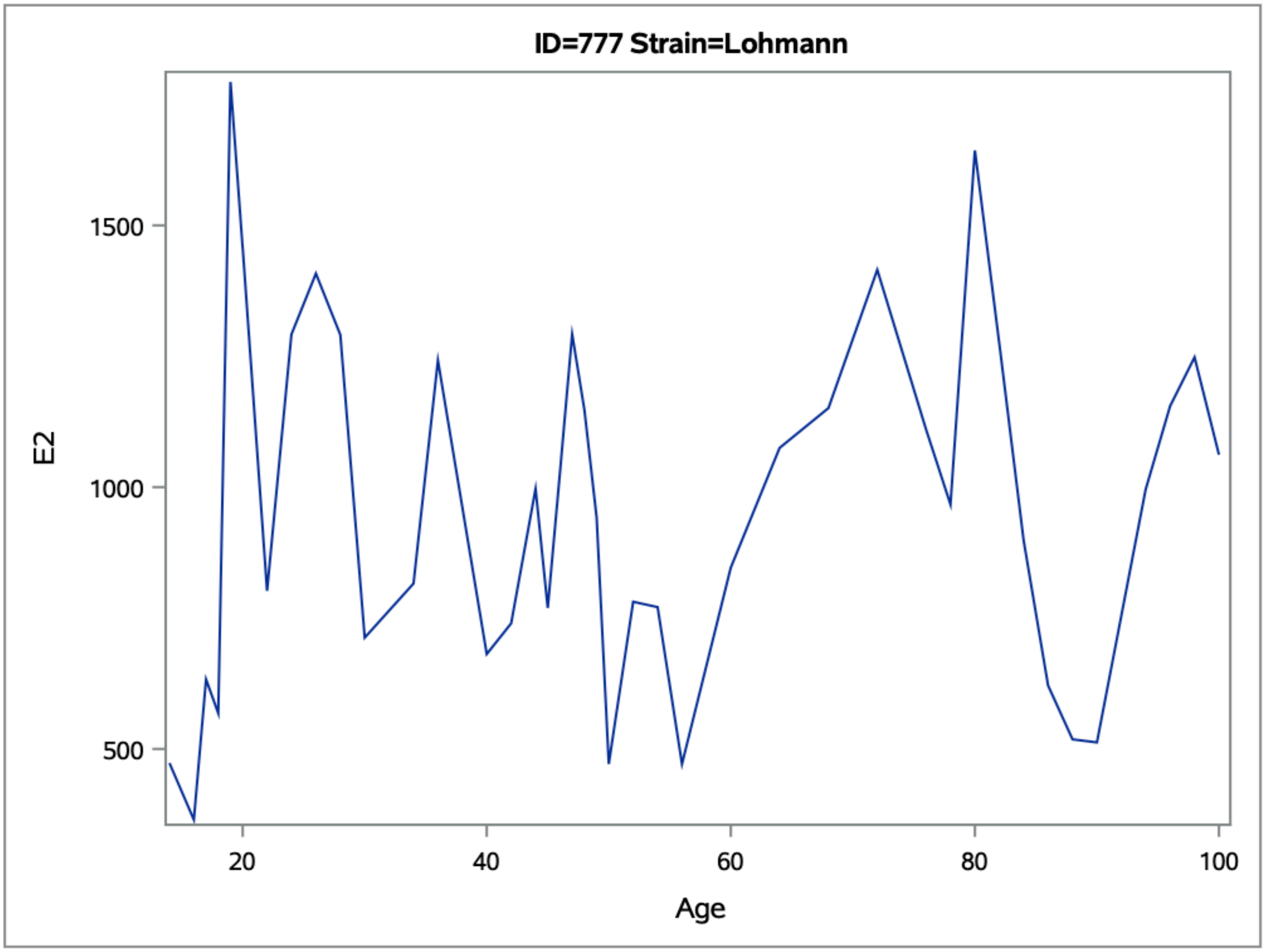
ID=777 Strain=Lohmann
E2
1500
1000
500
20
40
60
80
100
Age

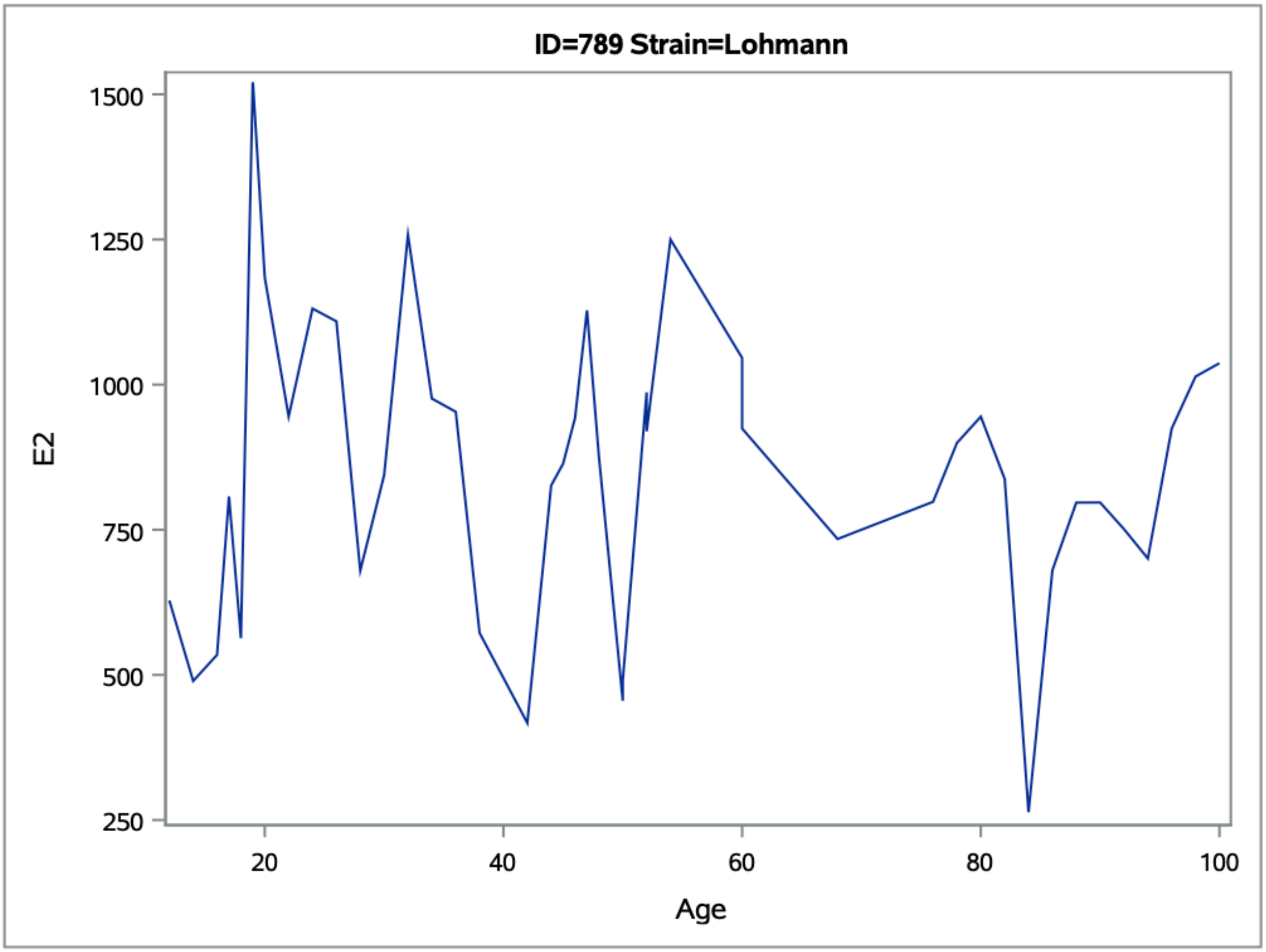
ID=789 Strain=Lohmann
1500
1250
1000
750
500
250
E2
20
40
60
80
100
Age

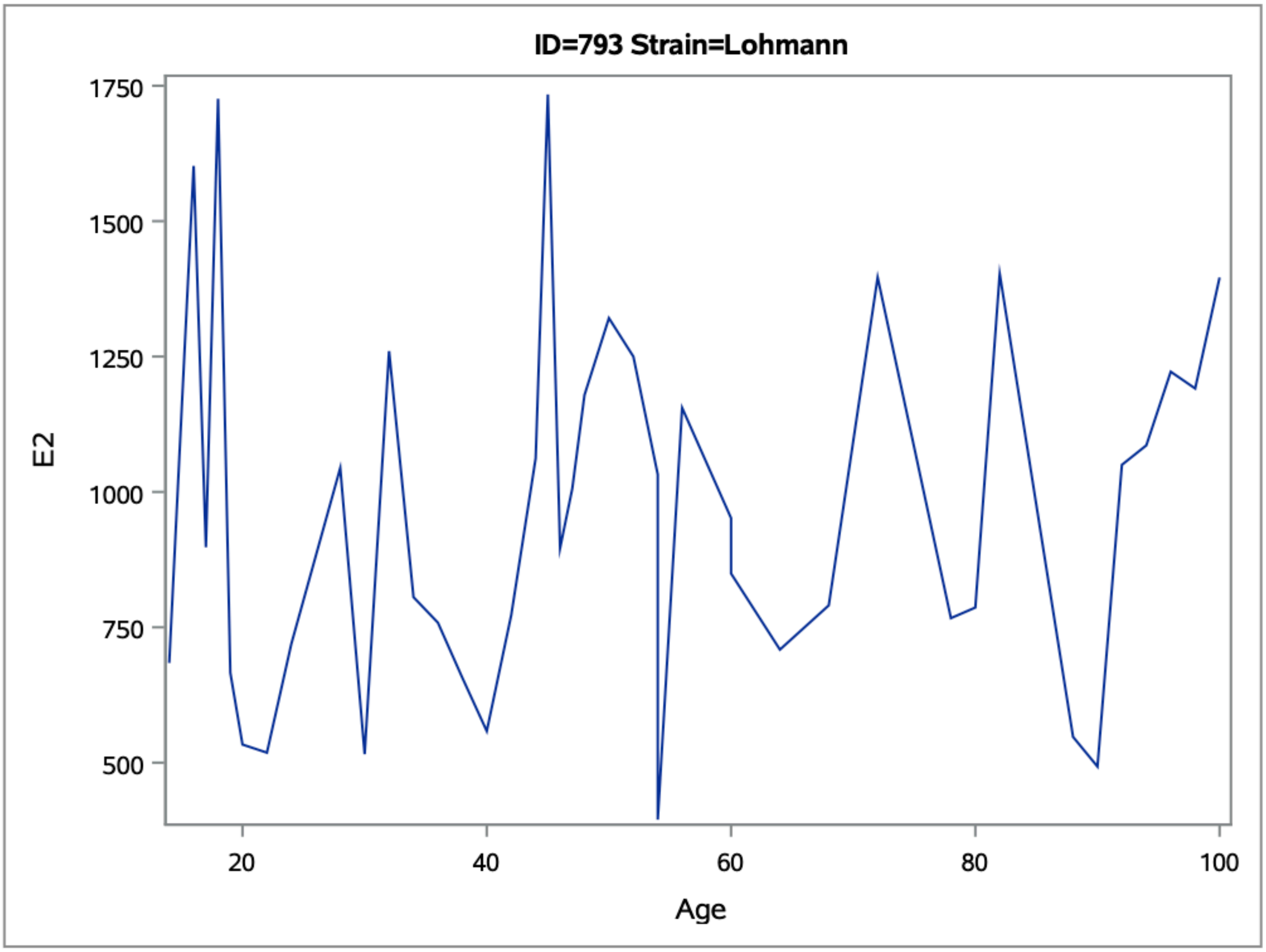
ID=793 Strain=Lohmann
1750
1500
1250
1000
750
500
E2
20
40
60
80
100
Age

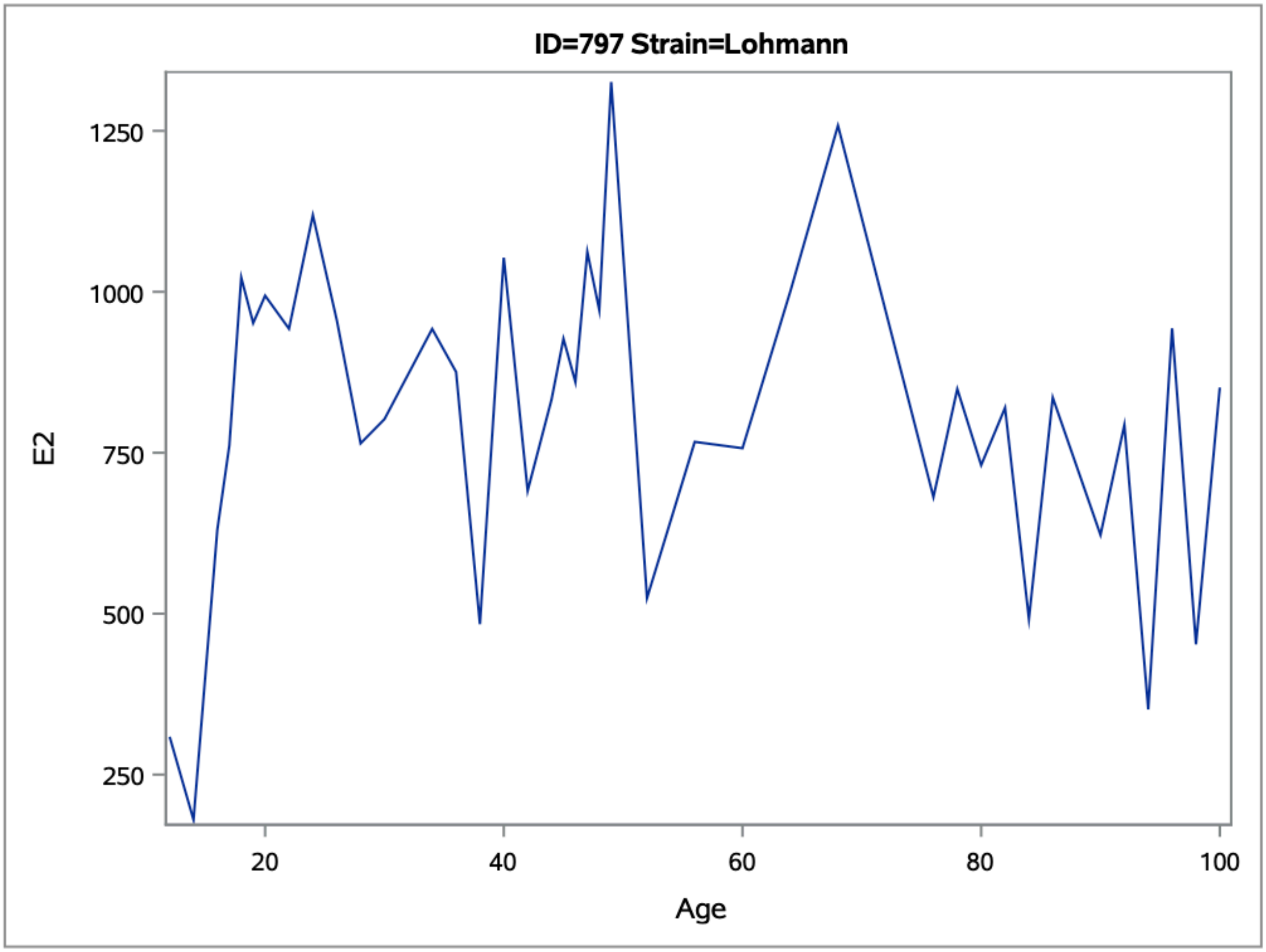
ID=797 Strain=Lohmann
E2
1250
1000
750
500
250
20
40
60
80
100
Age

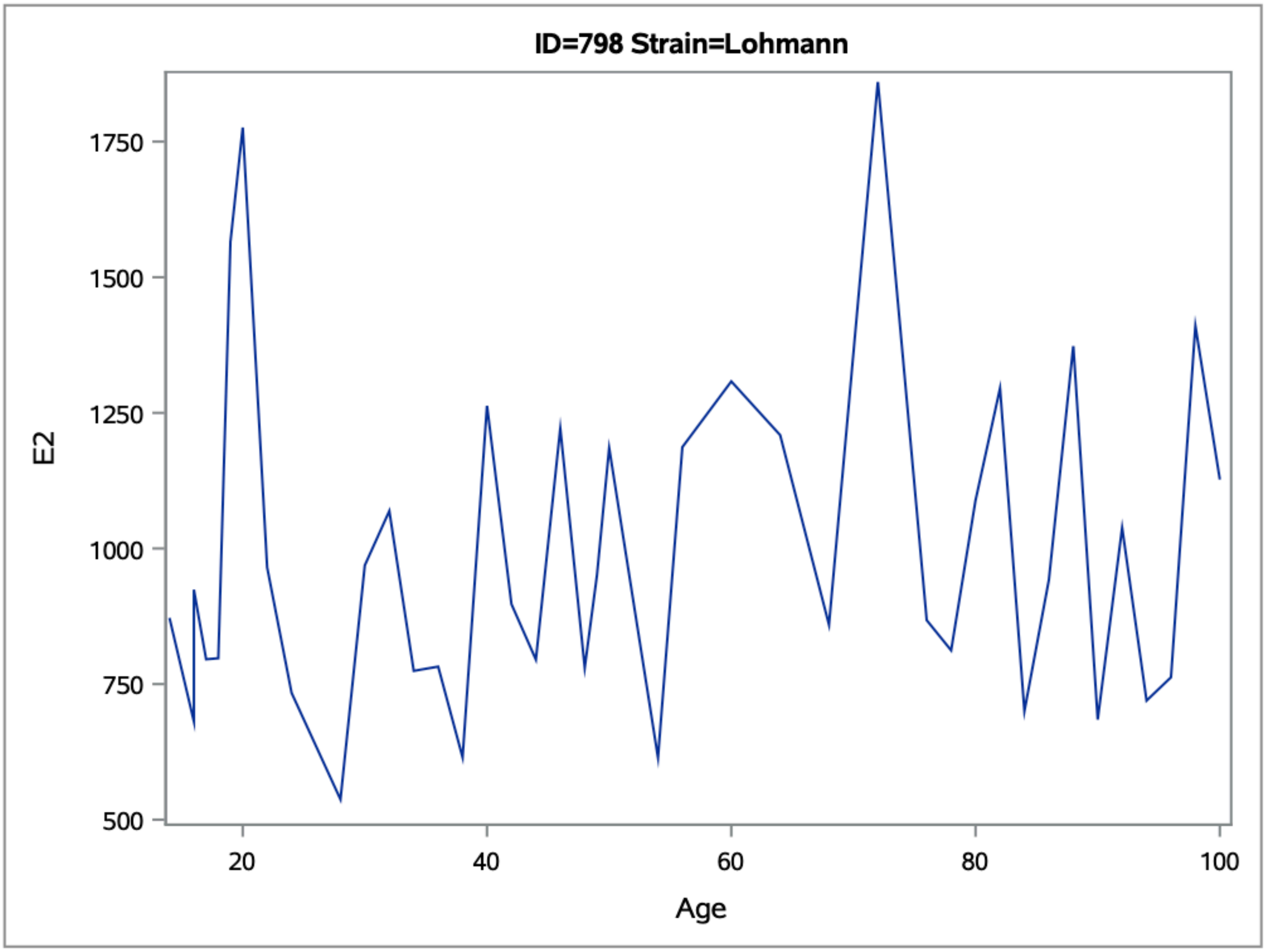
ID=798 Strain=Lohmann
E2
1750
1500
1250
1000
750
500
20
40
60
80
100
Age

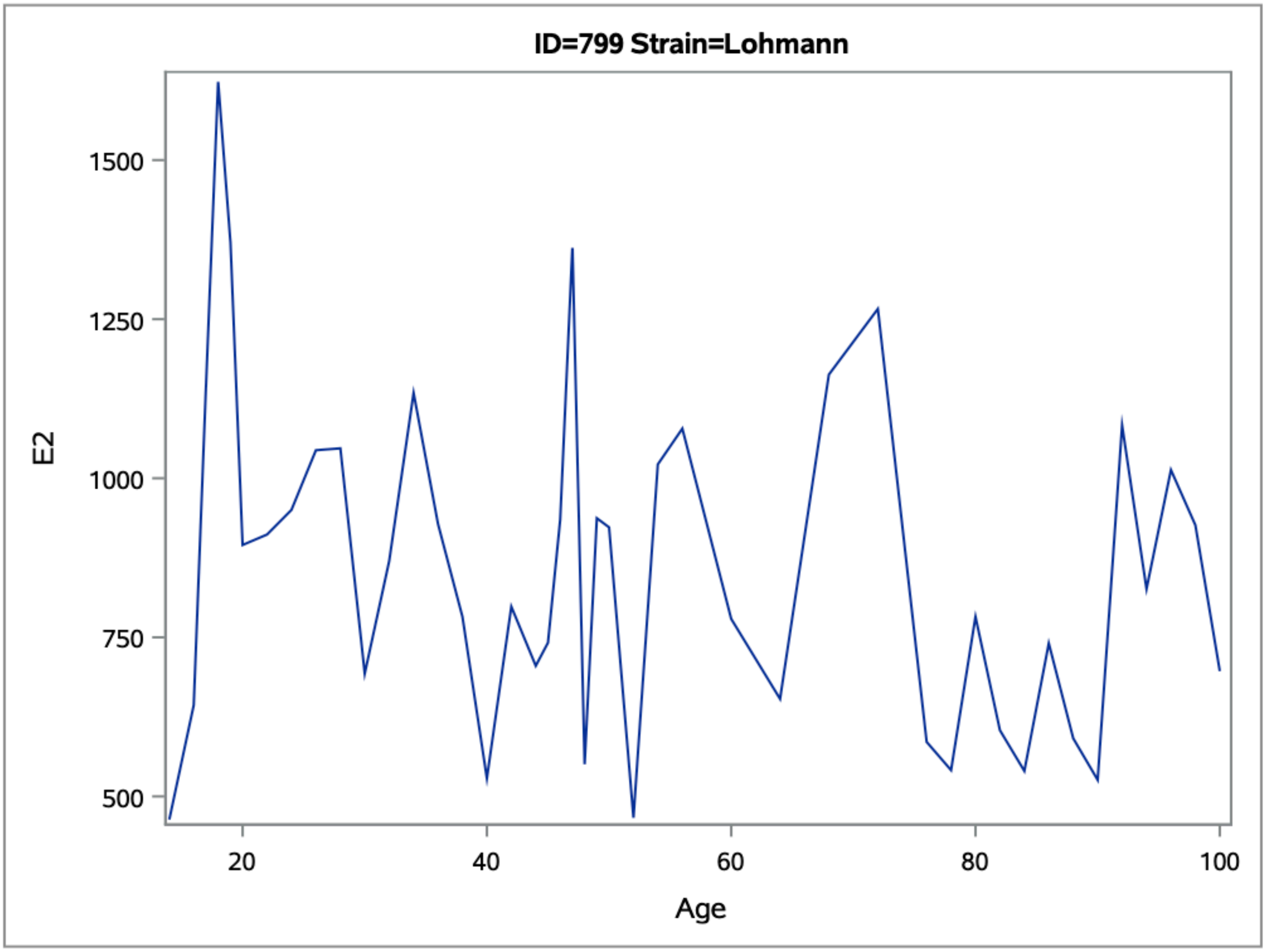
ID=799 Strain=Lohmann
E2
1500
1250
1000
750
500
20
40
60
80
100
Age

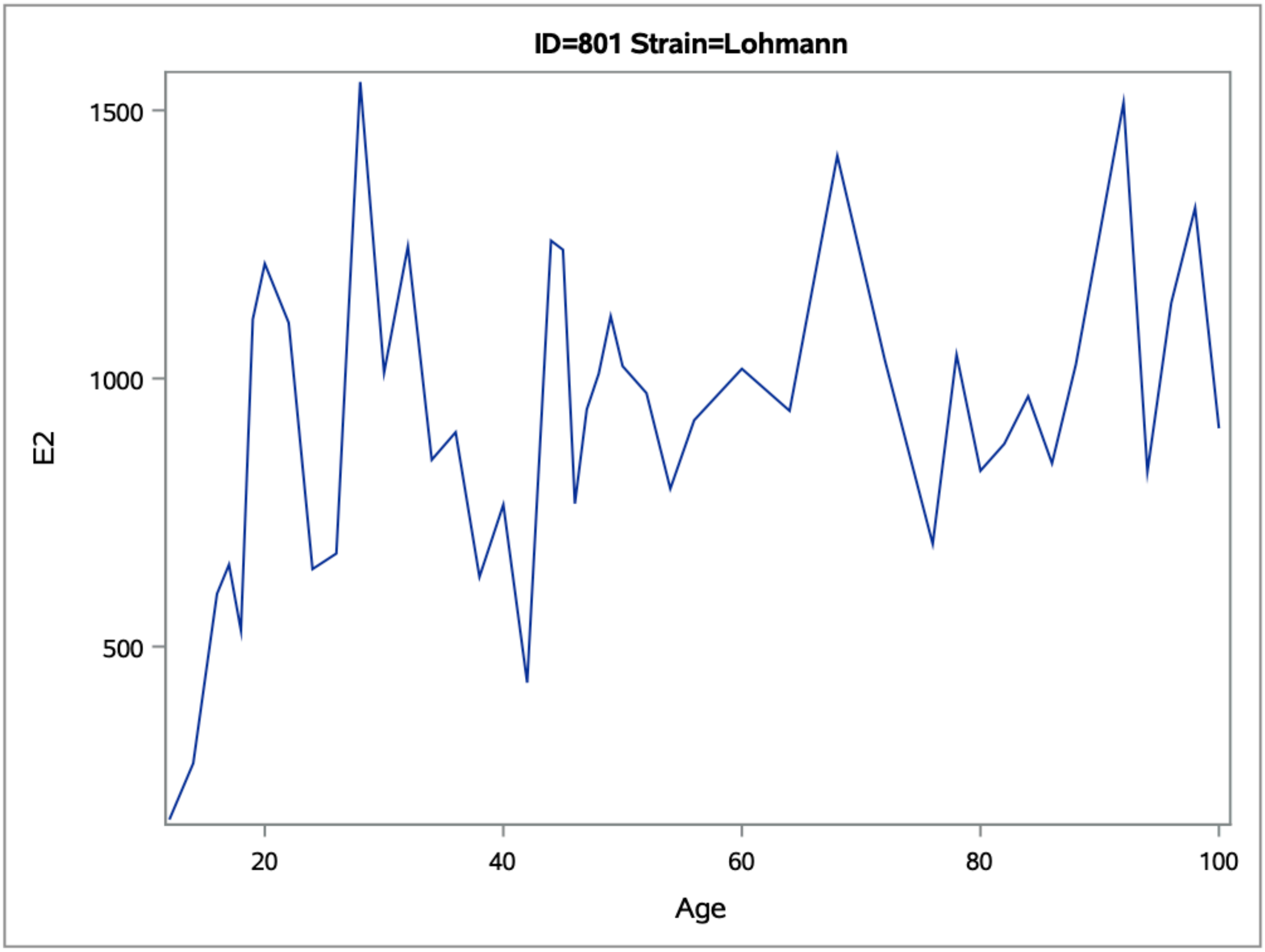
ID=801 Strain=Lohmann
1500
1000
500
E2
20
40
60
80
100
Age

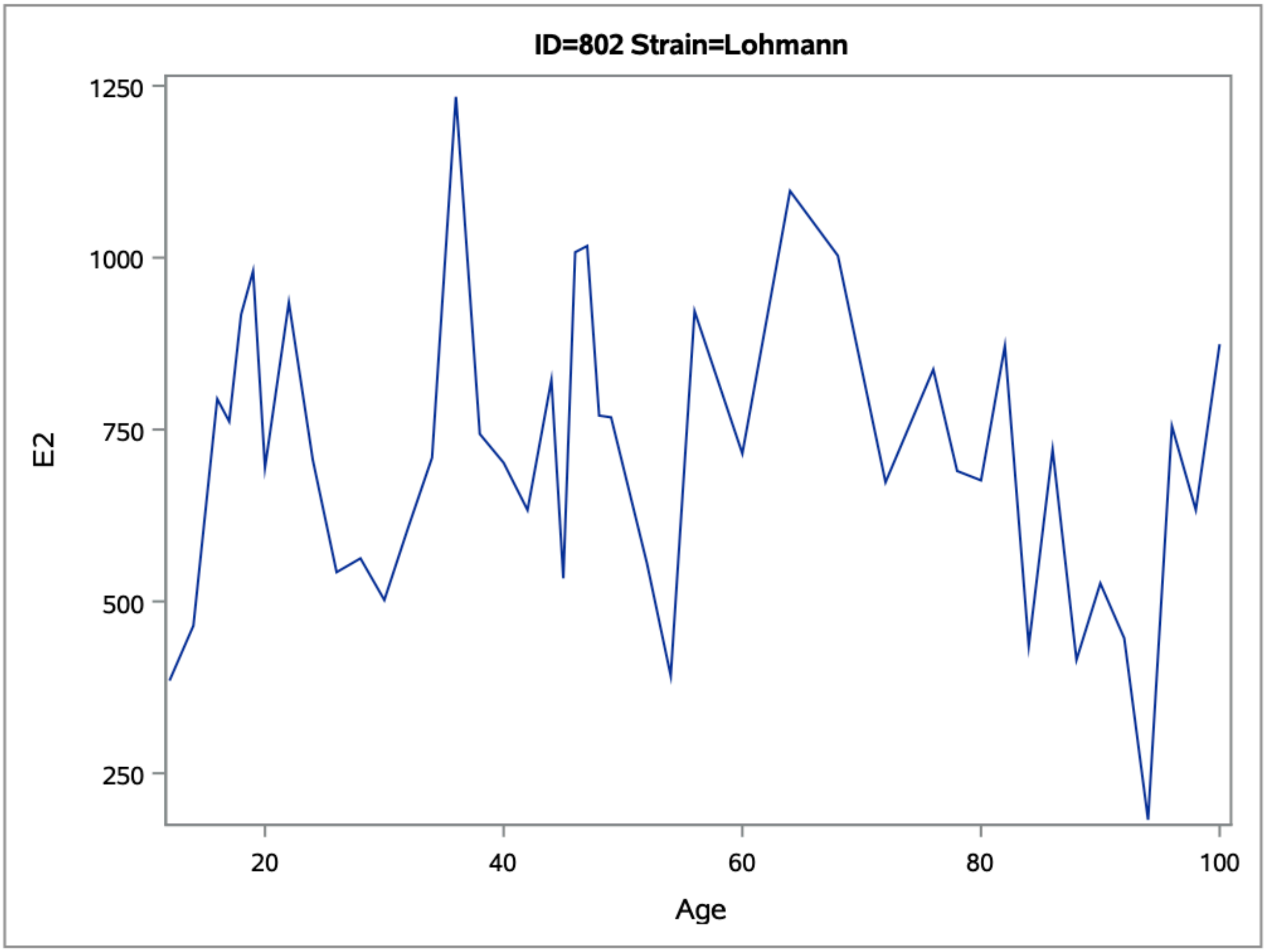
ID=802 Strain=Lohmann
1250
1000
750
500
250
E2
20
40
60
80
100
Age

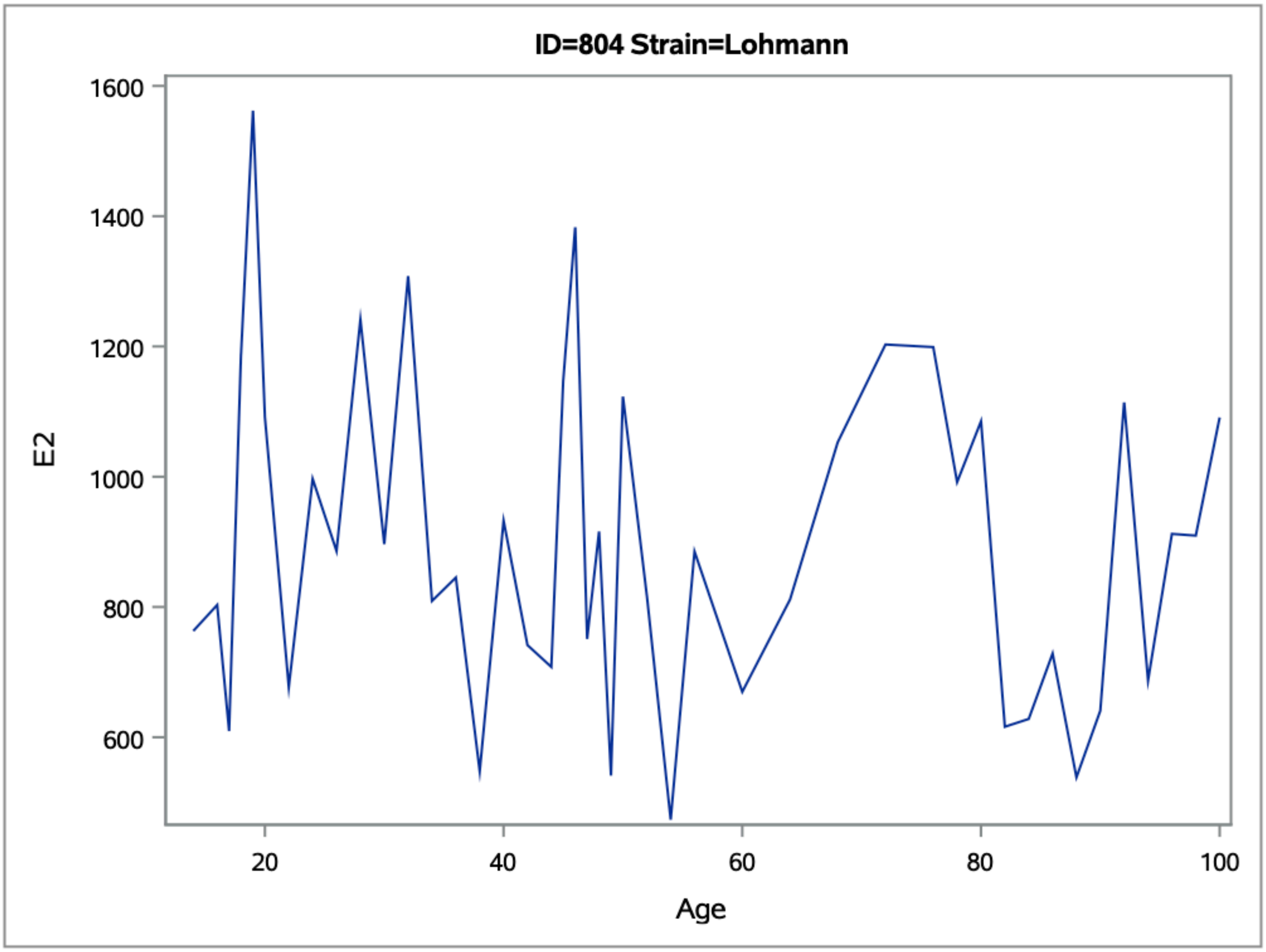
ID=804 Strain=Lohmann
E2
1600
1400
1200
1000
800
600
20
40
60
80
100
Age

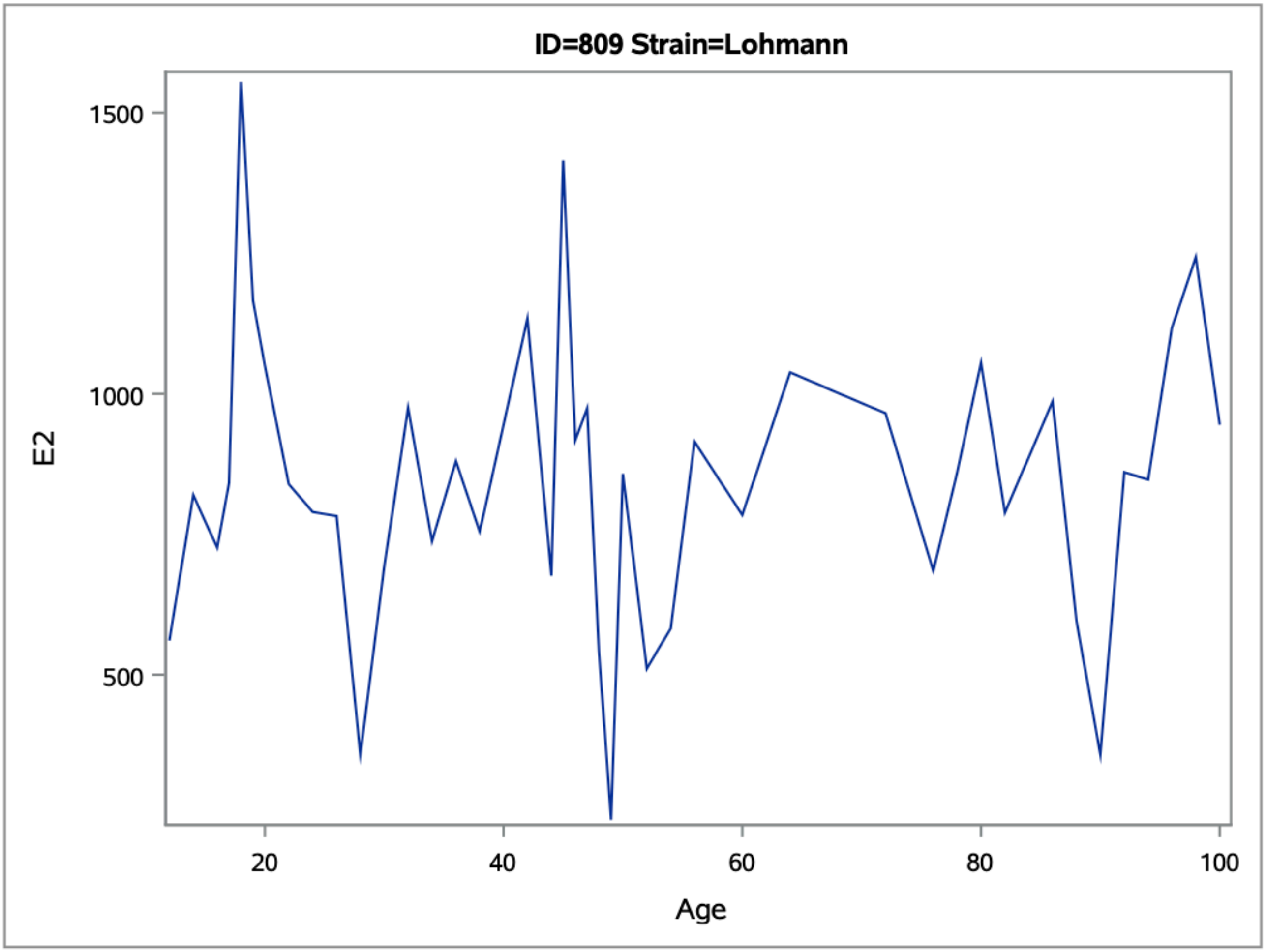
ID=809 Strain=Lohmann
1500
1000
500
E2
20
40
60
80
100
Age

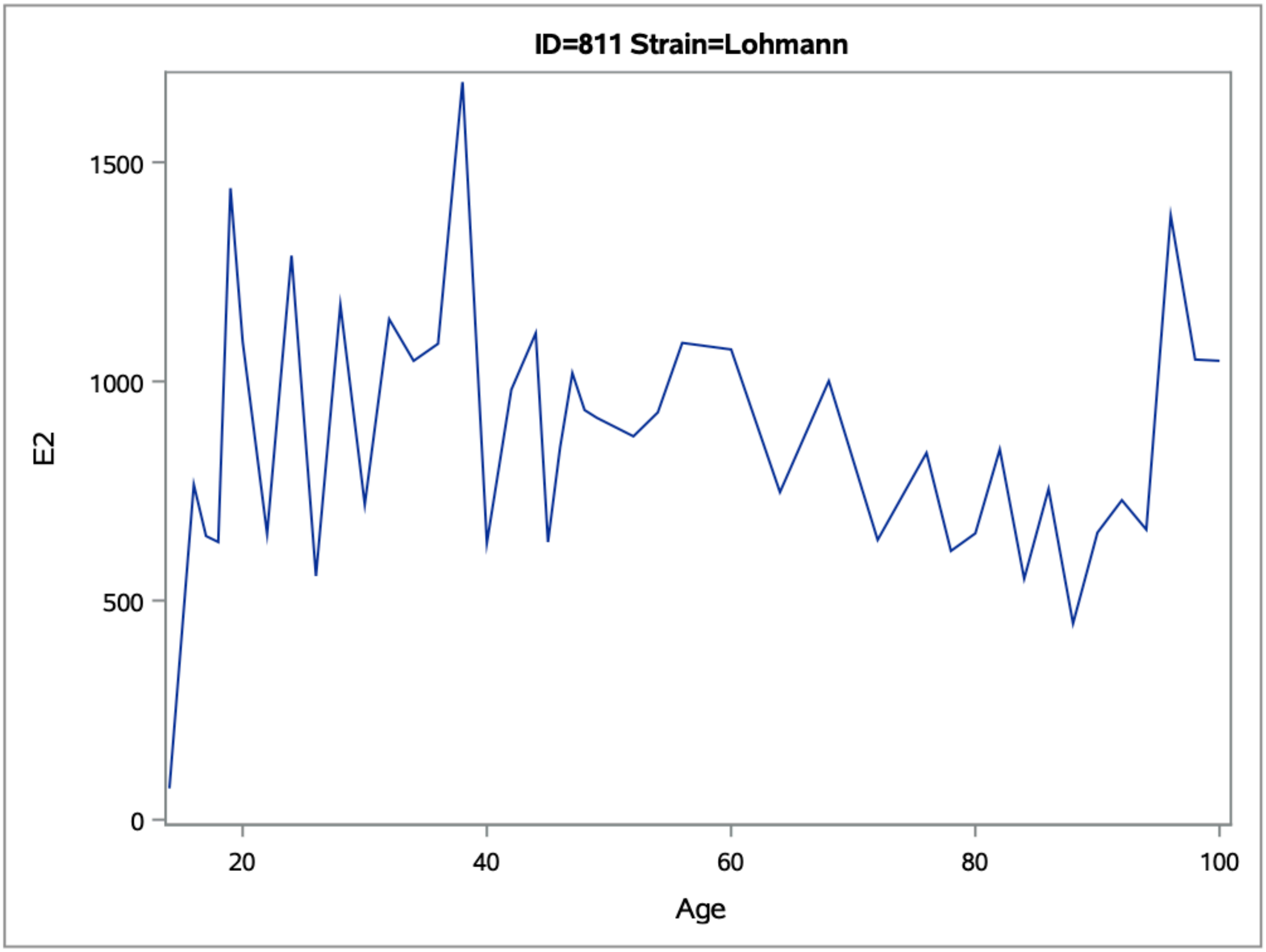
ID=811 Strain=Lohmann
1500
1000
500
0
E2
20
40
60
80
100
Age

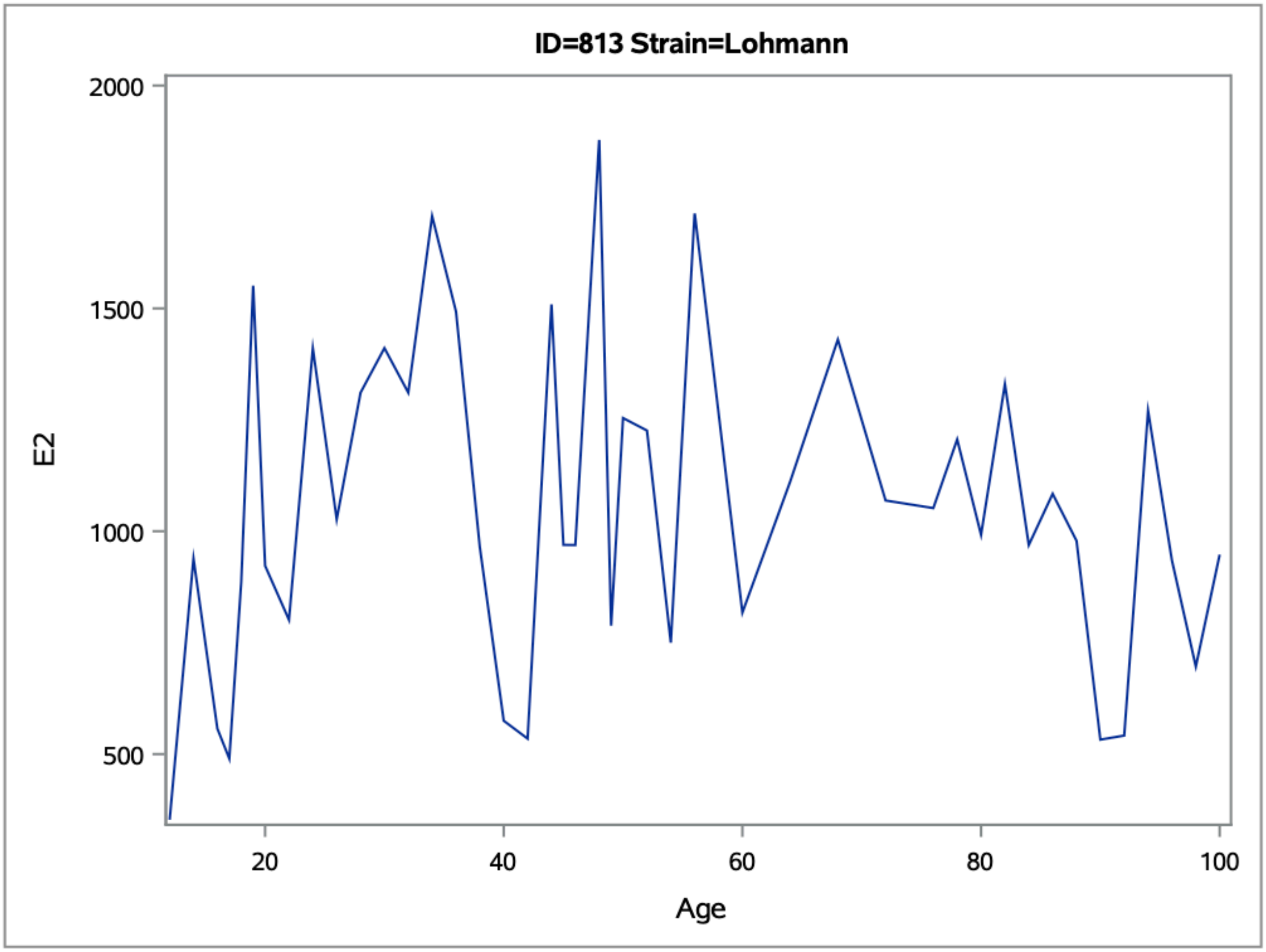
ID=813 Strain=Lohmann
2000
1500
1000
500
E2
20
40
60
80
100
Age

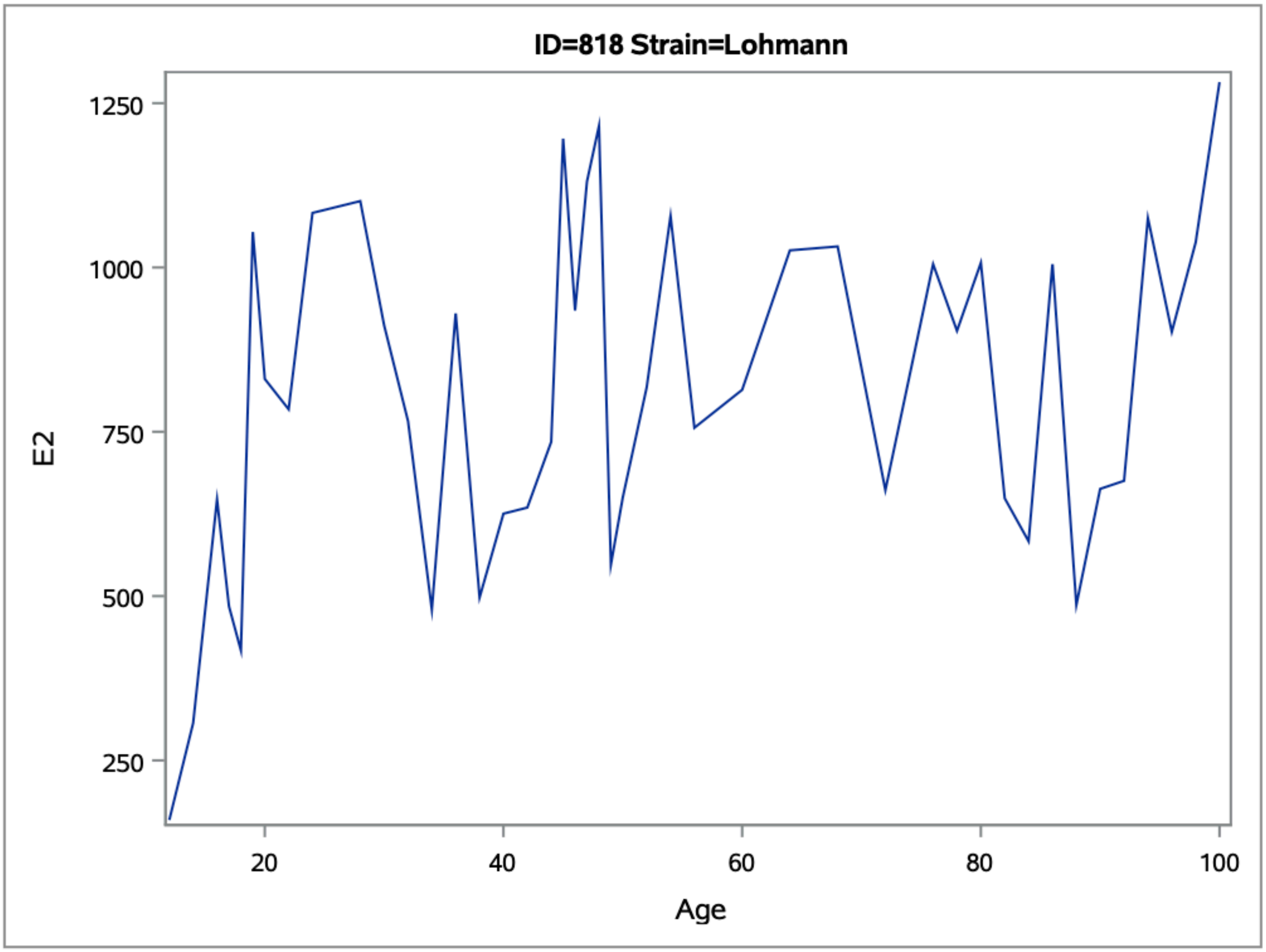
ID=818 Strain=Lohmann
E2
1250
1000
750
500
250
20
40
60
80
100
Age

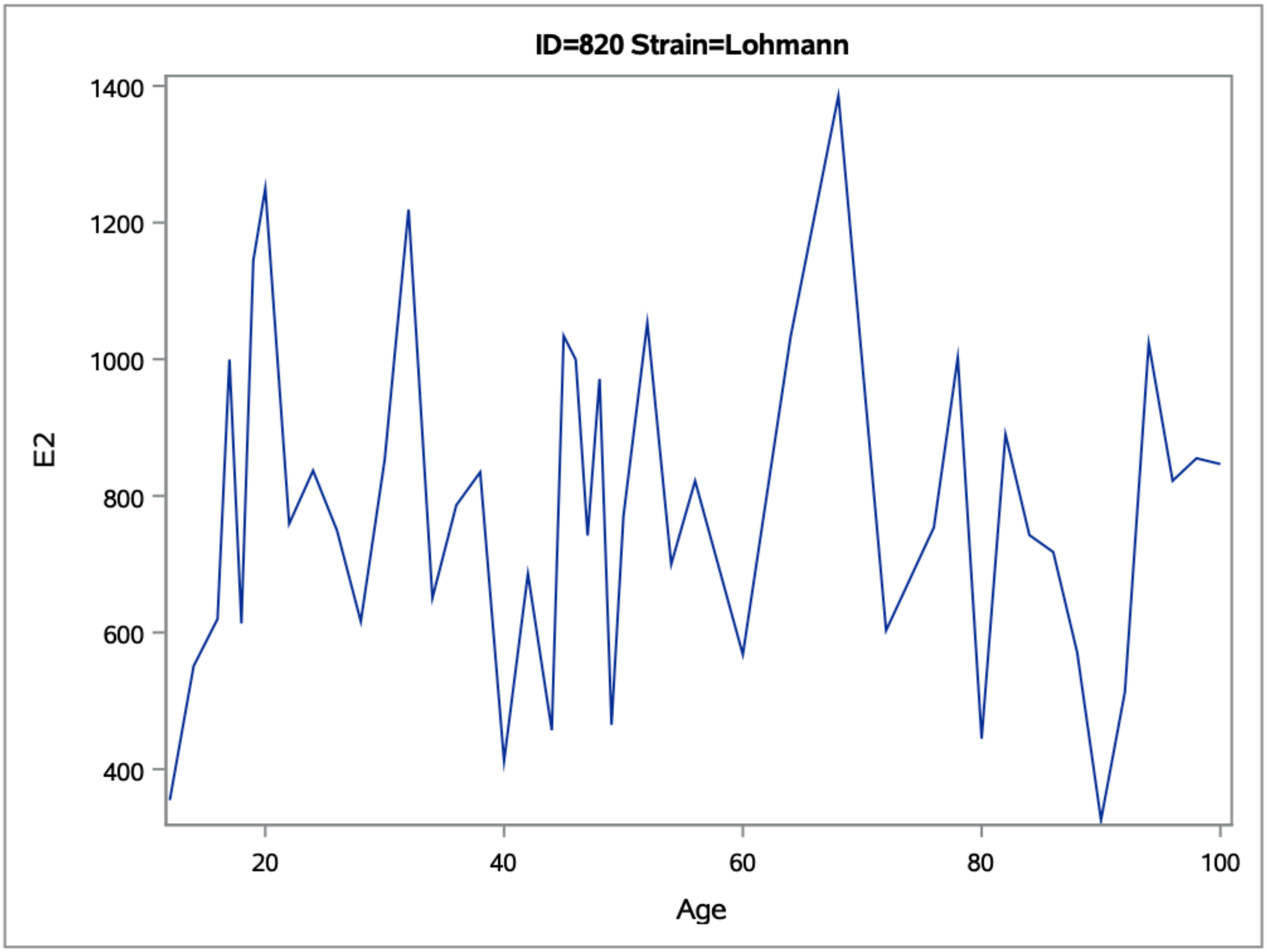

ID=820 Strain=Lohmann
1400
1200
1000
800
600
400
E2
20
40
60
80
100
Age

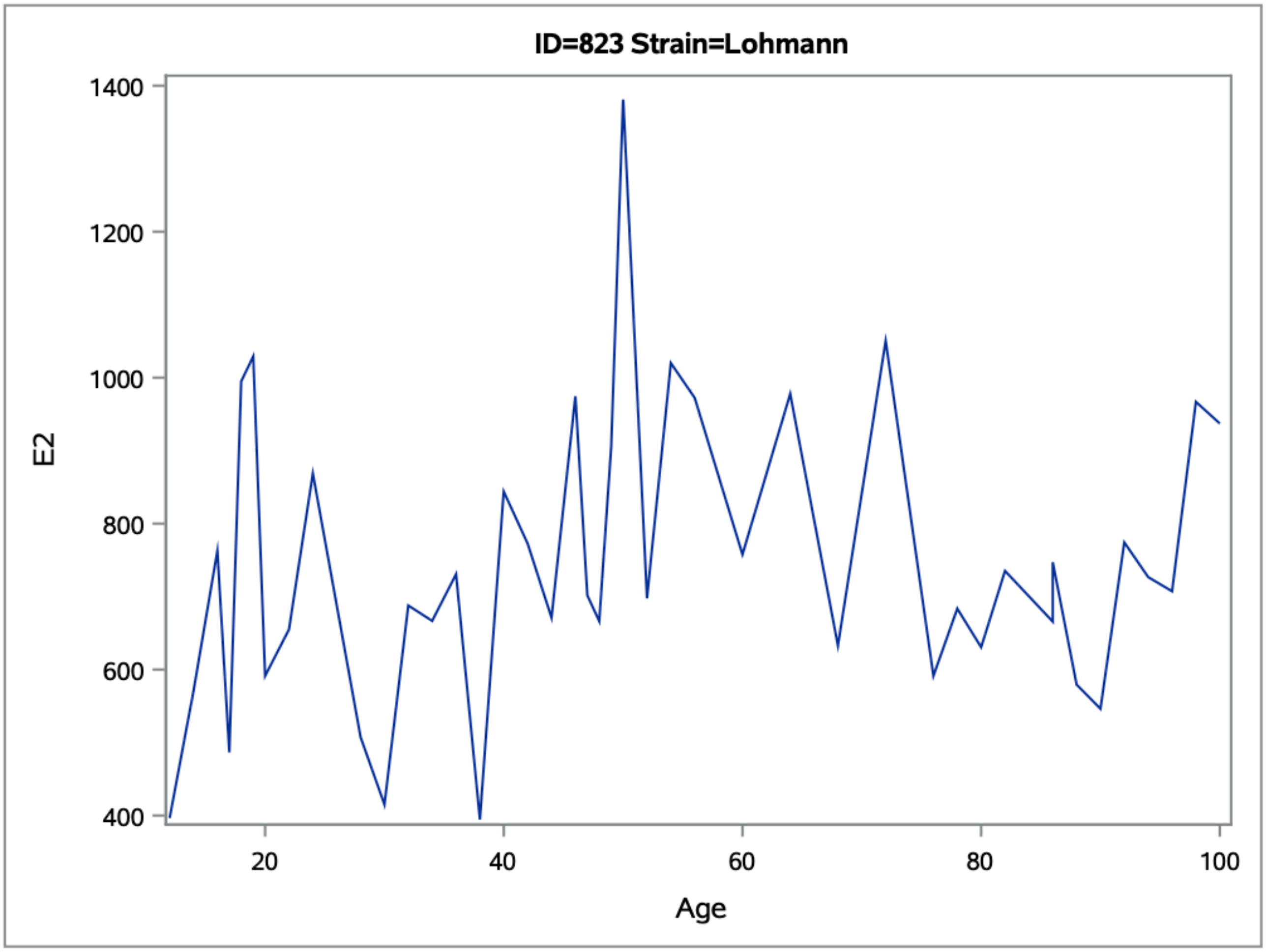

ID=823 Strain=Lohmann
1400
1200
1000
800
600
400
E2
20
40
60
80
100
Age

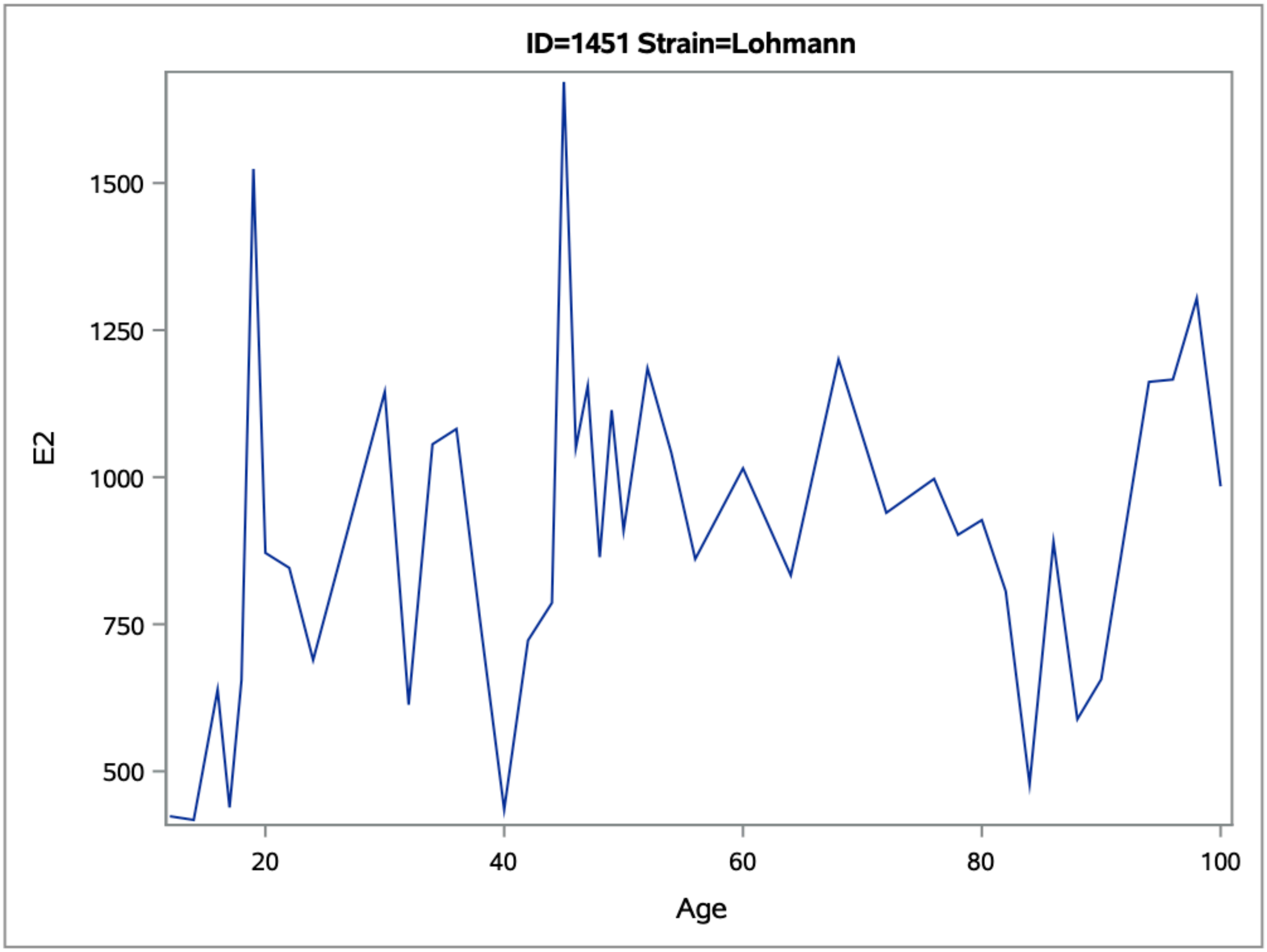
ID=1451 Strain=Lohmann
E2
1500
1250
1000
750
500
20
40
60
80
100
Age

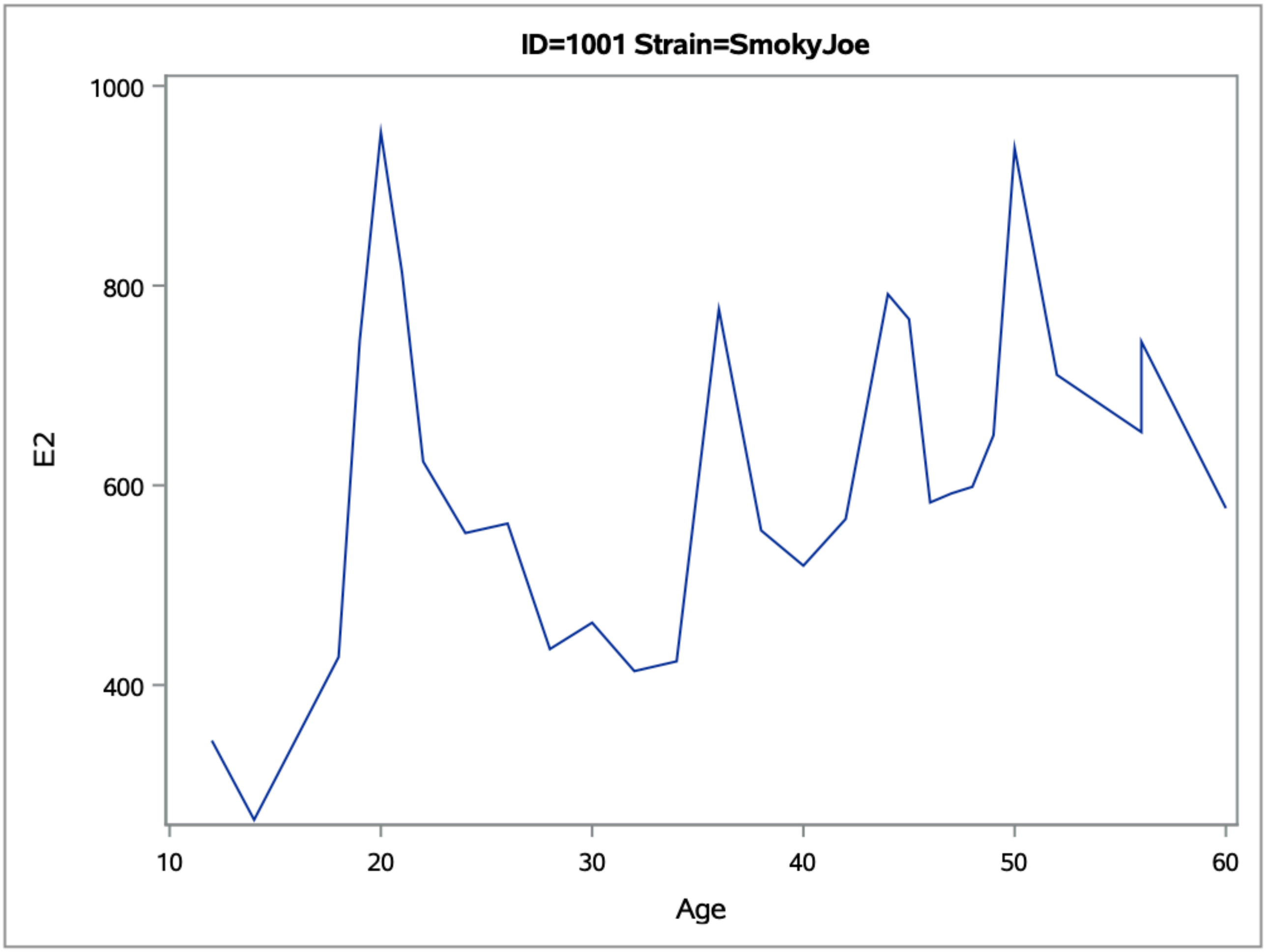
ID=1001 Strain=SmokyJoe
1000
800
600
400
E2
10
20
30
40
50
60
Age

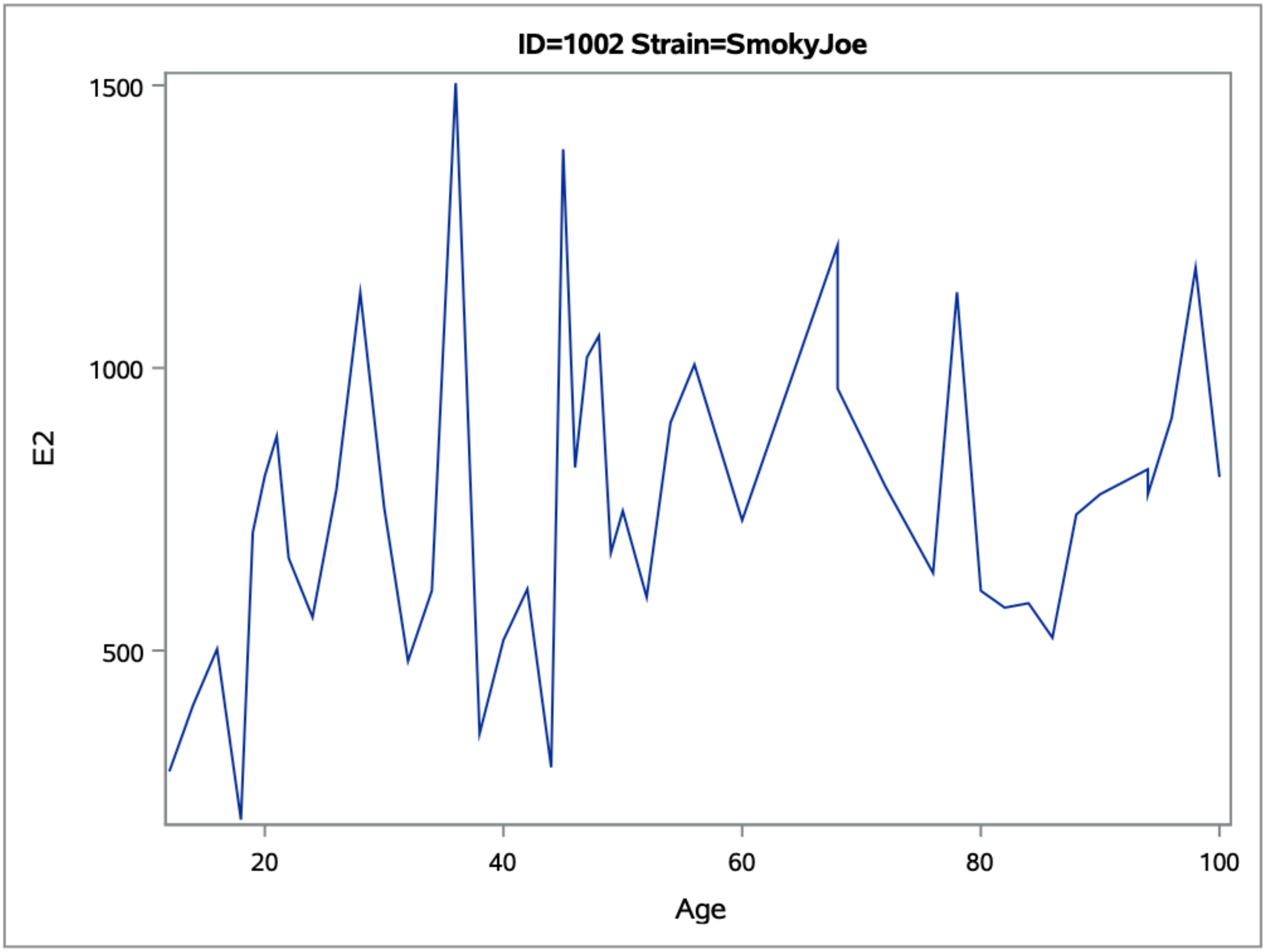
ID=1002 Strain=SmokyJoe
1500
1000
500
E2
20
40
60
80
100
Age

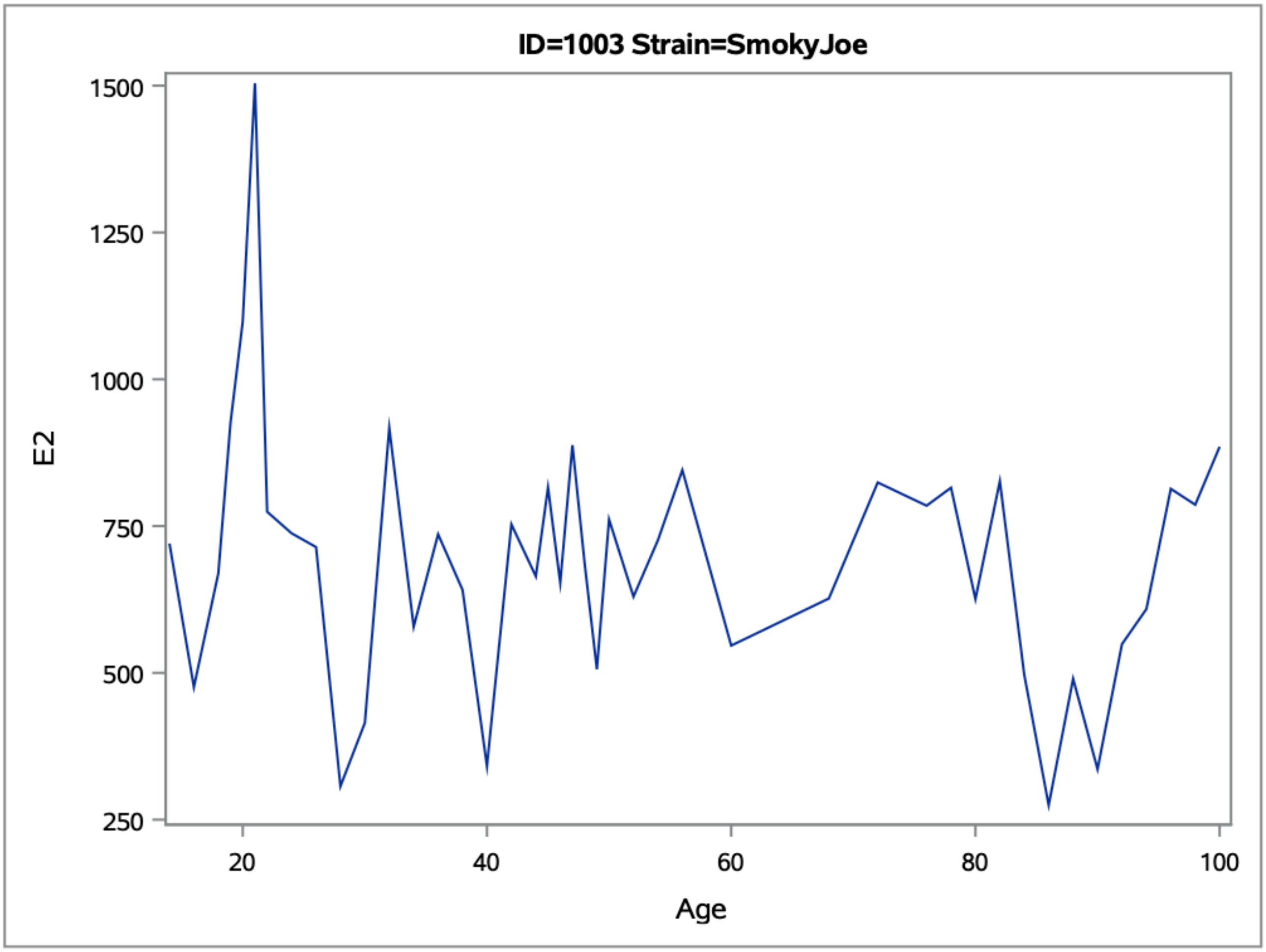
ID=1003 Strain=SmokyJoe
1500
1250
1000
750
500
250
E2
20
40
60
80
100
Age

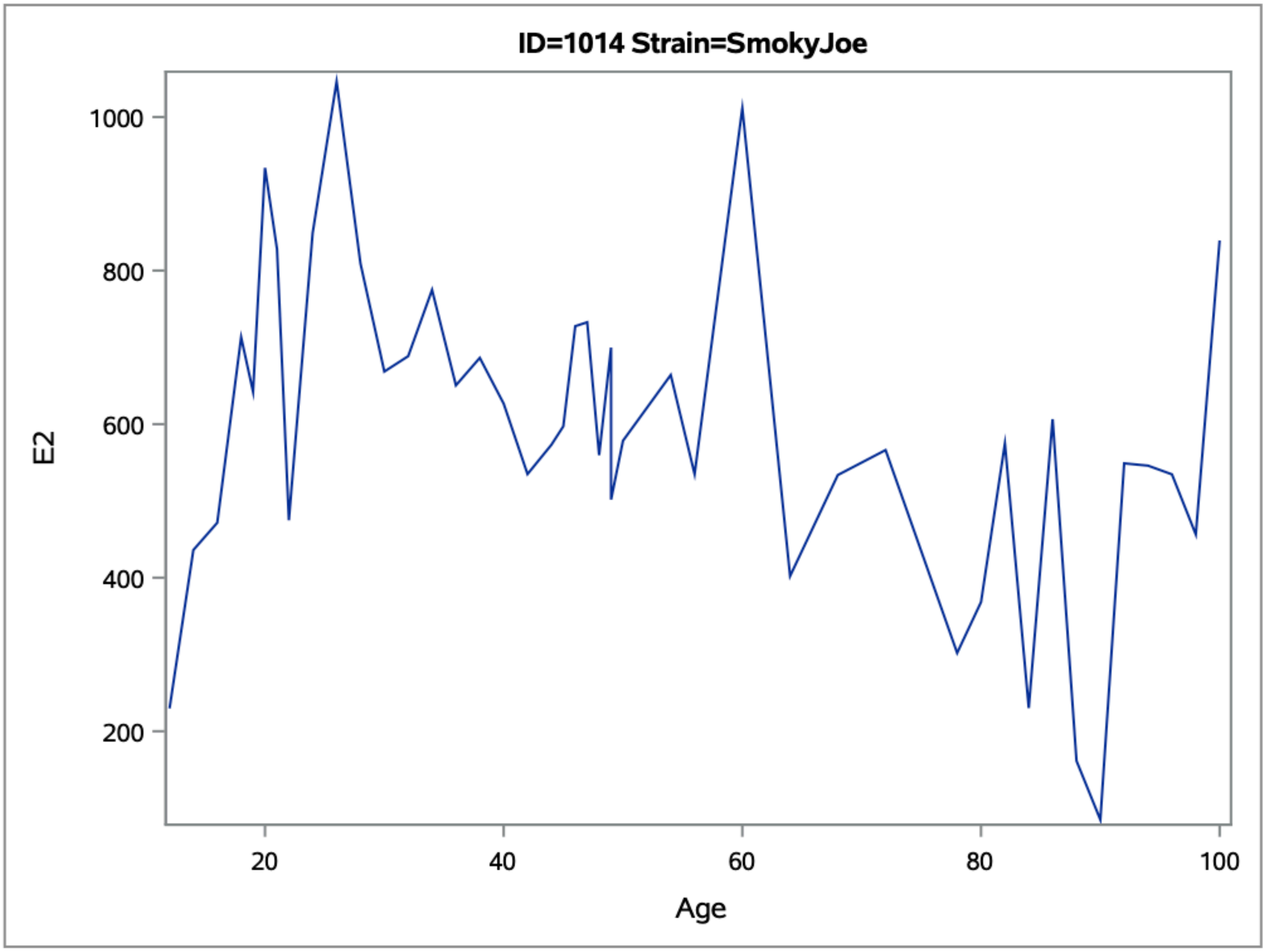
ID=1014 Strain=SmokyJoe
1000
800
600
400
200
E2
20
40
60
80
100
Age

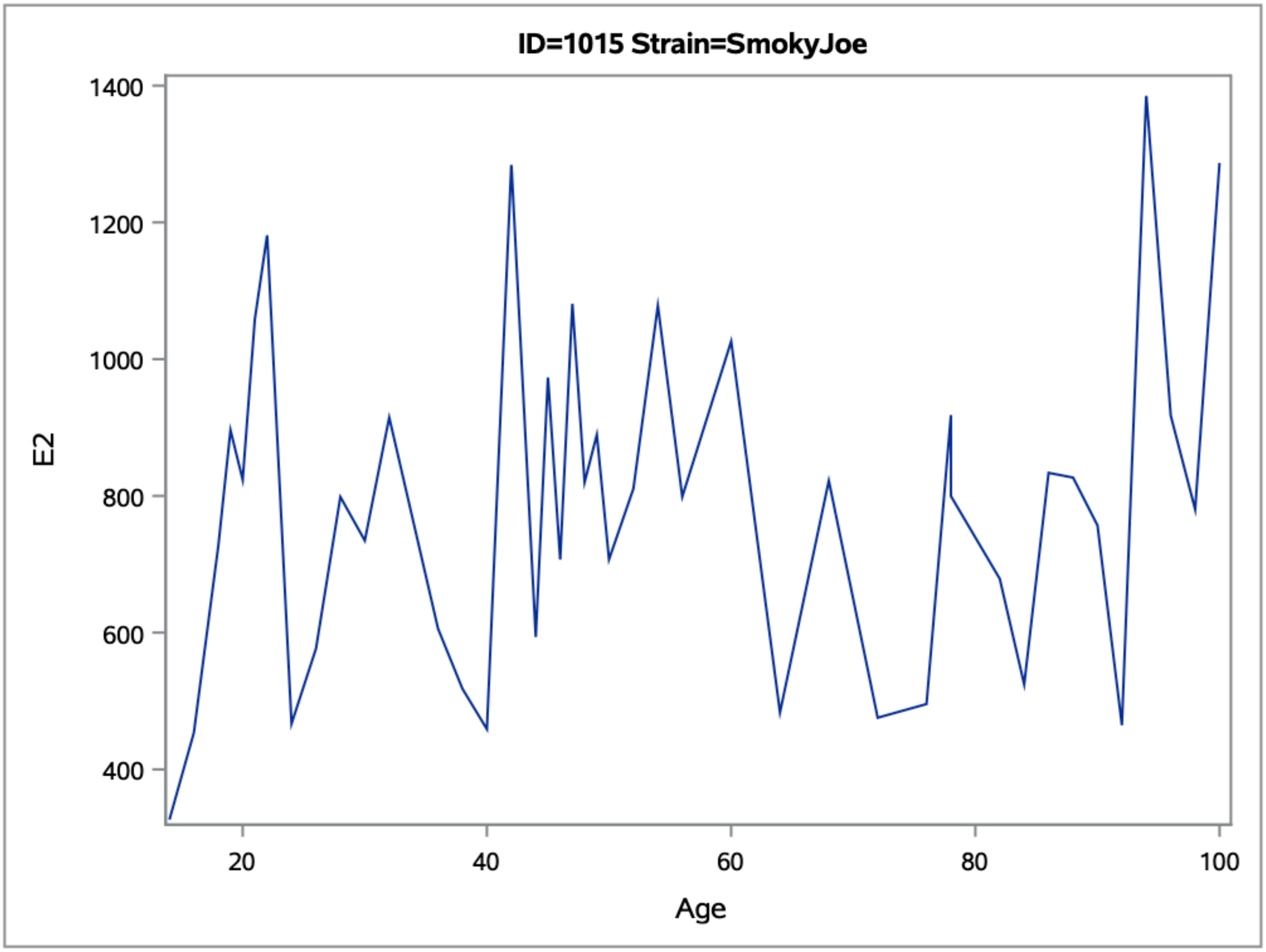

ID=1015 Strain=SmokyJoe
1400
1200
1000
800
600
400
E2
20
40
60
80
100
Age

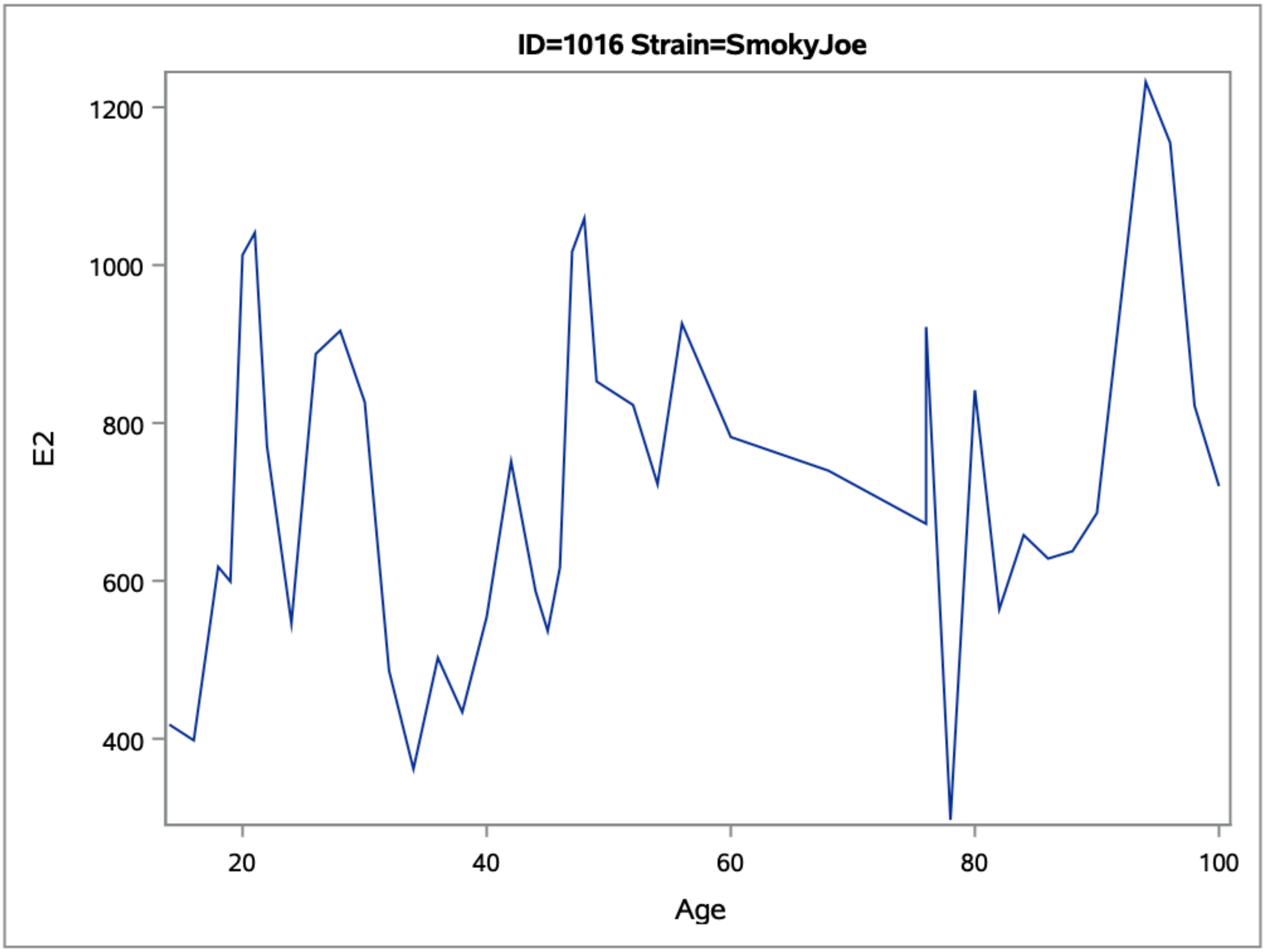

ID=1016 Strain=SmokyJoe
1200
1000
800
600
400
E2
20
40
60
80
100
Age

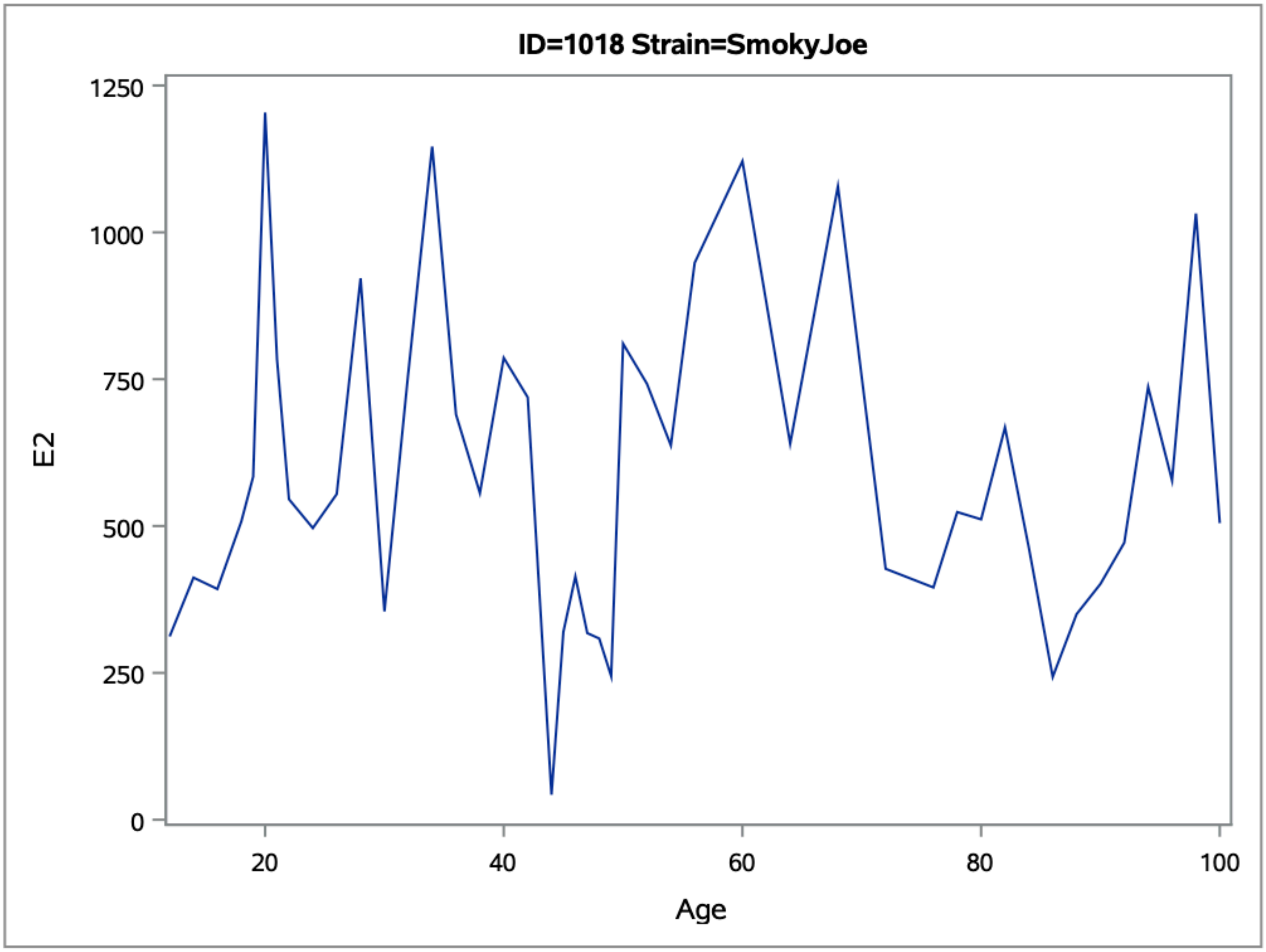

ID=1018 Strain=SmokyJoe
1250
1000
750
500
250
0
E2
20
40
60
80
100
Age

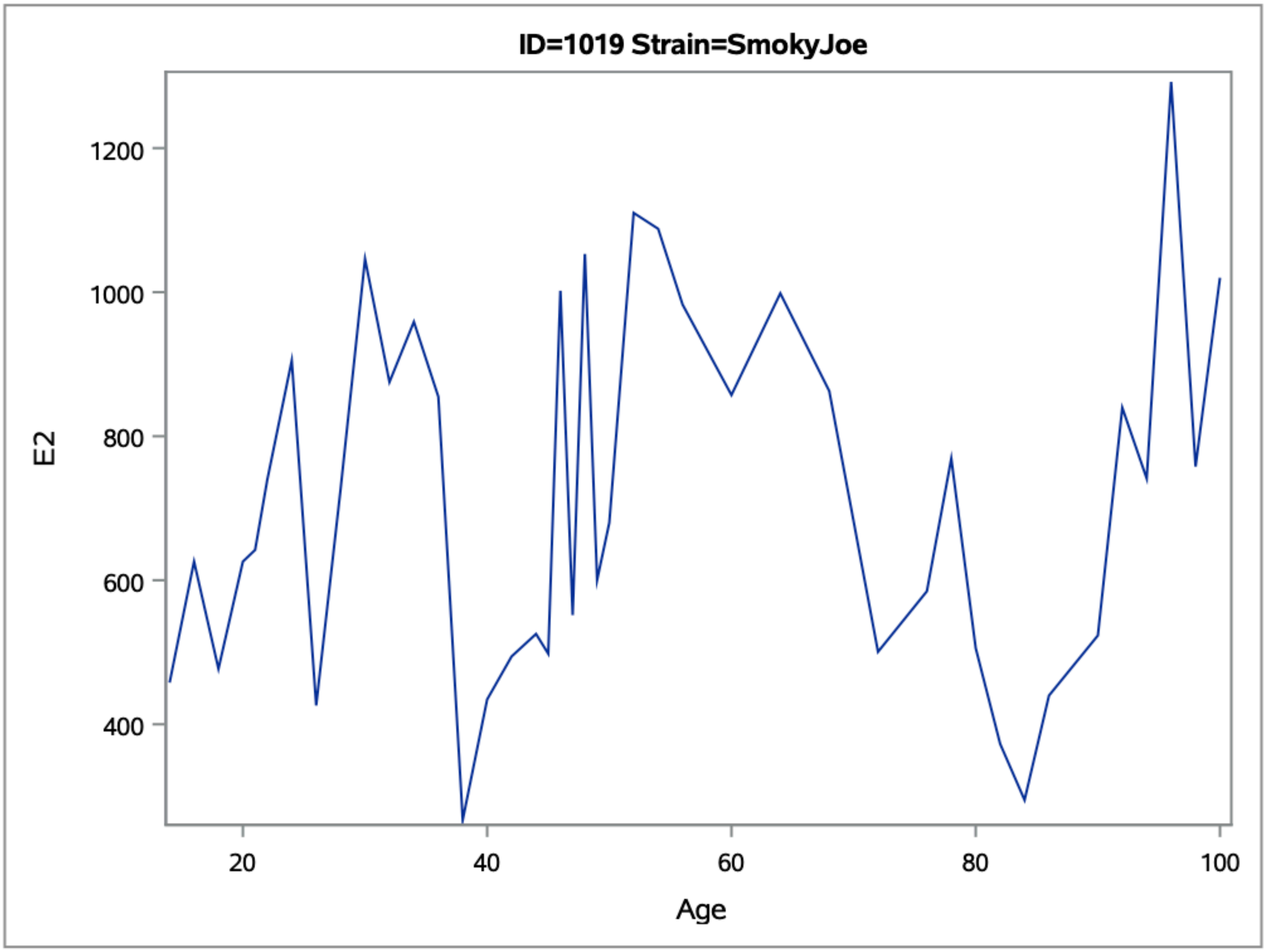

ID=1019 Strain=SmokyJoe
1200
1000
800
600
400
E2
20
40
60
80
100
Age

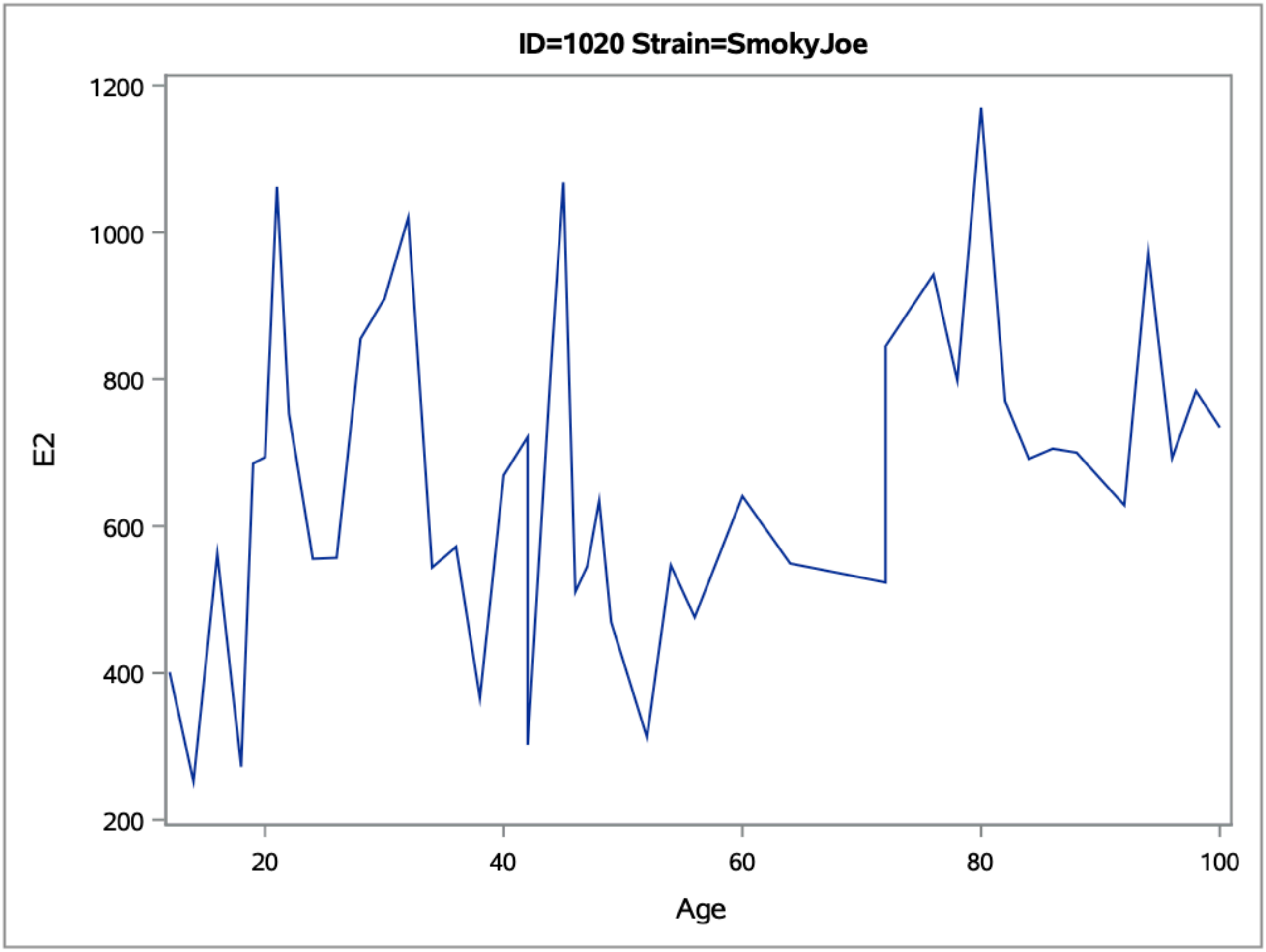

ID=1020 Strain=SmokyJoe
E2
1200
1000
800
600
400
200
20
40
60
80
100
Age

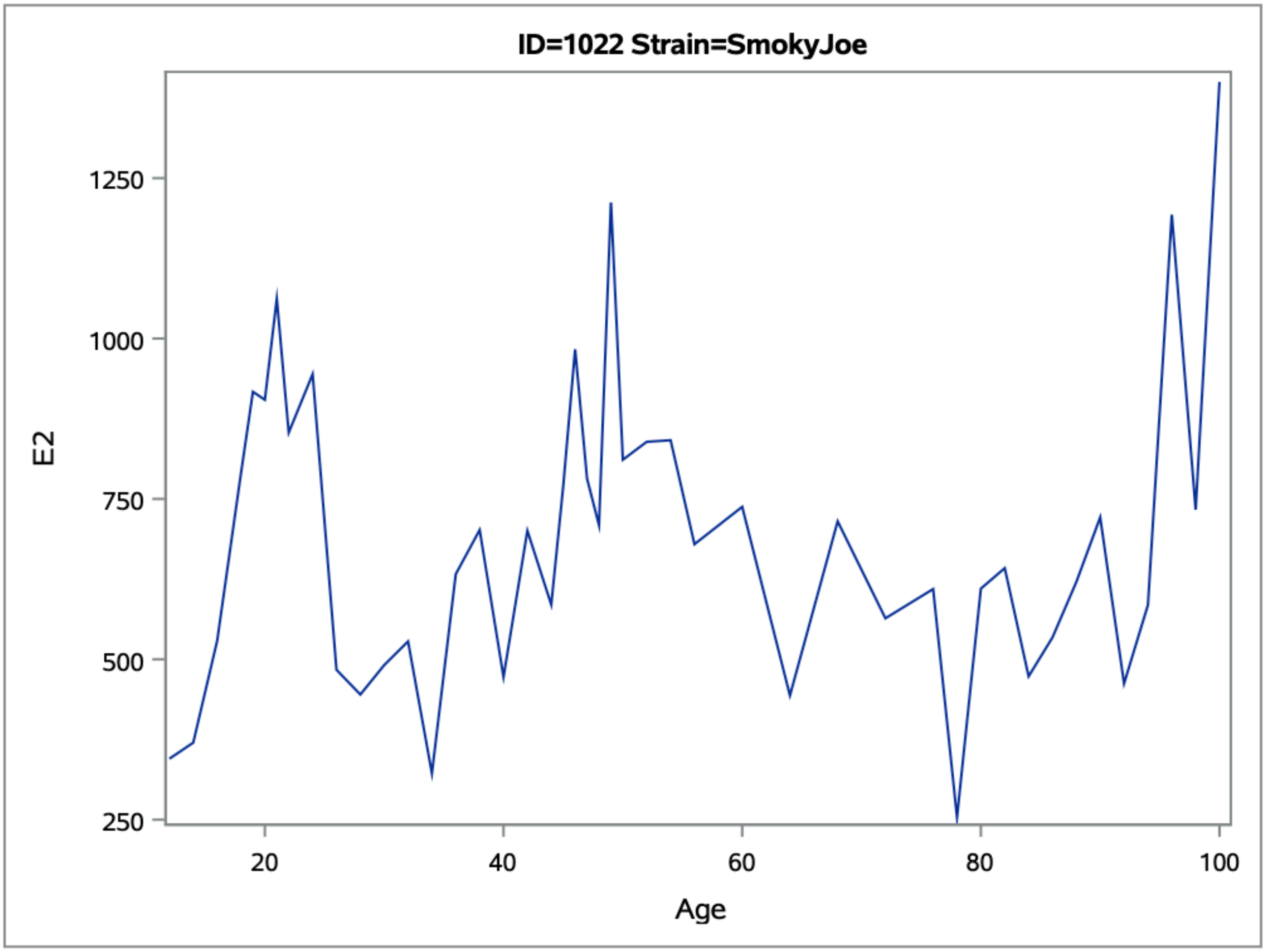
ID=1022 Strain=SmokyJoe
1250
1000
750
500
250
E2
20
40
60
80
100
Age

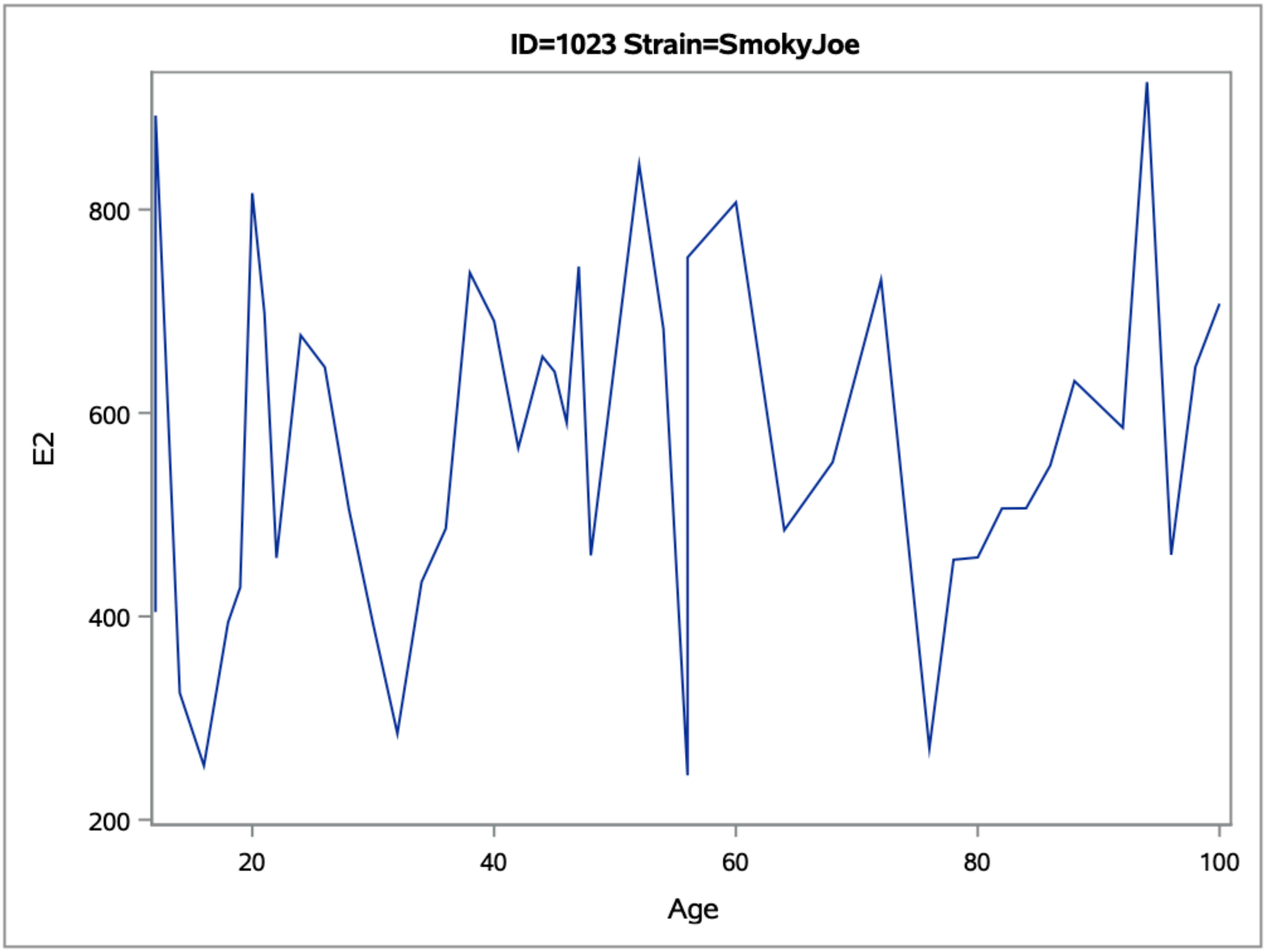
ID=1023 Strain=SmokyJoe
E2
800
600
400
200
20
40
60
80
100
Age

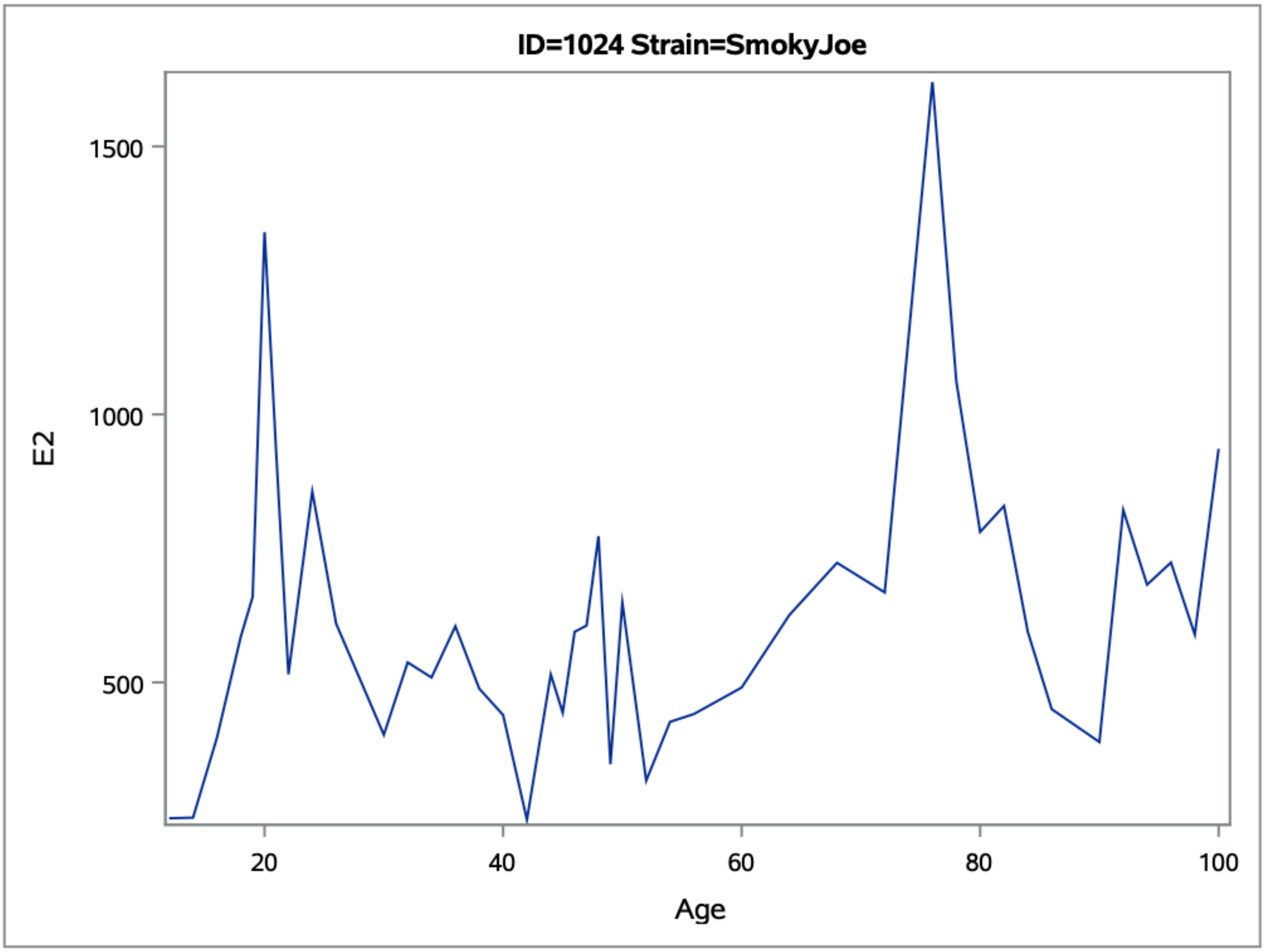
ID=1024 Strain=SmokyJoe
1500
1000
500
E2
20
40
60
80
100
Age

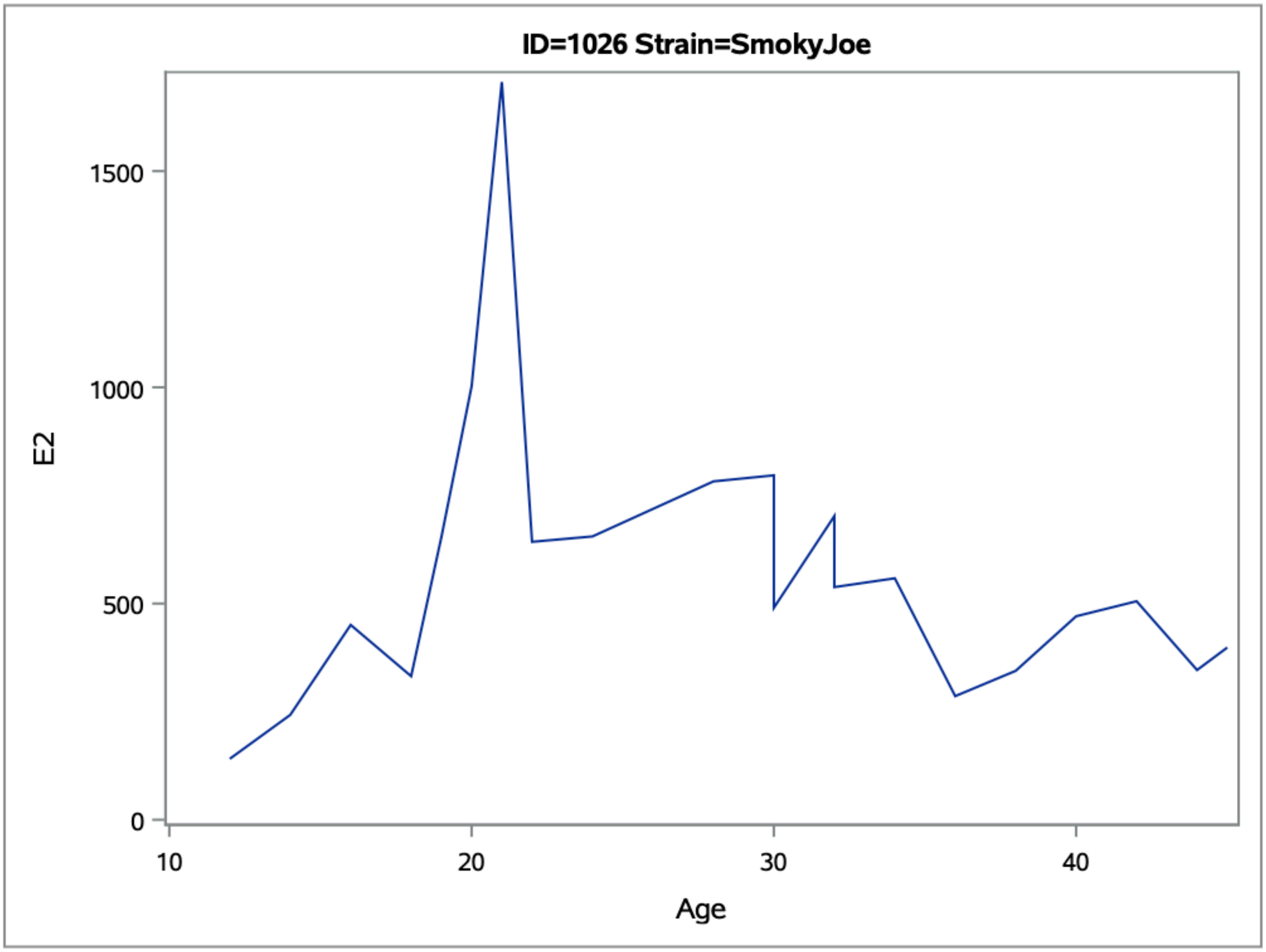

ID=1026 Strain=SmokyJoe
1500
1000
500
0
E2
10
20
30
40
Age

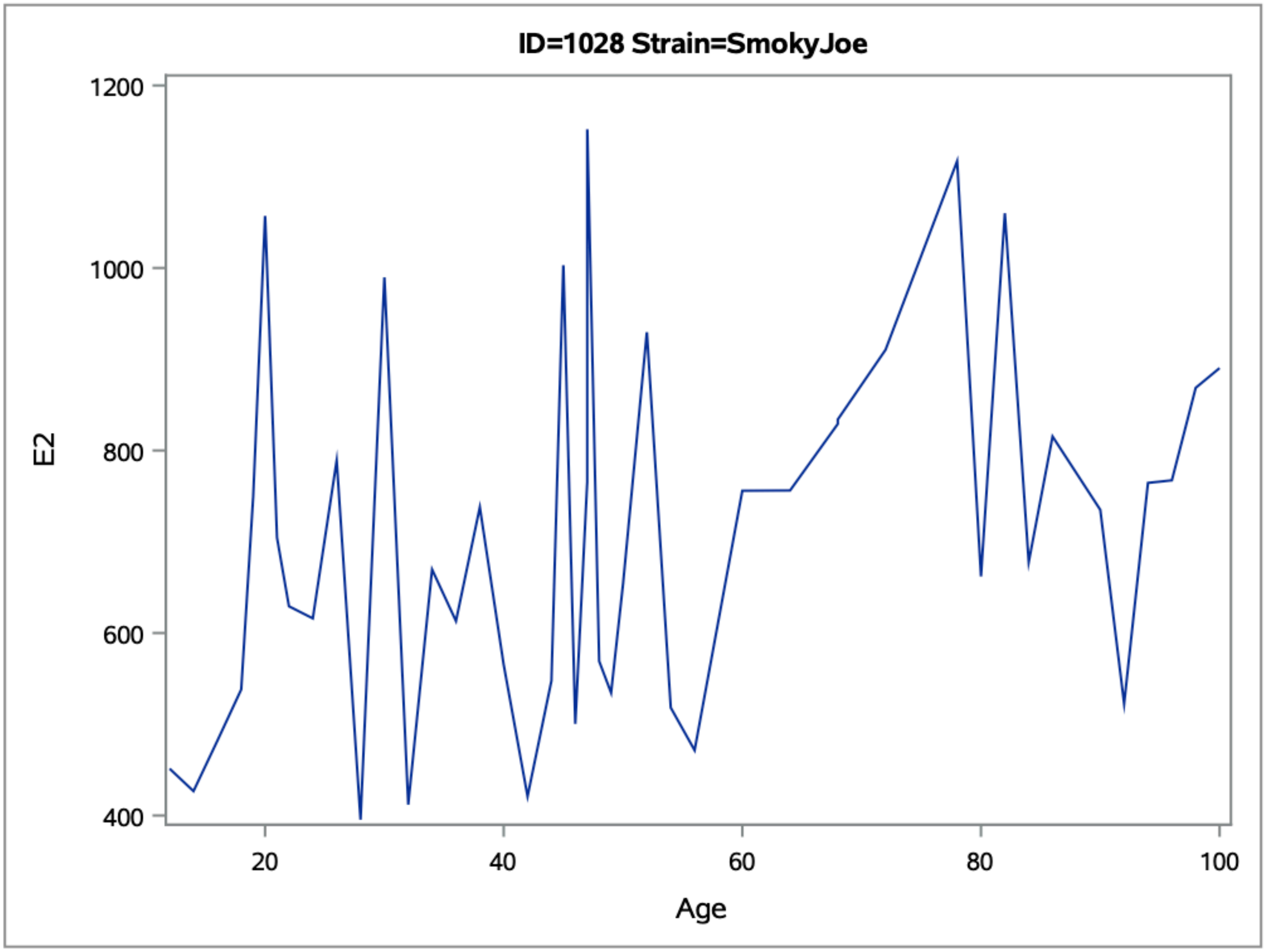
ID=1028 Strain=SmokyJoe
1200
1000
800
600
400
E2
20
40
60
80
100
Age

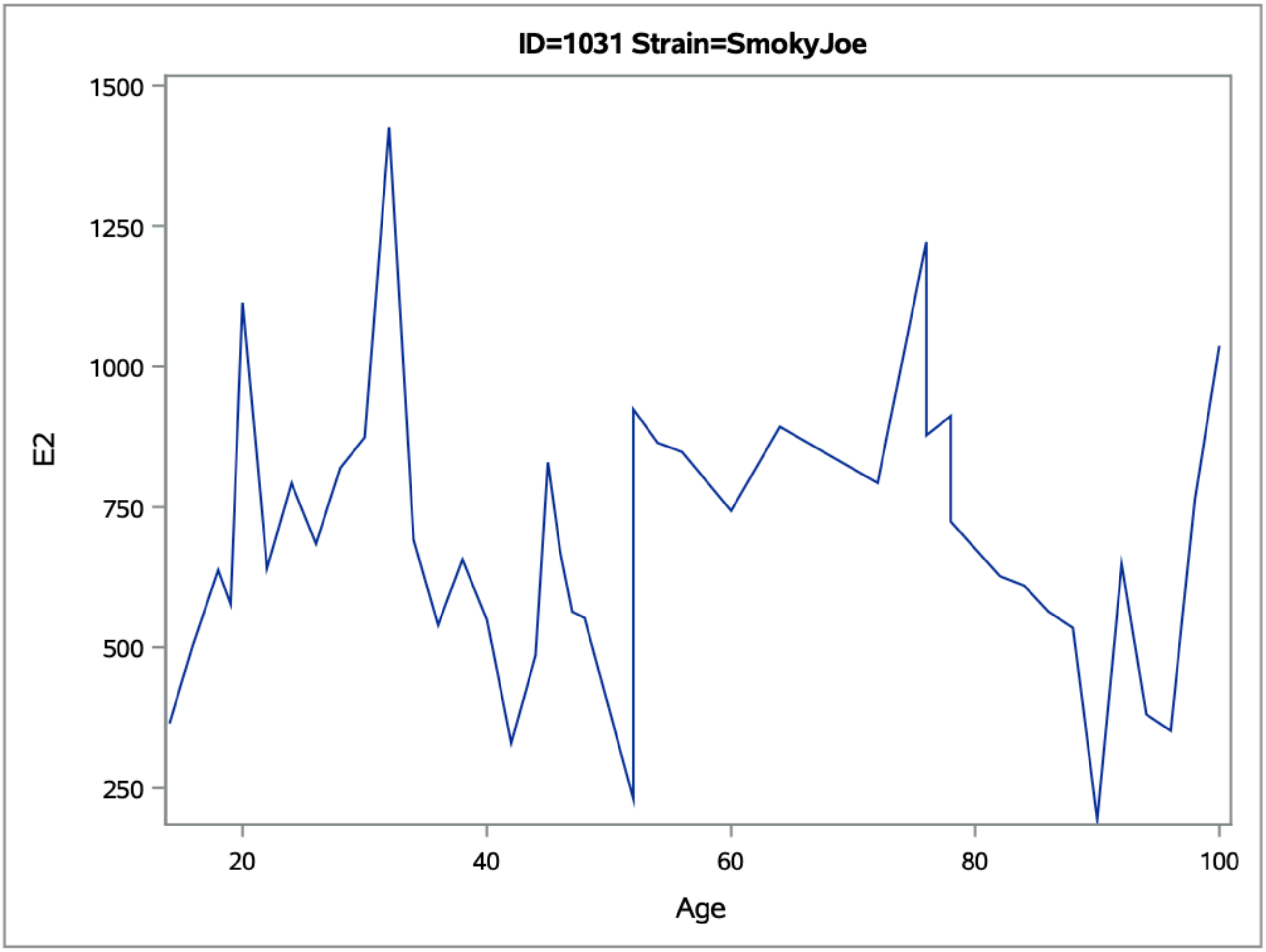
ID=1031 Strain=SmokyJoe
1500
1250
1000
750
500
250
E2
20
40
60
80
100
Age

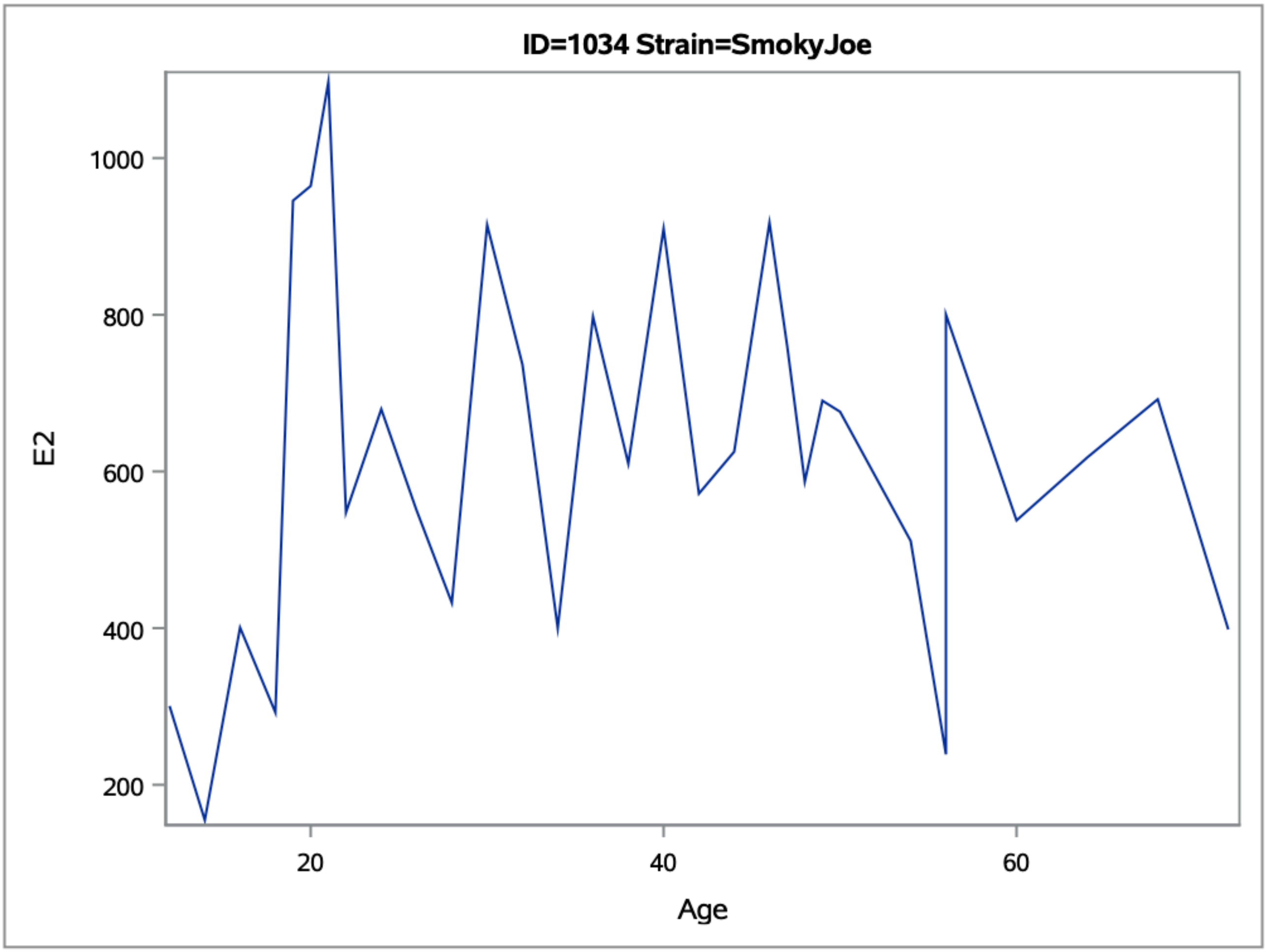
ID=1034 Strain=SmokyJoe
E2
1000
800
600
400
200
20
40
60
Age

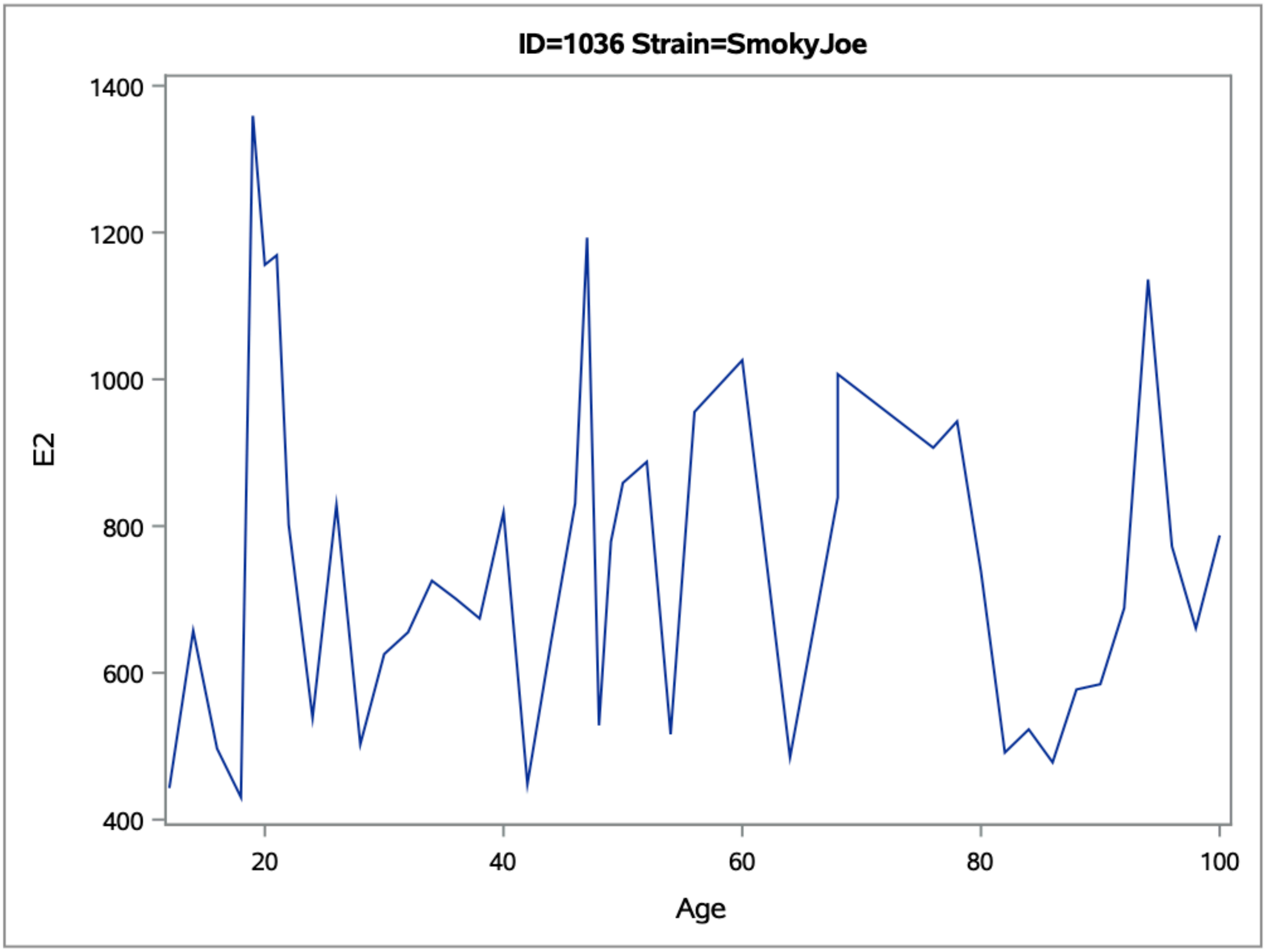

ID=1036 Strain=SmokyJoe
E2
1400
1200
1000
800
600
400
20
40
60
80
100
Age

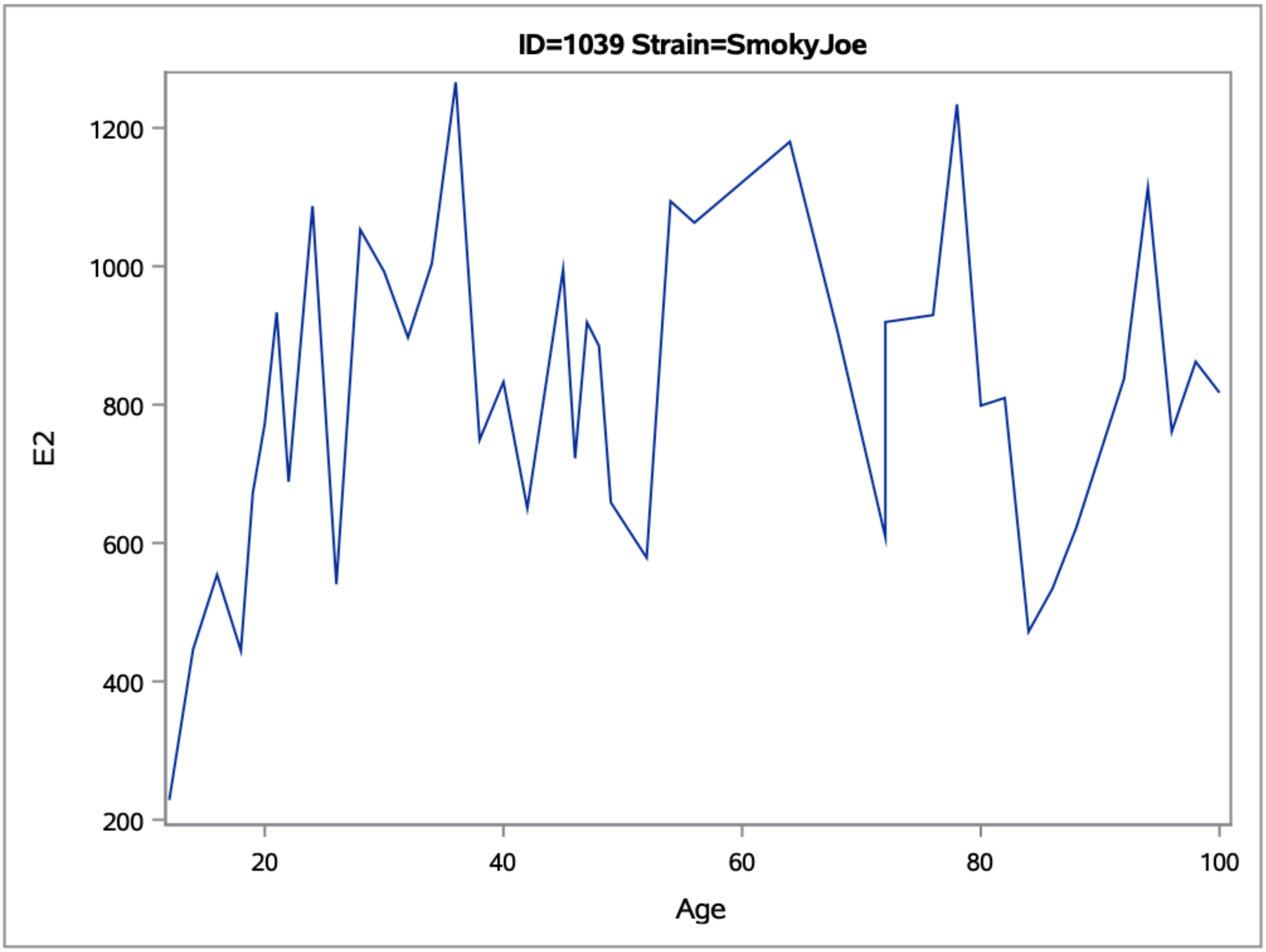

ID=1039 Strain=SmokyJoe
1200
1000
800
600
400
200
E2
20
40
60
80
100
Age

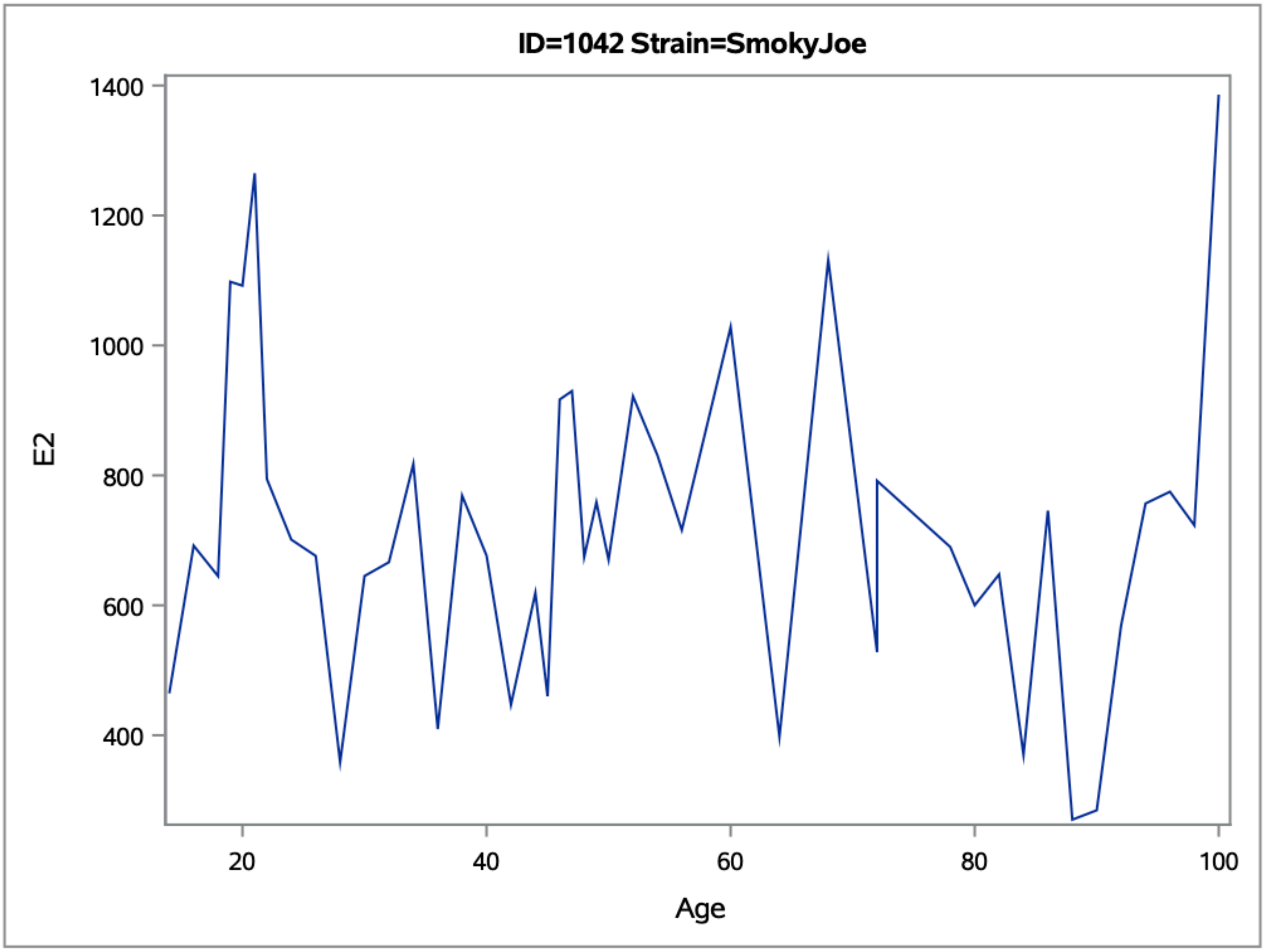

ID=1042 Strain=SmokyJoe
1400
1200
1000
800
600
400
E2
20
40
60
80
100
Age

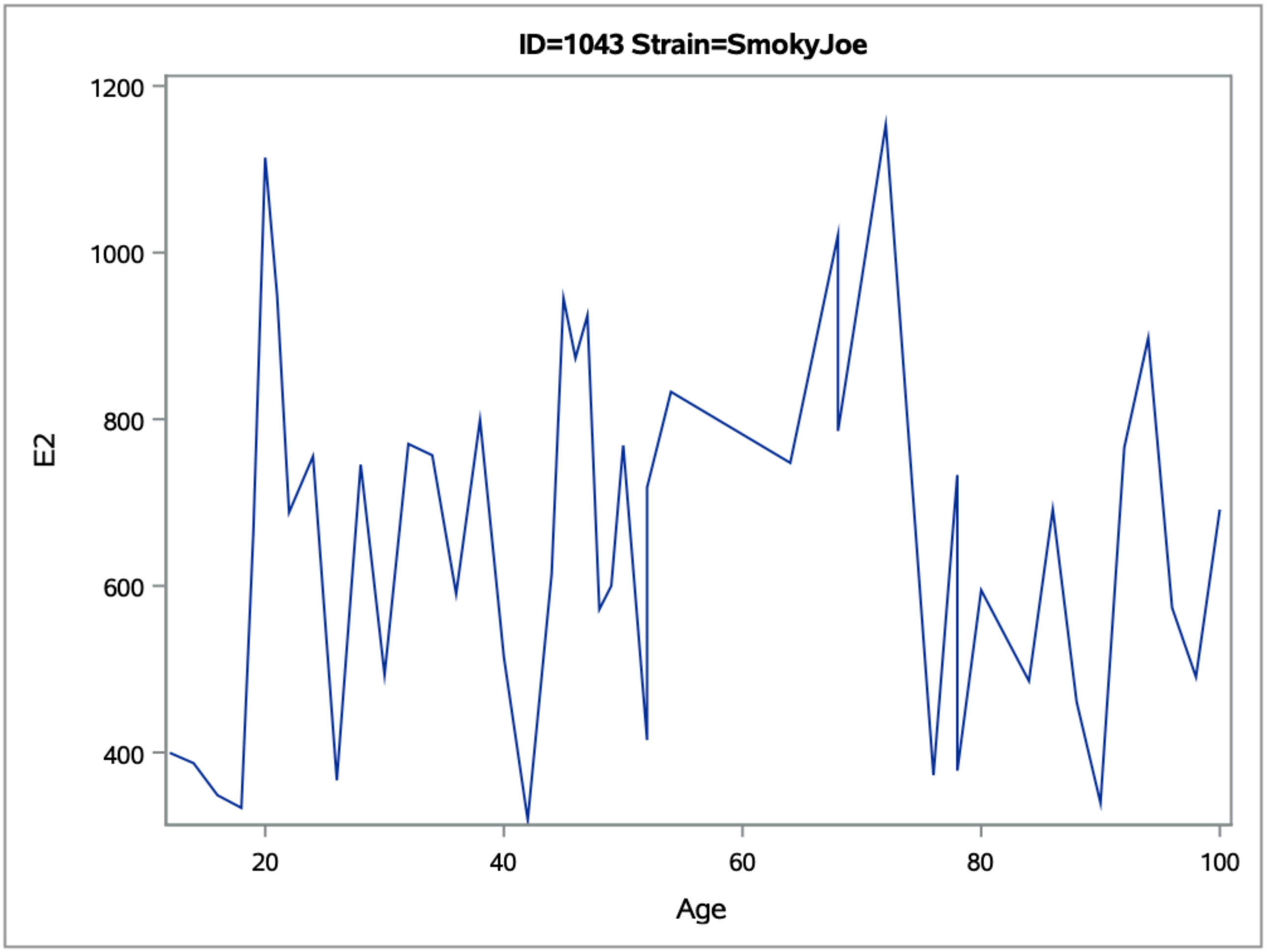

ID=1043 Strain=SmokyJoe
1200
1000
800
600
400
E2
20
40
60
80
100
Age

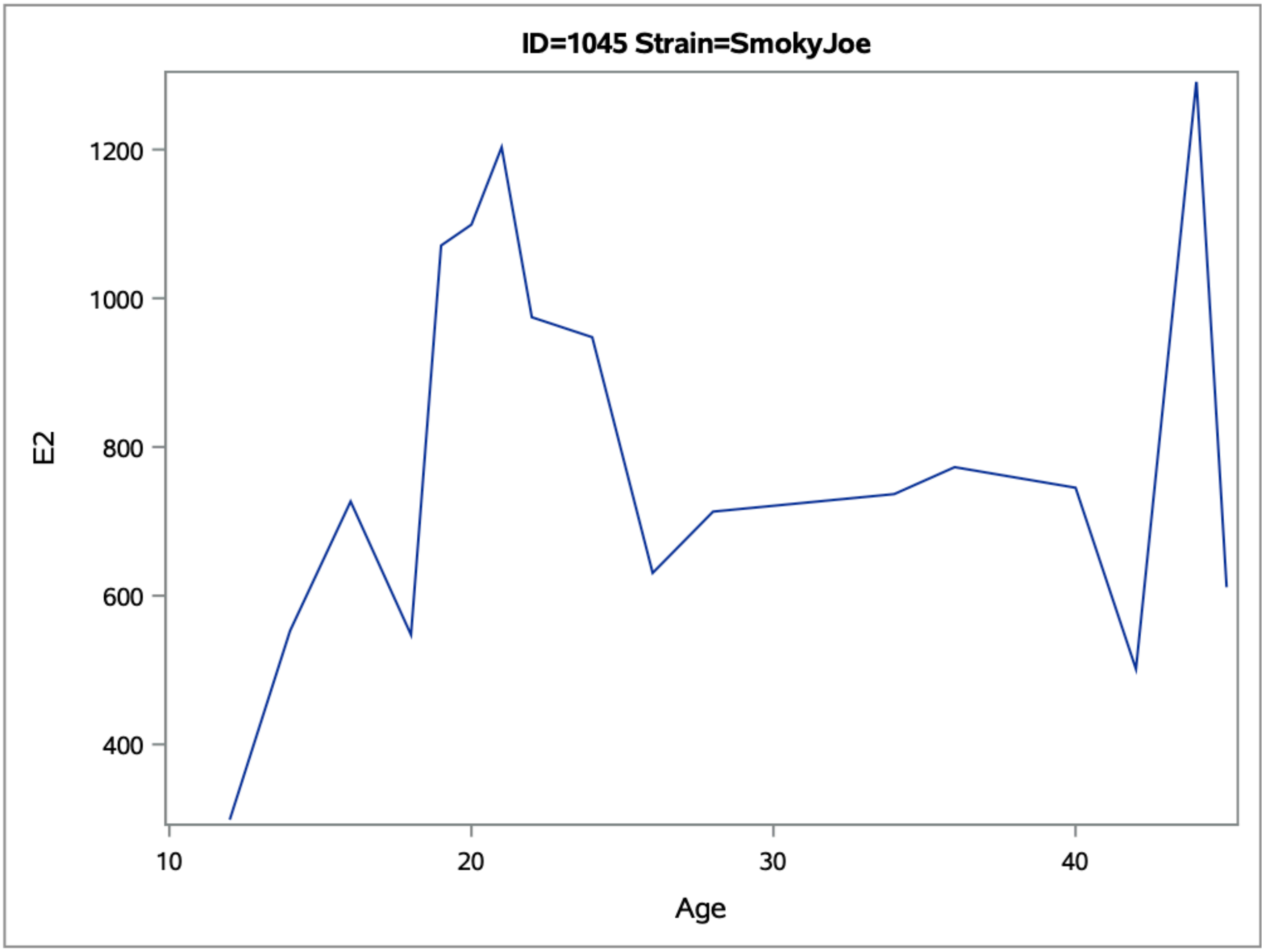

ID=1045 Strain=SmokyJoe
1200
1000
800
600
400
E2
10
20
30
40
Age

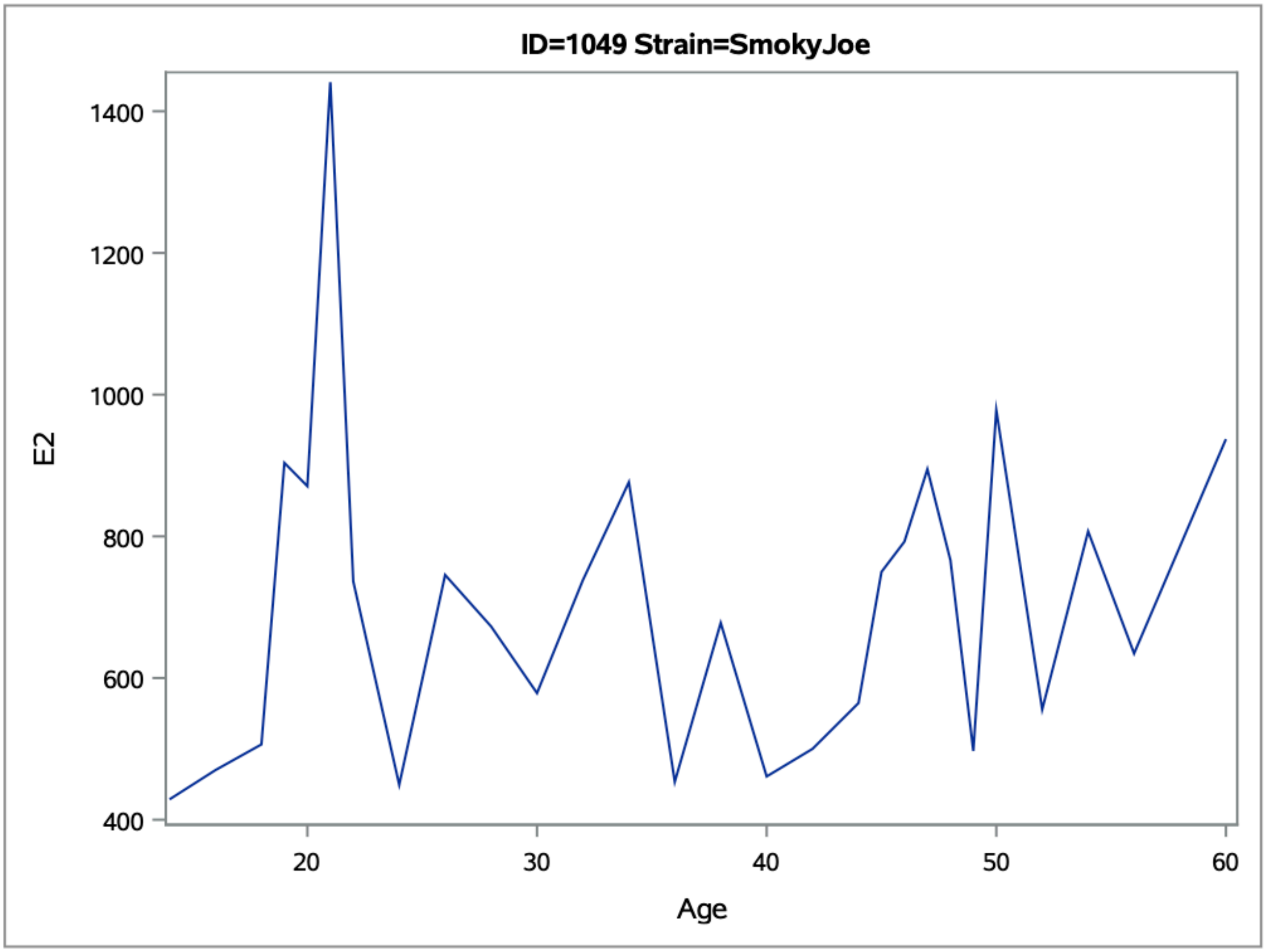
ID=1049 Strain=SmokyJoe
1400
1200
1000
800
600
400
E2
20
30
40
50
60
Age

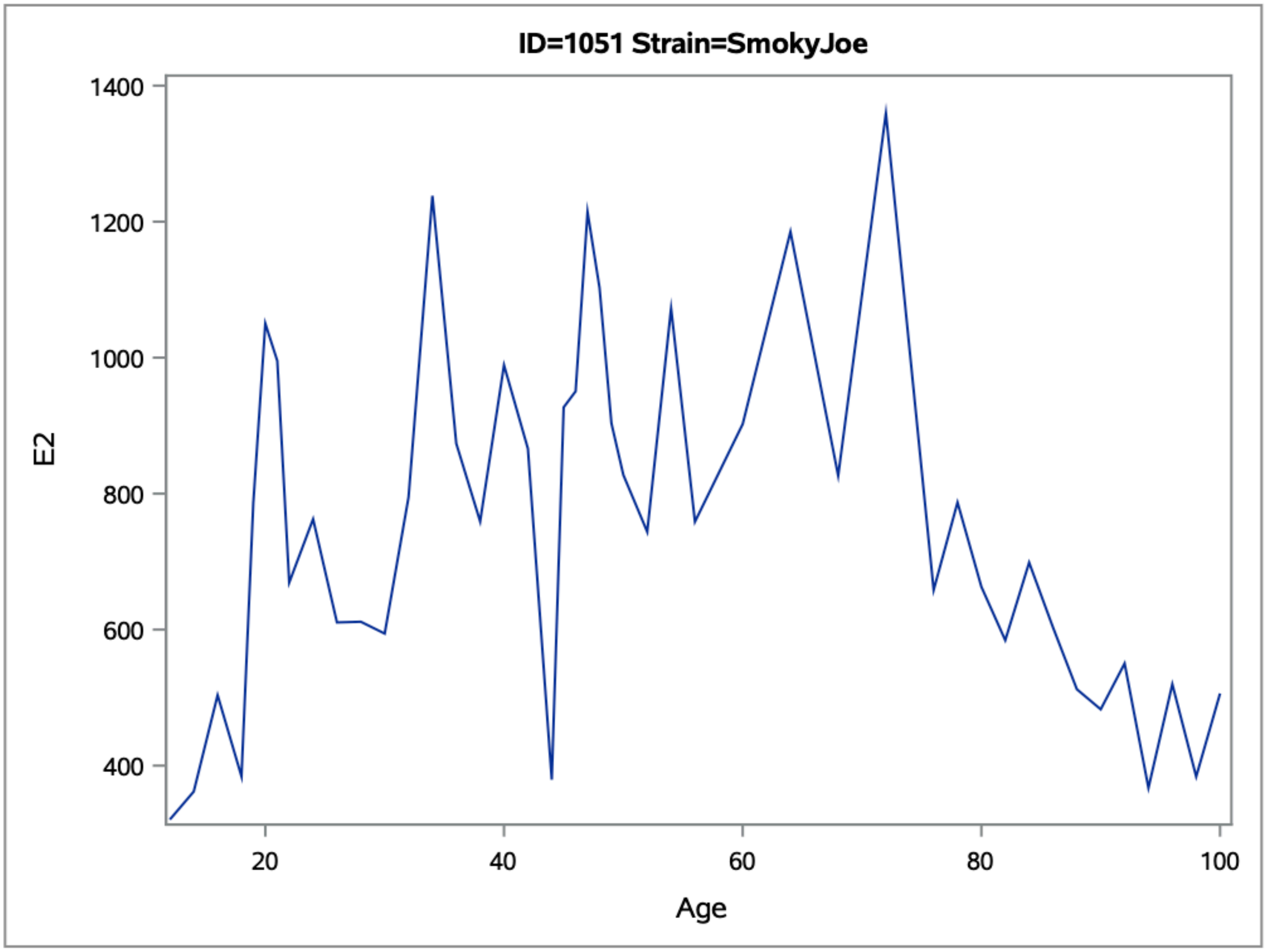
ID=1051 Strain=SmokyJoe
1400
1200
1000
800
600
400
E2
20
40
60
80
100
Age

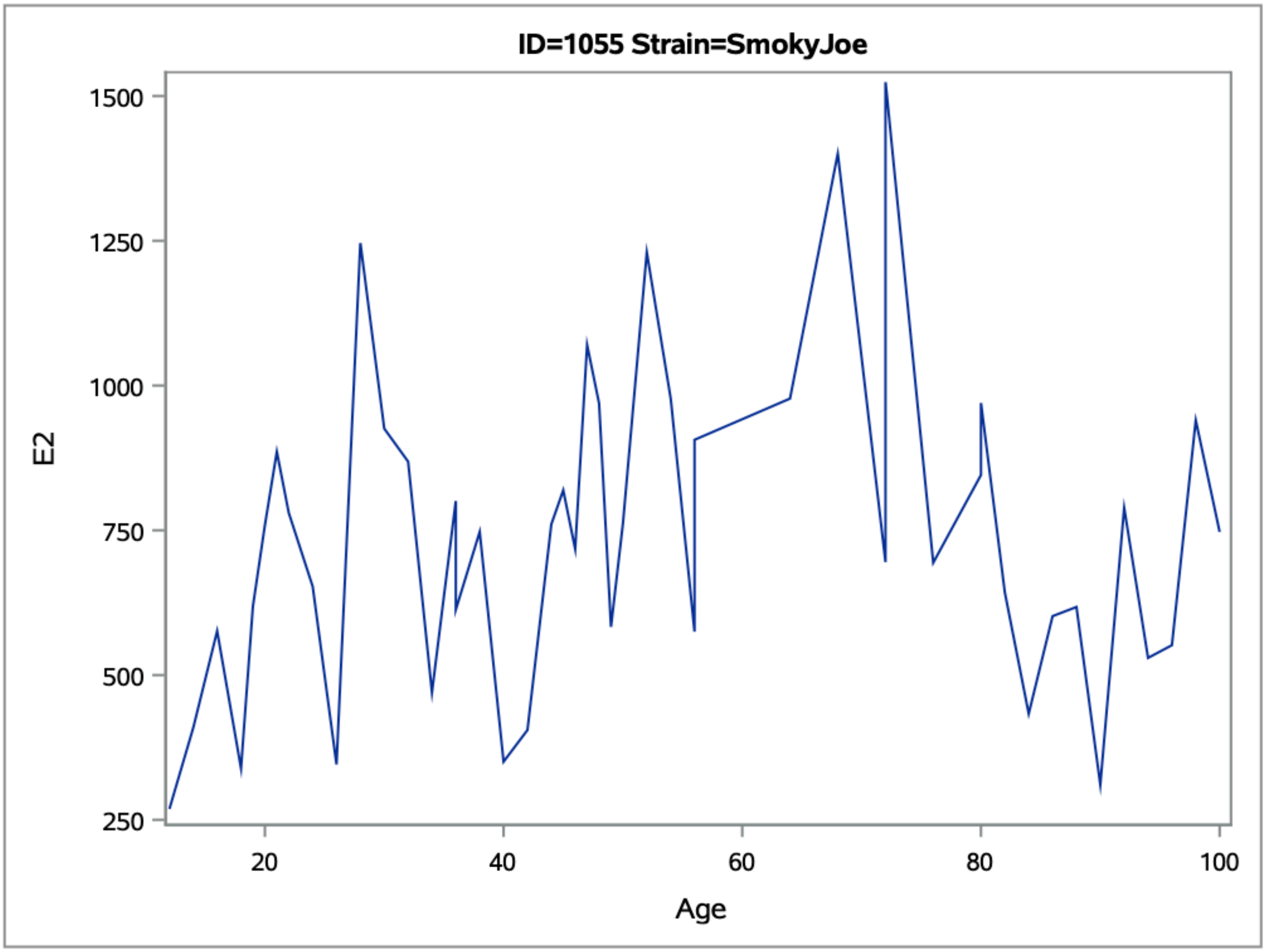
ID=1055 Strain=SmokyJoe
1500
1250
1000
750
500
250
E2
20
40
60
80
100
Age

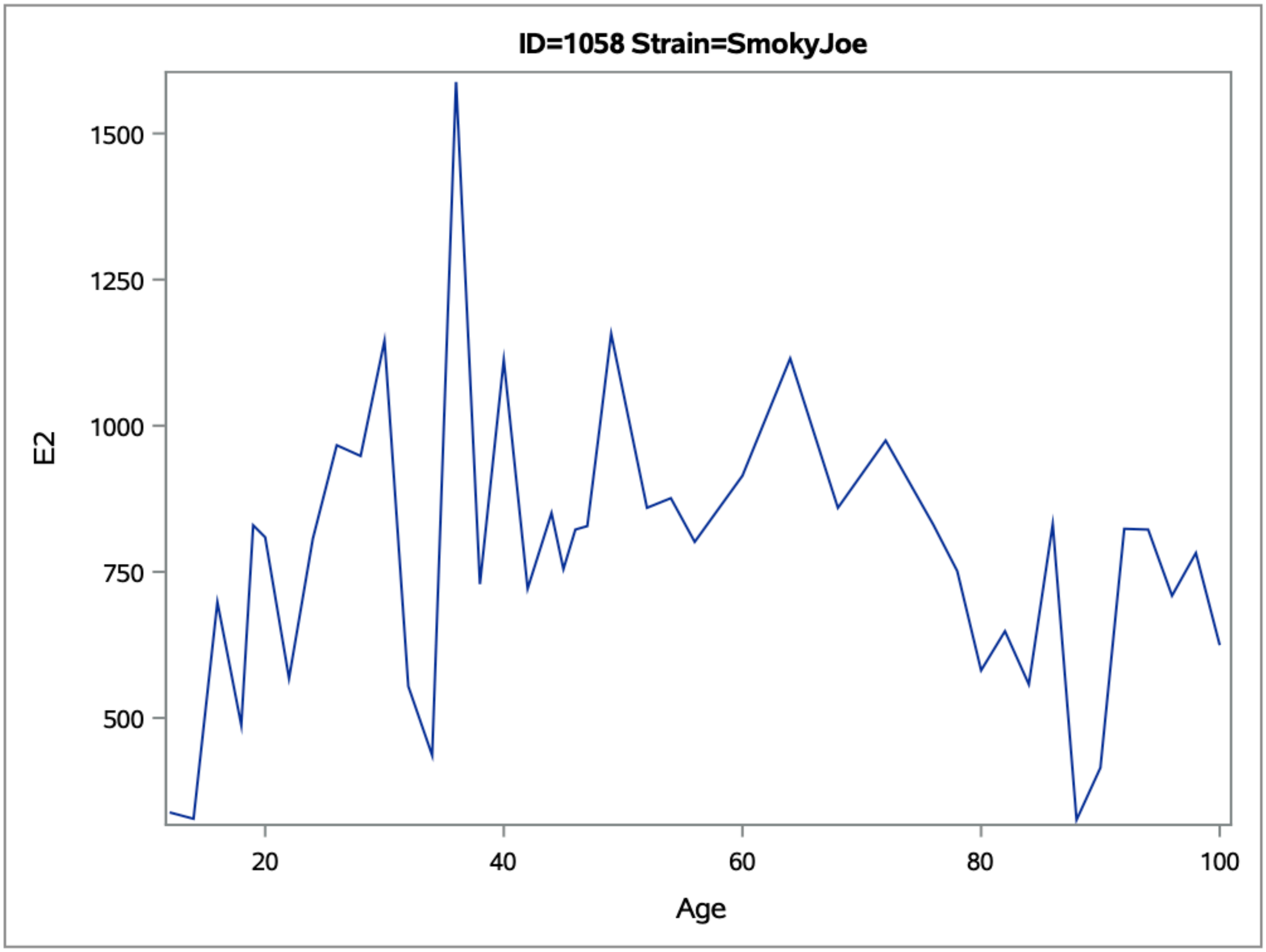
ID=1058 Strain=SmokyJoe
E2
1500
1250
1000
750
500
20
40
60
80
100
Age

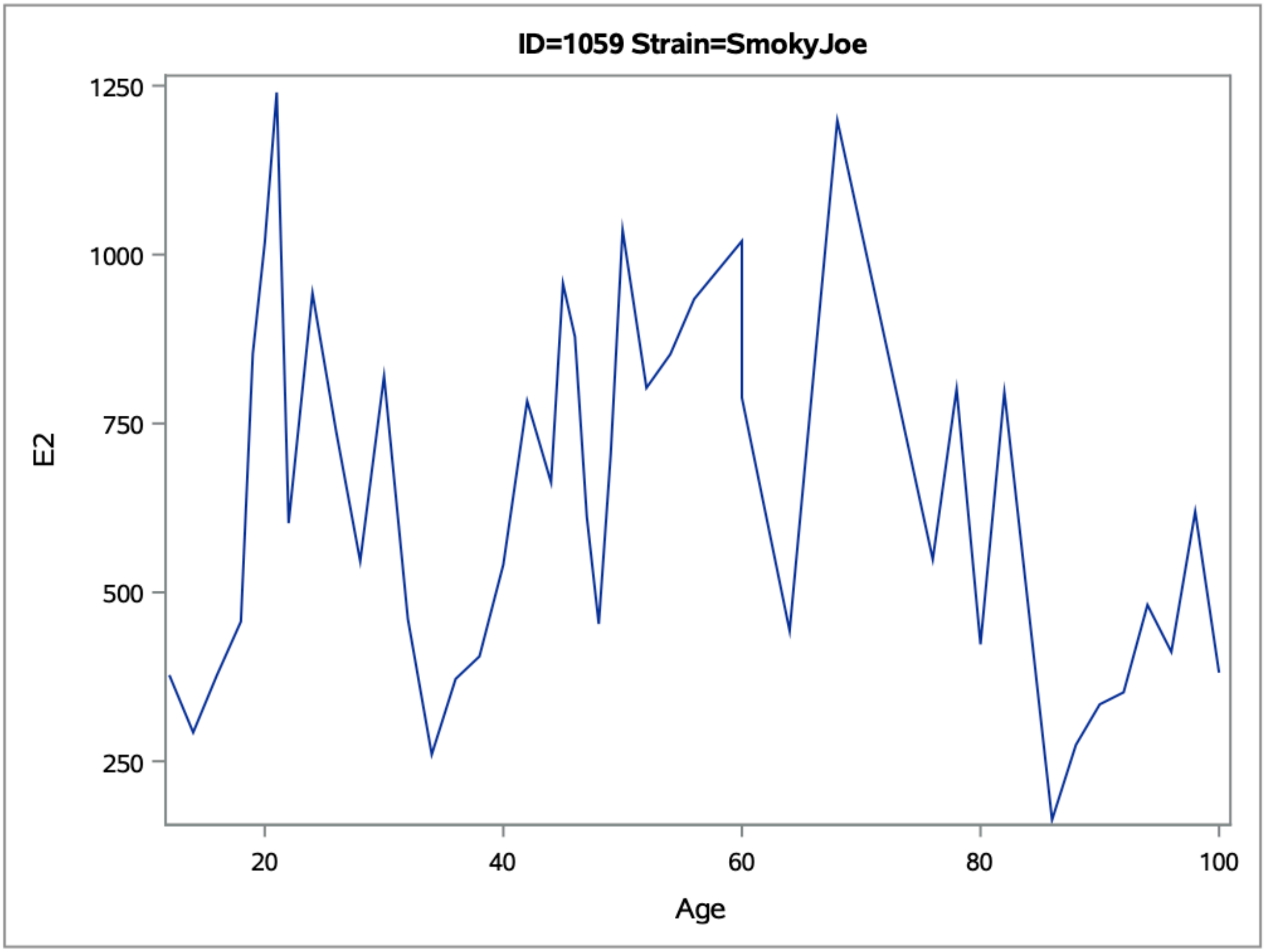
ID=1059 Strain=SmokyJoe
1250
1000
750
500
250
E2
20
40
60
80
100
Age

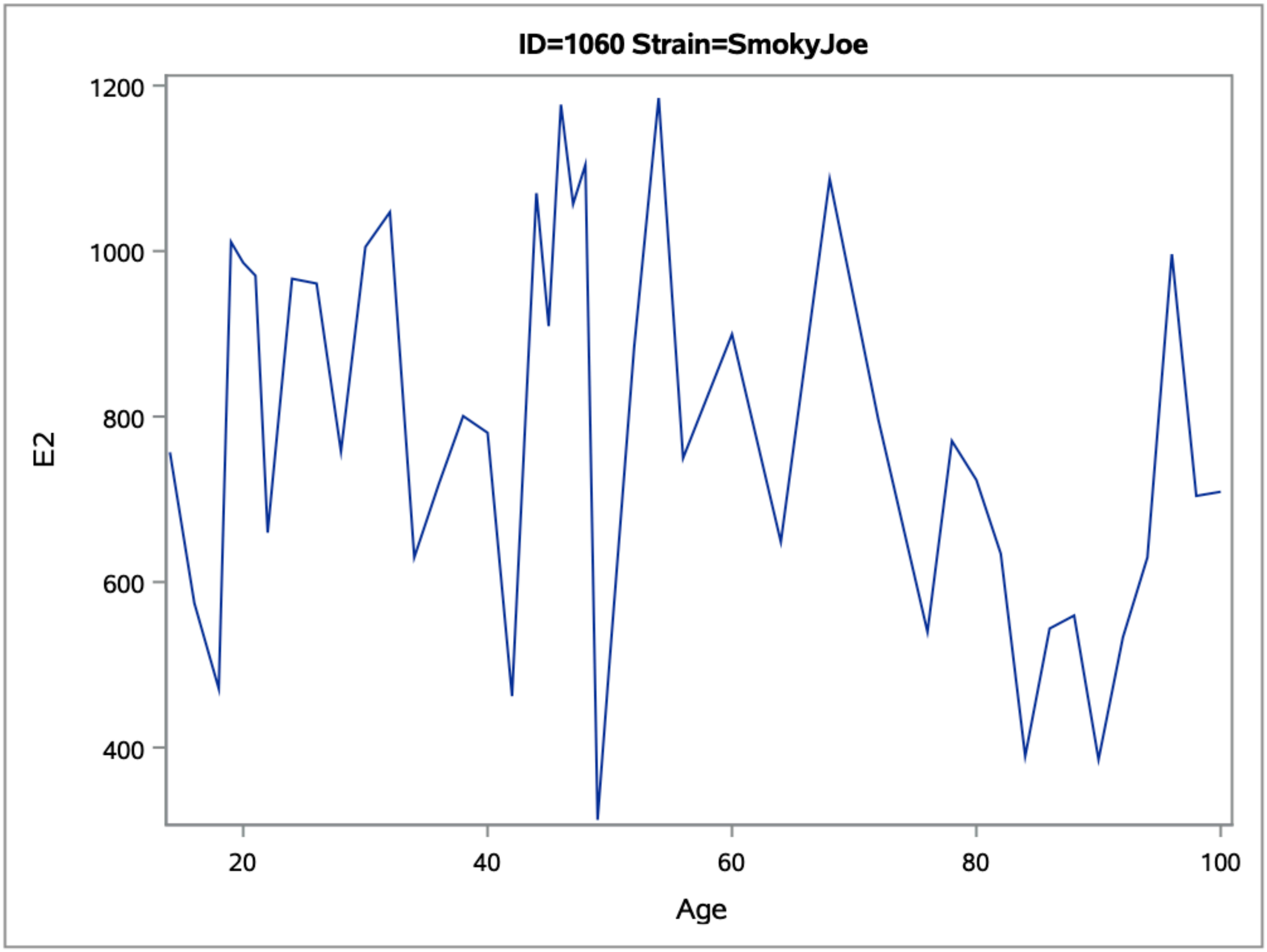
ID=1060 Strain=SmokyJoe
E2
1200
1000
800
600
400
20
40
60
80
100
Age

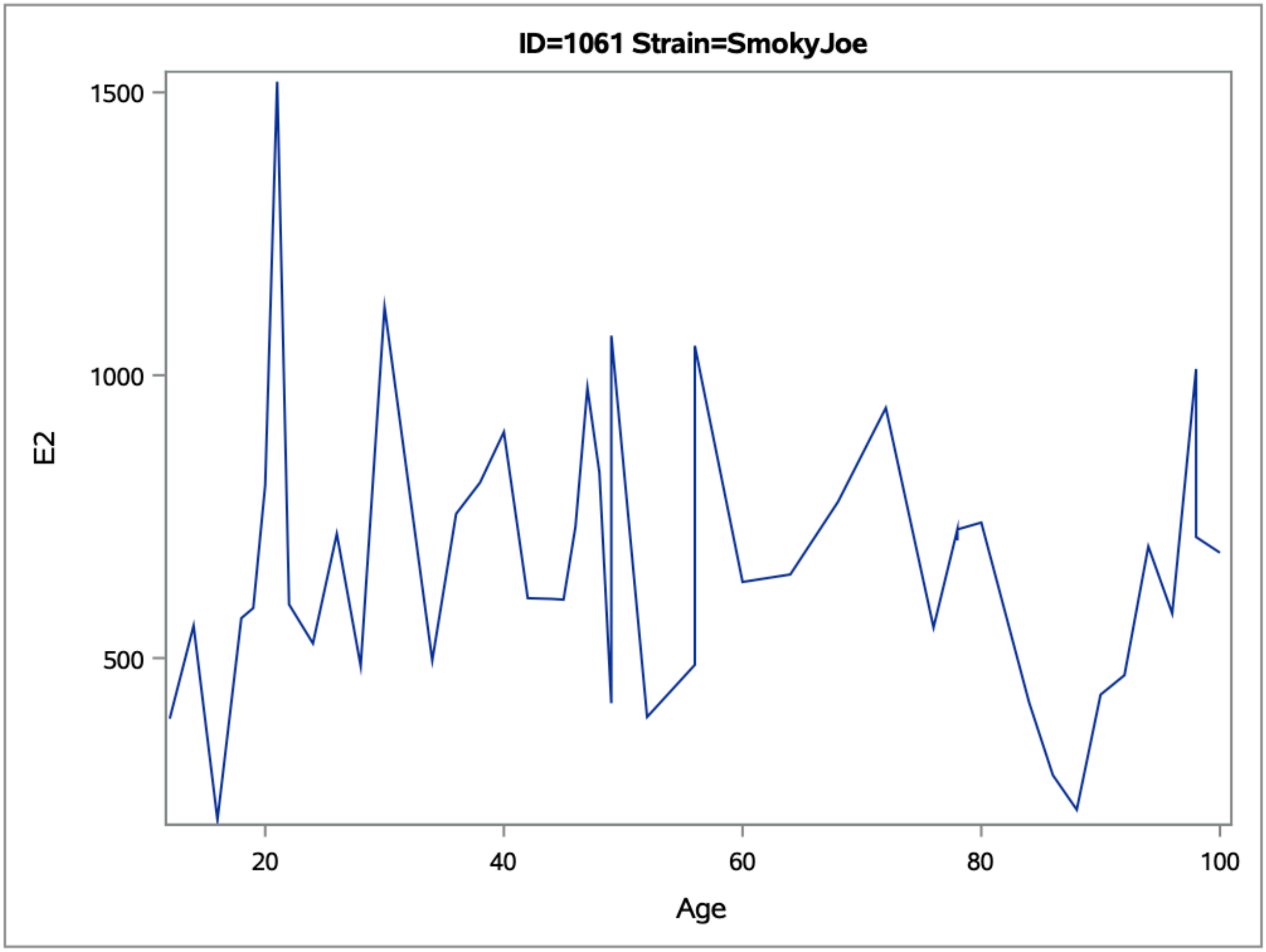
ID=1061 Strain=SmokyJoe
1500
1000
500
E2
20
40
60
80
100
Age

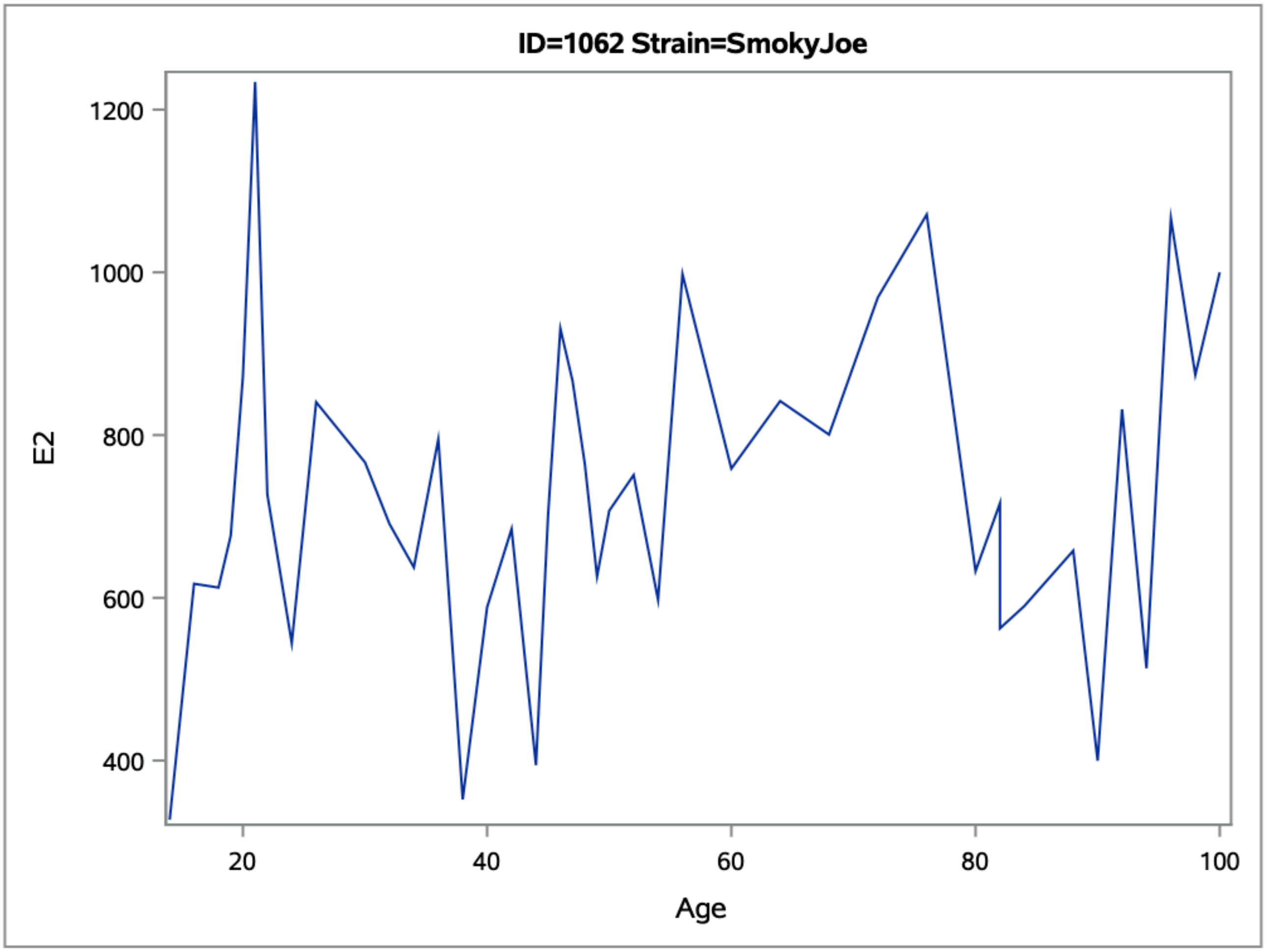

ID=1062 Strain=SmokyJoe
1200
1000
800
600
400
E2
20
40
60
80
100
Age

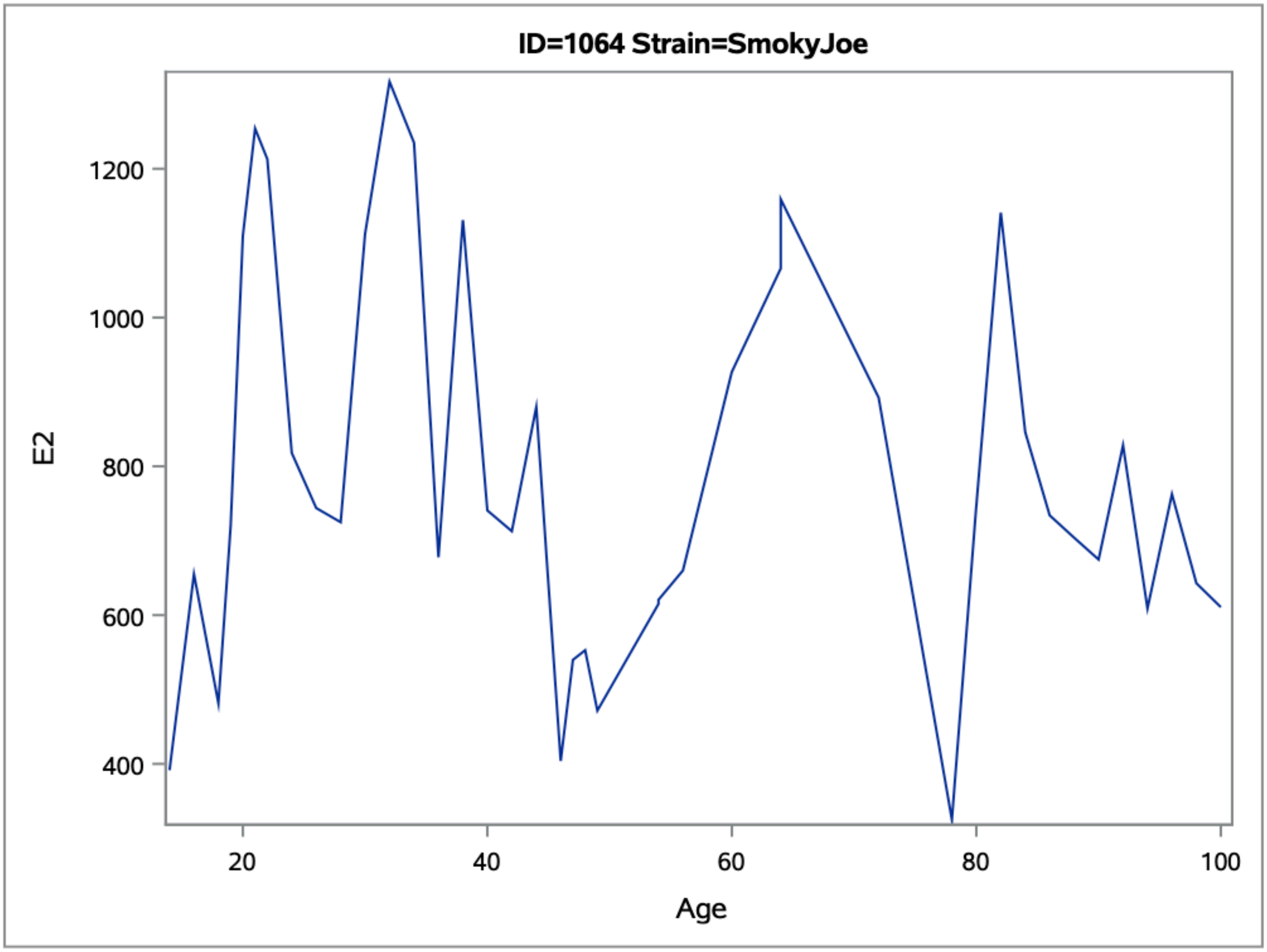
ID=1064 Strain=SmokyJoe
E2
1200
1000
800
600
400
20
40
60
80
100
Age

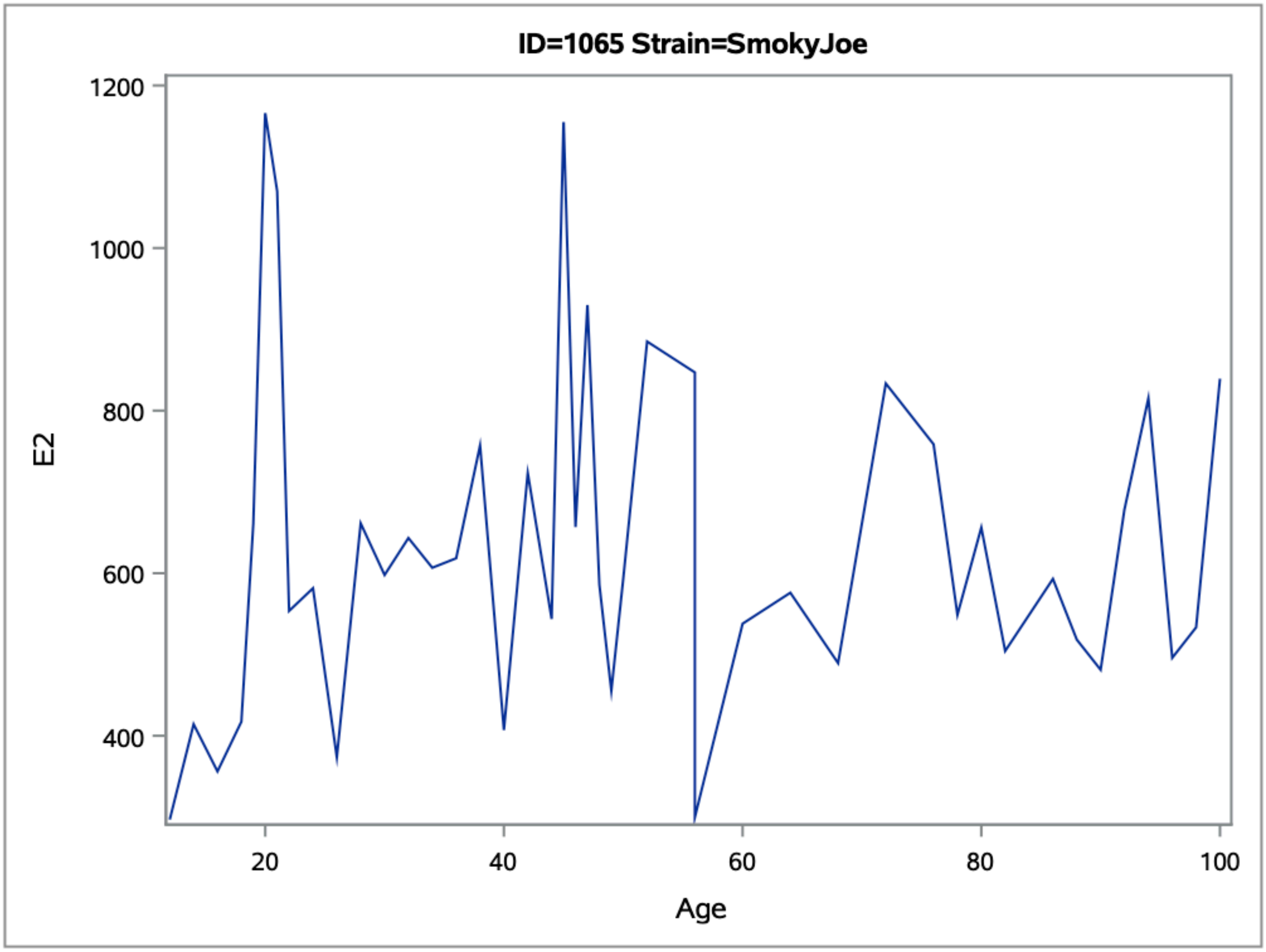
ID=1065 Strain=SmokyJoe
1200
1000
800
600
400
E2
20
40
60
80
100
Age

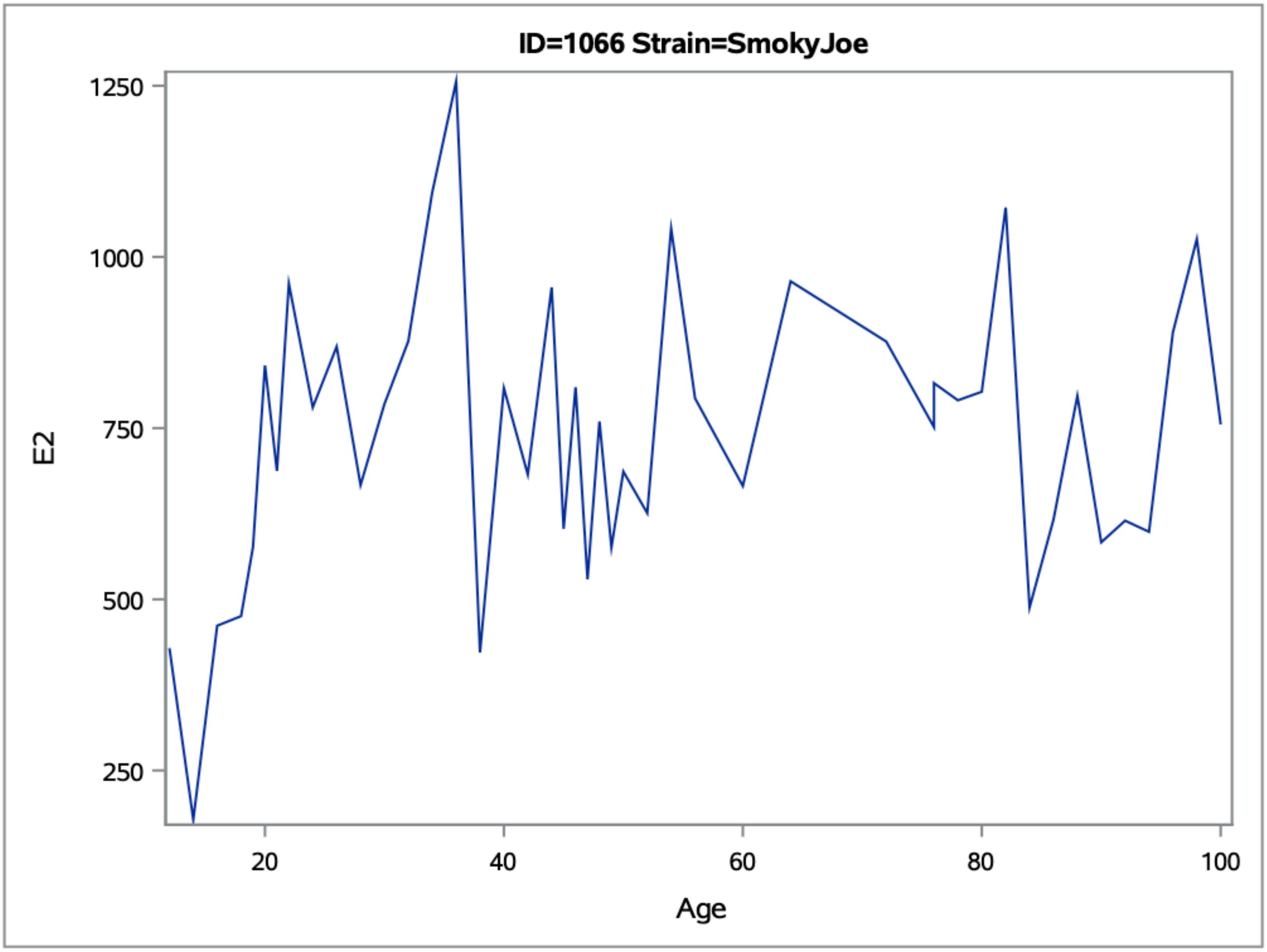
ID=1066 Strain=SmokyJoe
E2
1250
1000
750
500
250
20
40
60
80
100
Age

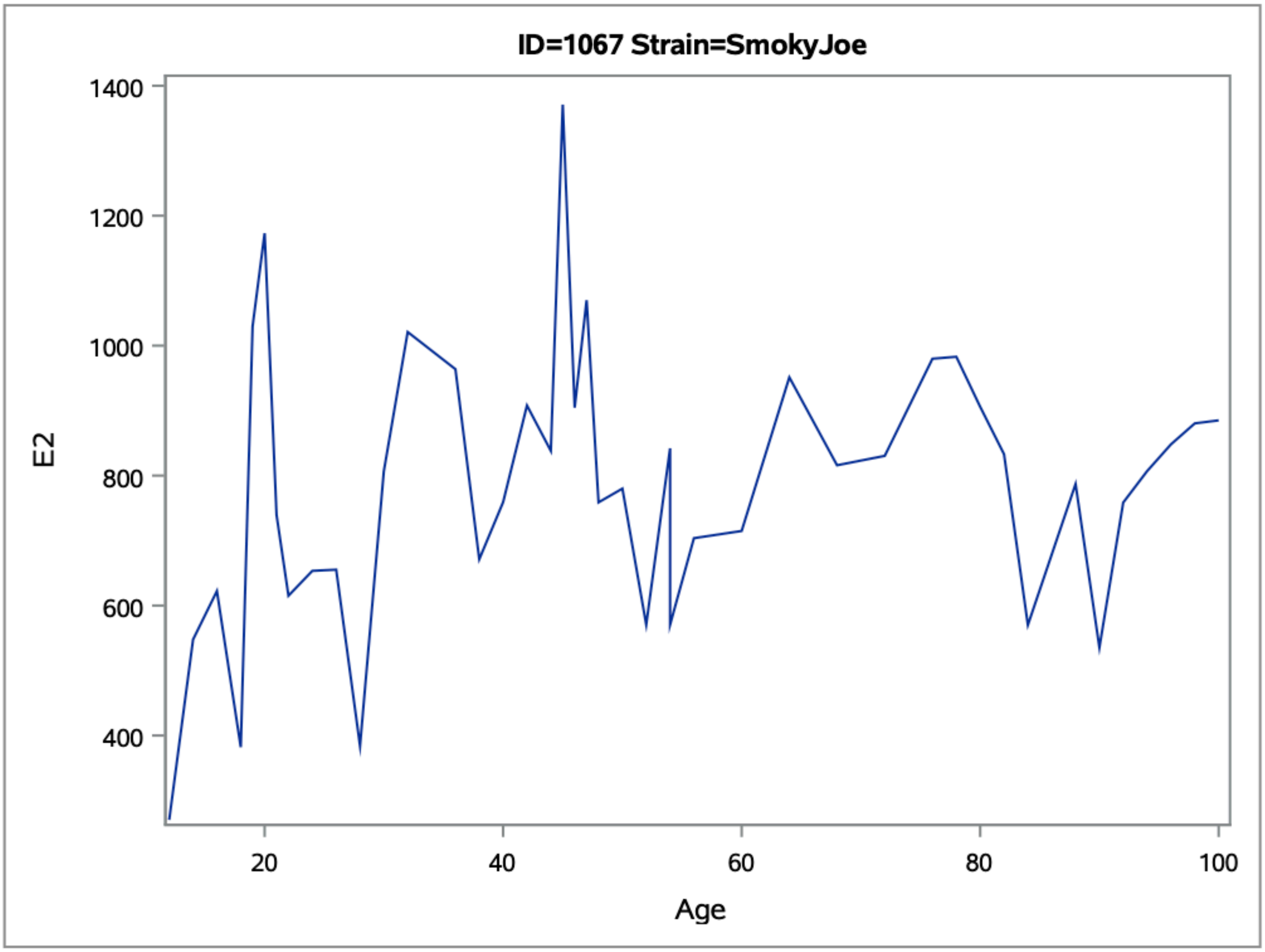

ID=1067 Strain=SmokyJoe
E2
1400
1200
1000
800
600
400
20
40
60
80
100
Age

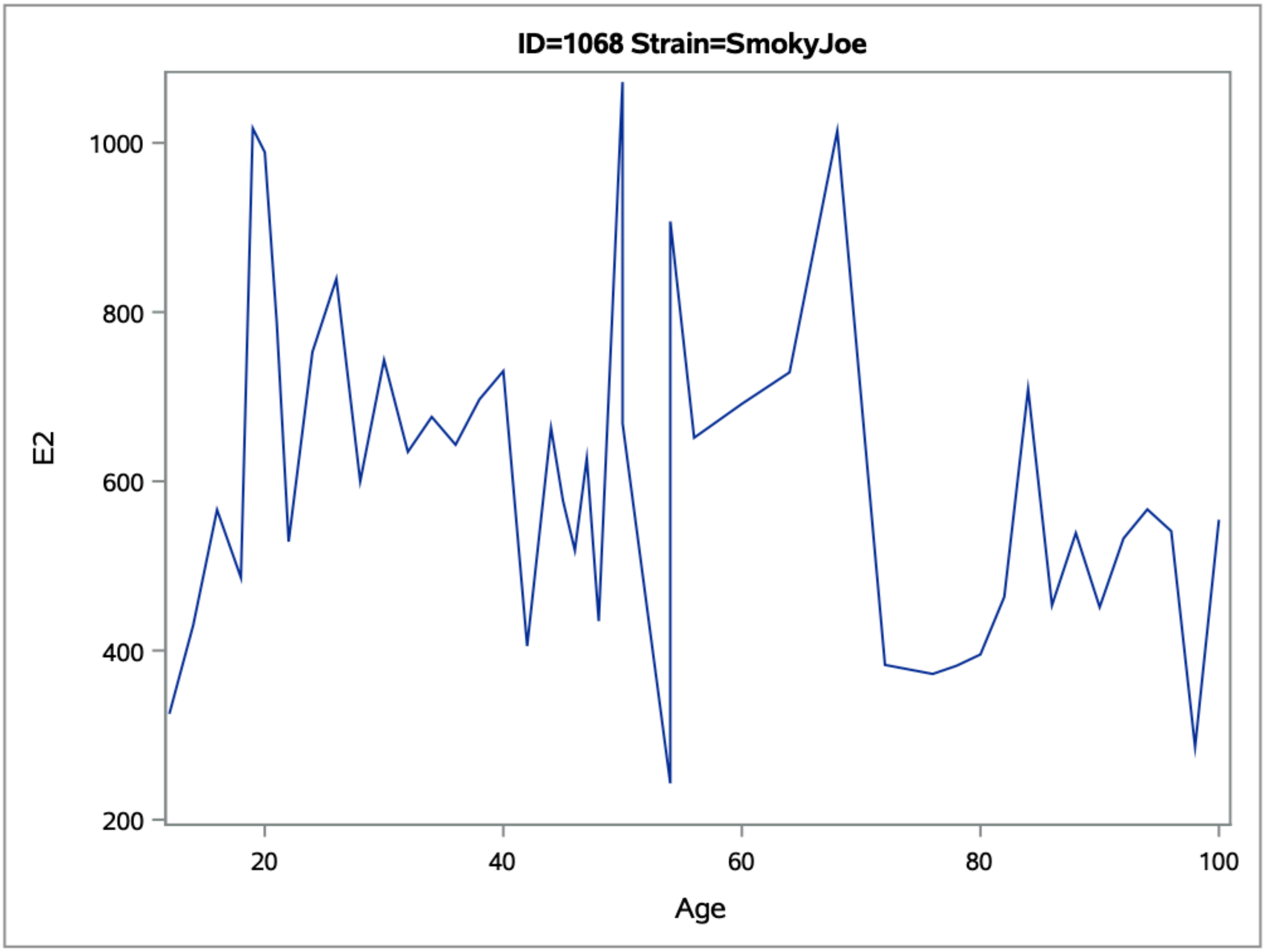
ID=1068 Strain=SmokyJoe
E2
1000
800
600
400
200
20
40
60
80
100
Age

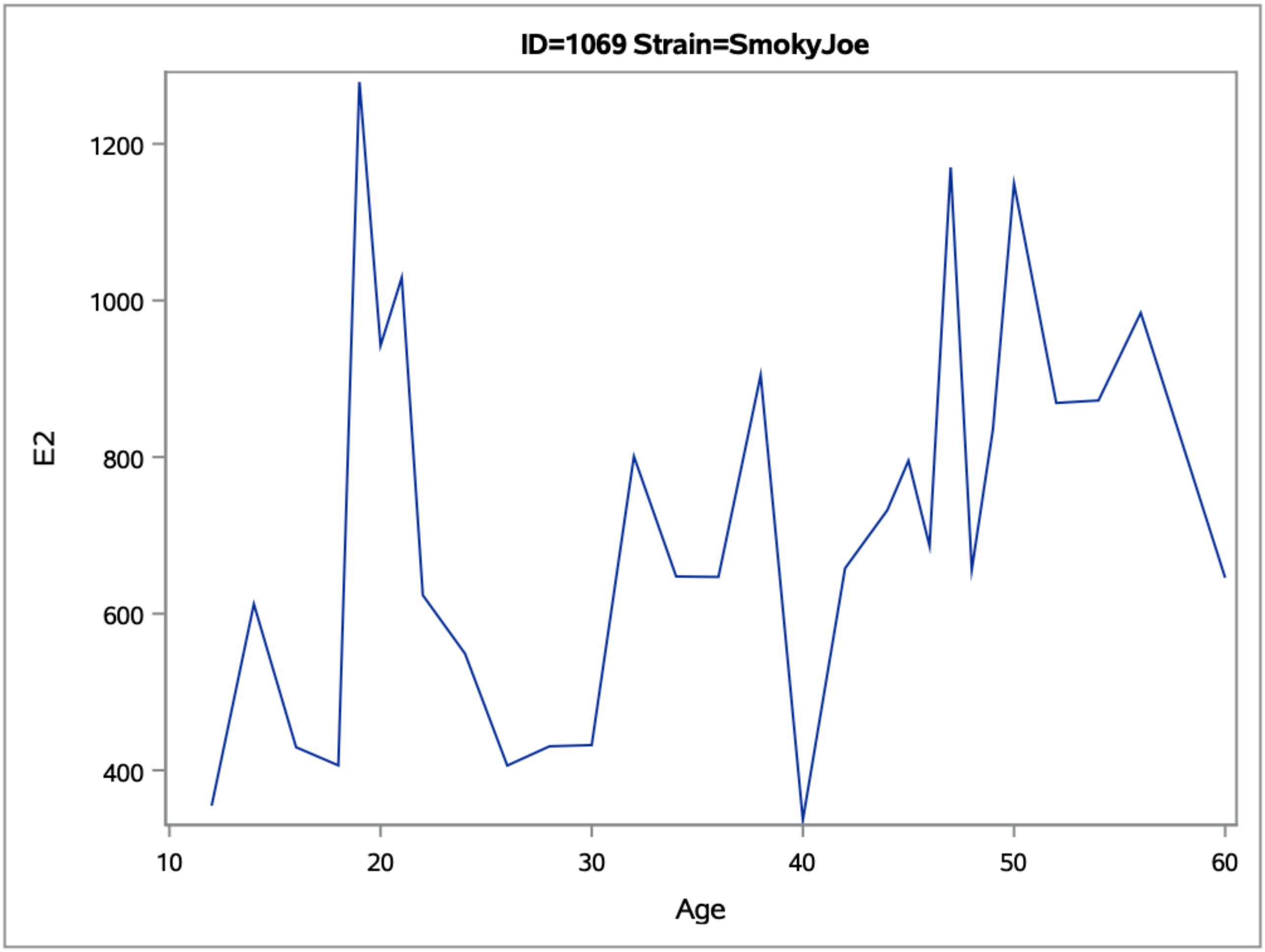
ID=1069 Strain=SmokyJoe
E2
1200
1000
800
600
400
10
20
30
40
50
60
Age

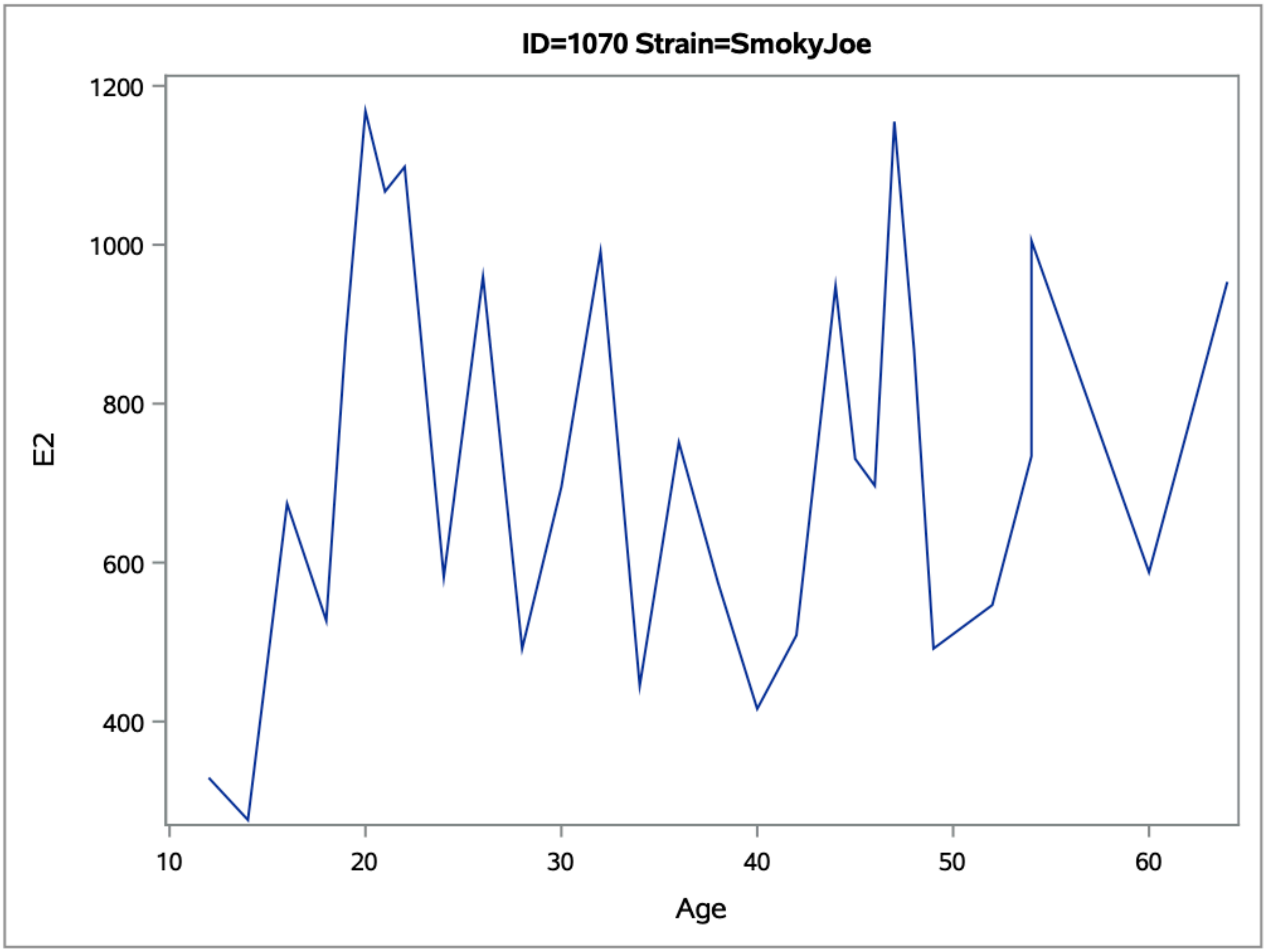
ID=1070 Strain=SmokyJoe
1200
1000
800
600
400
E2
10
20
30
40
50
60
Age

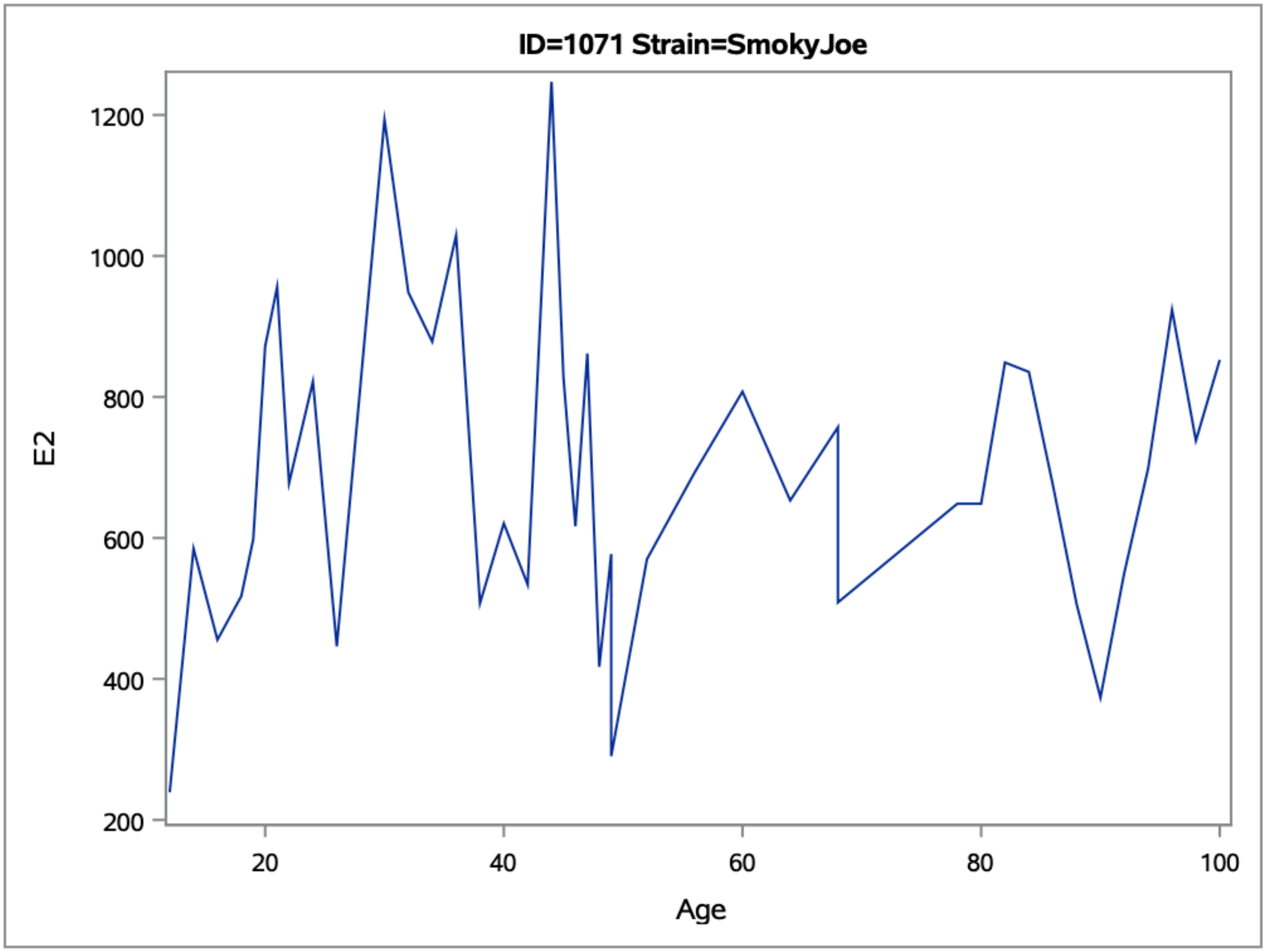
ID=1071 Strain=SmokyJoe
E2
1200
1000
800
600
400
200
20
40
60
80
100
Age

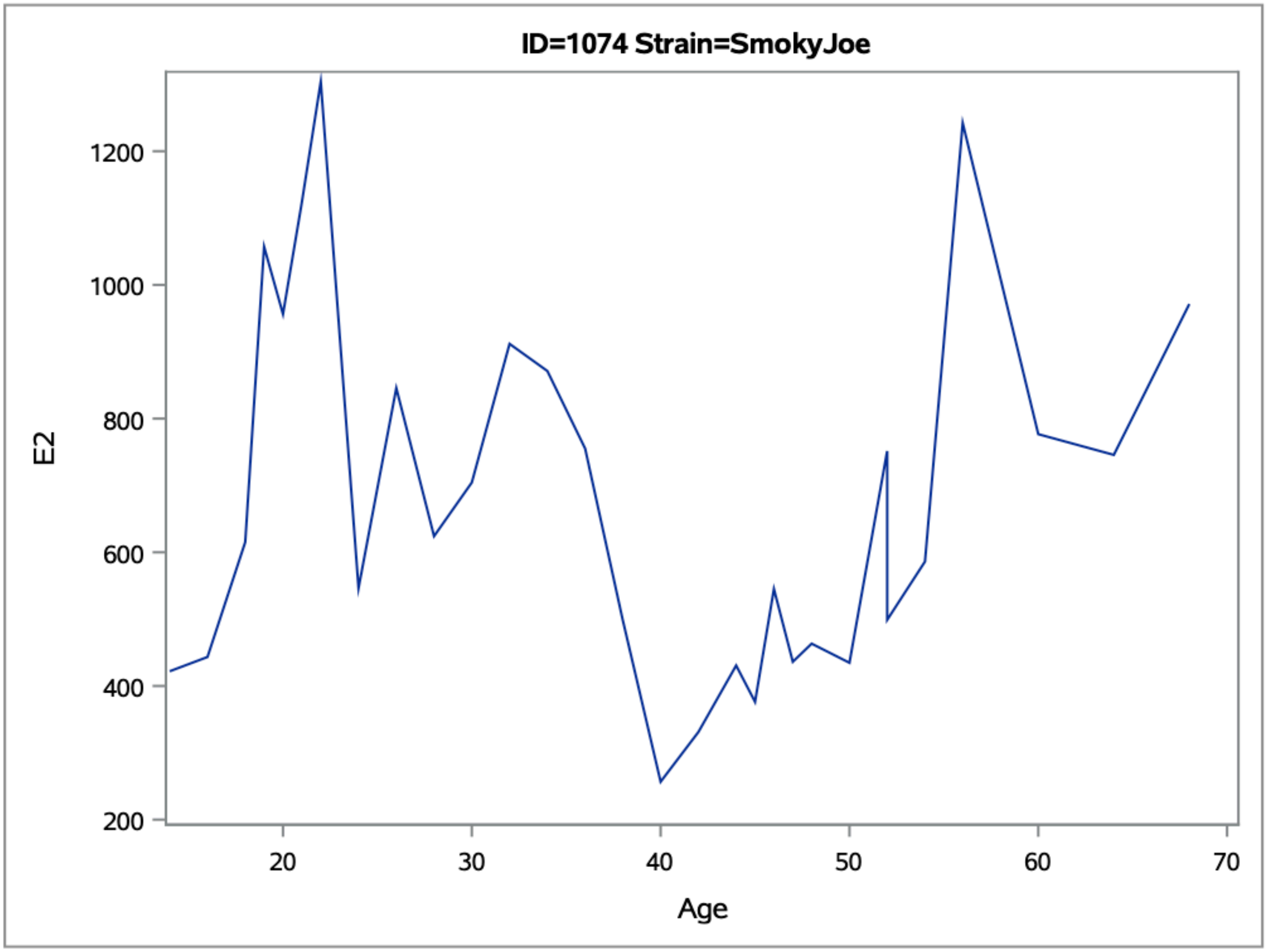

ID=1074 Strain=SmokyJoe
E2
1200
1000
800
600
400
200
20
30
40
50
60
70
Age

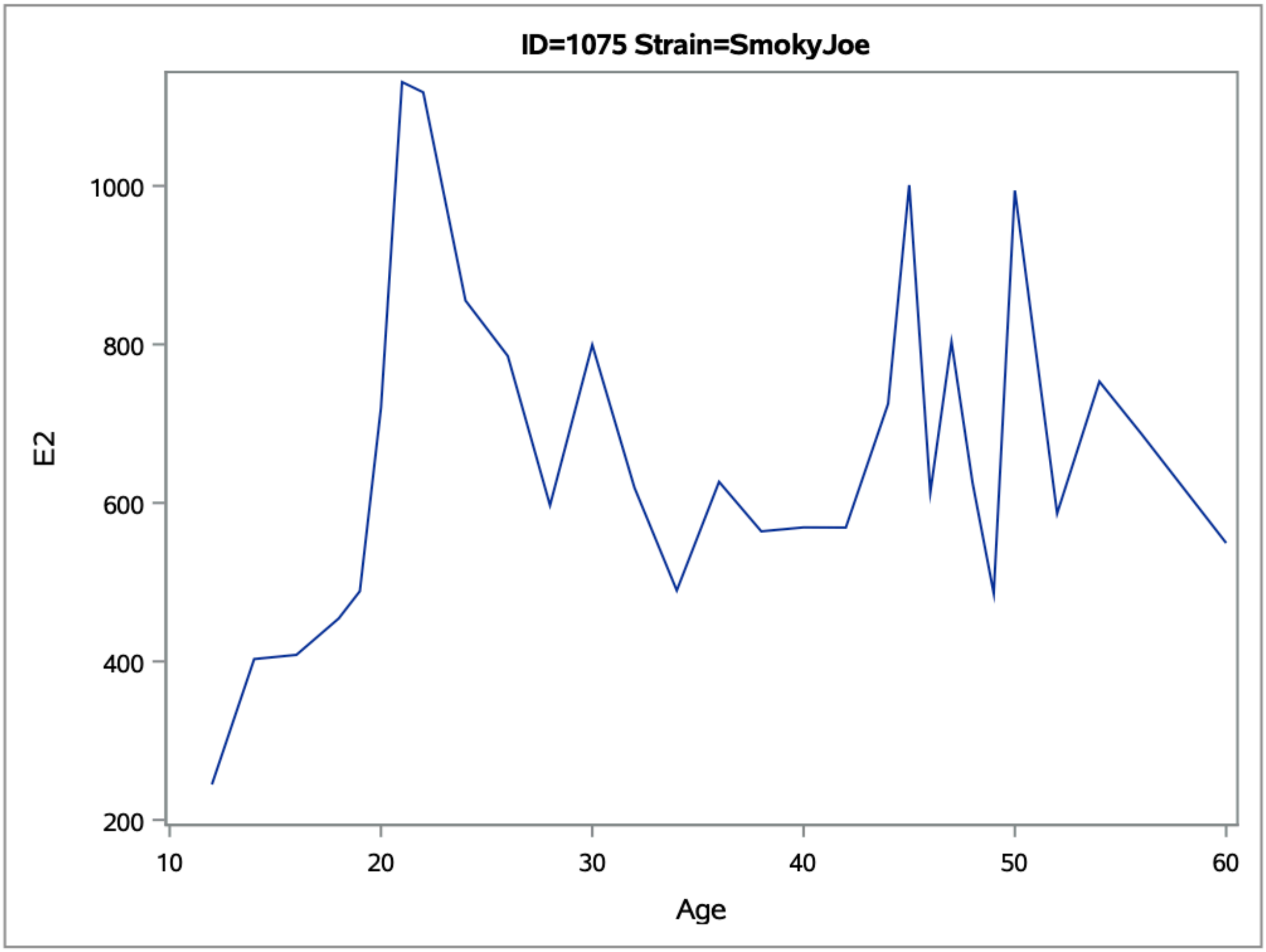
ID=1075 Strain=SmokyJoe
E2
1000
800
600
400
200
10
20
30
40
50
60
Age

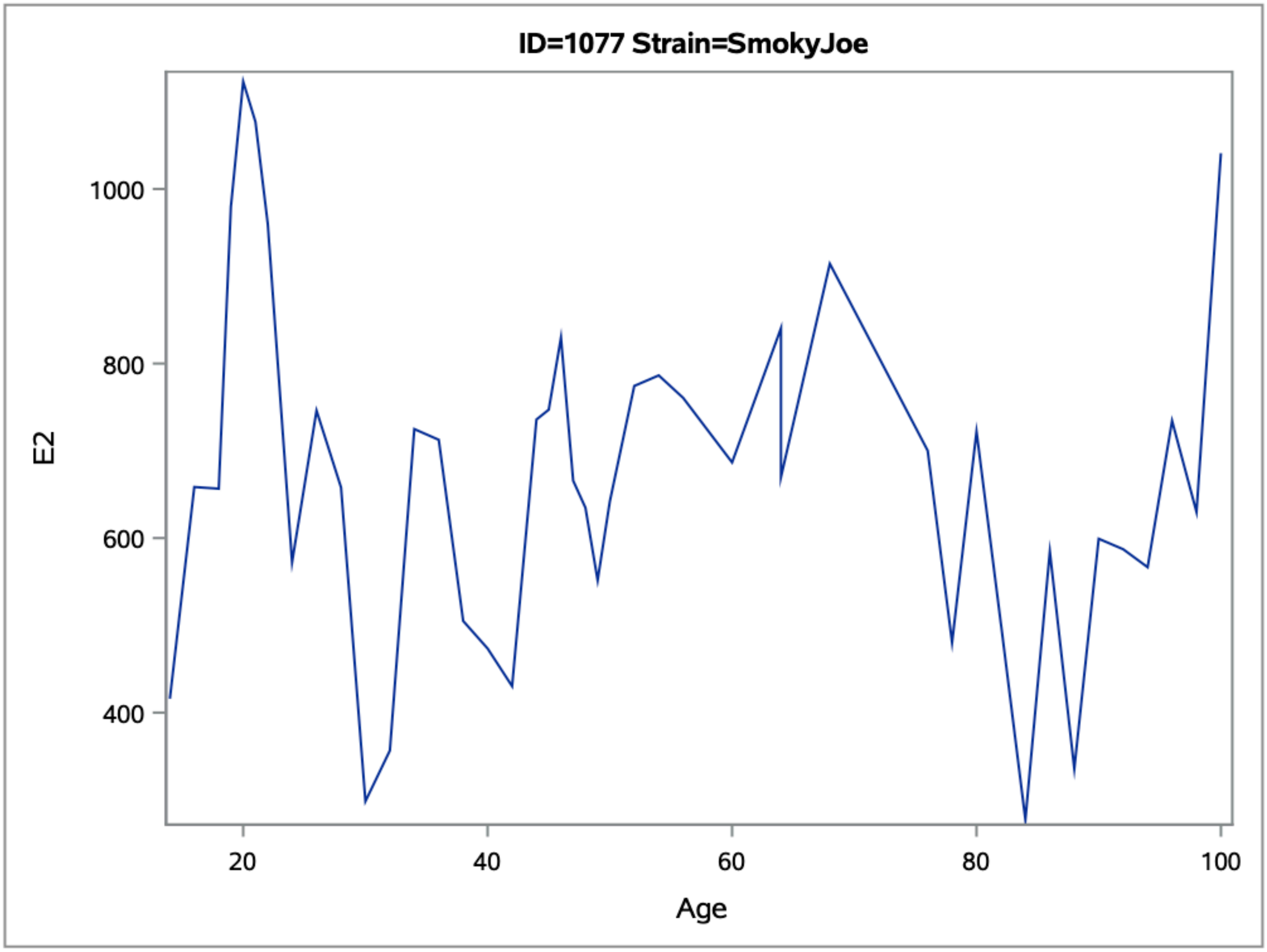
ID=1077 Strain=SmokyJoe
E2
1000
800
600
400
20
40
60
80
100
Age

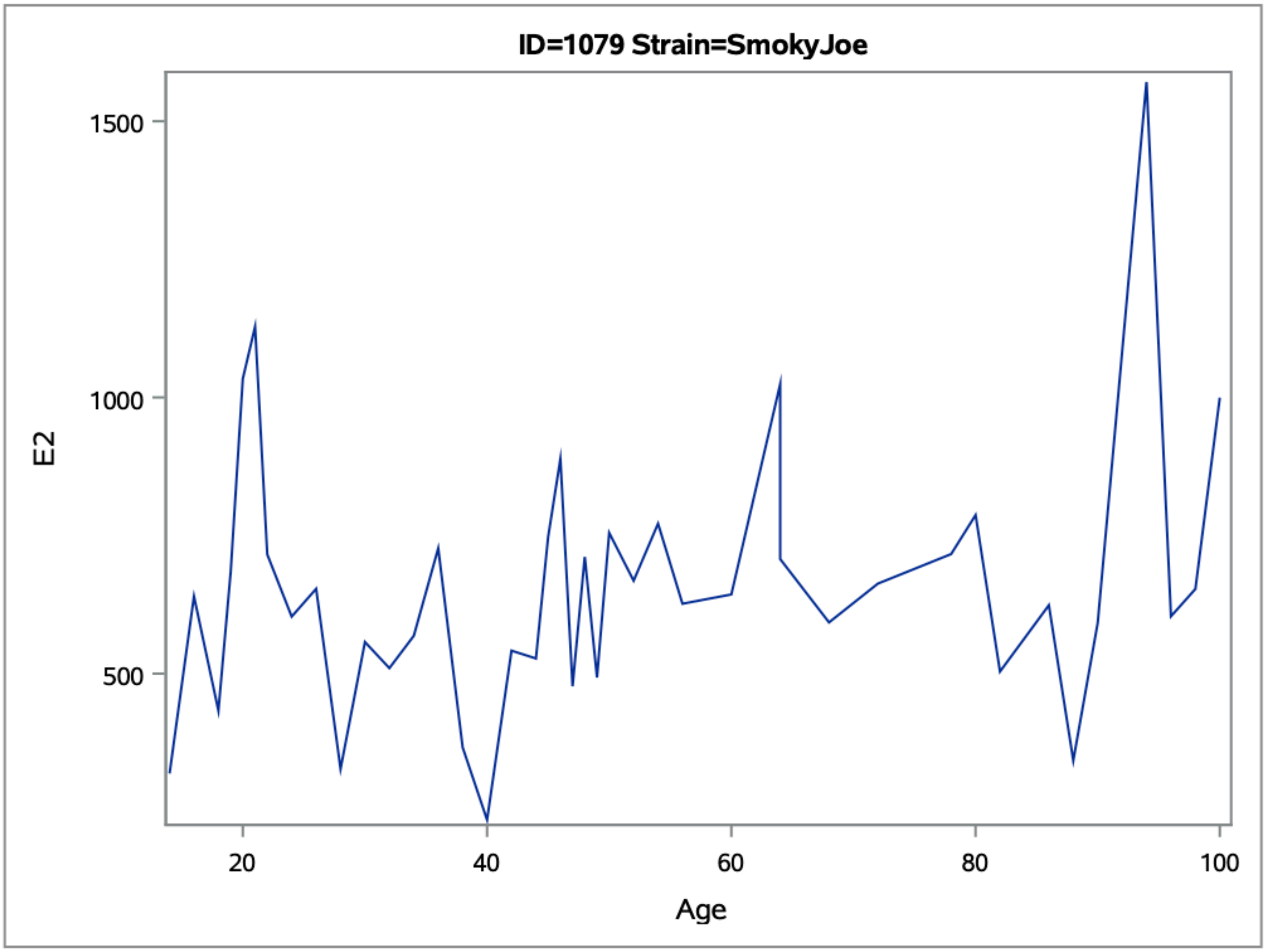
ID=1079 Strain=SmokyJoe
1500
1000
500
E2
20
40
60
80
100
Age

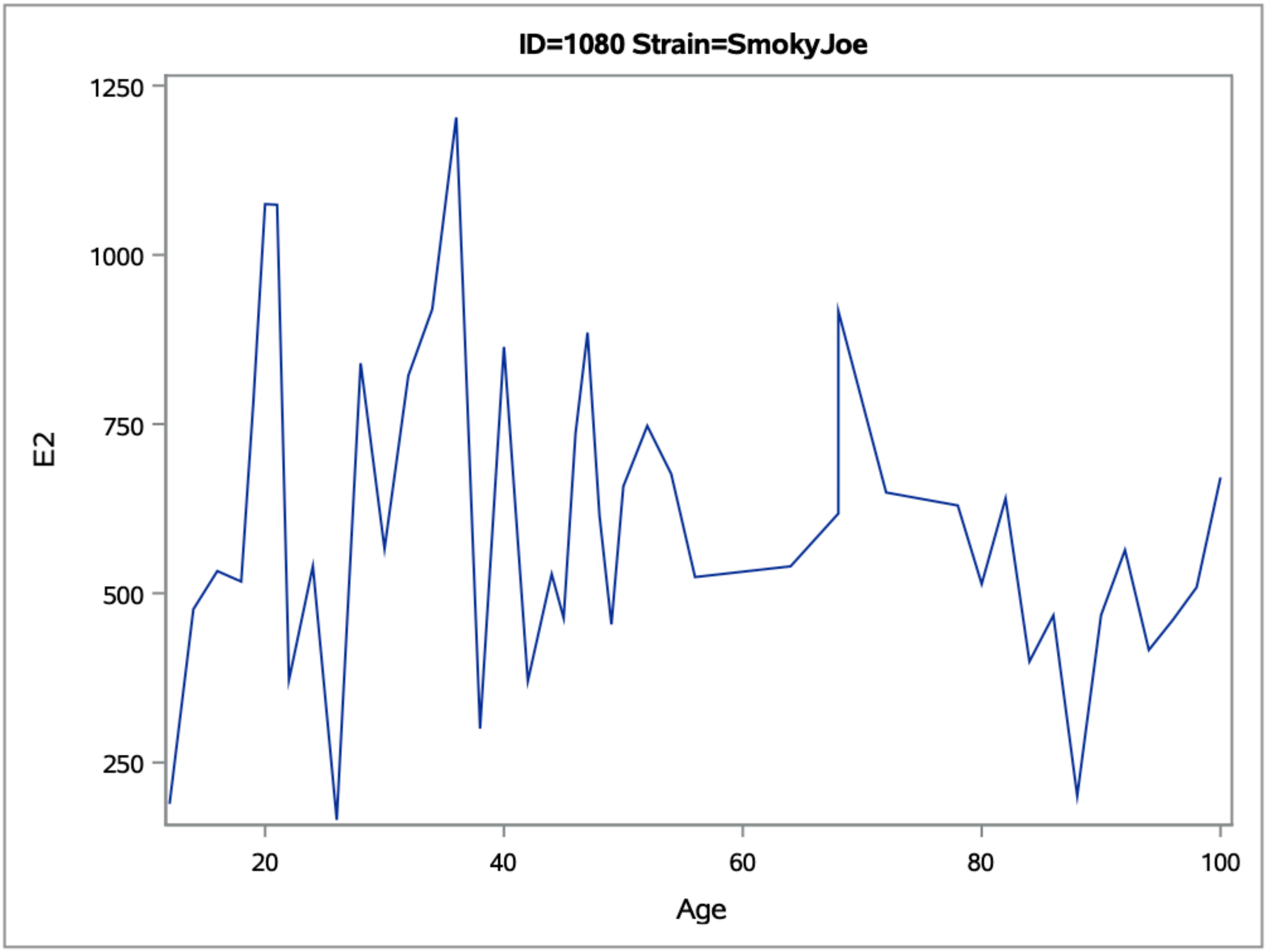
ID=1080 Strain=SmokyJoe
1250
1000
750
500
250
E2
20
40
60
80
100
Age

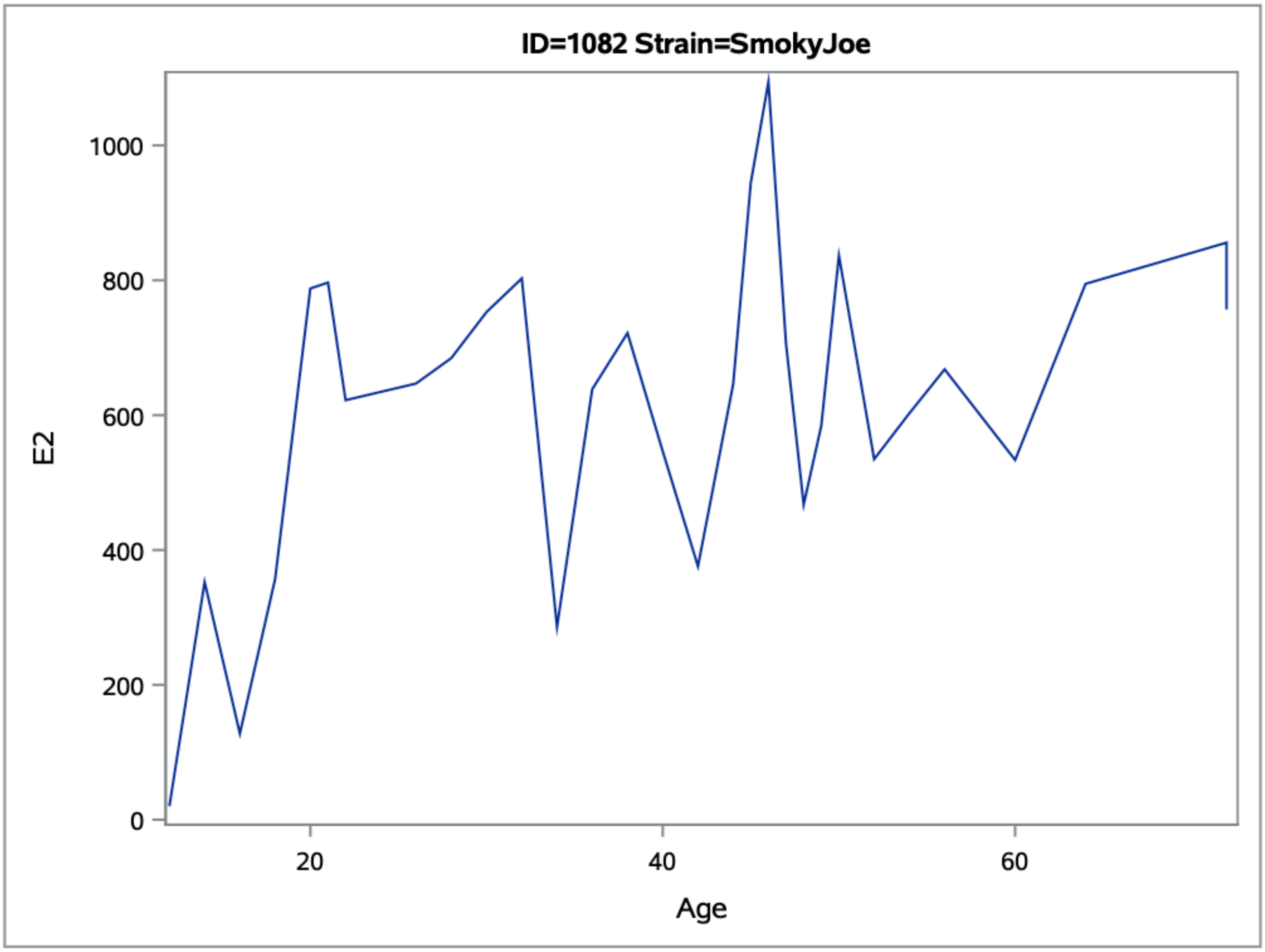

ID=1082 Strain=SmokyJoe
E2
0
200
400
600
800
1000
20
40
60
Age

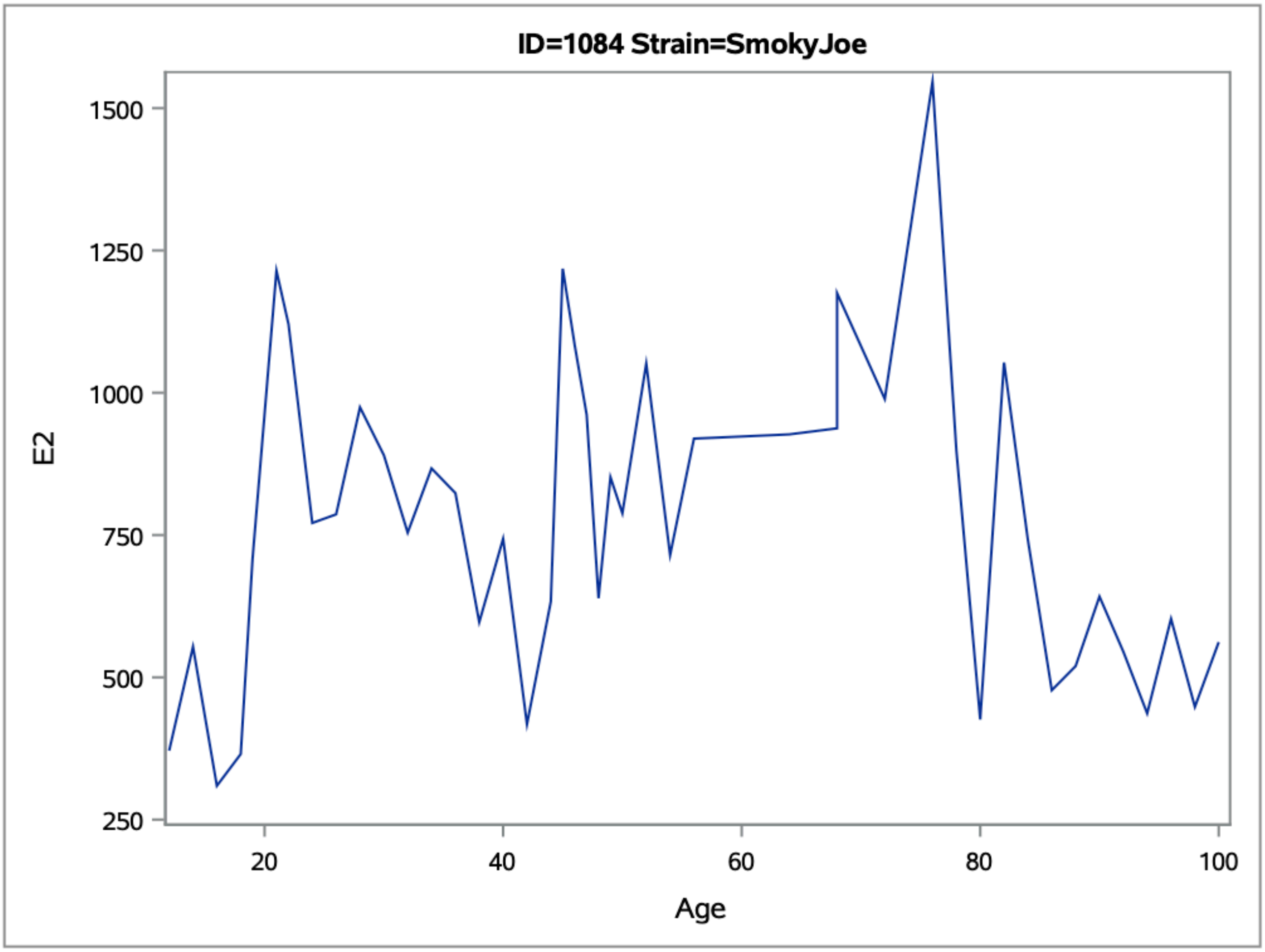
ID=1084 Strain=SmokyJoe
E2
1500
1250
1000
750
500
250
20
40
60
80
100
Age

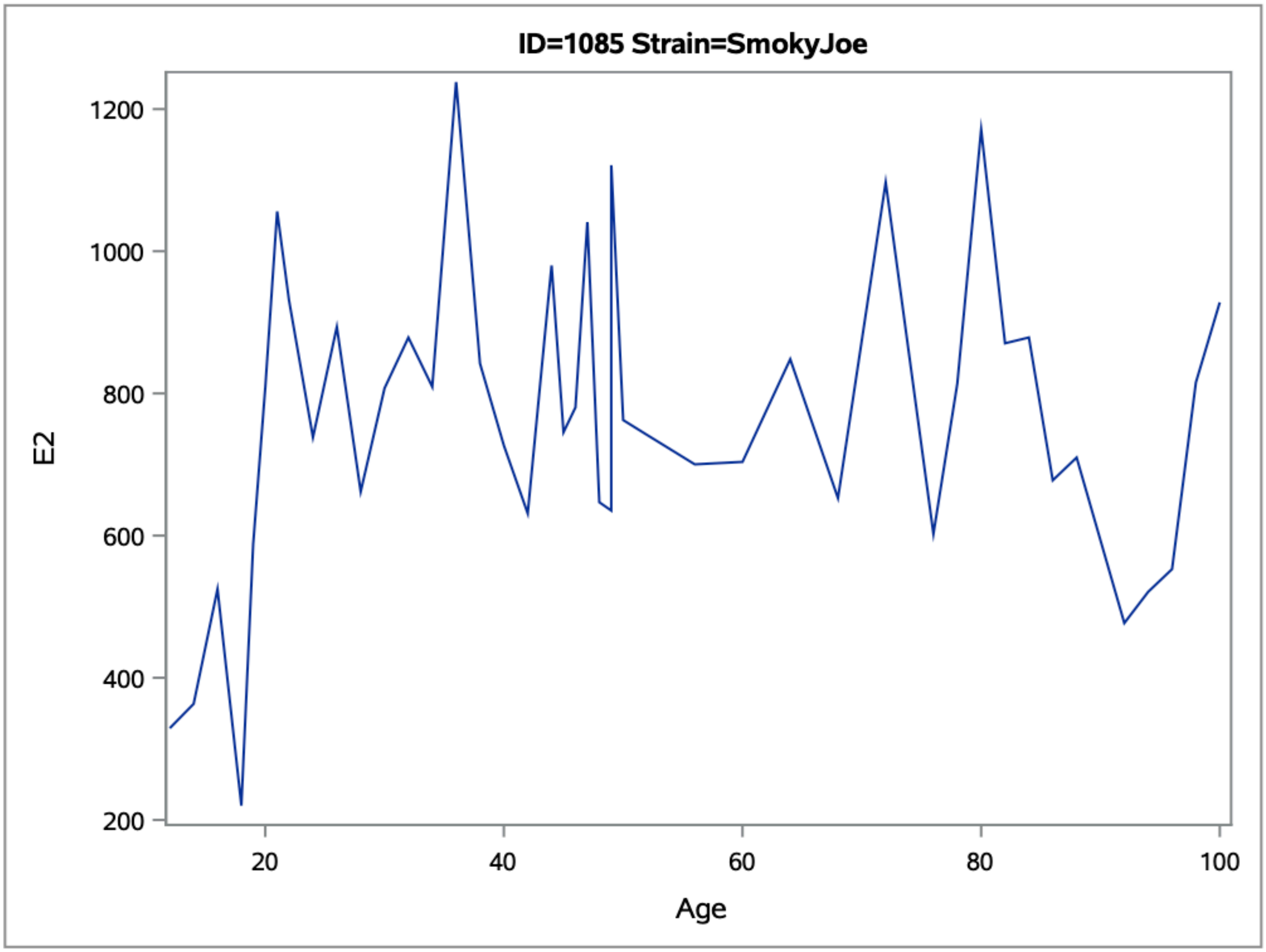
ID=1085 Strain=SmokyJoe
1200
1000
800
600
400
200
E2
20
40
60
80
100
Age

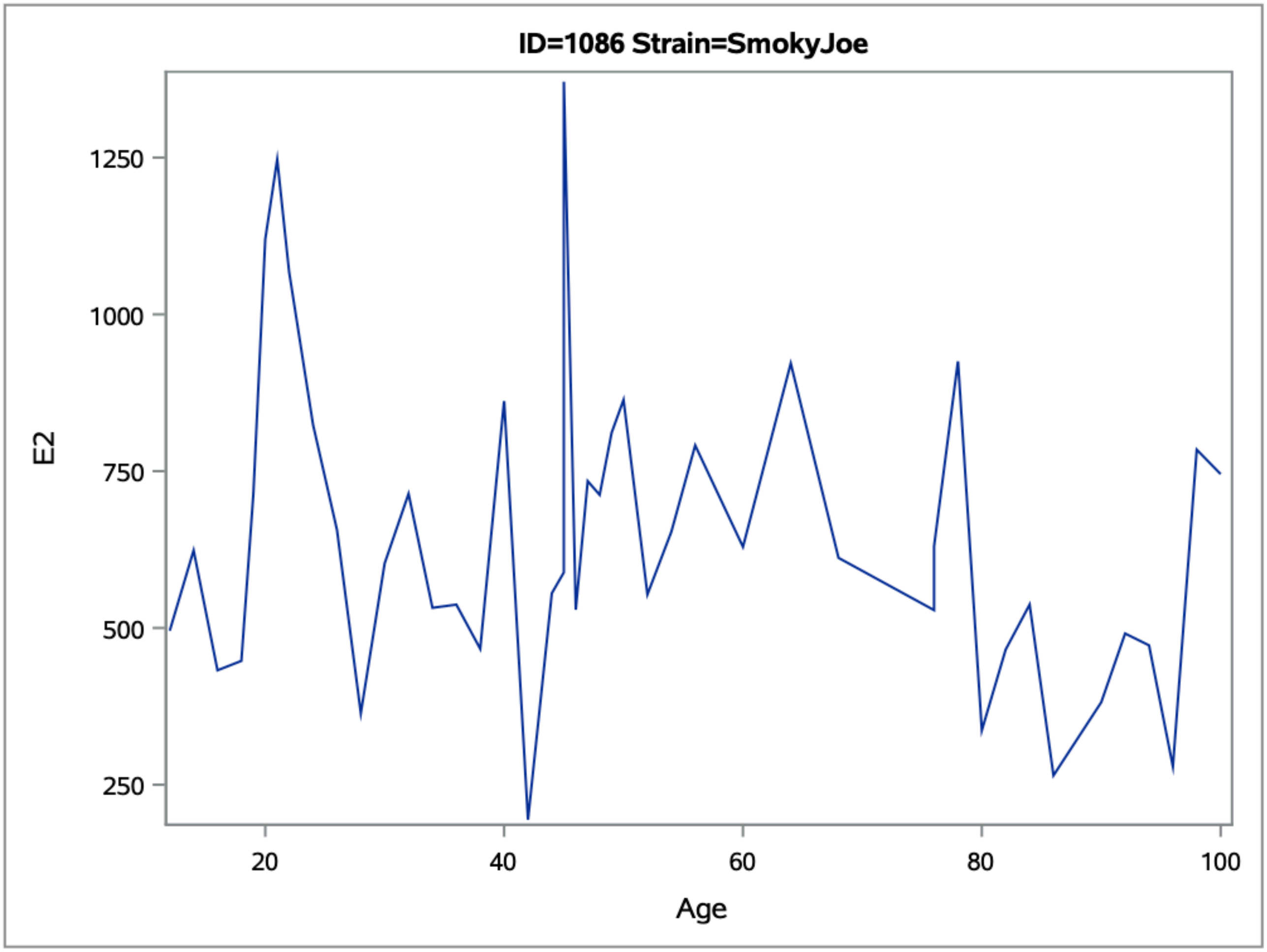
ID=1086 Strain=SmokyJoe
E2
1250
1000
750
500
250
20
40
60
80
100
Age

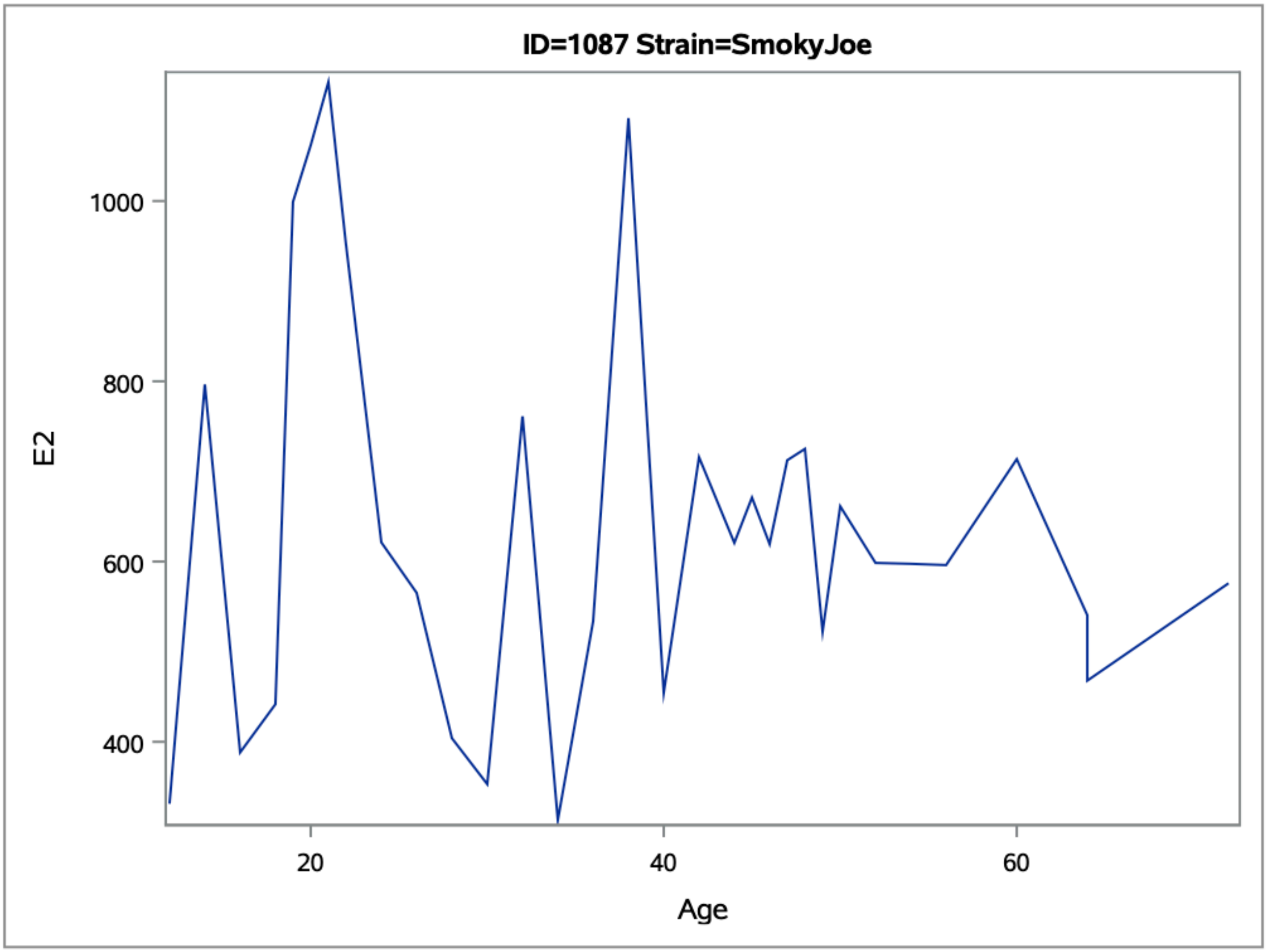
ID=1087 Strain=SmokyJoe
1000
800
600
400
E2
20
40
60
Age

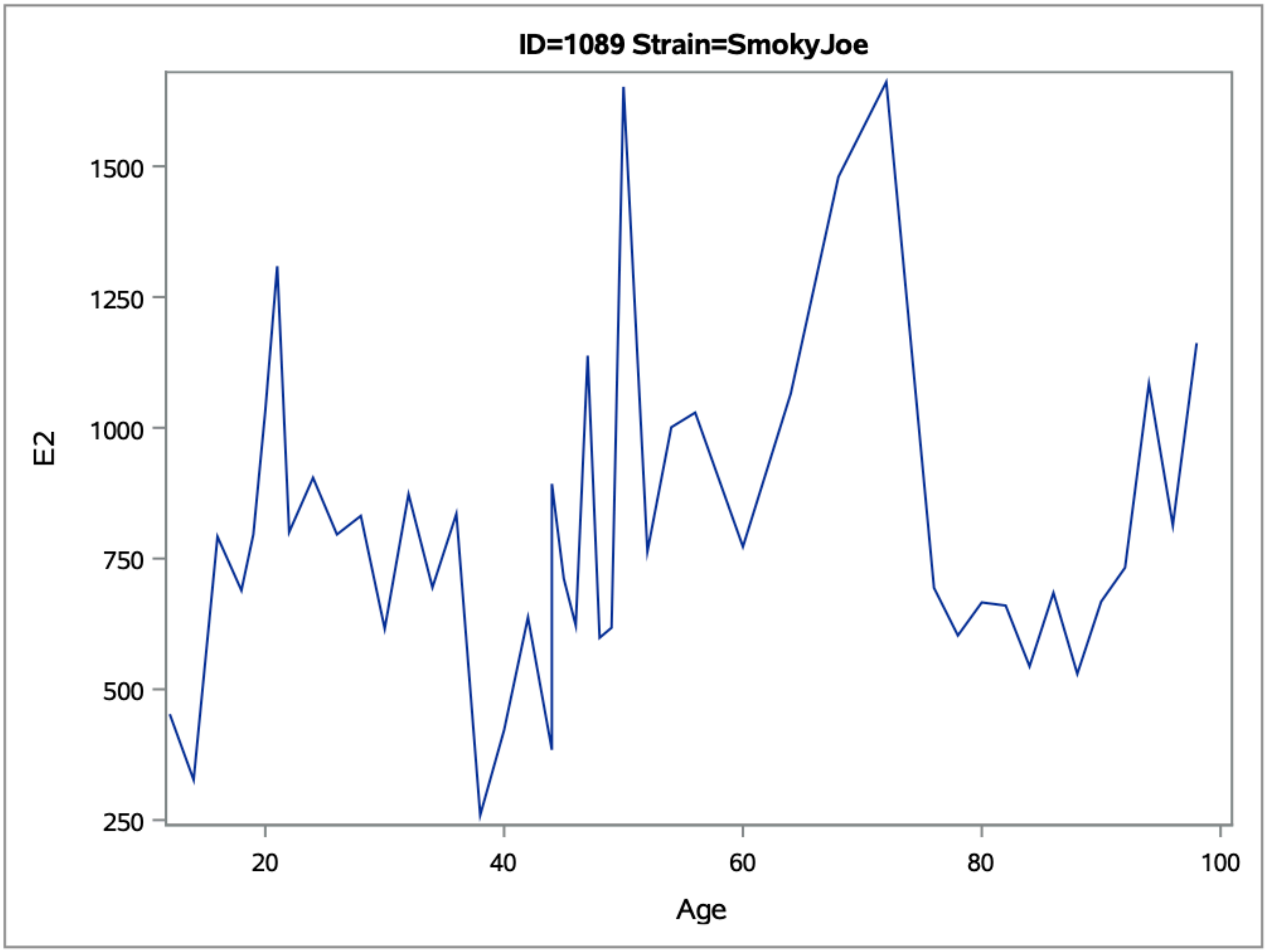
ID=1089 Strain=SmokyJoe
E2
1500
1250
1000
750
500
250
20
40
60
80
100
Age

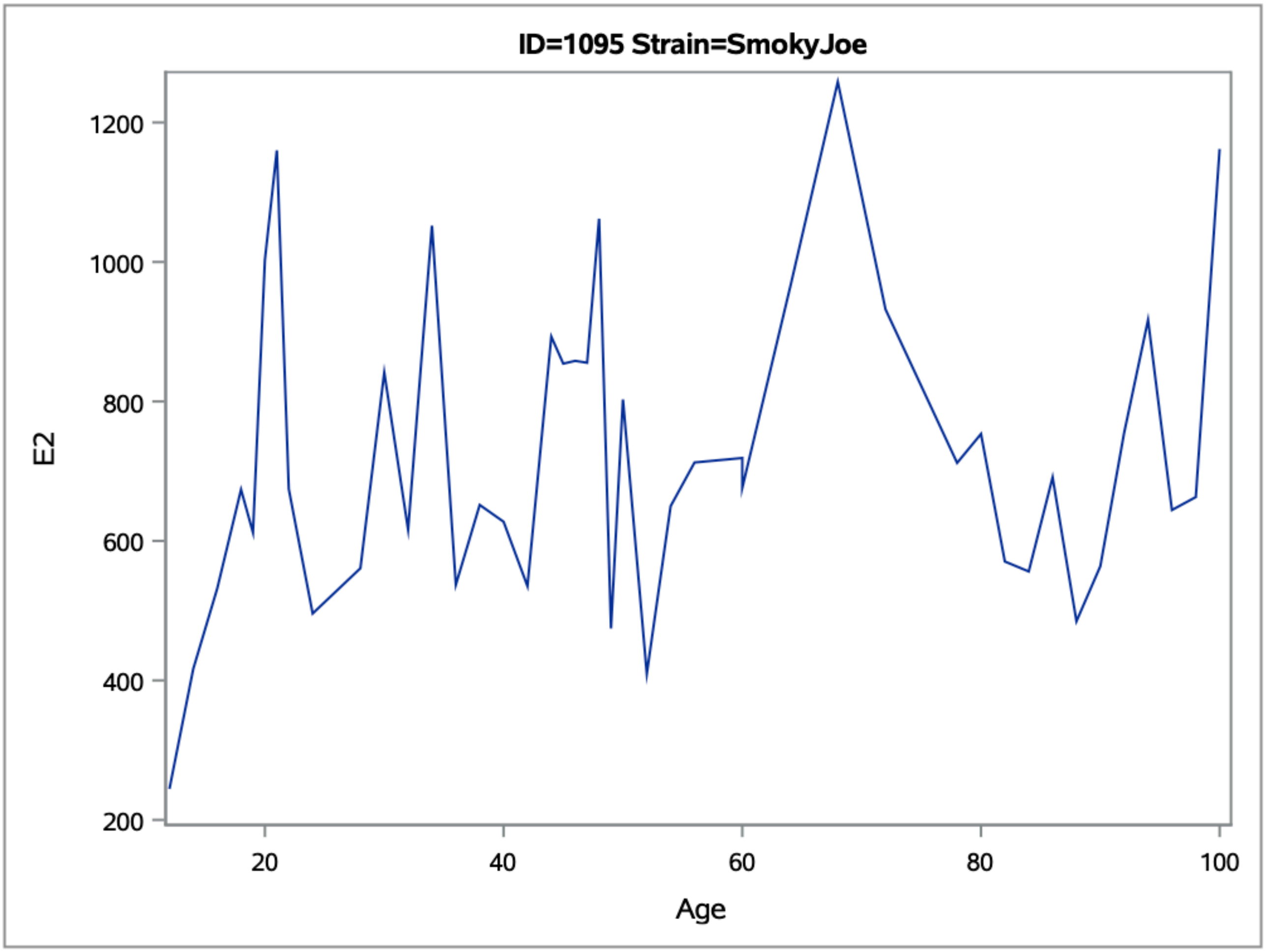
ID=1095 Strain=SmokyJoe
1200
1000
800
600
400
200
E2
20
40
60
80
100
Age

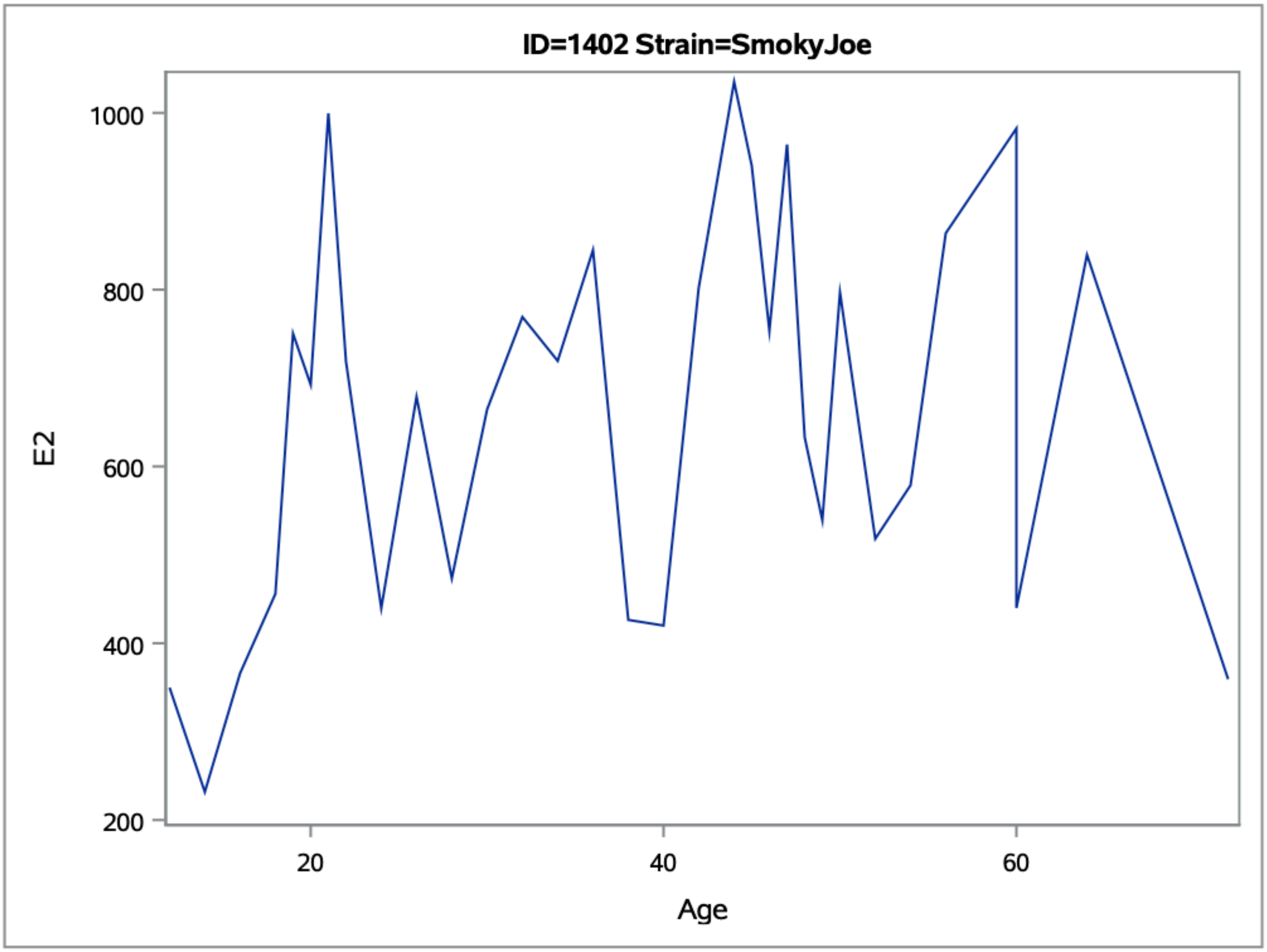
ID=1402 Strain=SmokyJoe
E2
1000
800
600
400
200
20
40
60
Age

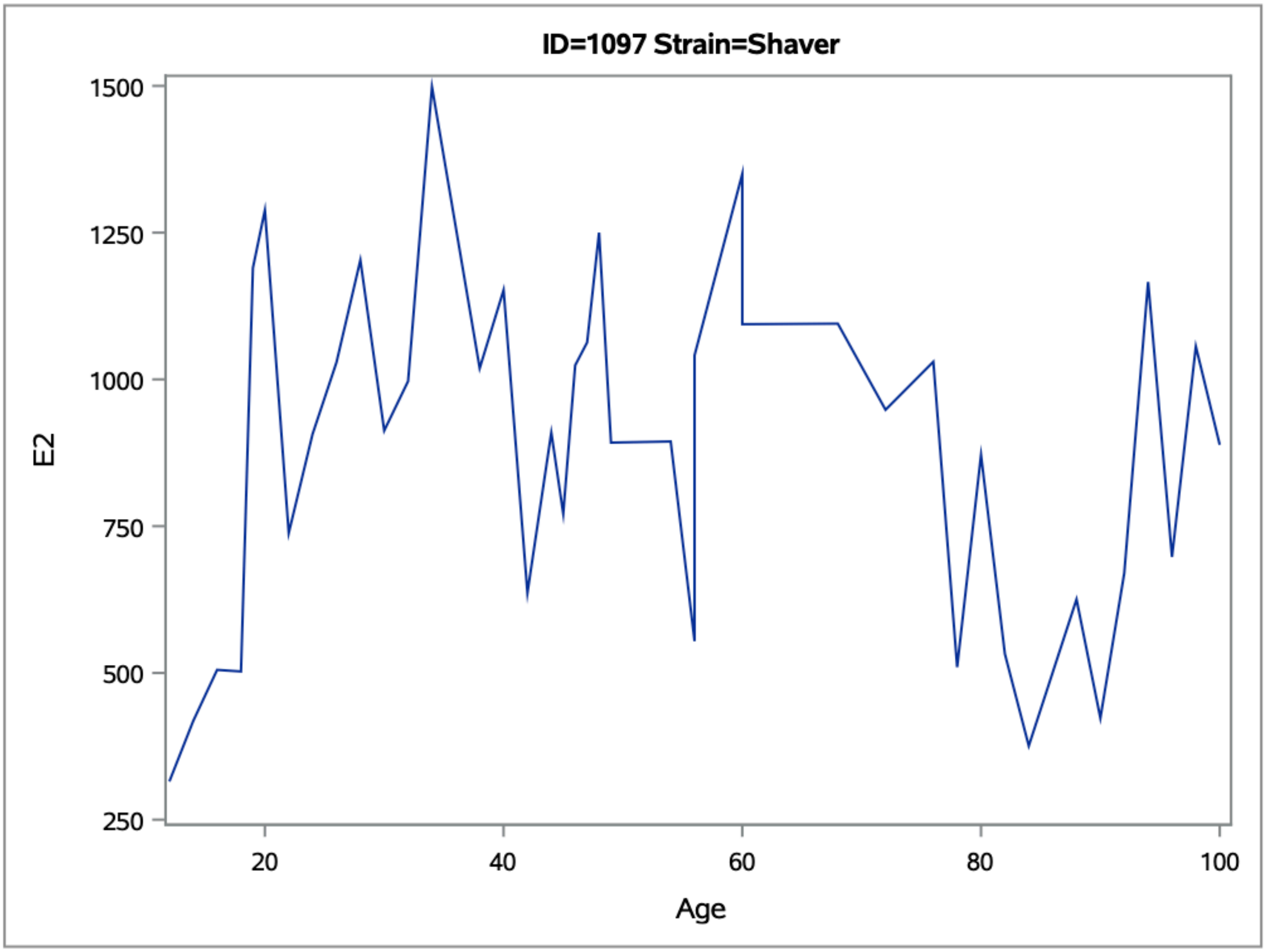
ID=1097 Strain=Shaver
1500
1250
1000
750
500
250
E2
20
40
60
80
100
Age

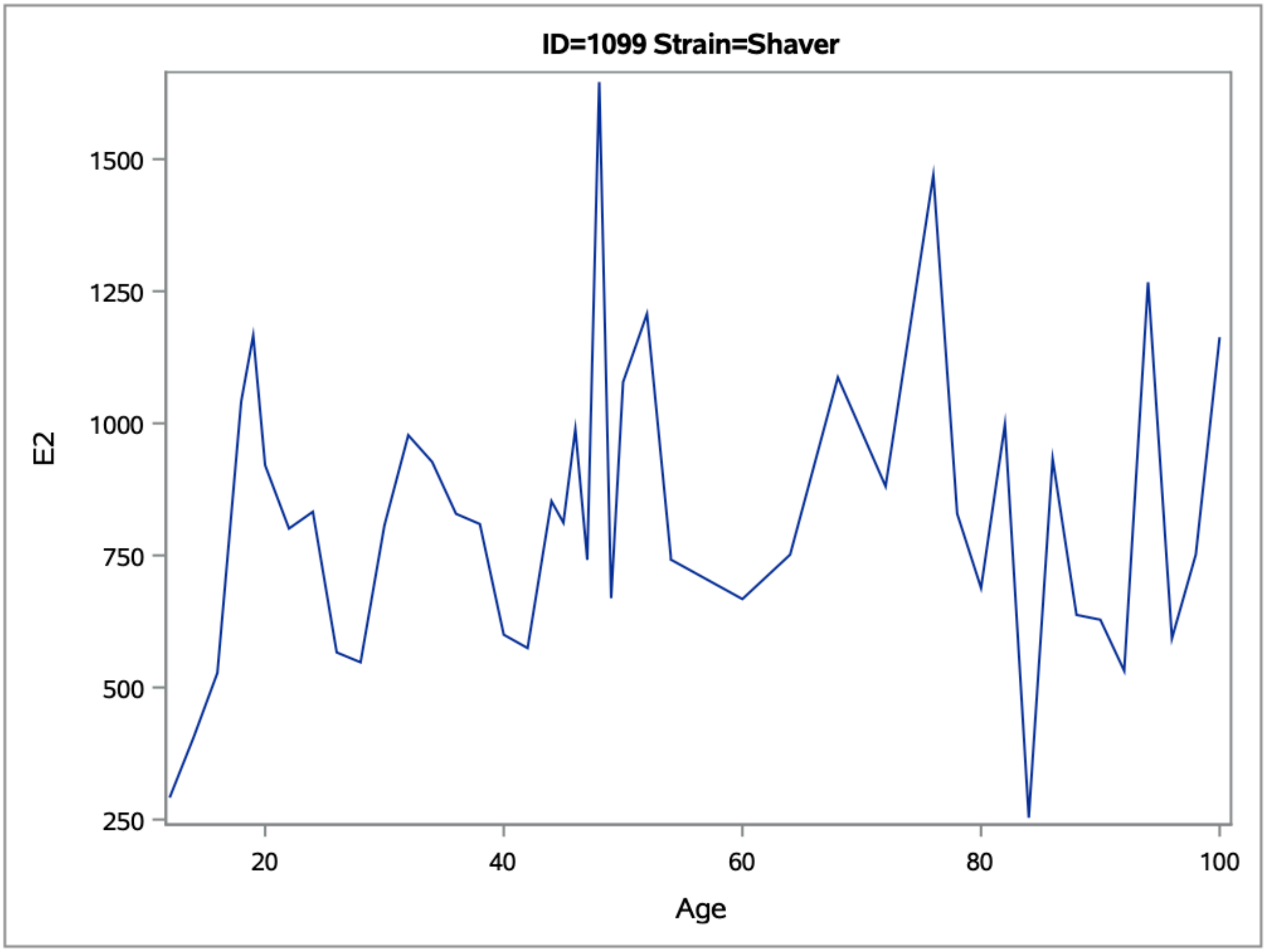
ID=1099 Strain=Shaver
E2
1500
1250
1000
750
500
250
20
40
60
80
100
Age

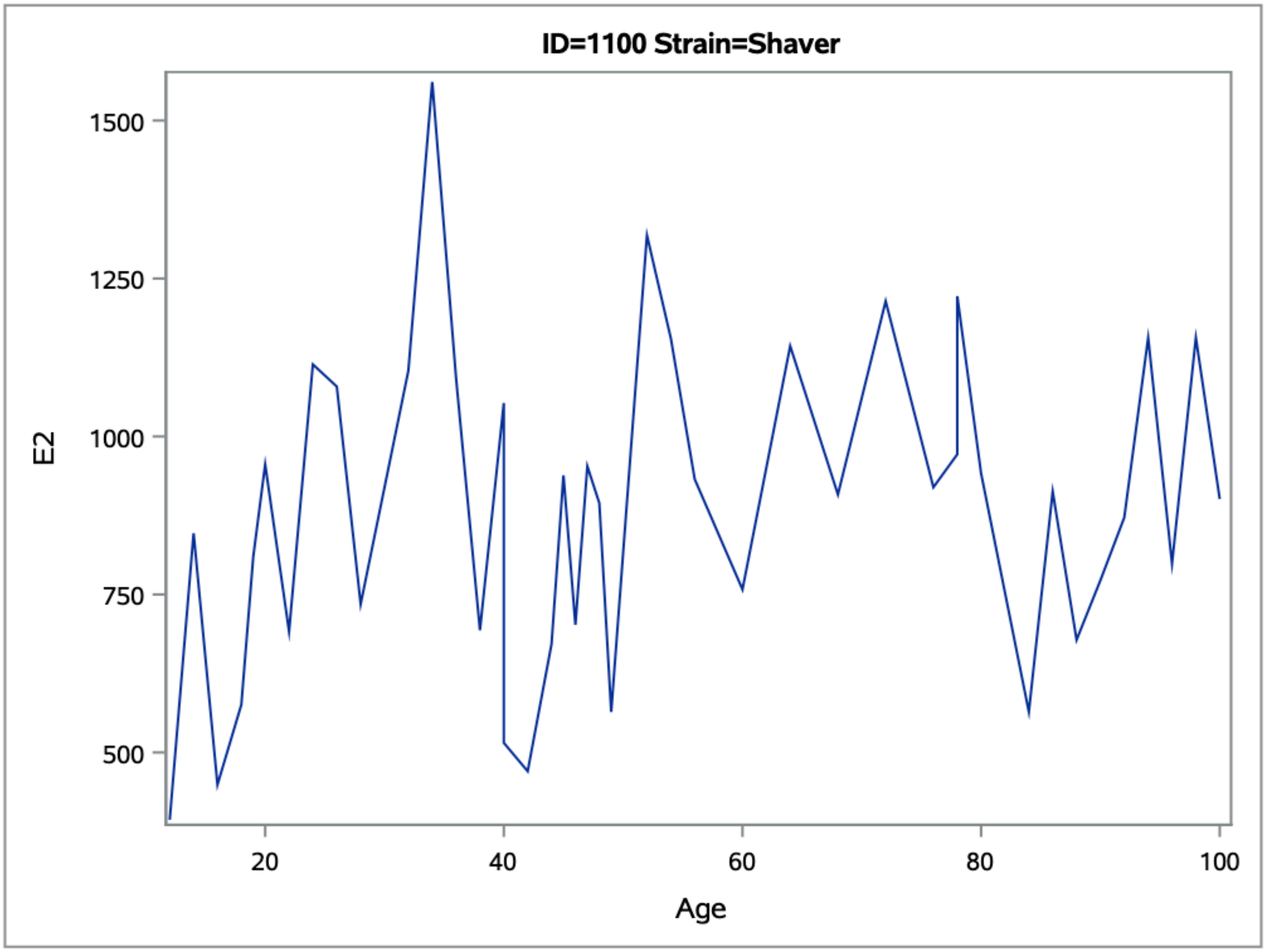
ID=1100 Strain=Shaver
E2
1500
1250
1000
750
500
20
40
60
80
100
Age

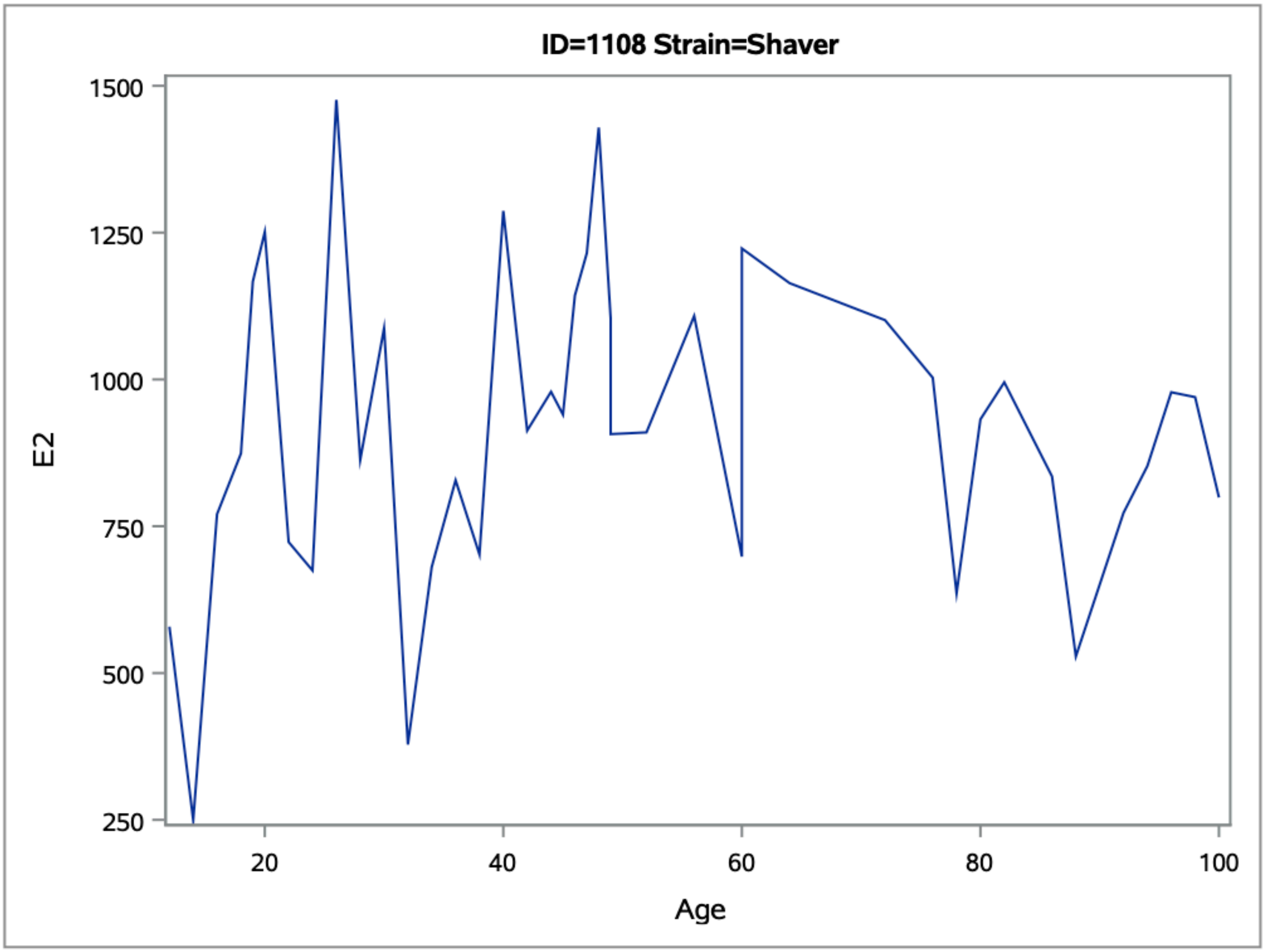
ID=1108 Strain=Shaver
1500
1250
1000
750
500
250
E2
20
40
60
80
100
Age

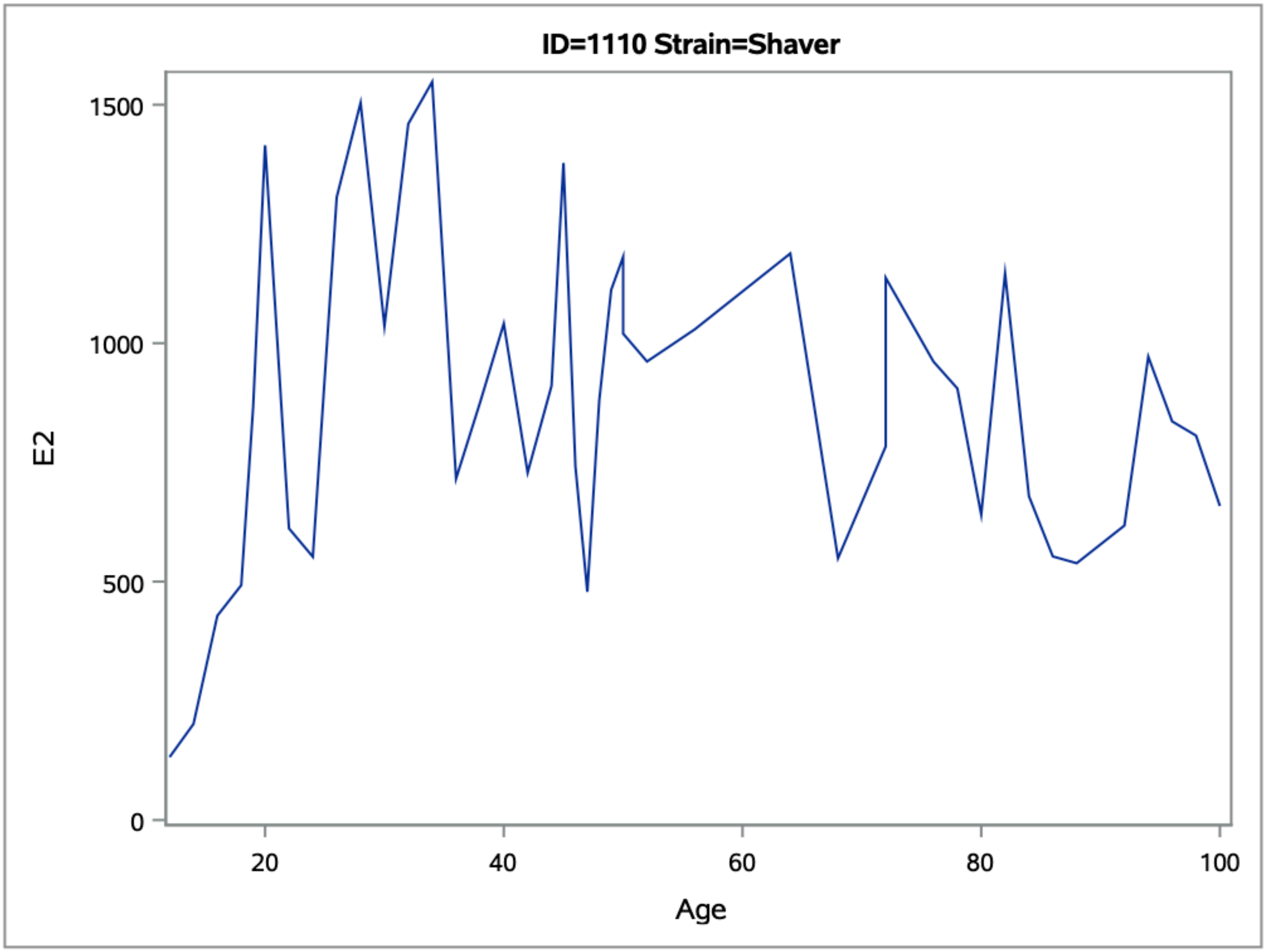
ID=1110 Strain=Shaver
1500
1000
500
0
E2
20
40
60
80
100
Age

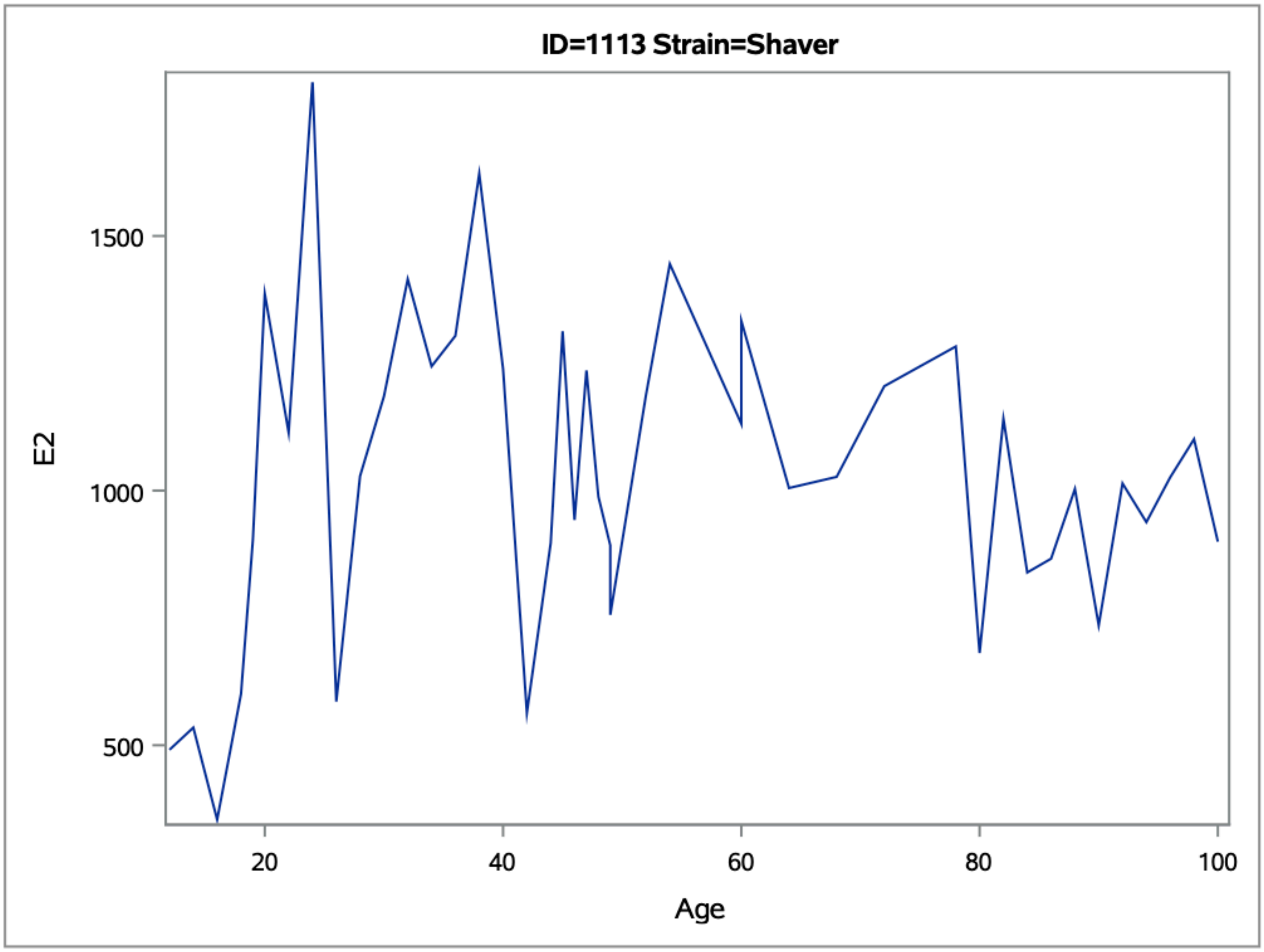
ID=1113 Strain=Shaver
E2
1500
1000
500
20
40
60
80
100
Age

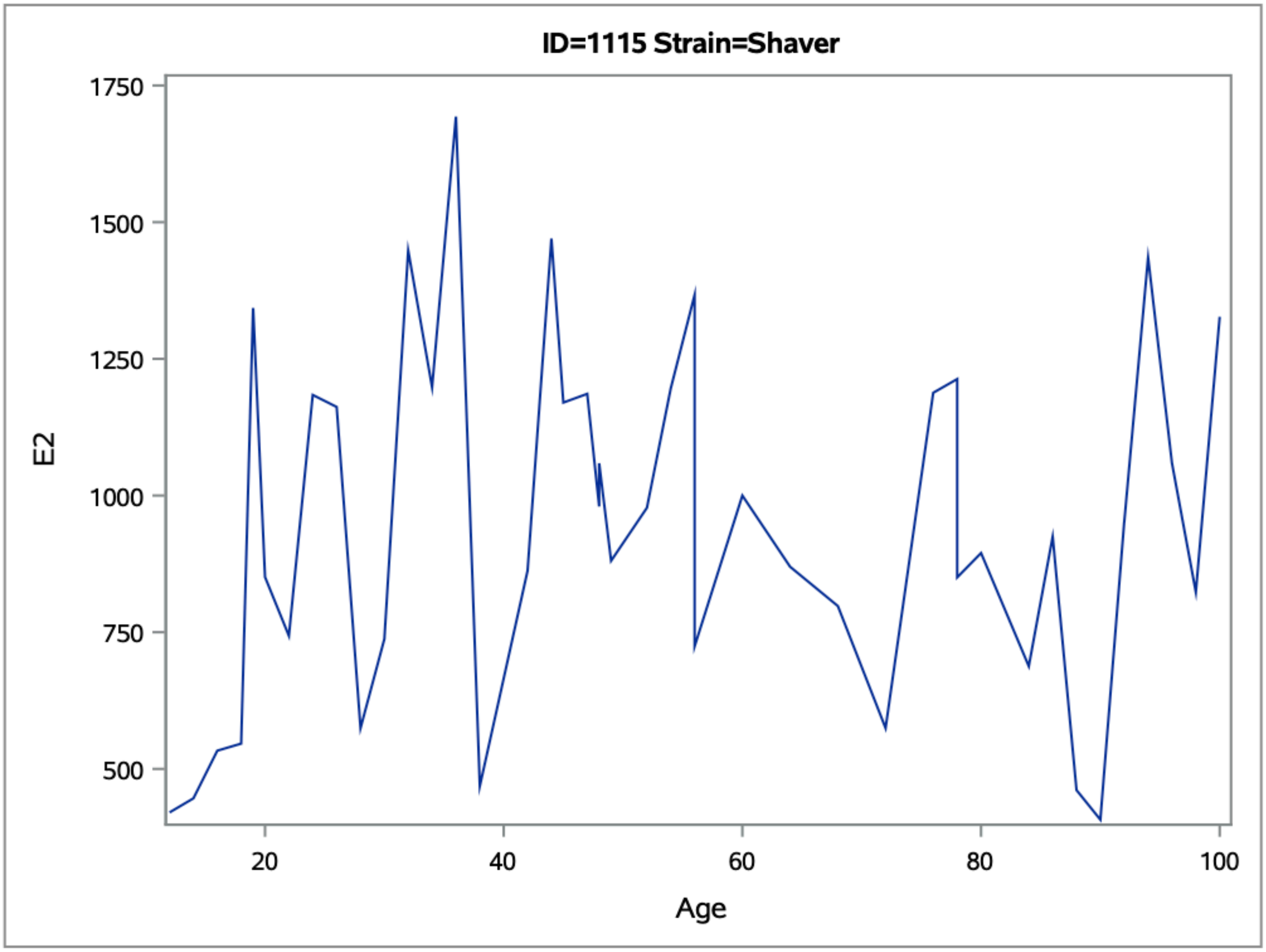
ID=1115 Strain=Shaver
1750
1500
1250
1000
750
500
E2
20
40
60
80
100
Age

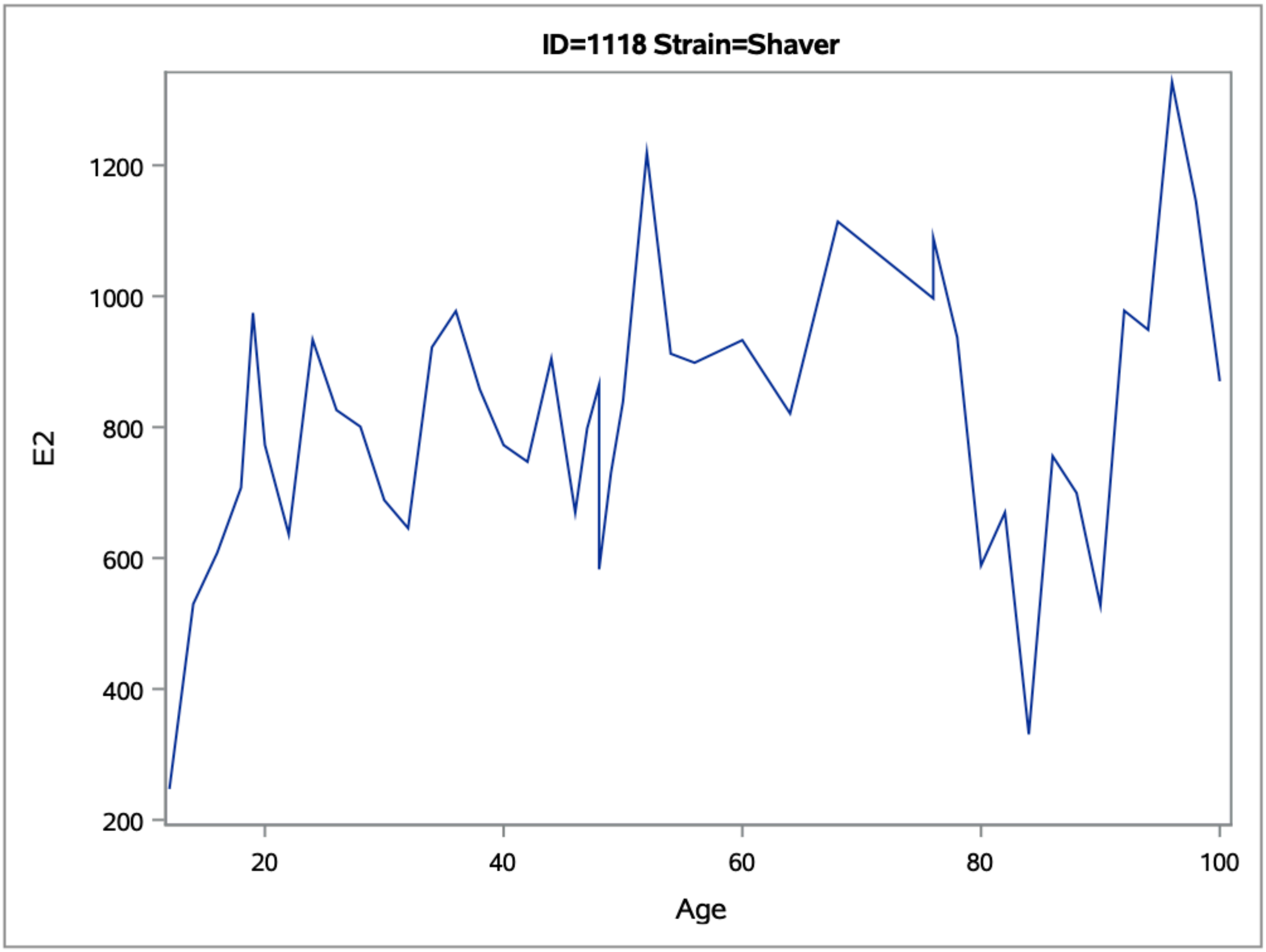
ID=1118 Strain=Shaver
E2
1200
1000
800
600
400
200
20
40
60
80
100
Age

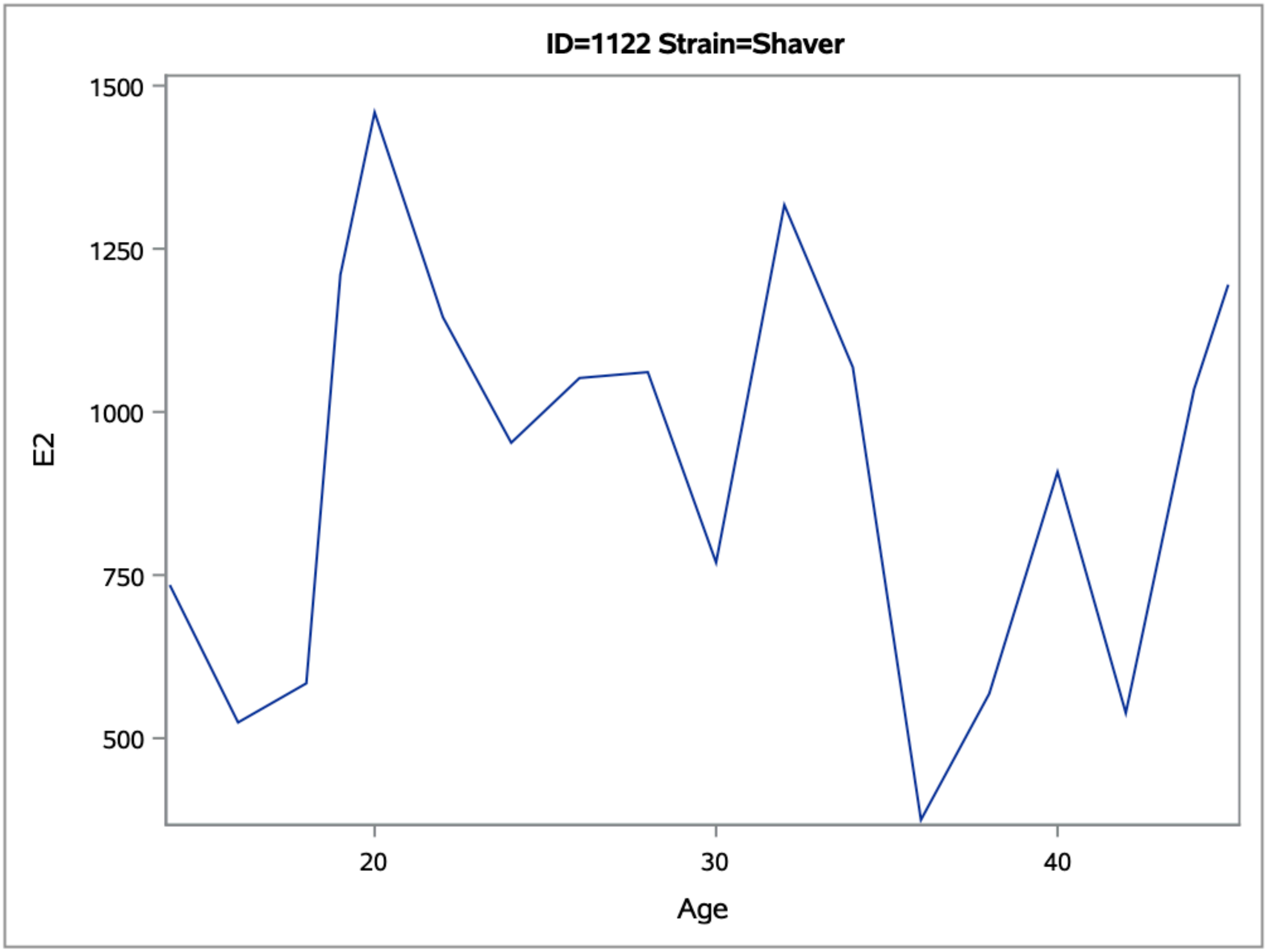
ID=1122 Strain=Shaver
1500
1250
1000
750
500
E2
20
30
40
Age

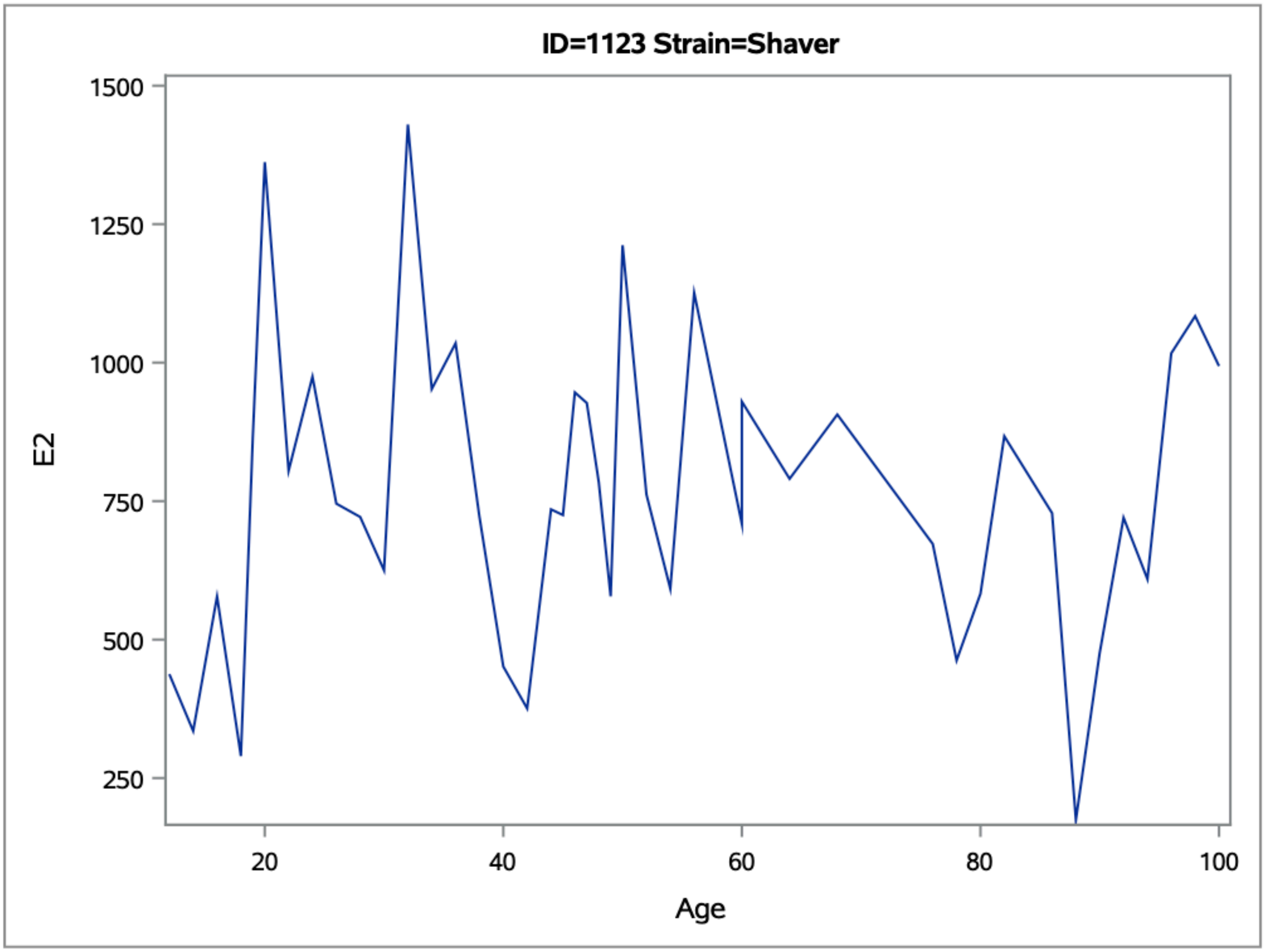

ID=1123 Strain=Shaver
1500
1250
1000
750
500
250
E2
20
40
60
80
100
Age

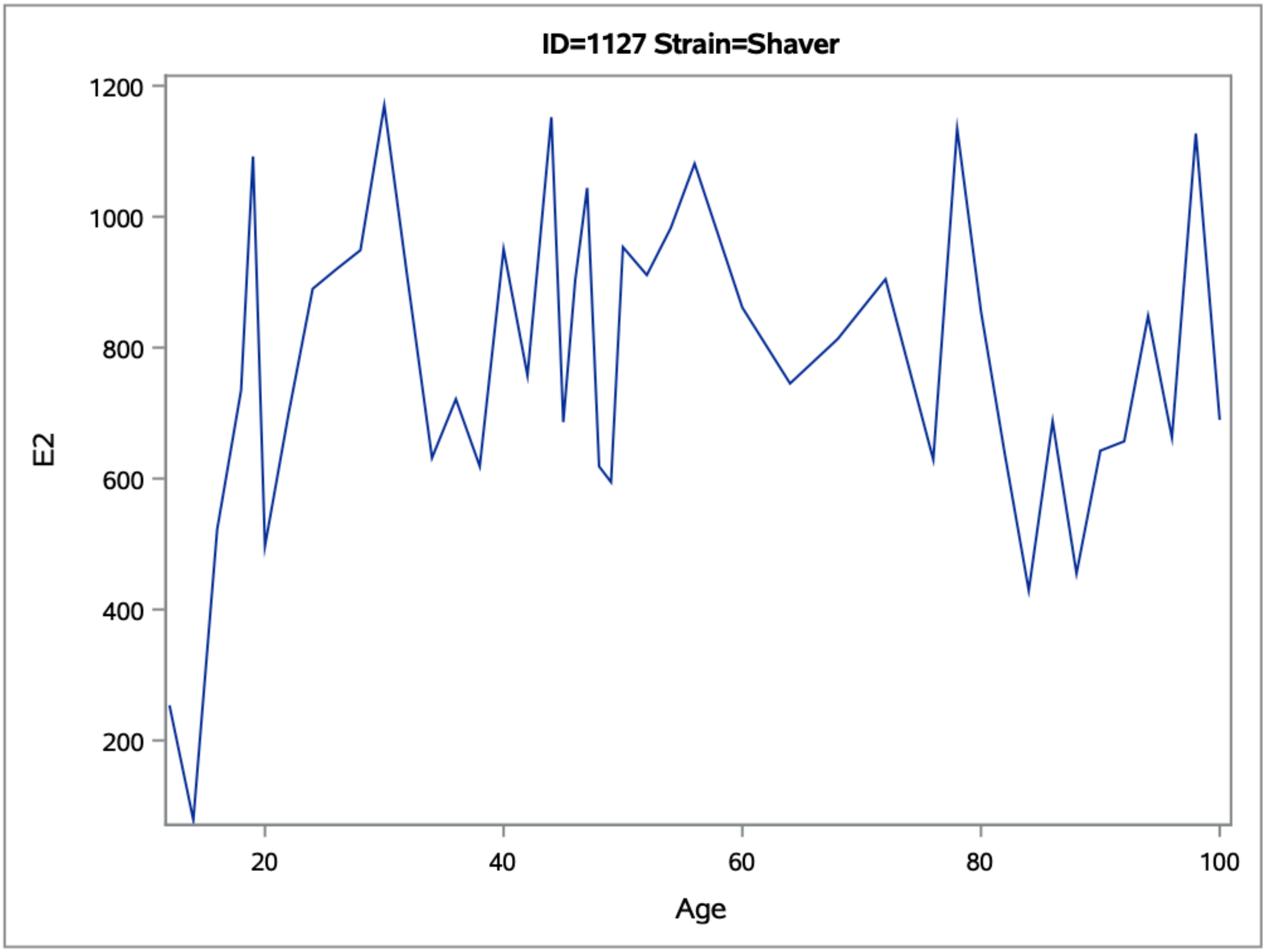

ID=1127 Strain=Shaver
1200
1000
800
600
400
200
E2
20
40
60
80
100
Age

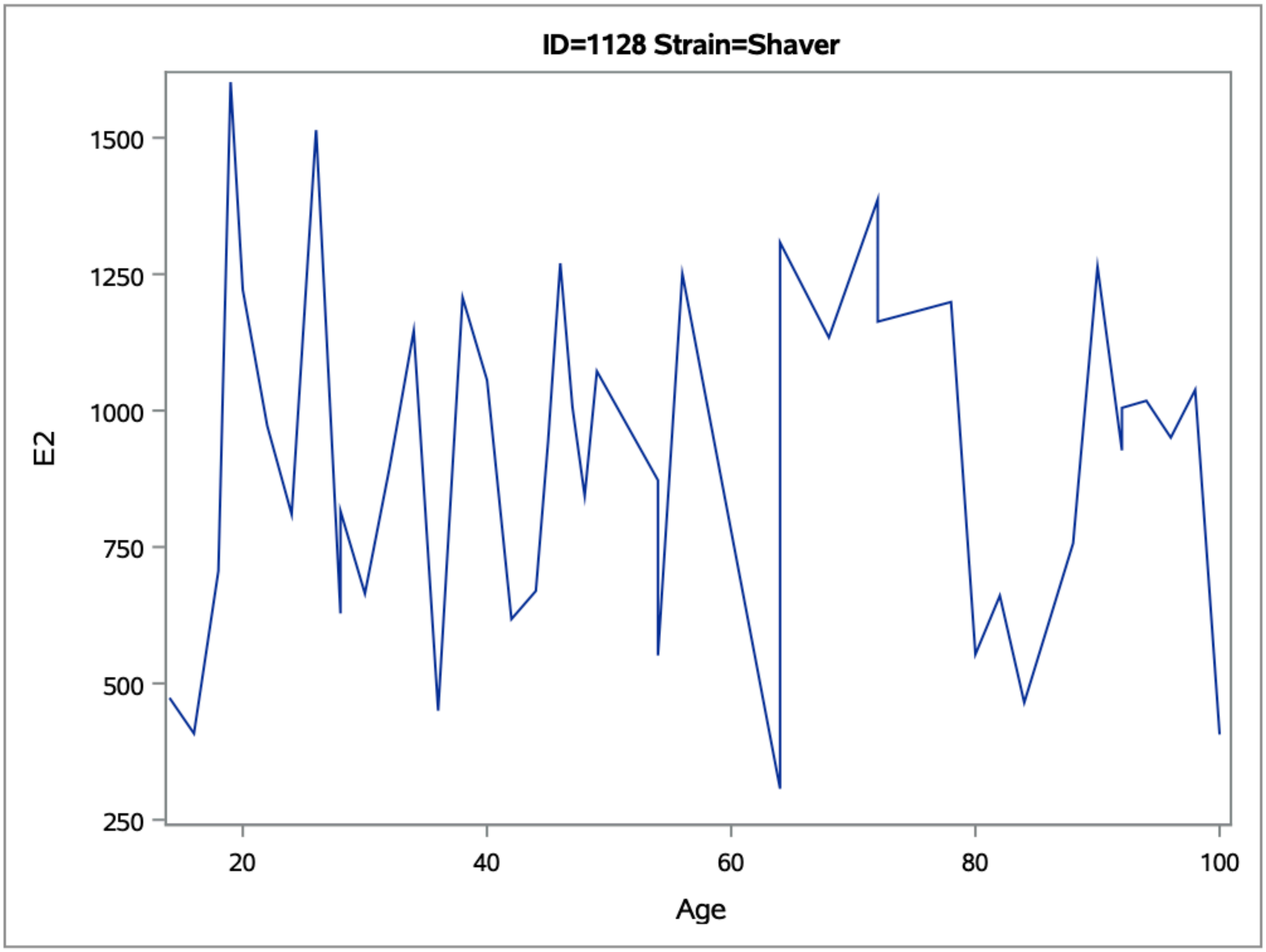
ID=1128 Strain=Shaver
E2
1500
1250
1000
750
500
250
20
40
60
80
100
Age

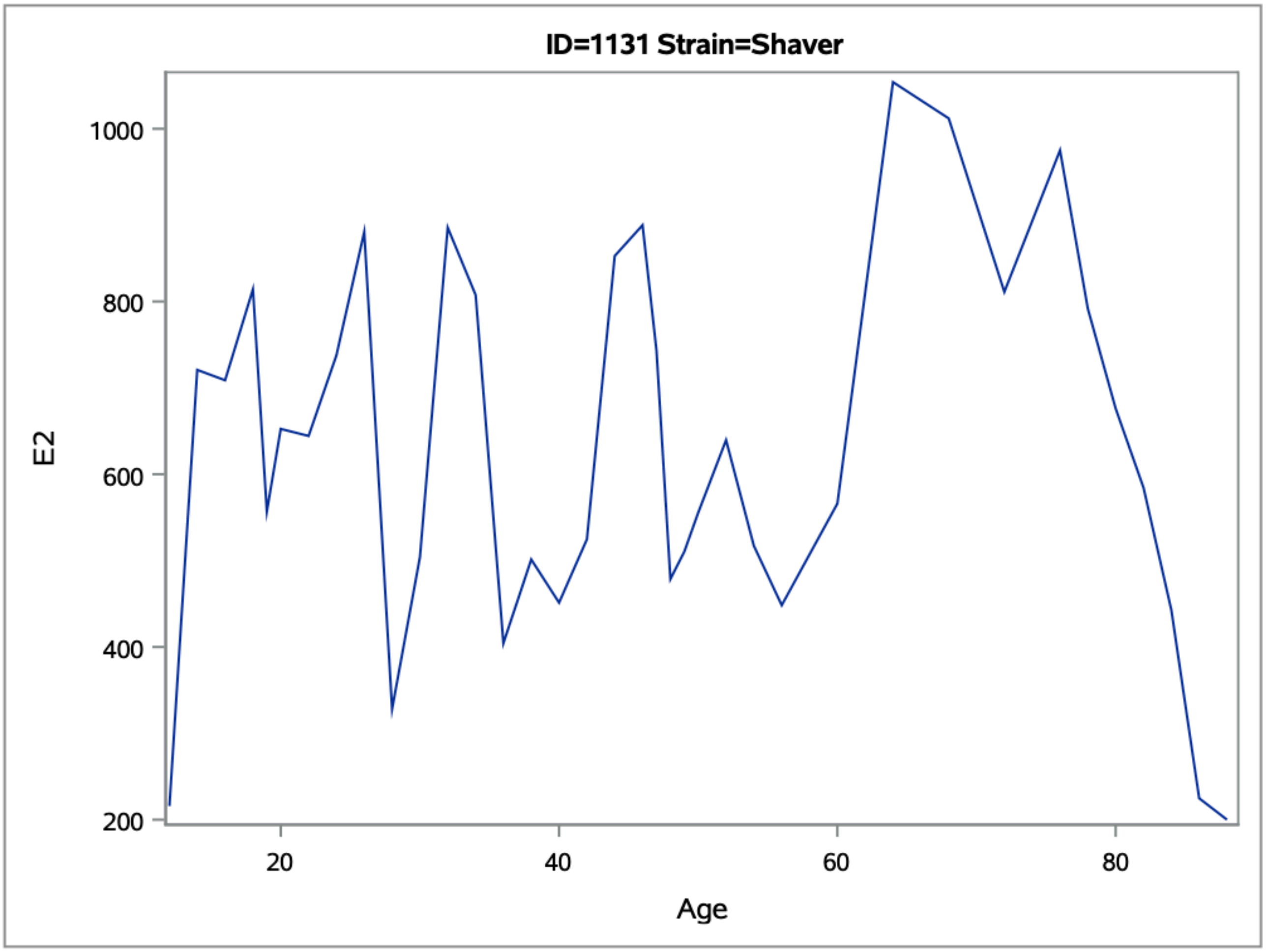
ID=1131 Strain=Shaver
E2
1000
800
600
400
200
20
40
60
80
Age

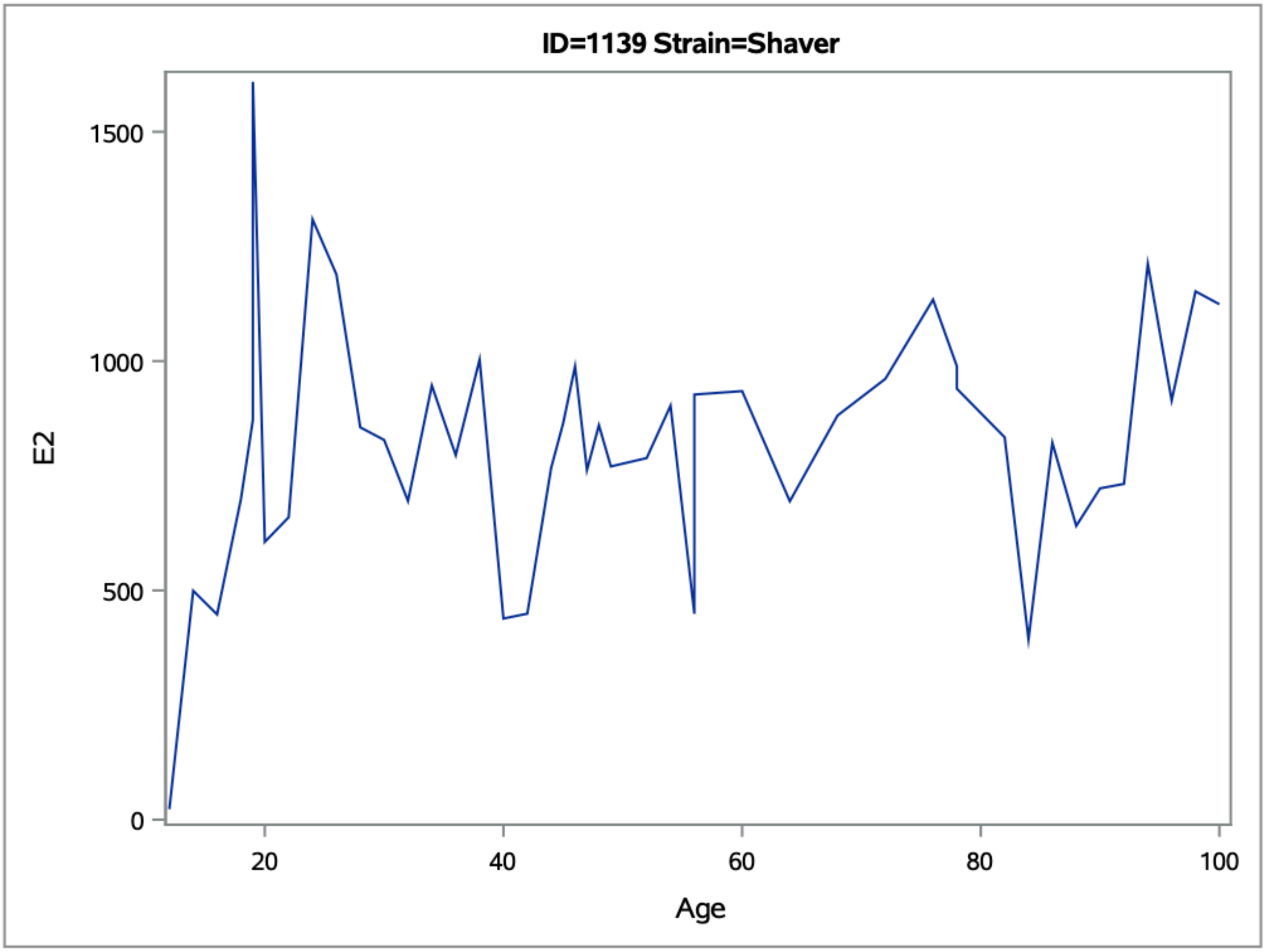
ID=1139 Strain=Shaver
1500
1000
500
0
E2
20
40
60
80
100
Age

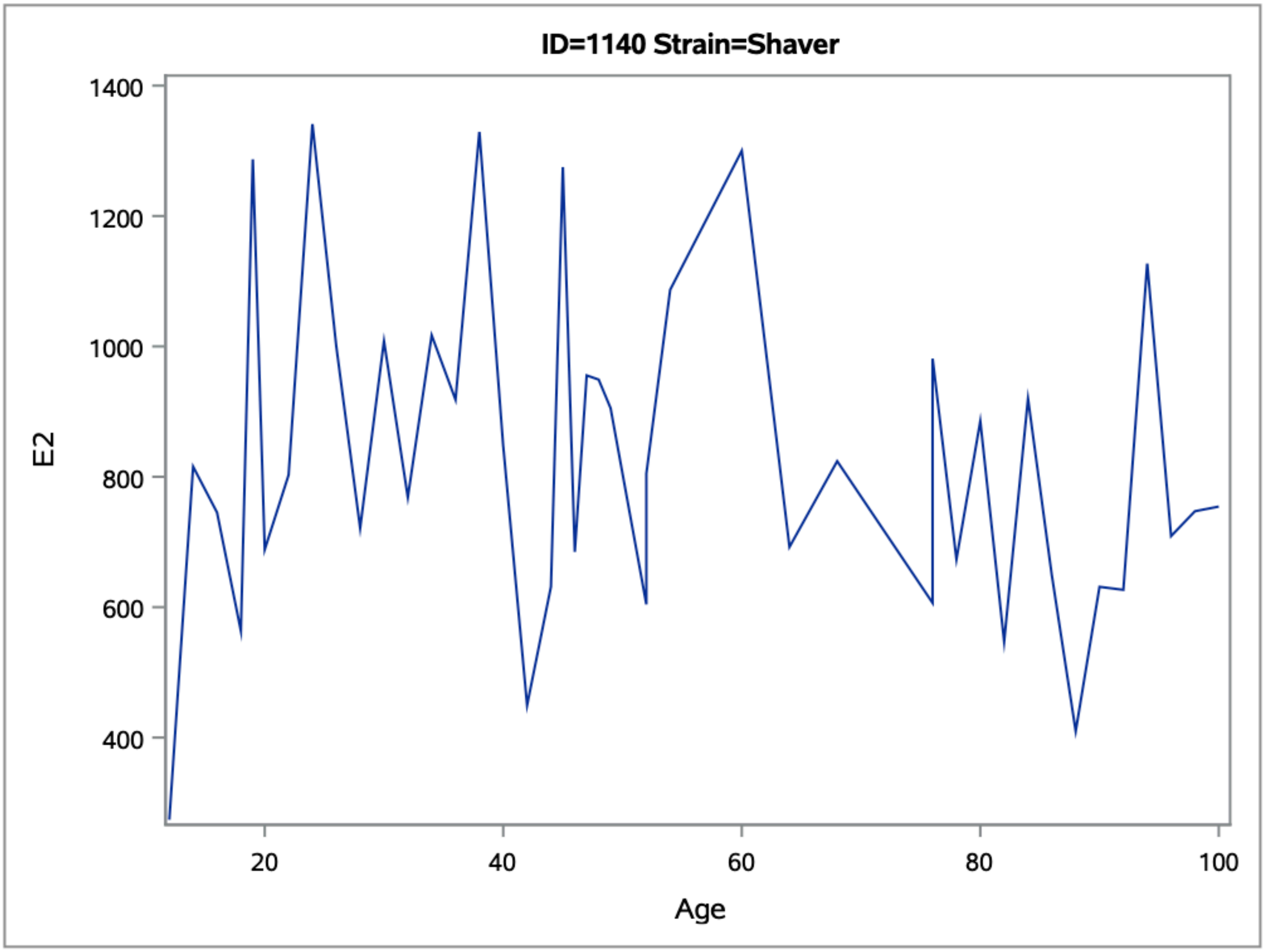
ID=1140 Strain=Shaver
1400
1200
1000
800
600
400
E2
20
40
60
80
100
Age

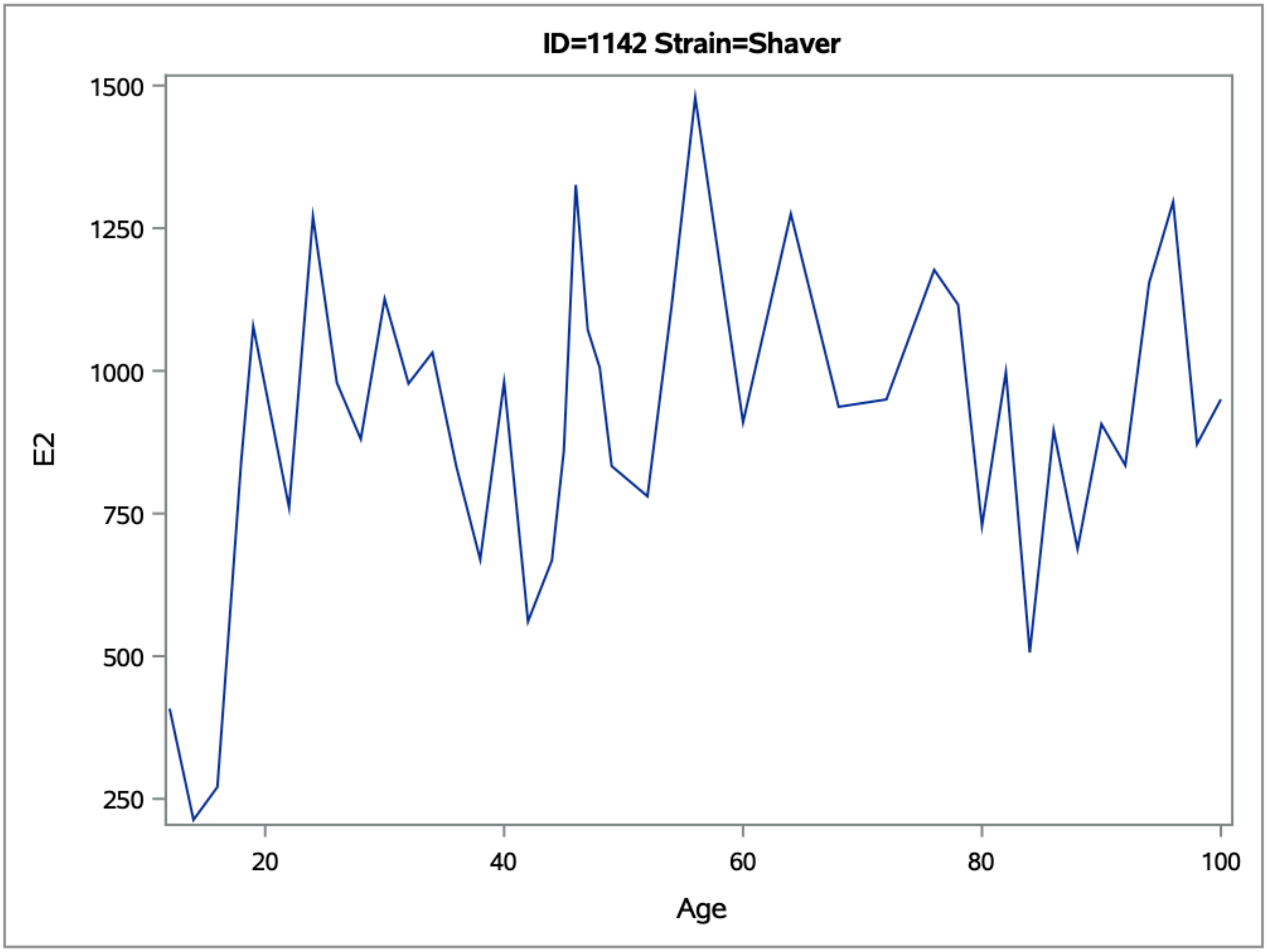
ID=1142 Strain=Shaver
1500
1250
1000
750
500
250
E2
20
40
60
80
100
Age

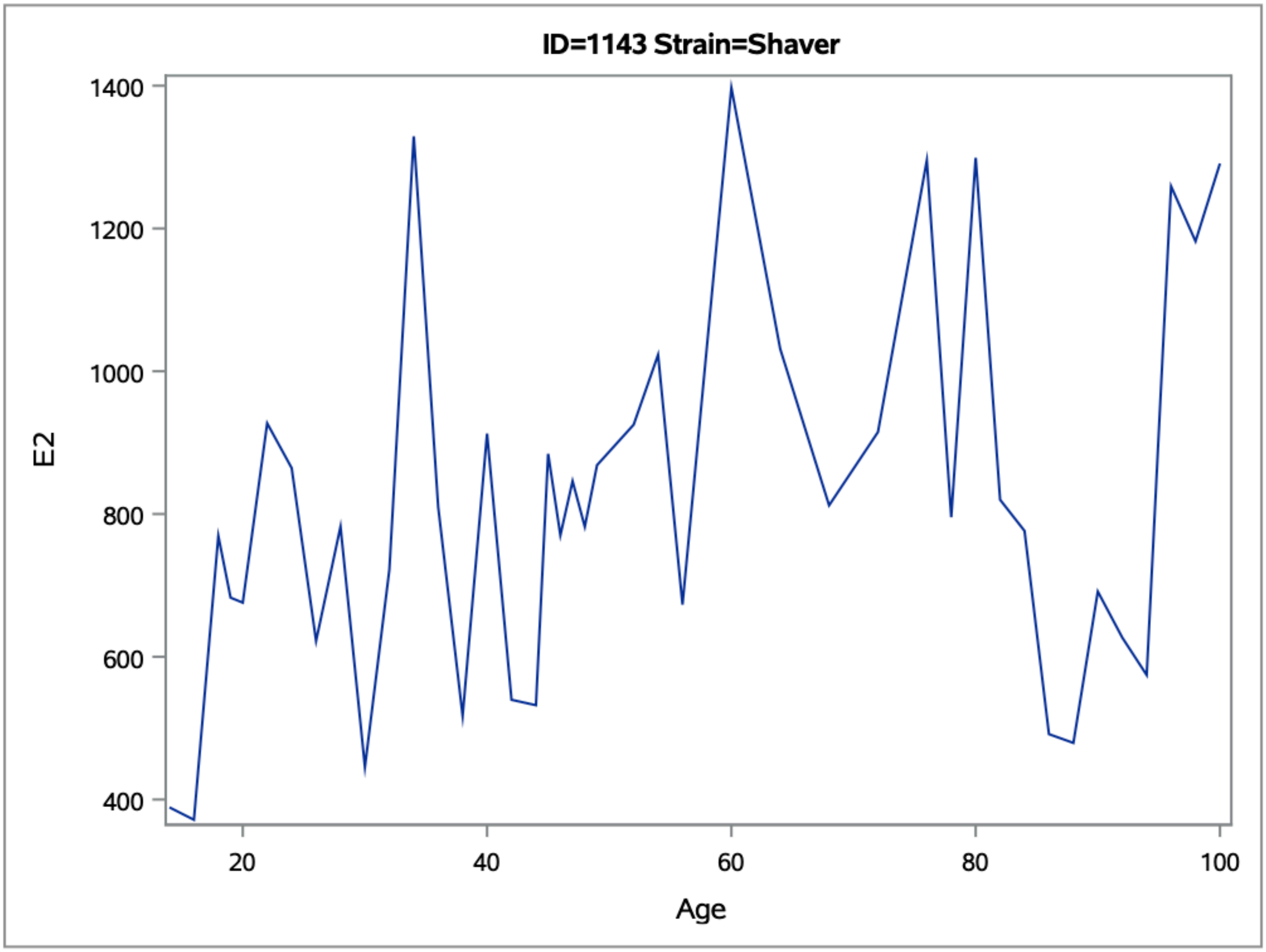

ID=1143 Strain=Shaver
1400
1200
1000
800
600
400
E2
20
40
60
80
100
Age

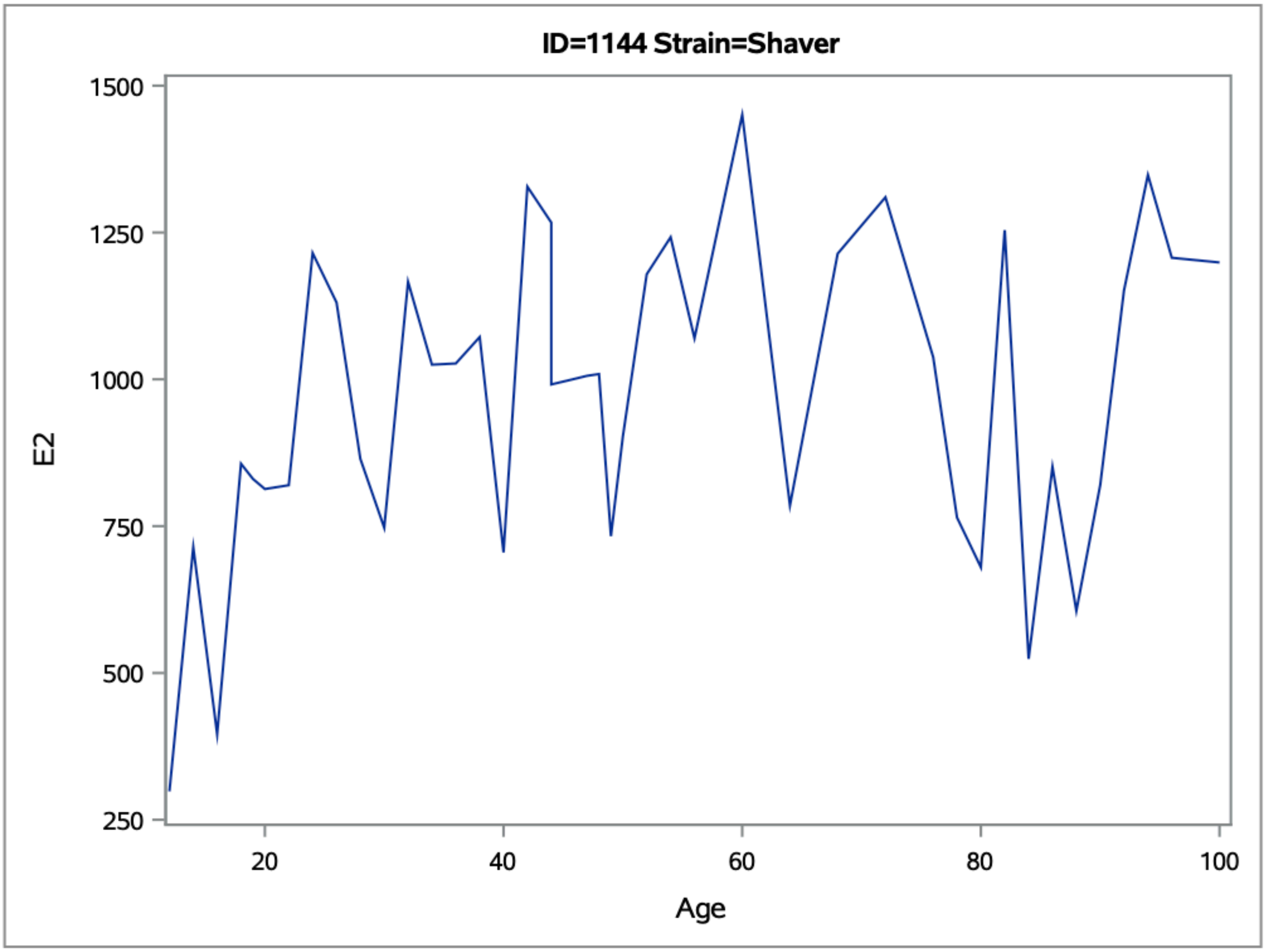
ID=1144 Strain=Shaver
1500
1250
1000
750
500
250
E2
20
40
60
80
100
Age

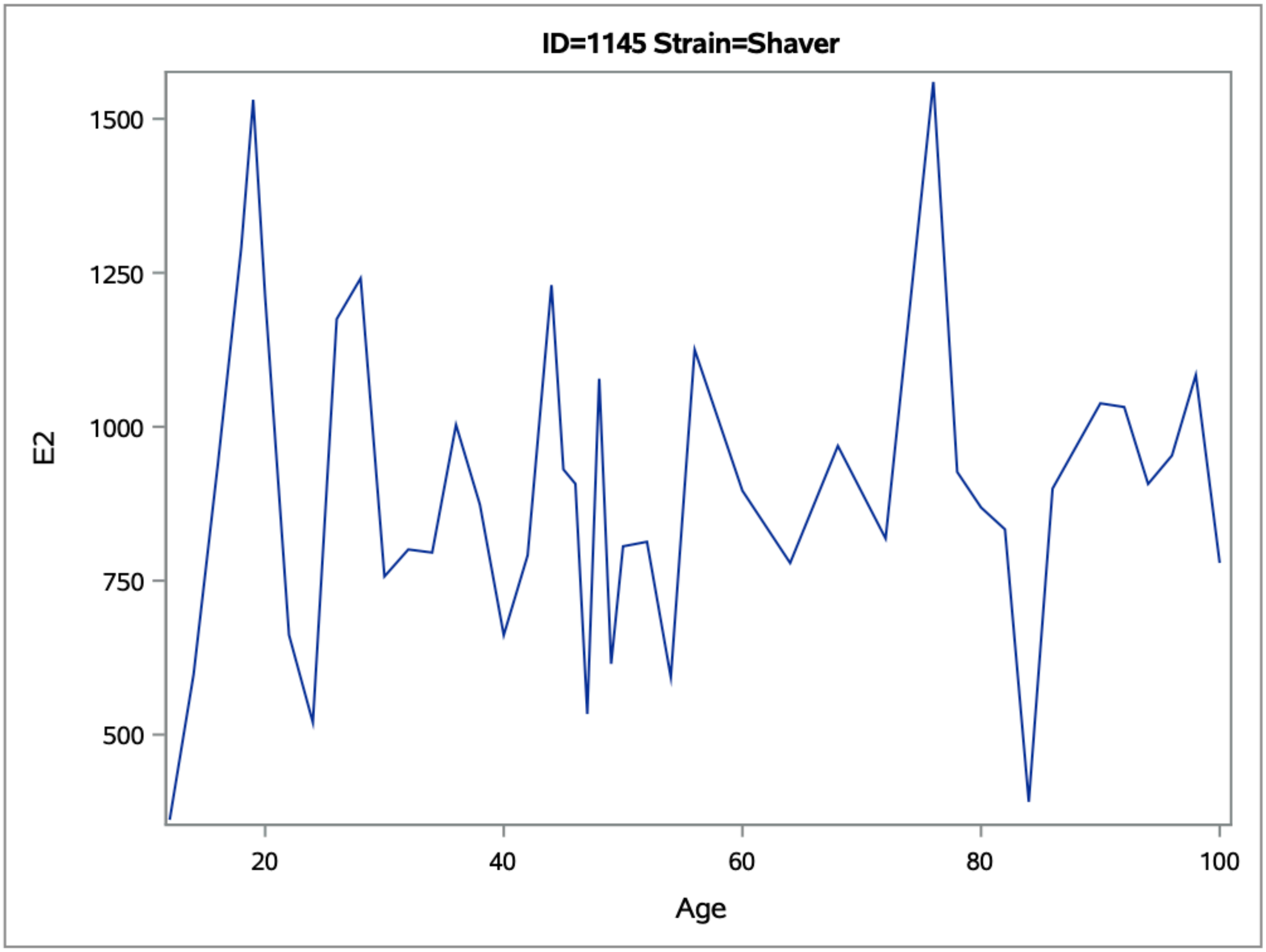
ID=1145 Strain=Shaver
1500
1250
1000
750
500
E2
20
40
60
80
100
Age

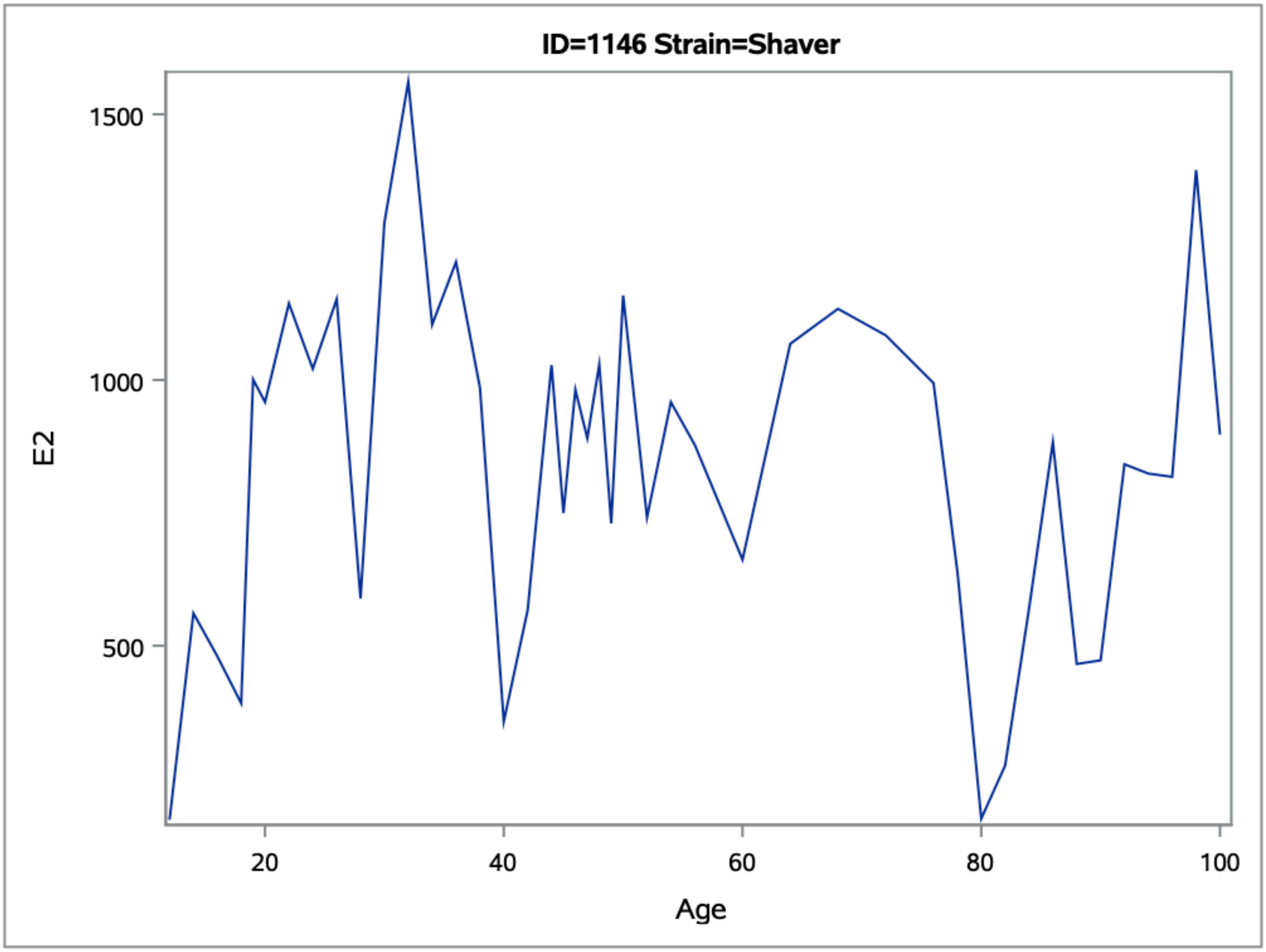
ID=1146 Strain=Shaver
1500
1000
500
E2
20
40
60
80
100
Age

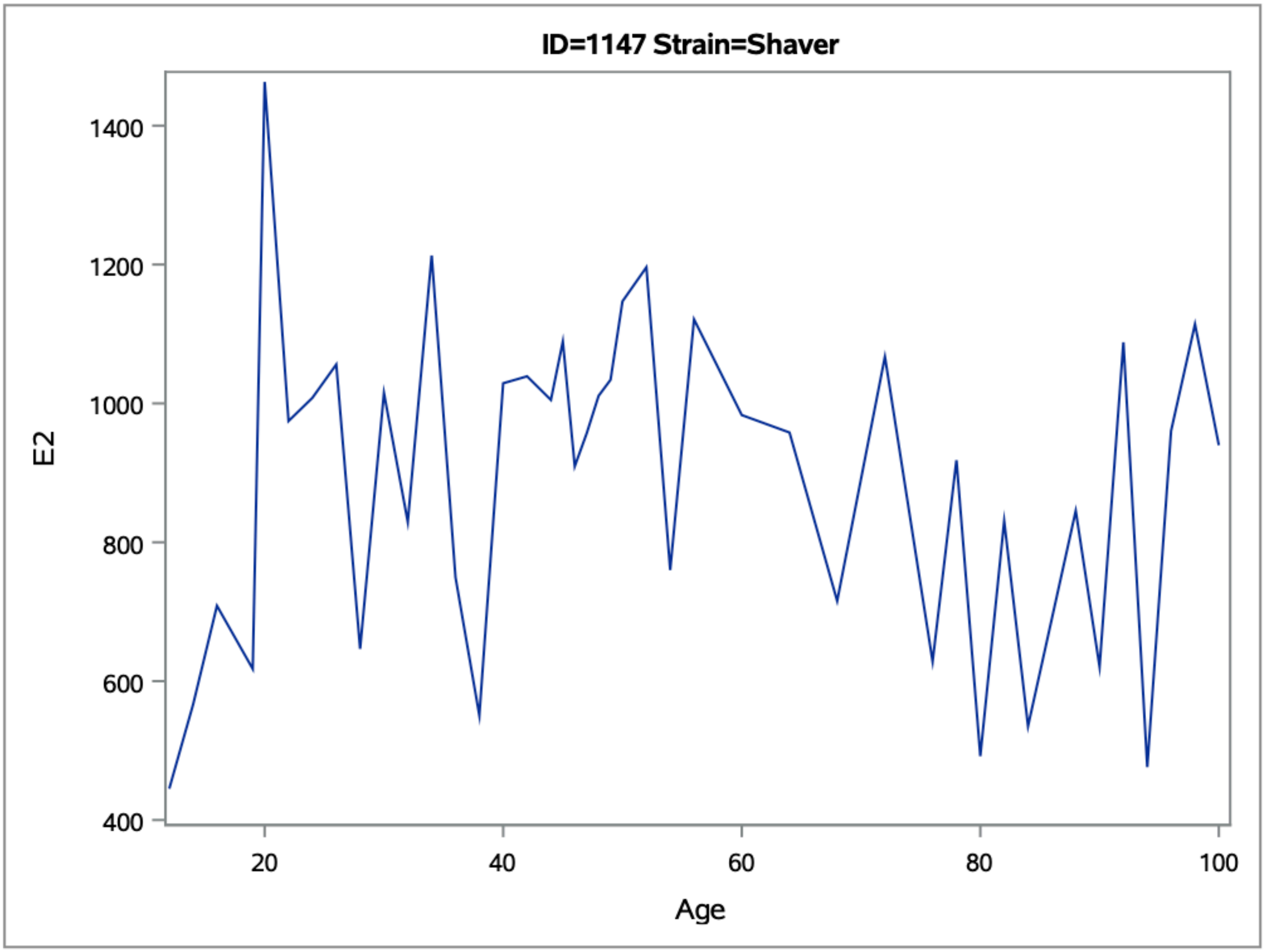
ID=1147 Strain=Shaver
E2
1400
1200
1000
800
600
400
20
40
60
80
100
Age

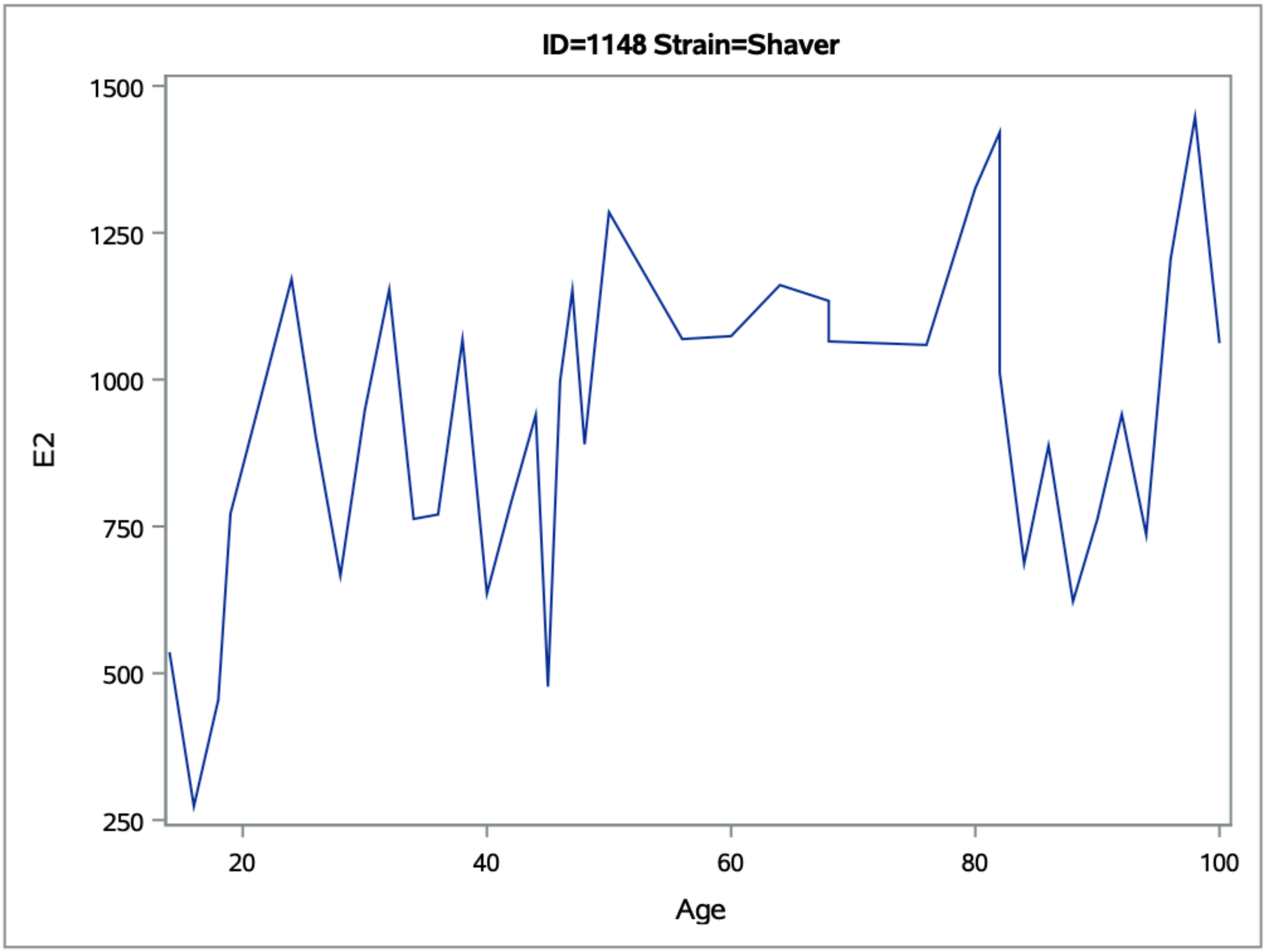
ID=1148 Strain=Shaver
1500
1250
1000
750
500
250
E2
20
40
60
80
100
Age

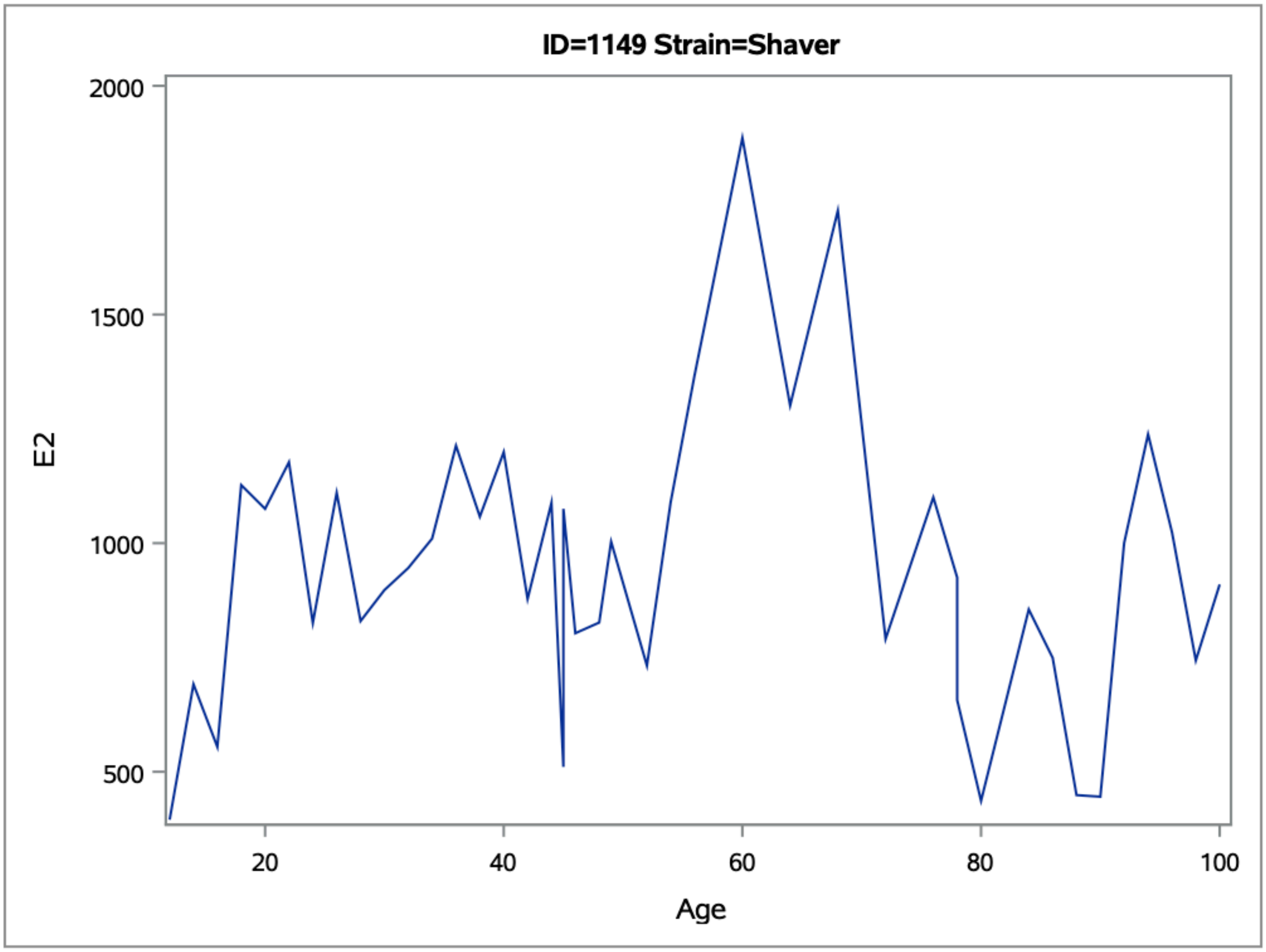
ID=1149 Strain=Shaver
2000
1500
1000
500
E2
20
40
60
80
100
Age

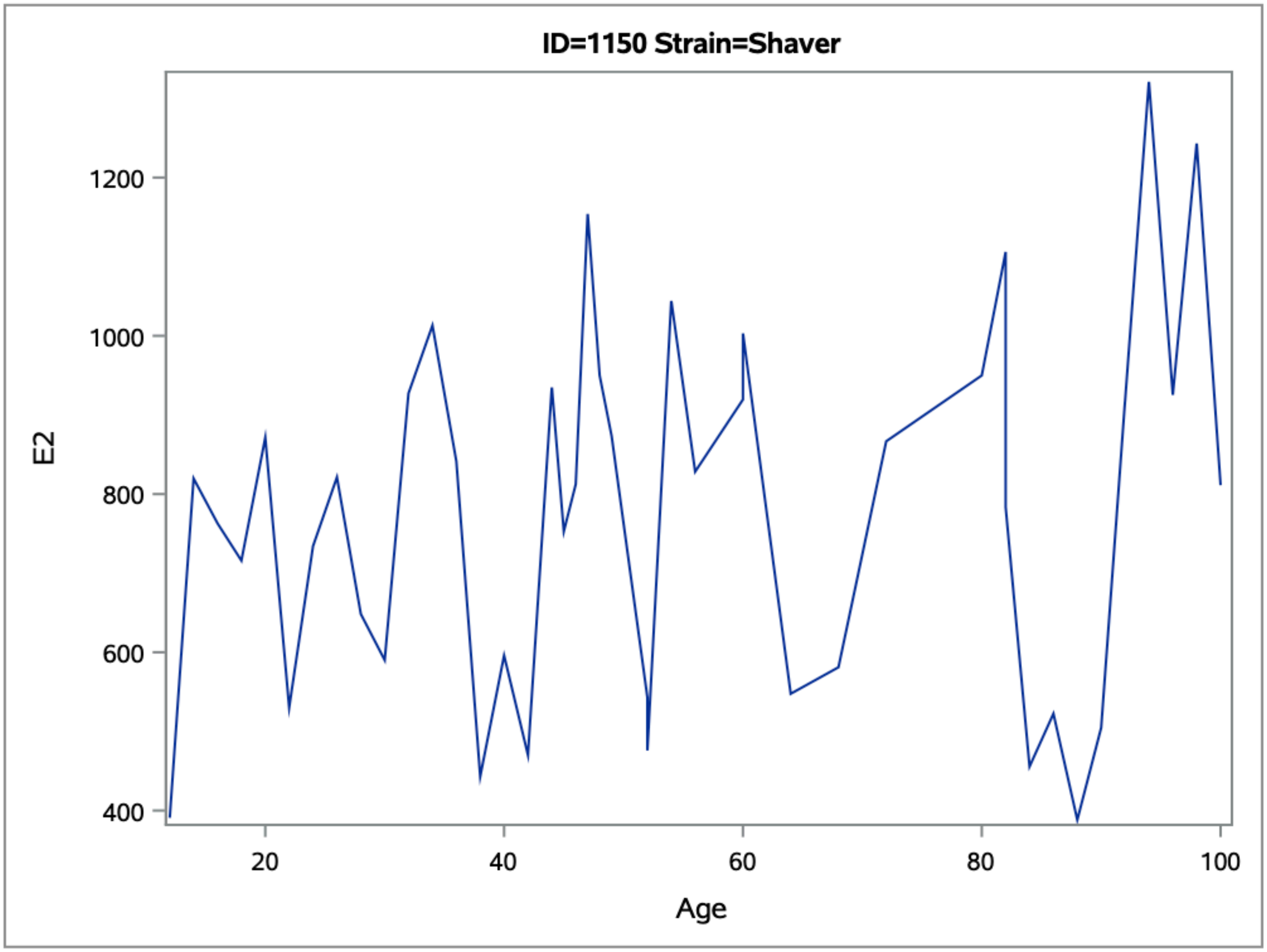

ID=1150 Strain=Shaver
E2
1200
1000
800
600
400
20
40
60
80
100
Age

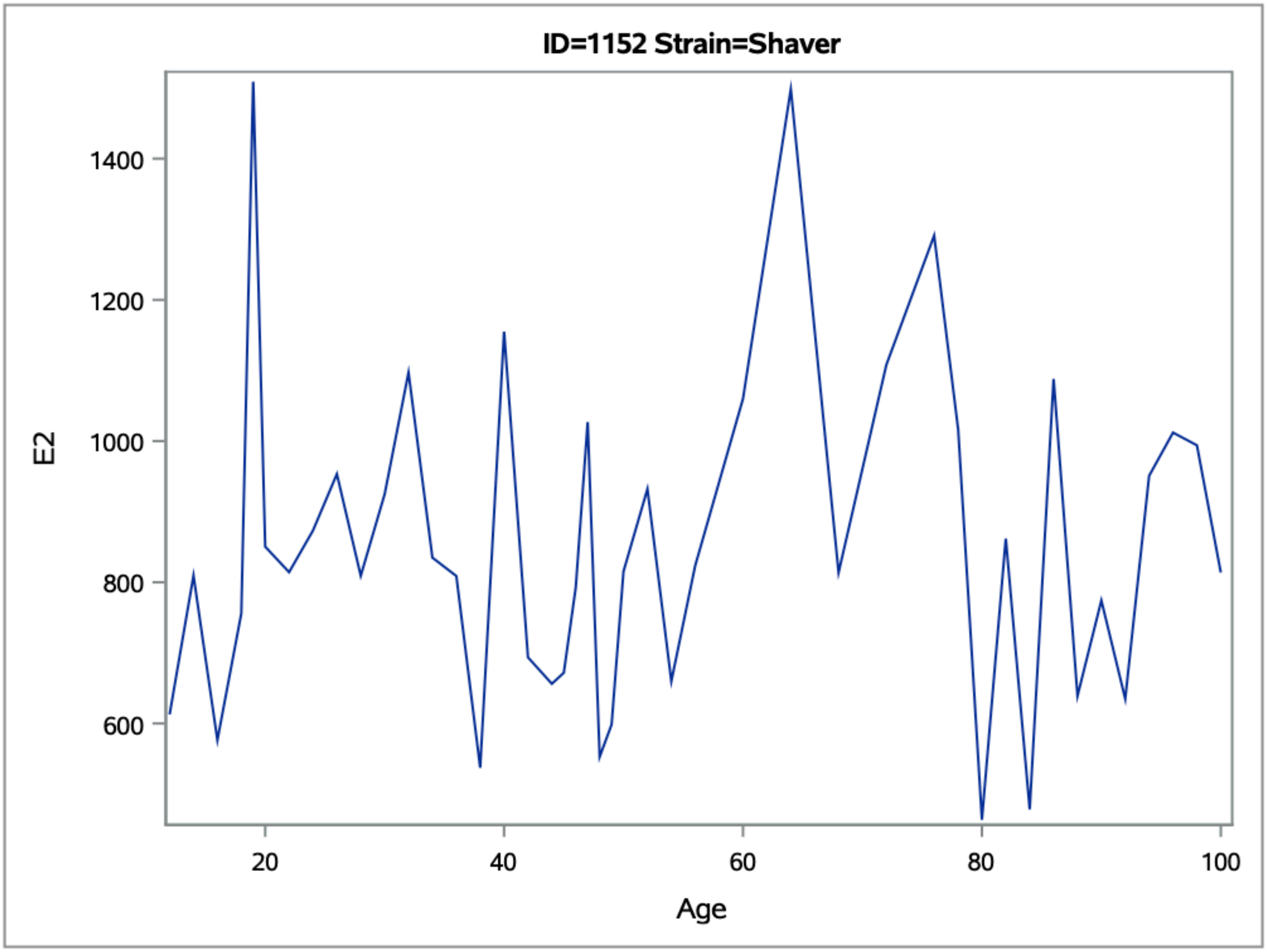

ID=1152 Strain=Shaver
1400
1200
1000
800
600
E2
20
40
60
80
100
Age

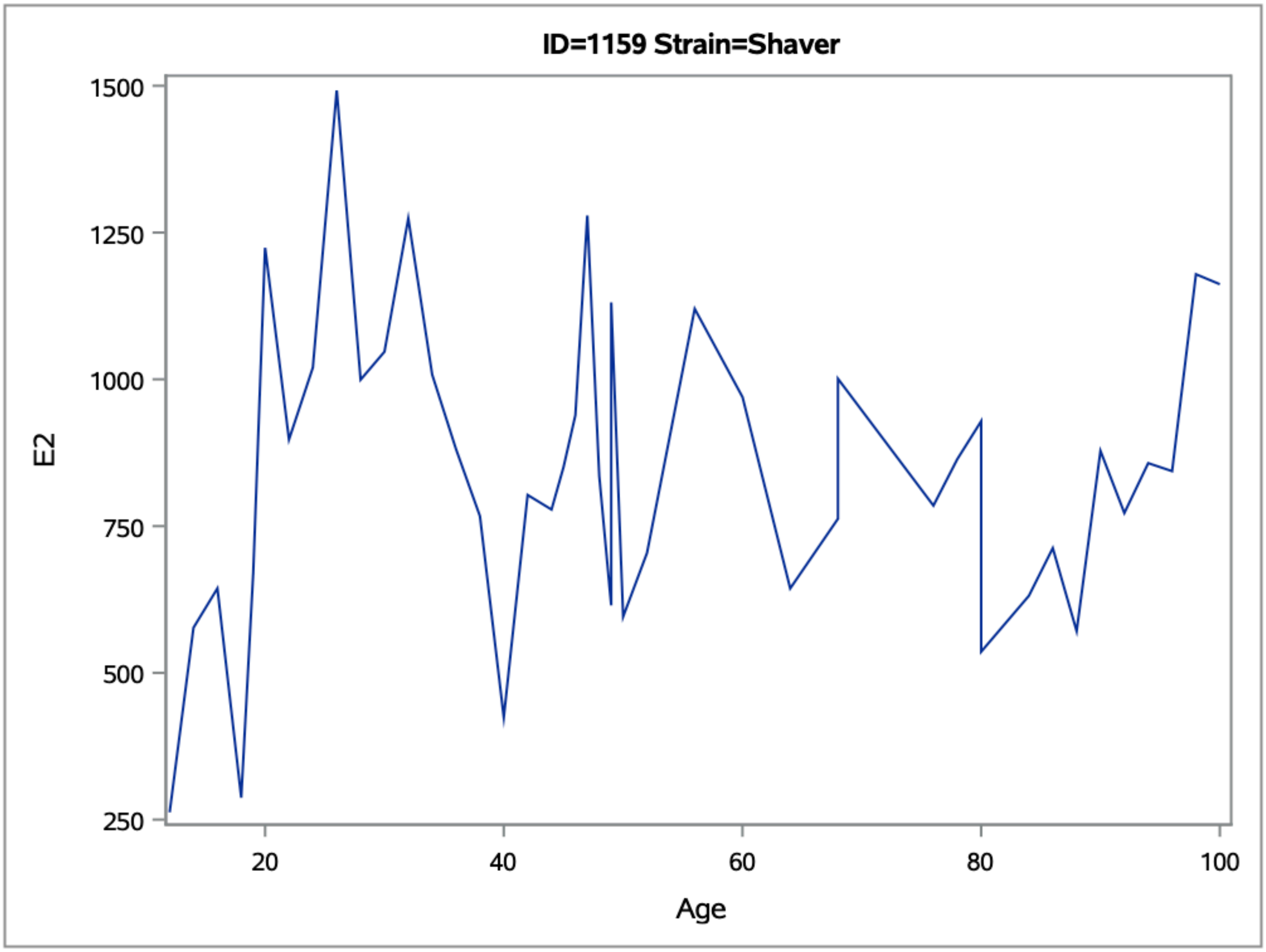
ID=1159 Strain=Shaver
1500
1250
1000
750
500
250
E2
20
40
60
80
100
Age

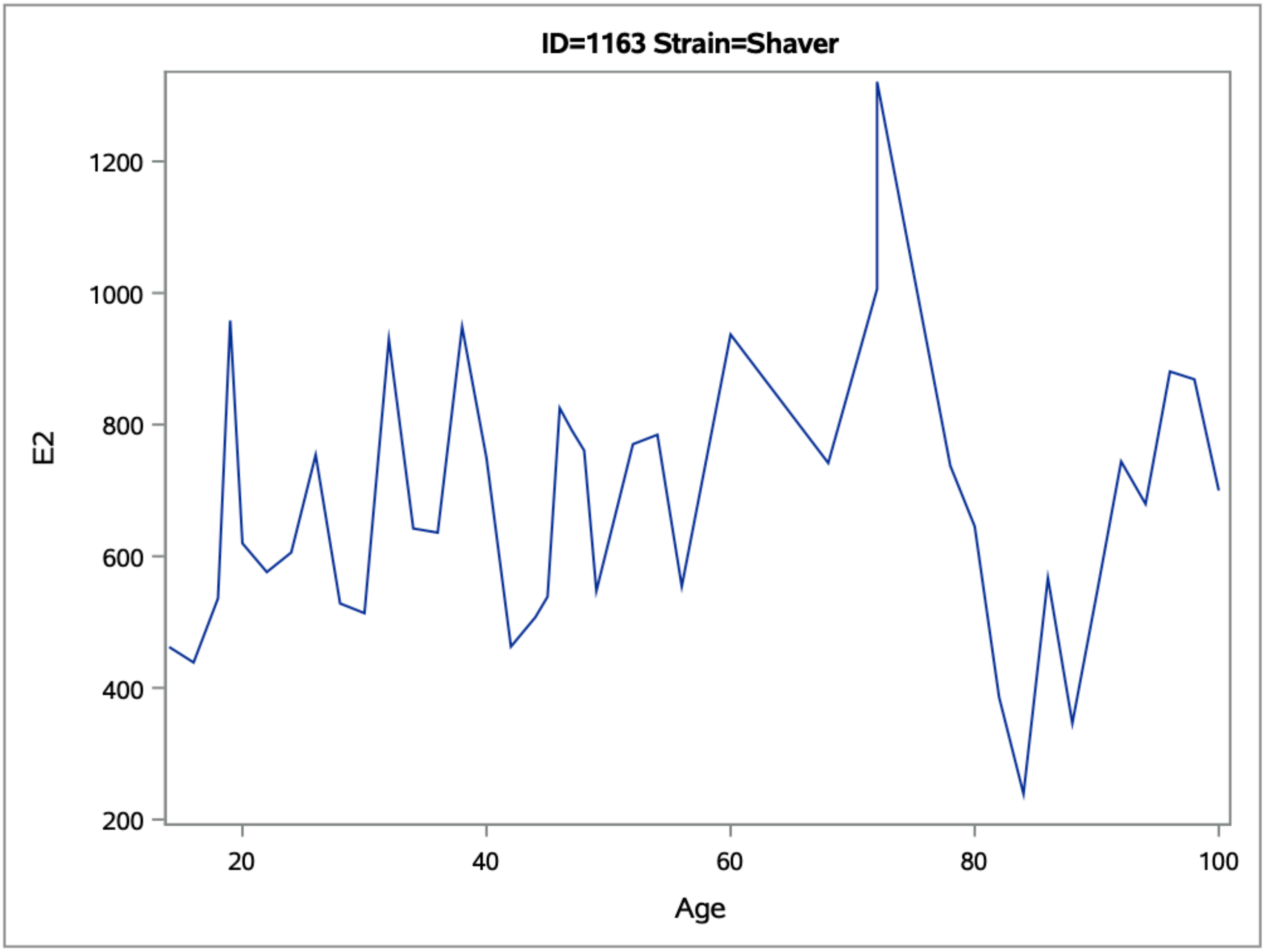
ID=1163 Strain=Shaver
E2
1200
1000
800
600
400
200
20
40
60
80
100
Age

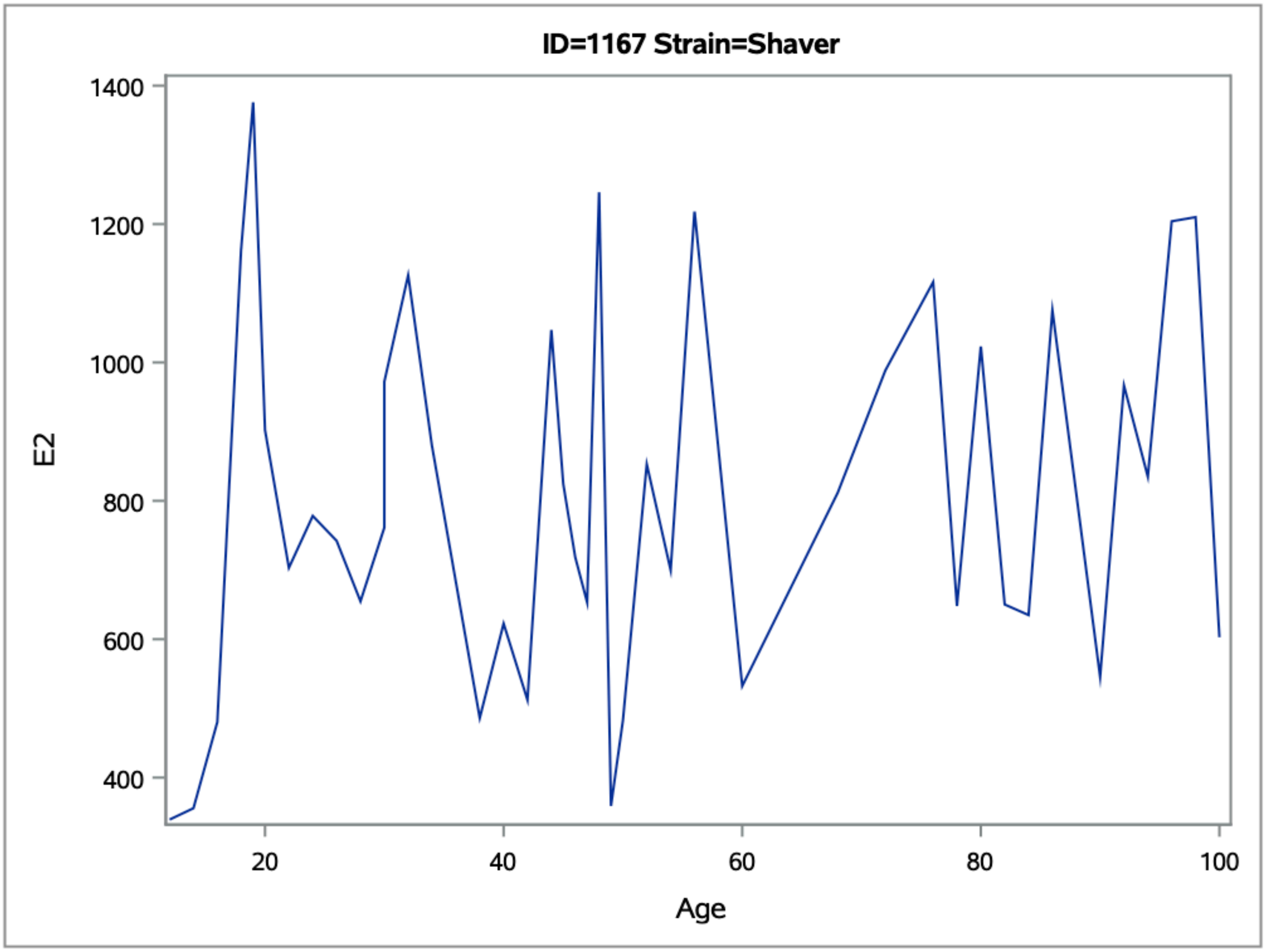
ID=1167 Strain=Shaver
E2
1400
1200
1000
800
600
400
20
40
60
80
100
Age

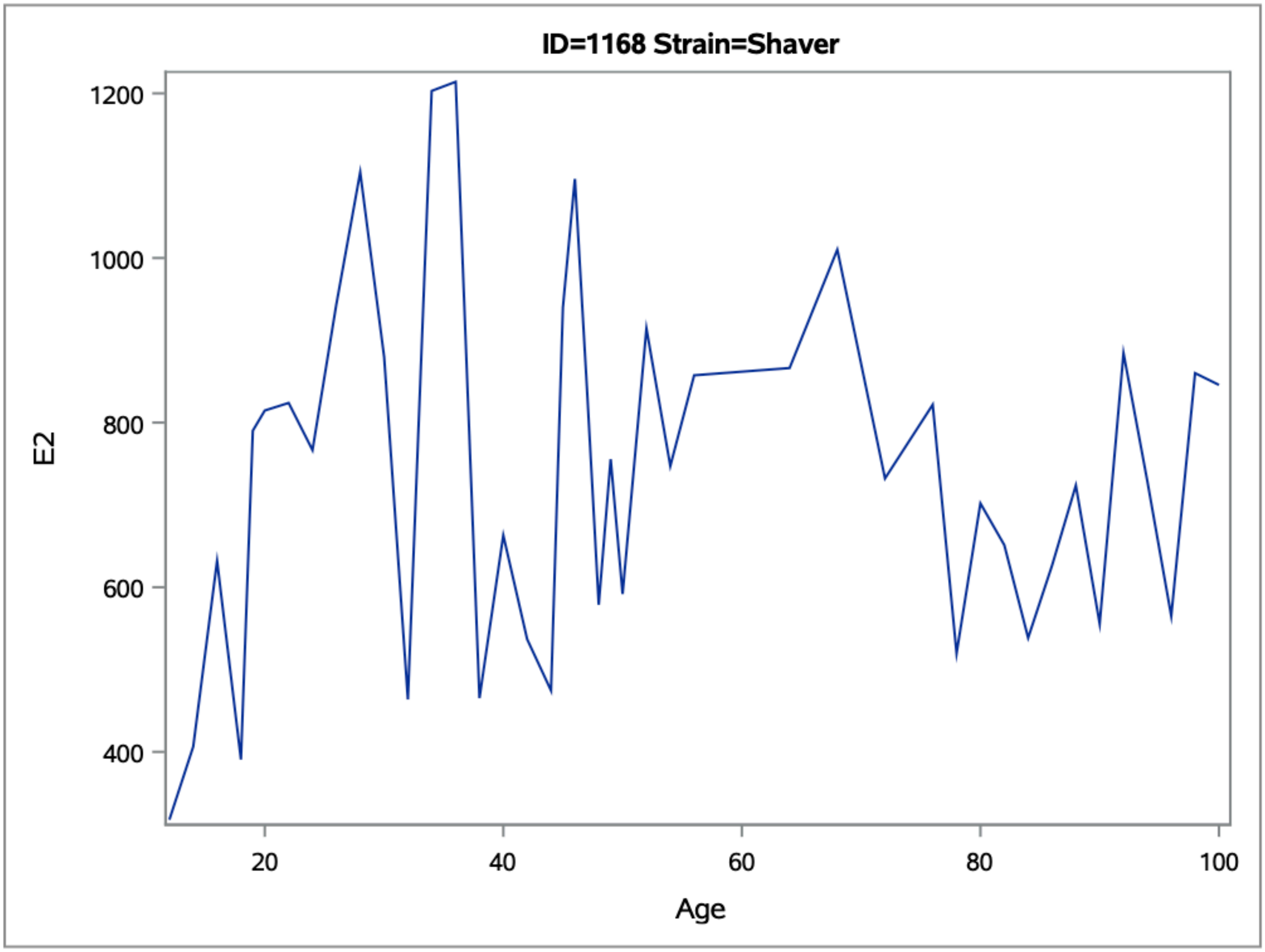
ID=1168 Strain=Shaver
1200
1000
800
600
400
E2
20
40
60
80
100
Age

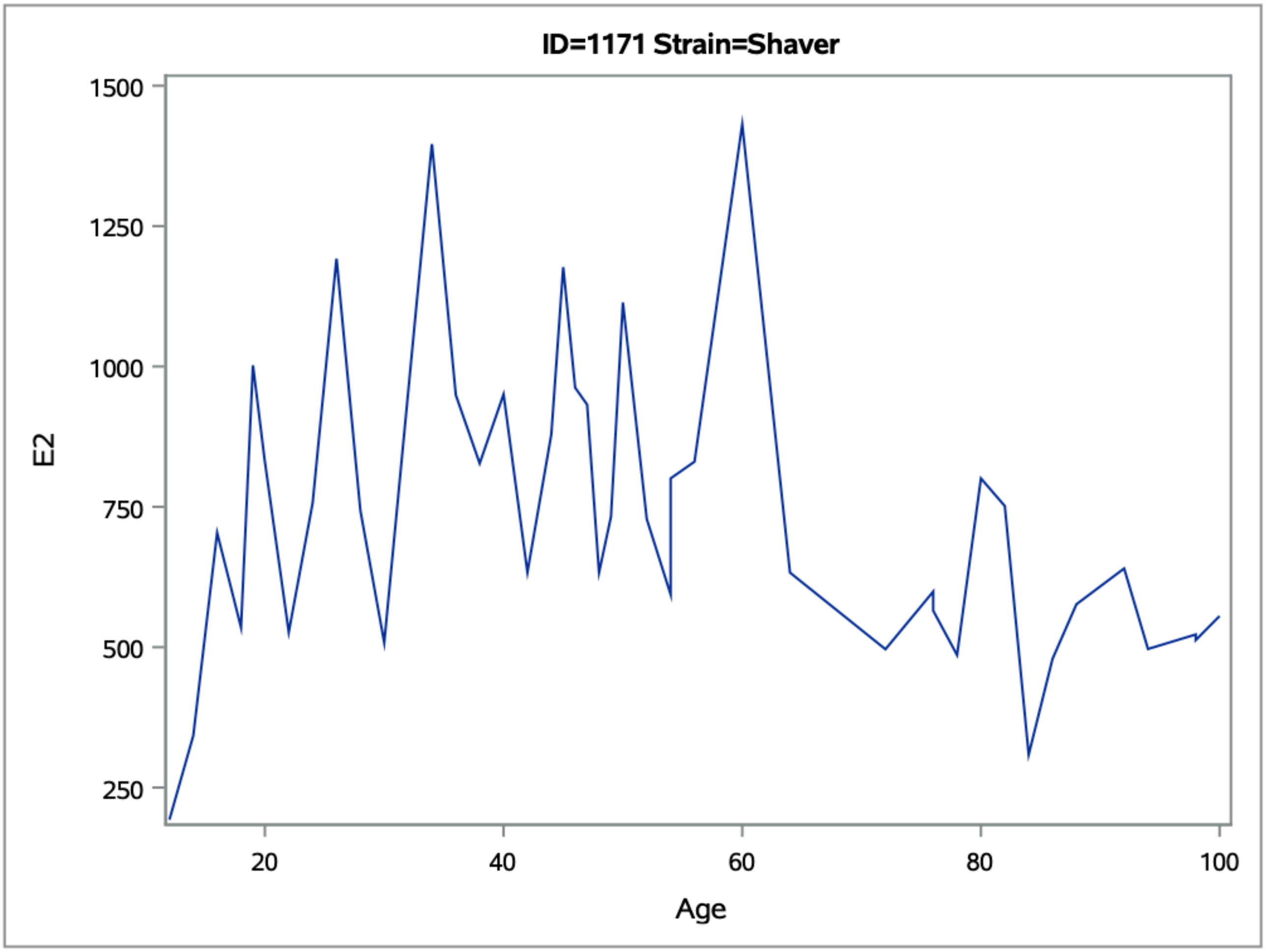
ID=1171 Strain=Shaver
1500
1250
1000
750
500
250
E2
20
40
60
80
100
Age

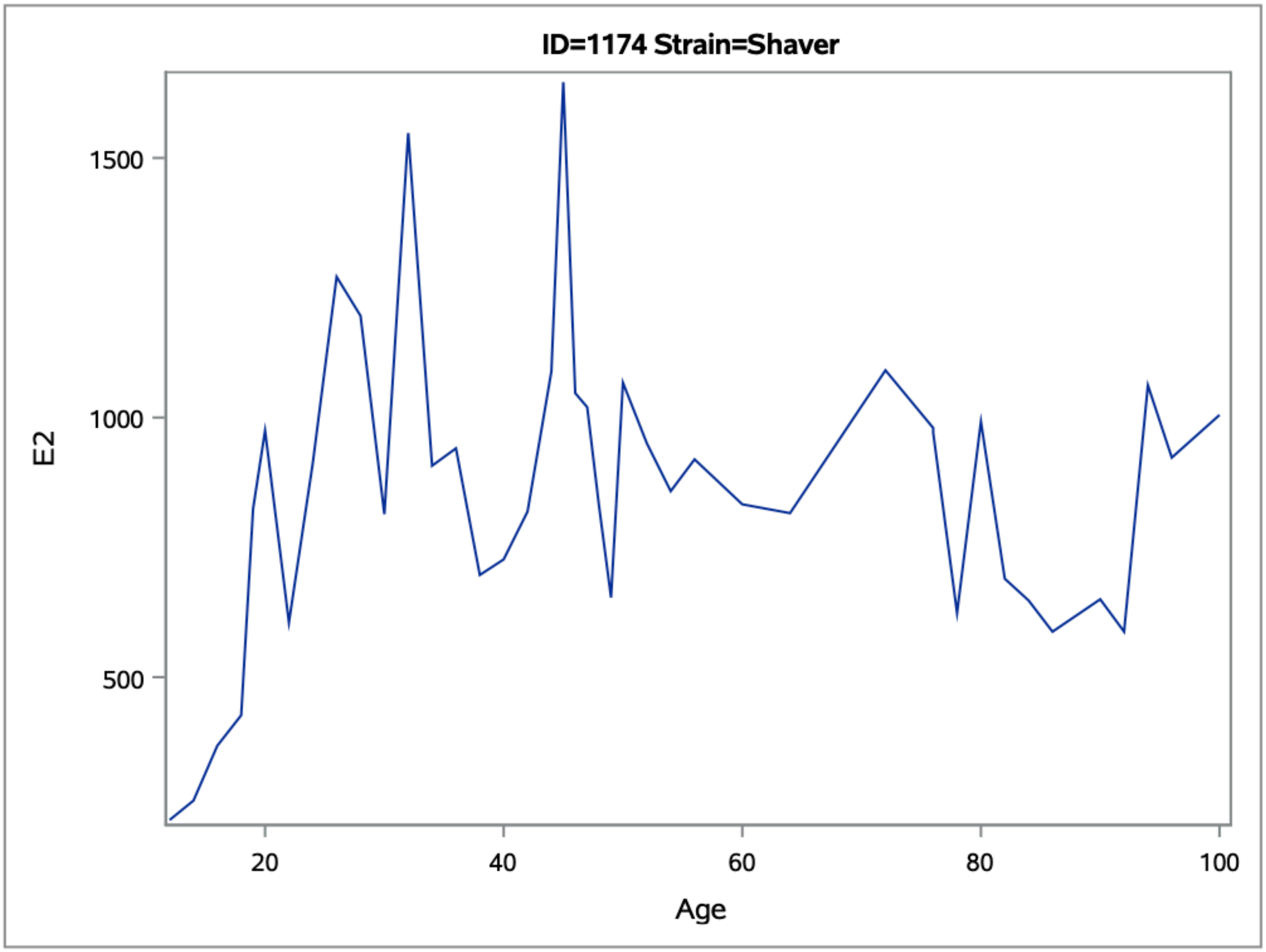
ID=1174 Strain=Shaver
E2
1500
1000
500
20
40
60
80
100
Age

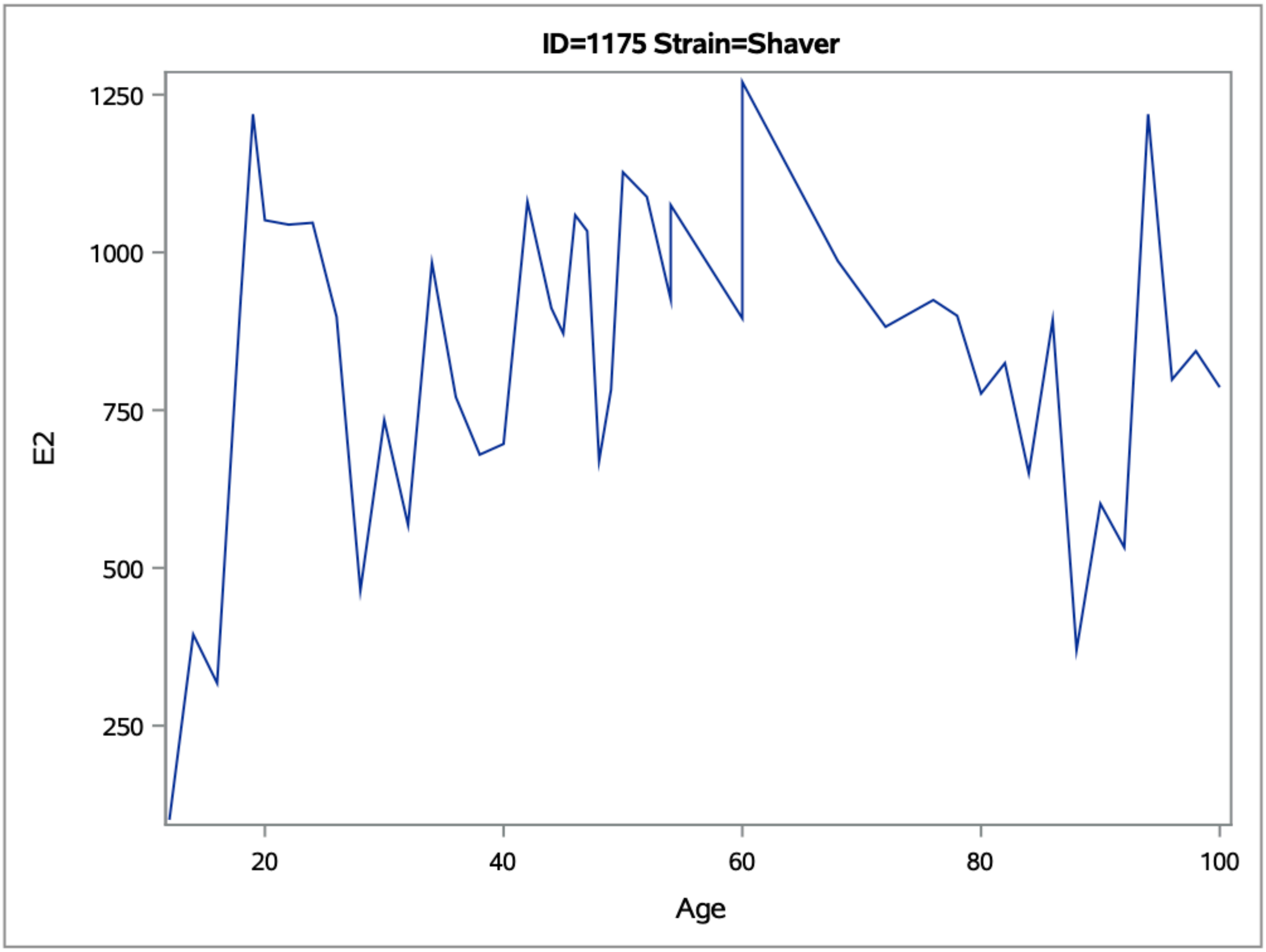
ID=1175 Strain=Shaver
1250
1000
750
500
250
E2
20
40
60
80
100
Age

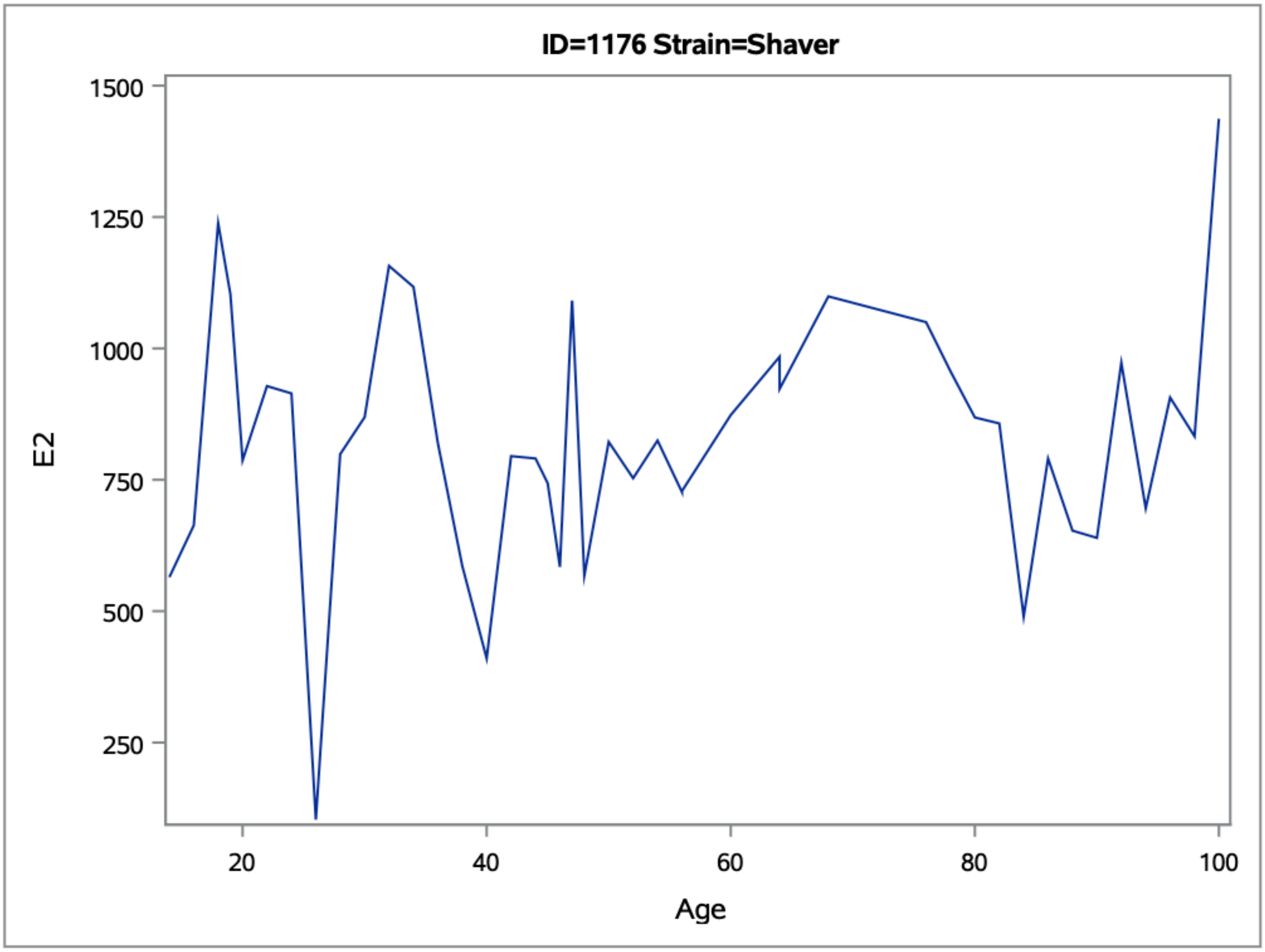
ID=1176 Strain=Shaver
1500
1250
1000
750
500
250
E2
20
40
60
80
100
Age

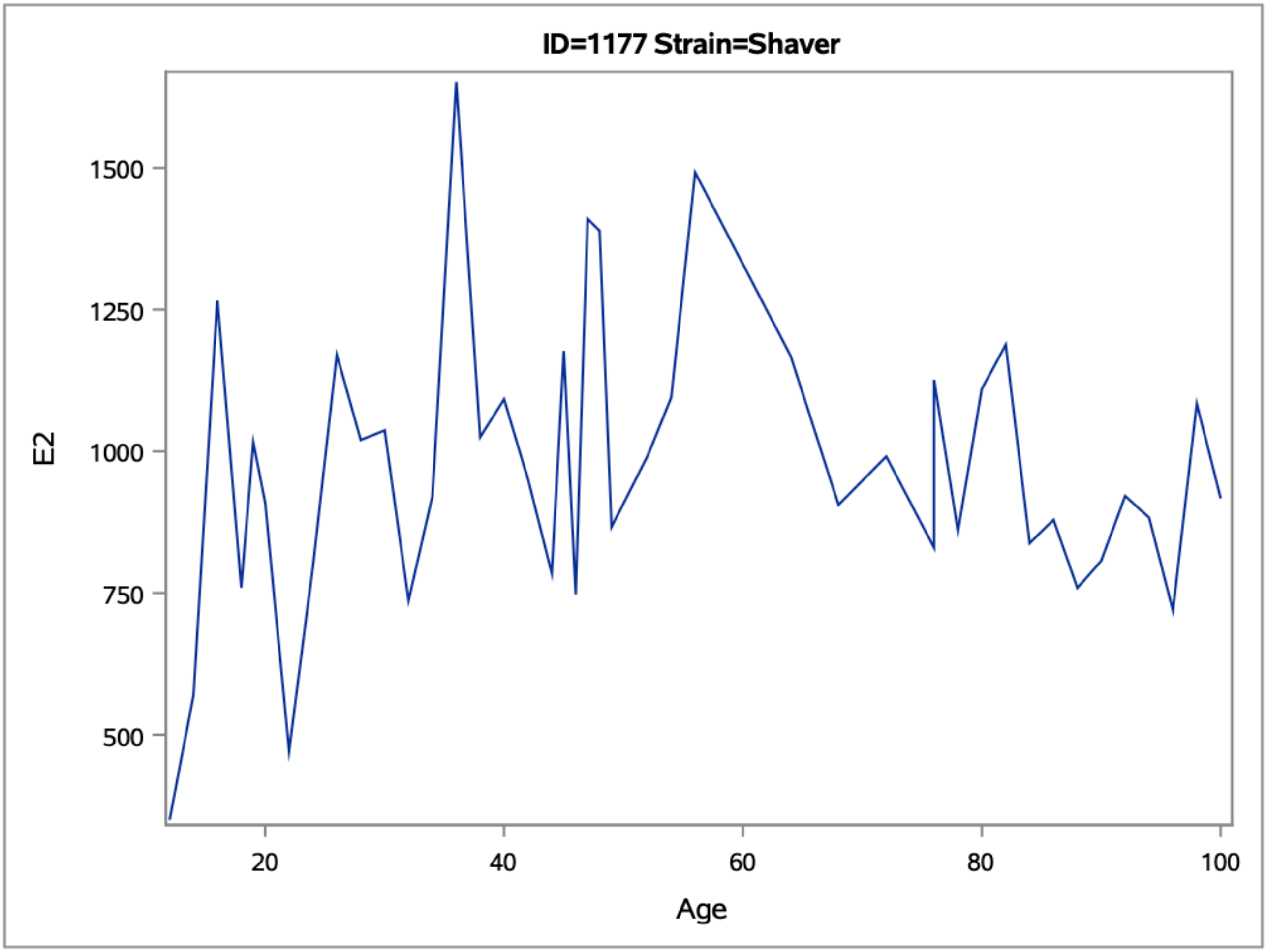
ID=1177 Strain=Shaver
E2
1500
1250
1000
750
500
20
40
60
80
100
Age

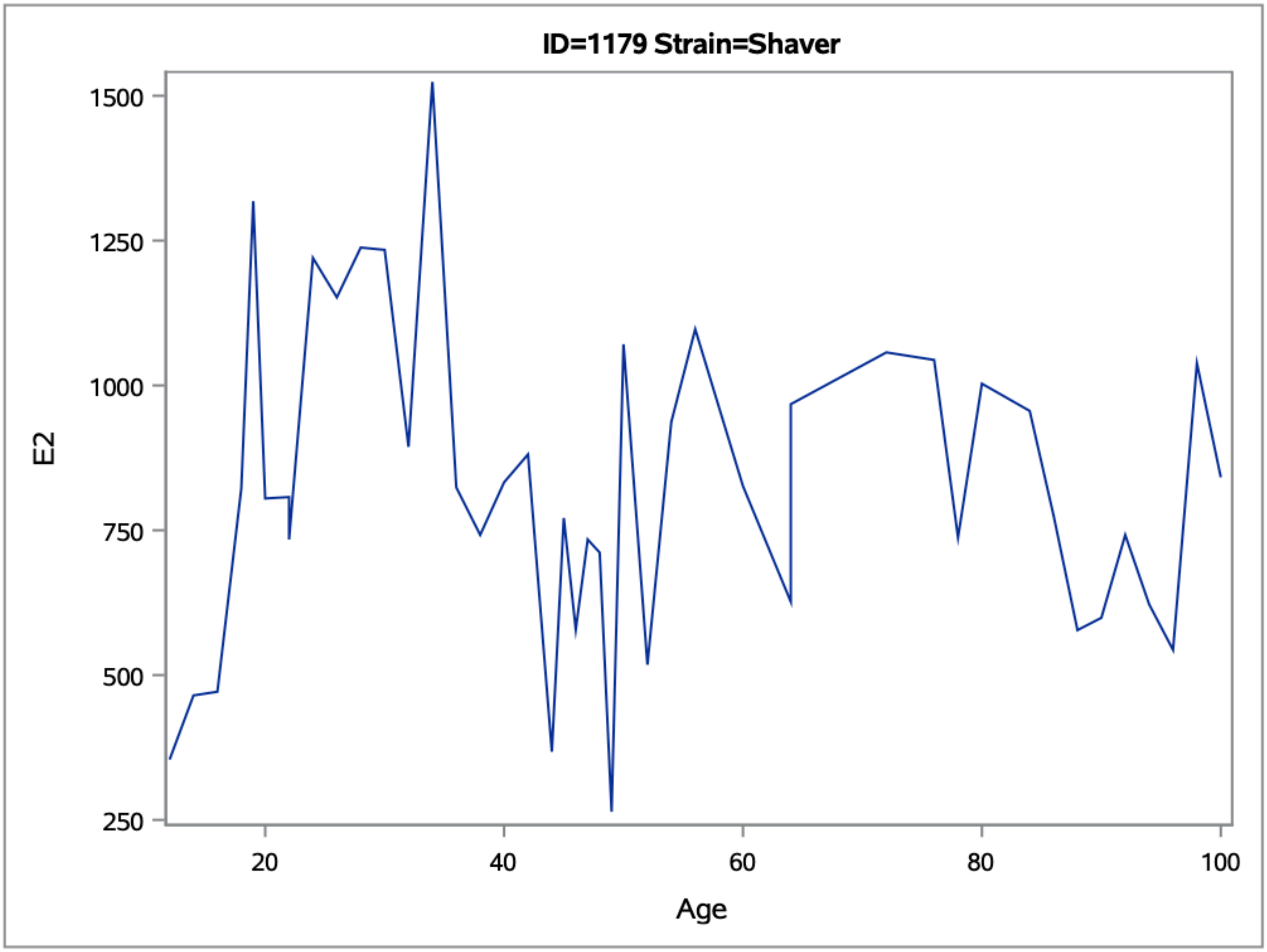
ID=1179 Strain=Shaver
E2
1500
1250
1000
750
500
250
20
40
60
80
100
Age

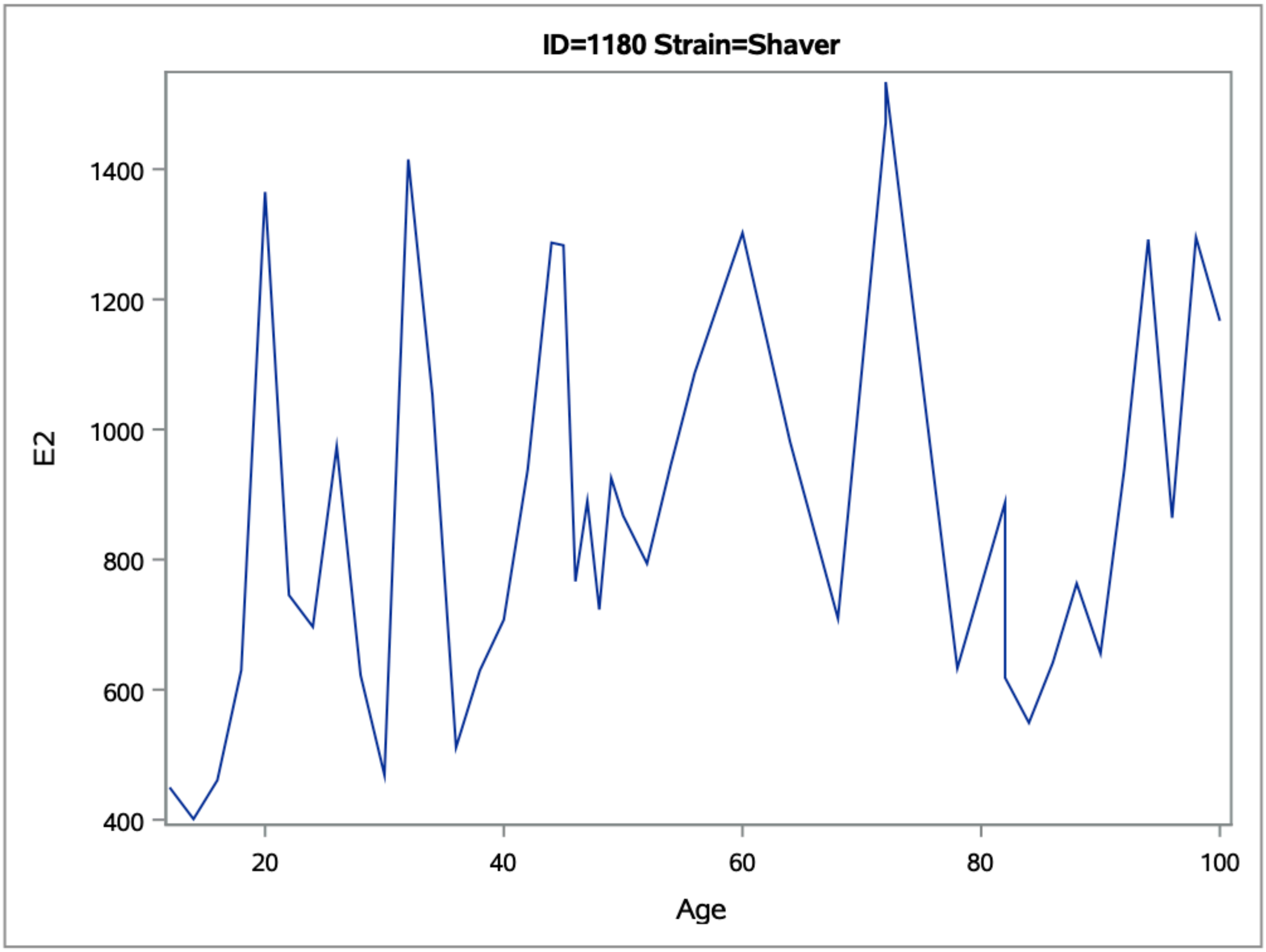

ID=1180 Strain=Shaver
E2
1400
1200
1000
800
600
400
20
40
60
80
100
Age

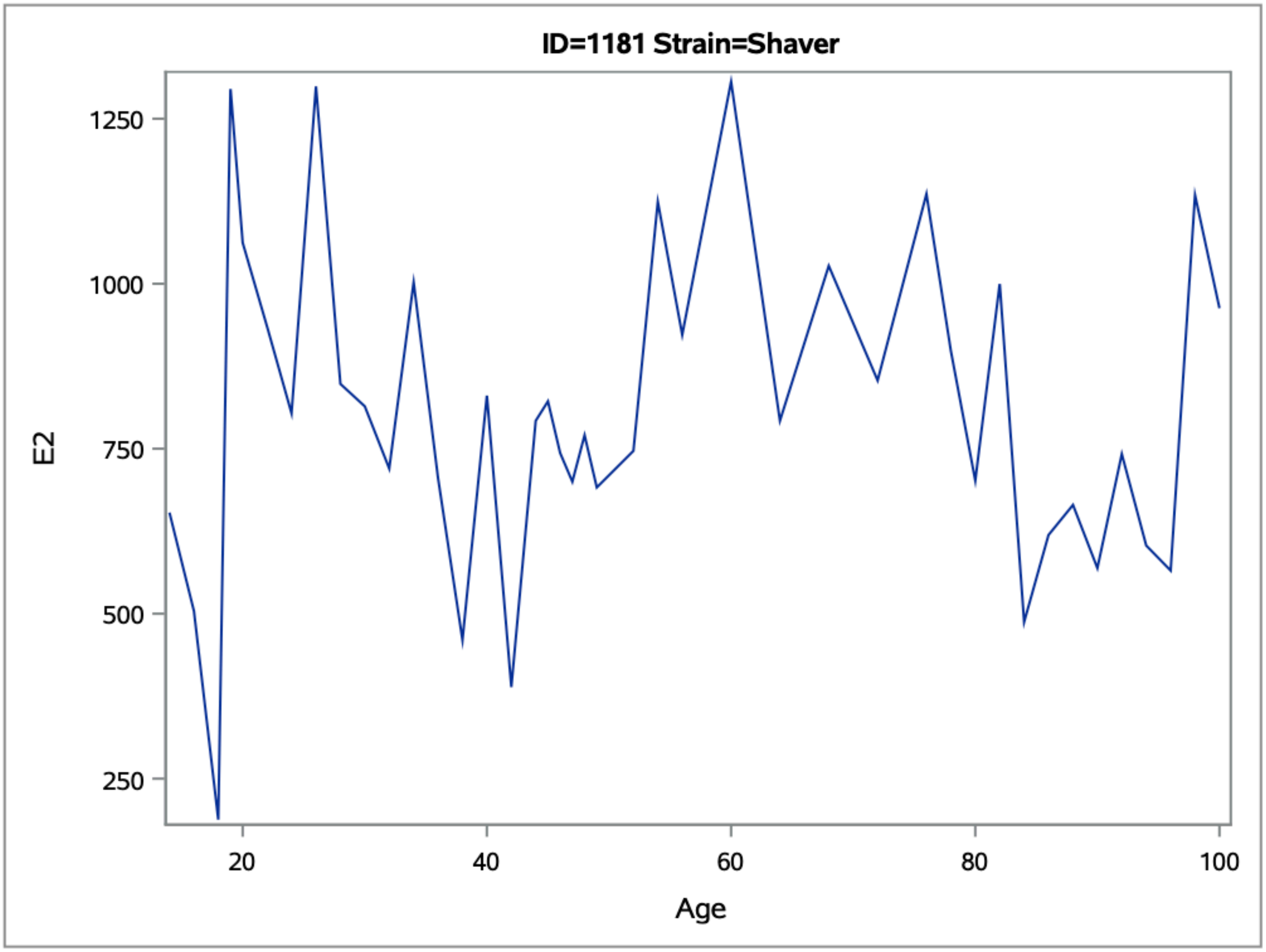
ID=1181 Strain=Shaver
E2
1250
1000
750
500
250
20
40
60
80
100
Age

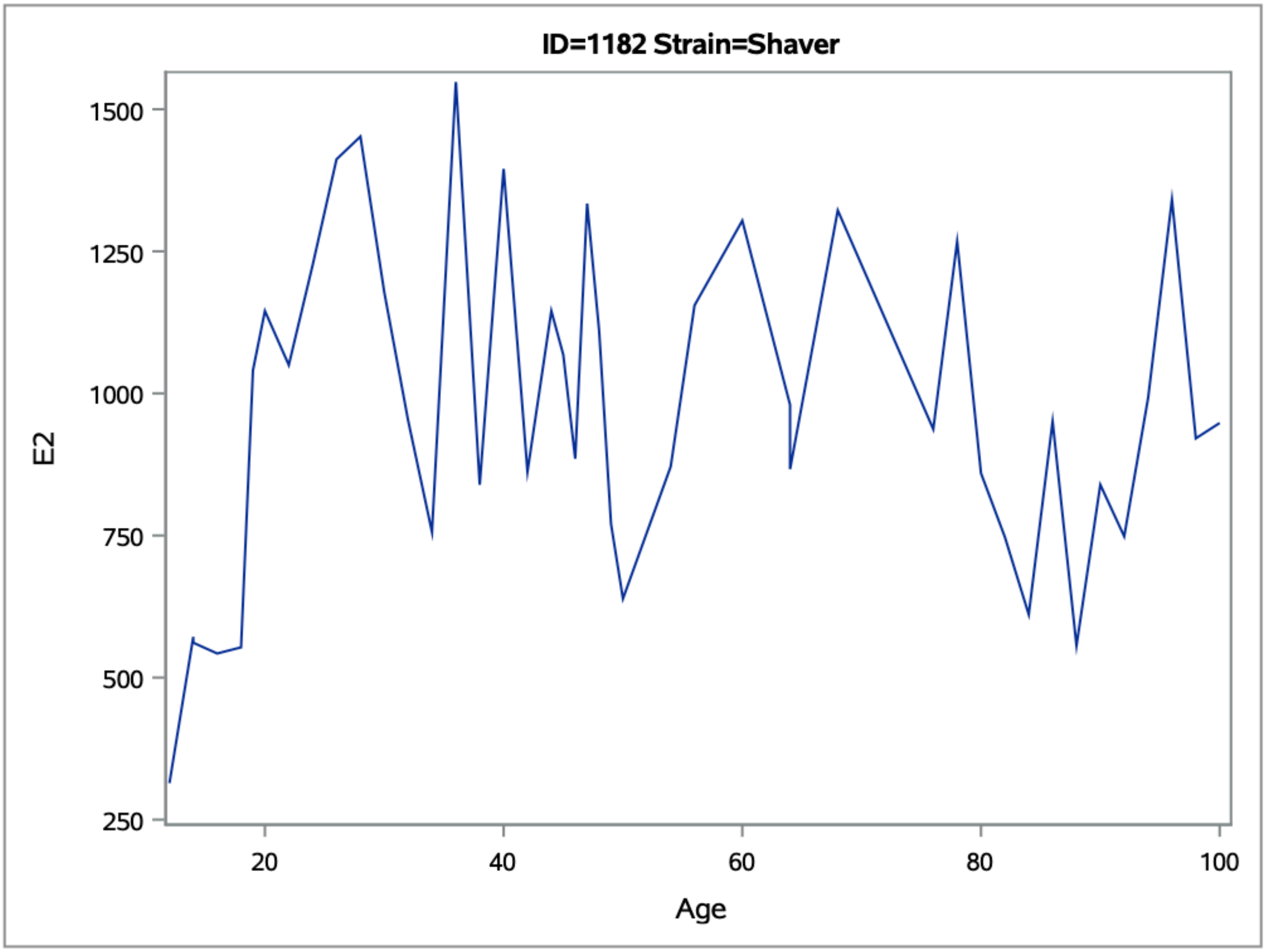
ID=1182 Strain=Shaver
1500
1250
1000
750
500
250
E2
20
40
60
80
100
Age

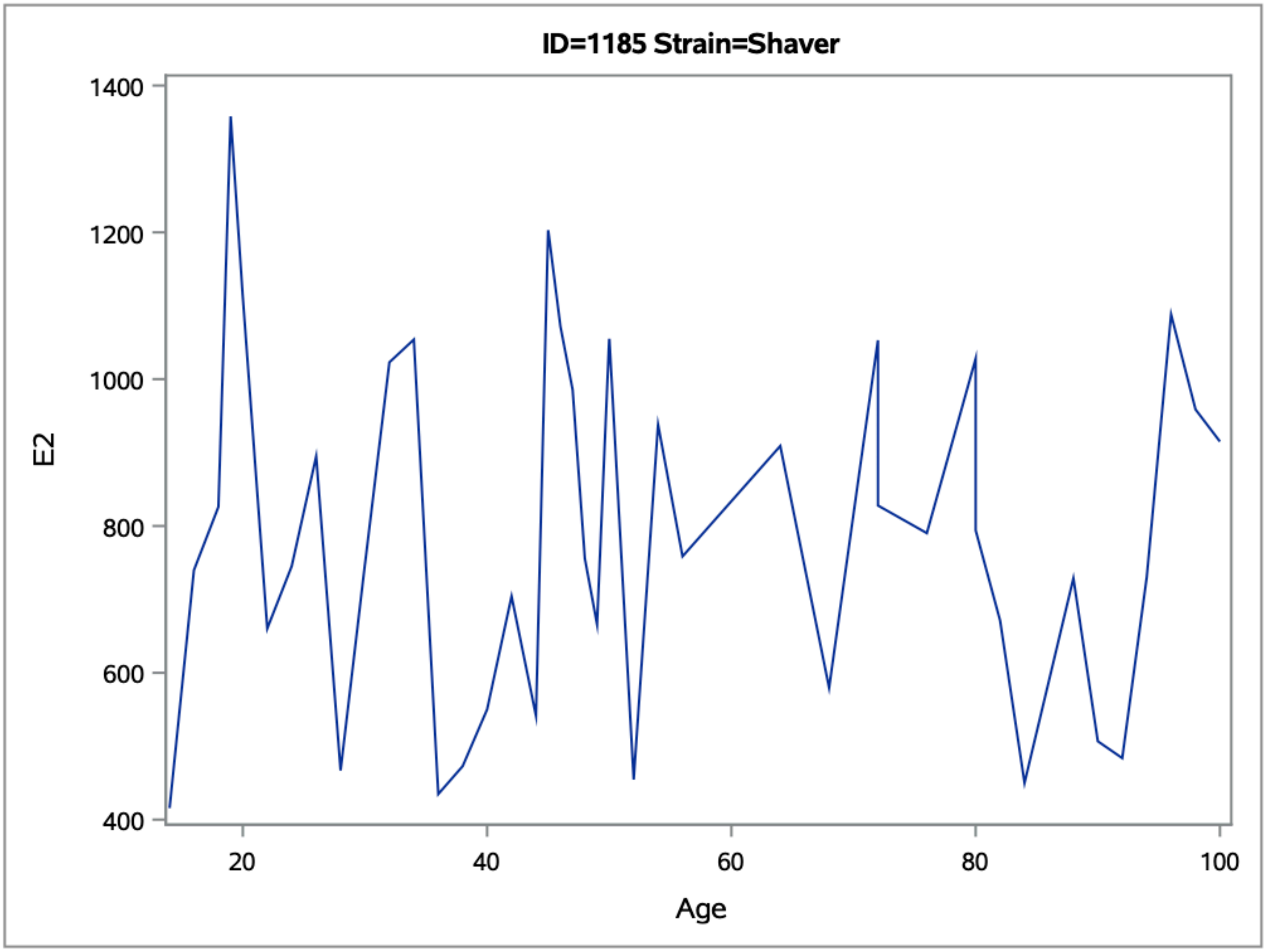
ID=1185 Strain=Shaver
1400
1200
1000
800
600
400
E2
20
40
60
80
100
Age

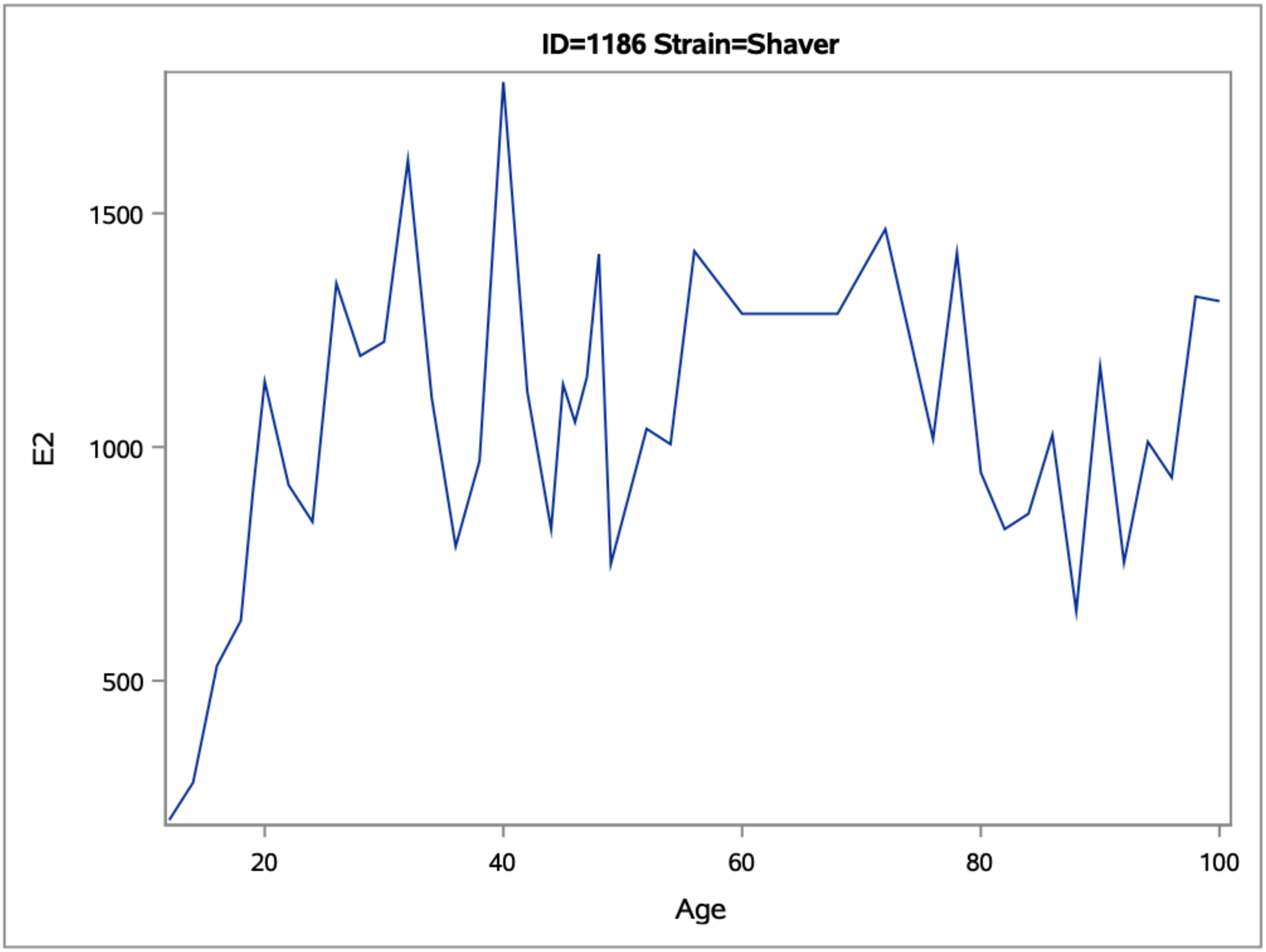
ID=1186 Strain=Shaver
E2
1500
1000
500
20
40
60
80
100
Age

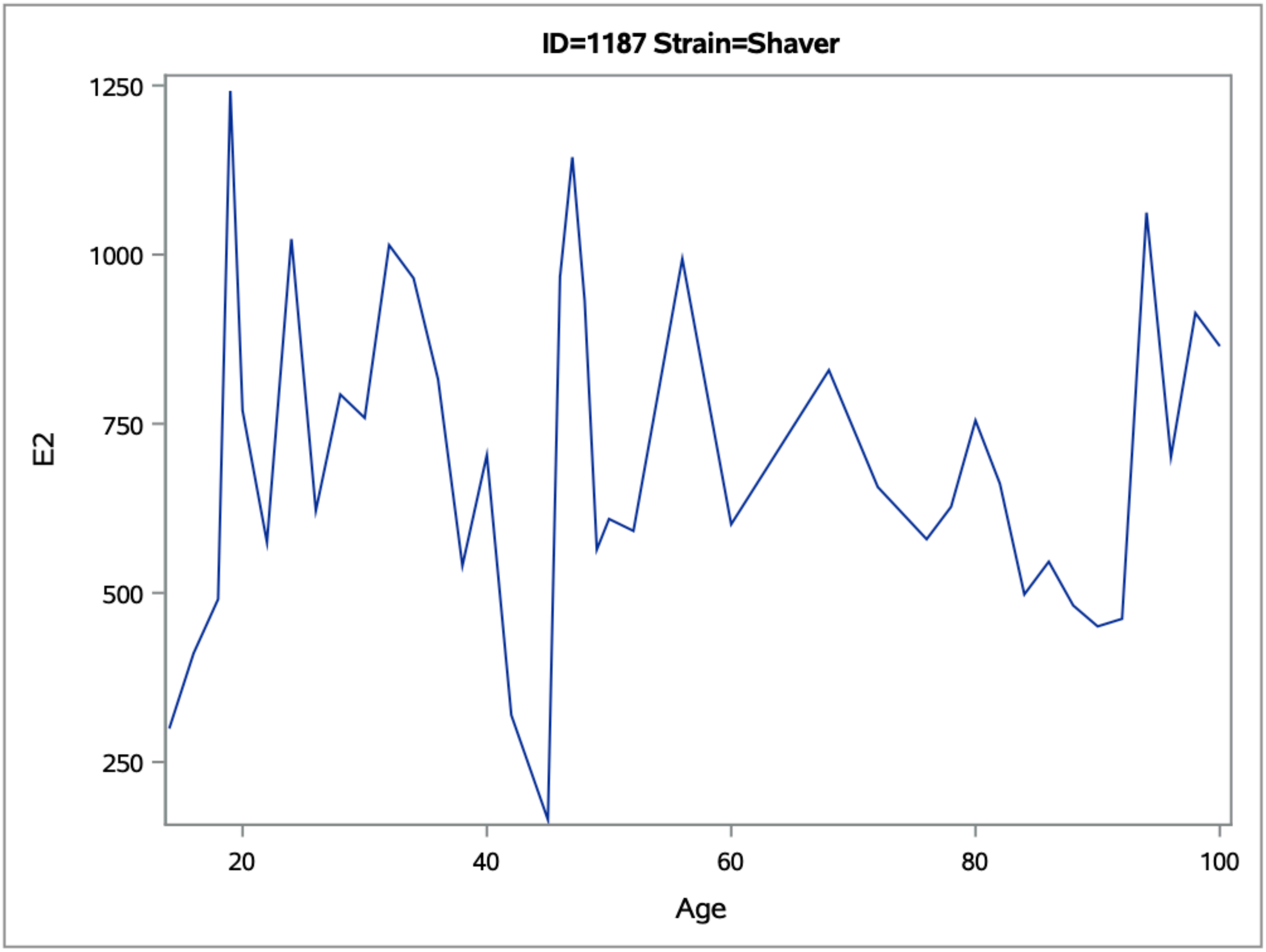

ID=1187 Strain=Shaver
1250
1000
750
500
250
E2
20
40
60
80
100
Age

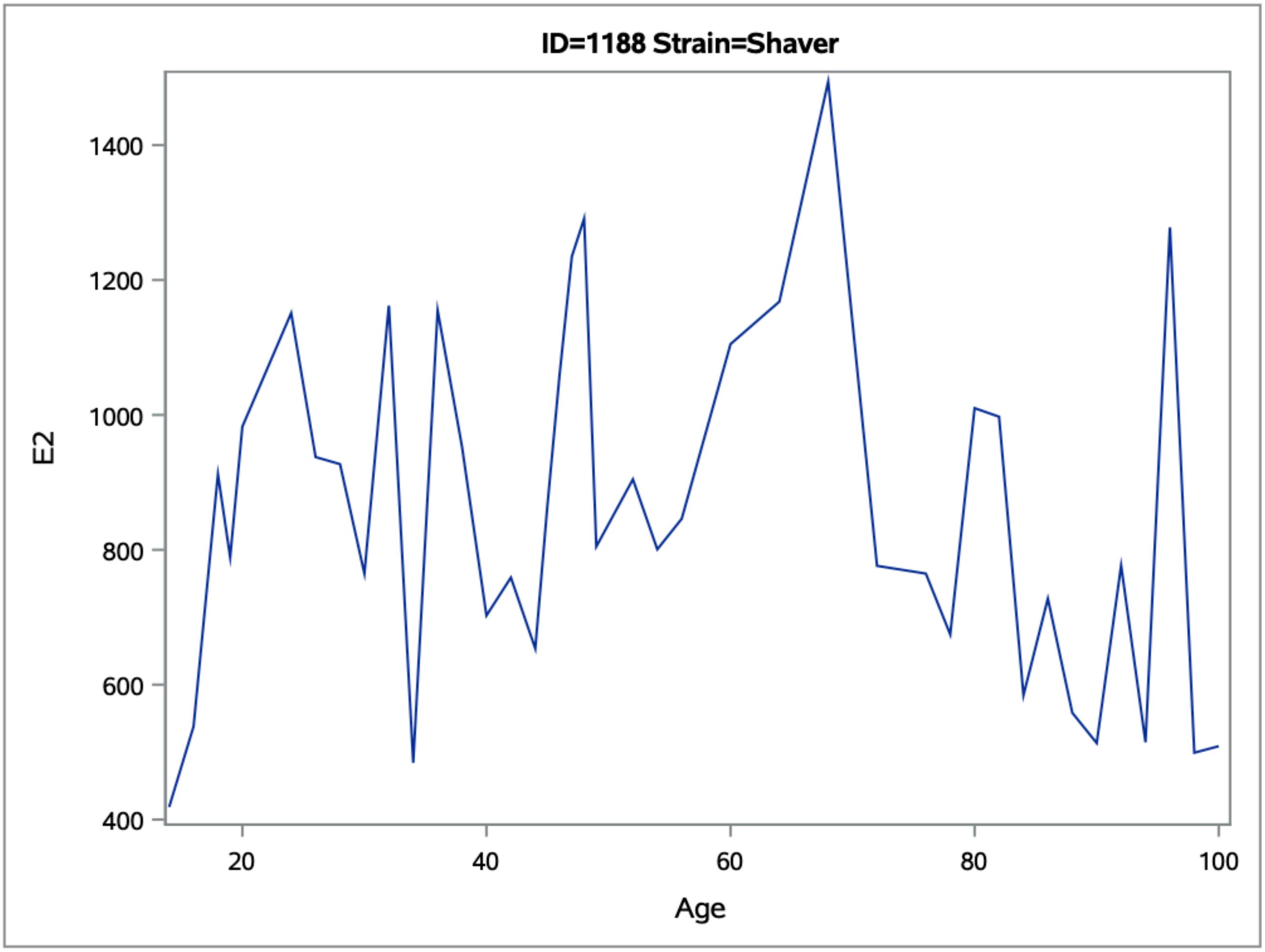
ID=1188 Strain=Shaver
1400
1200
1000
800
600
400
E2
20
40
60
80
100
Age

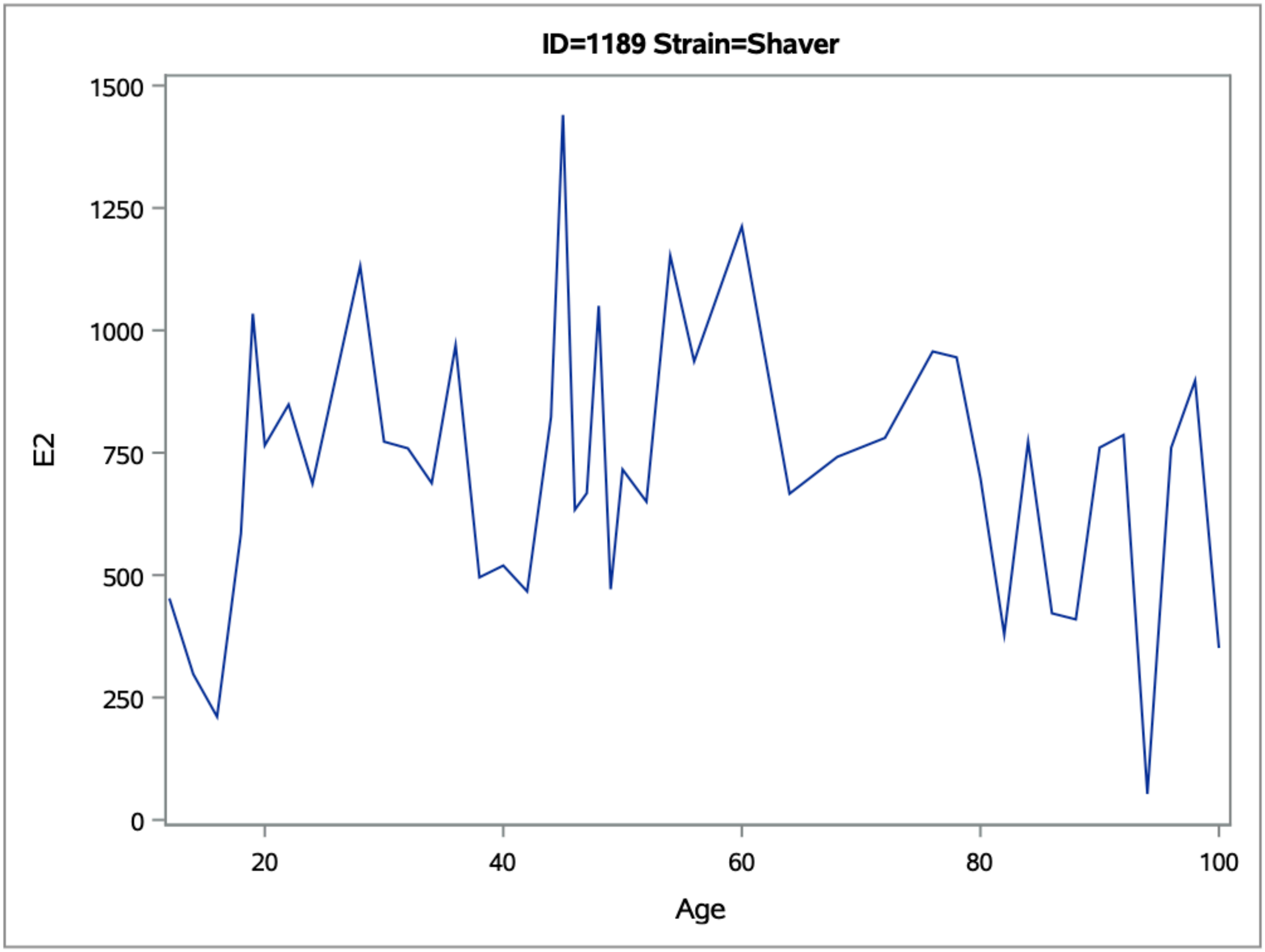

ID=1189 Strain=Shaver
1500
1250
1000
750
500
250
0
E2
20
40
60
80
100
Age

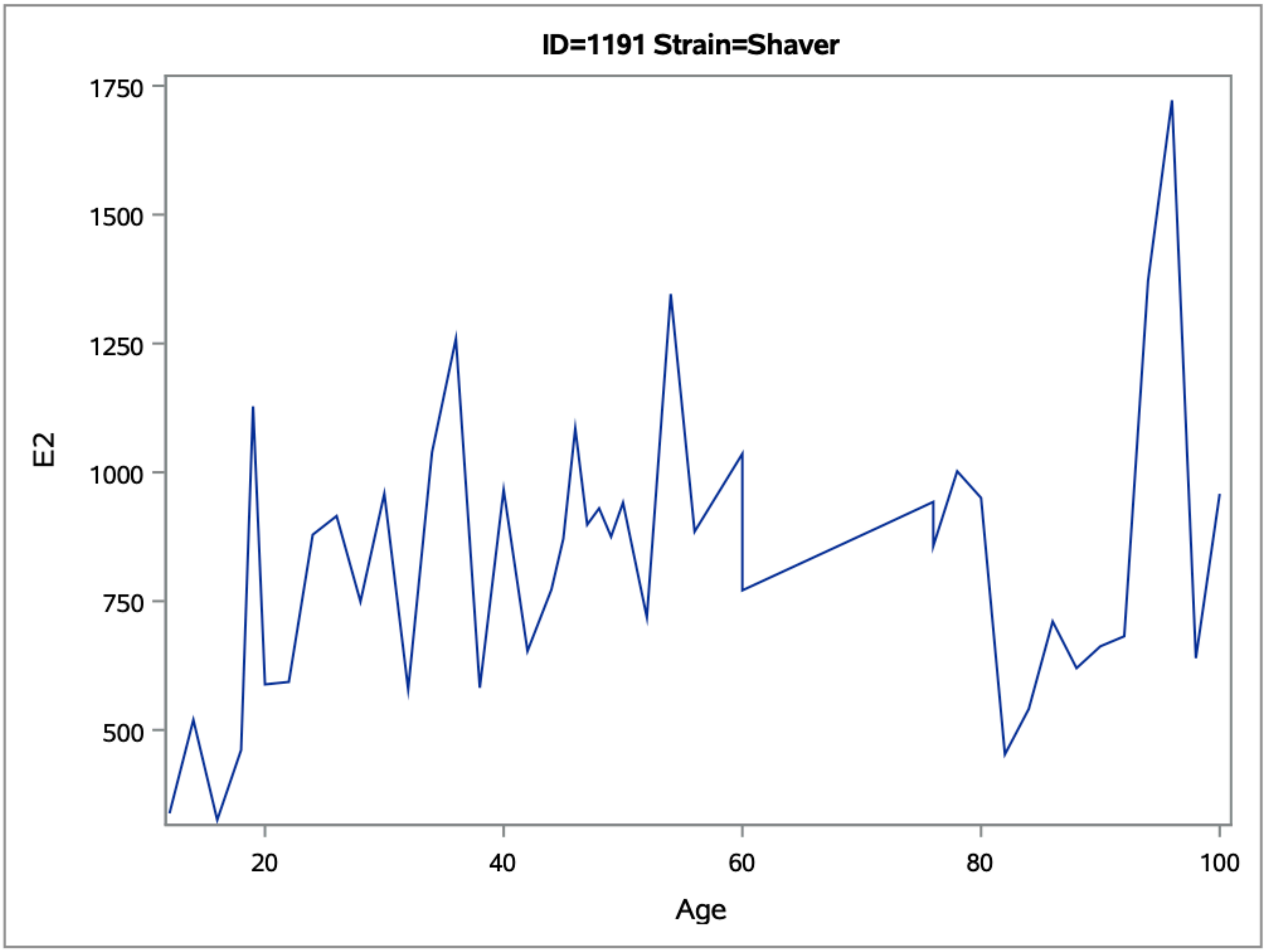

ID=1191 Strain=Shaver
1750
1500
1250
1000
750
500
E2
20
40
60
80
100
Age

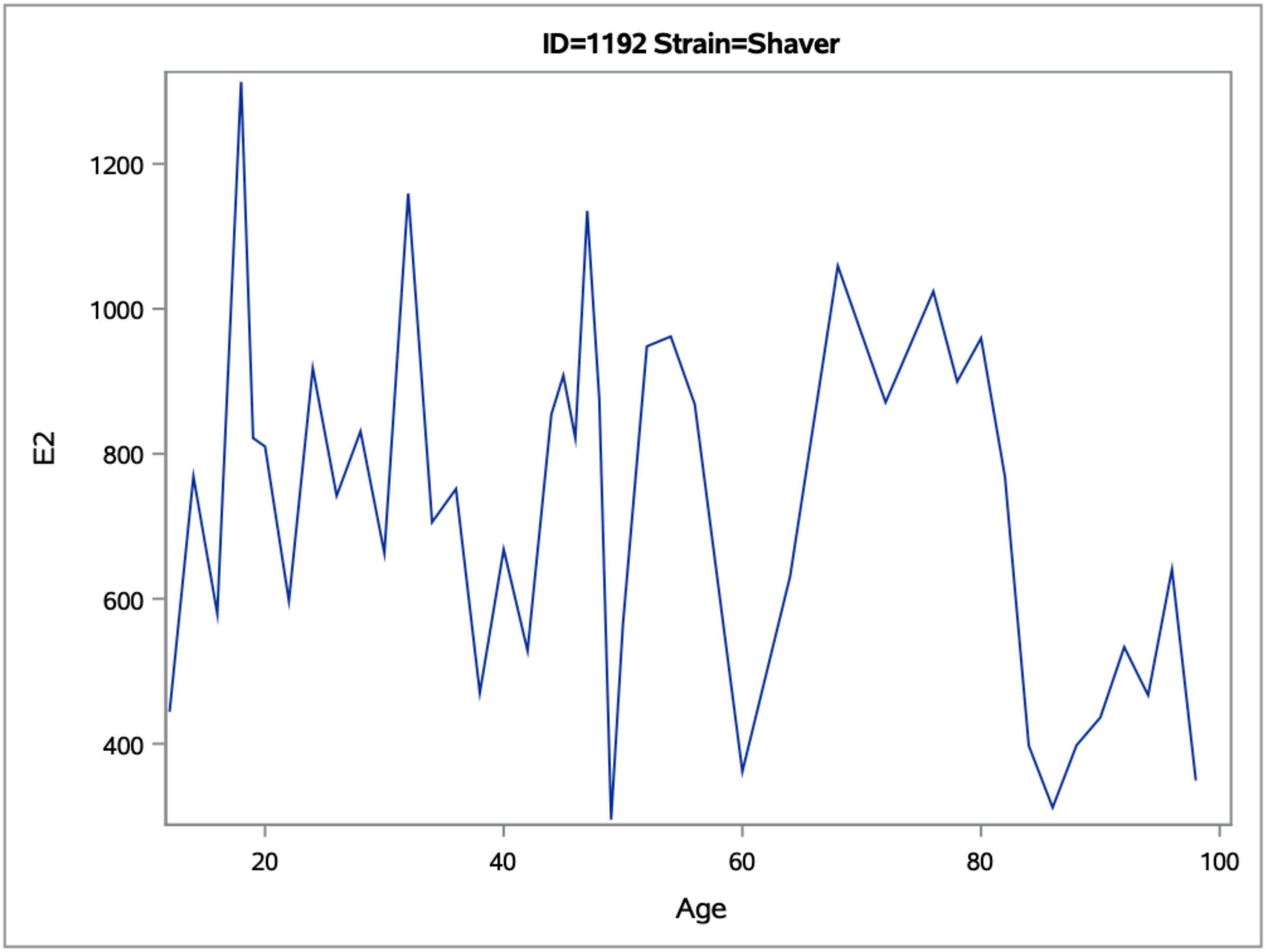
ID=1192 Strain=Shaver
1200
1000
800
600
400
E2
20
40
60
80
100
Age

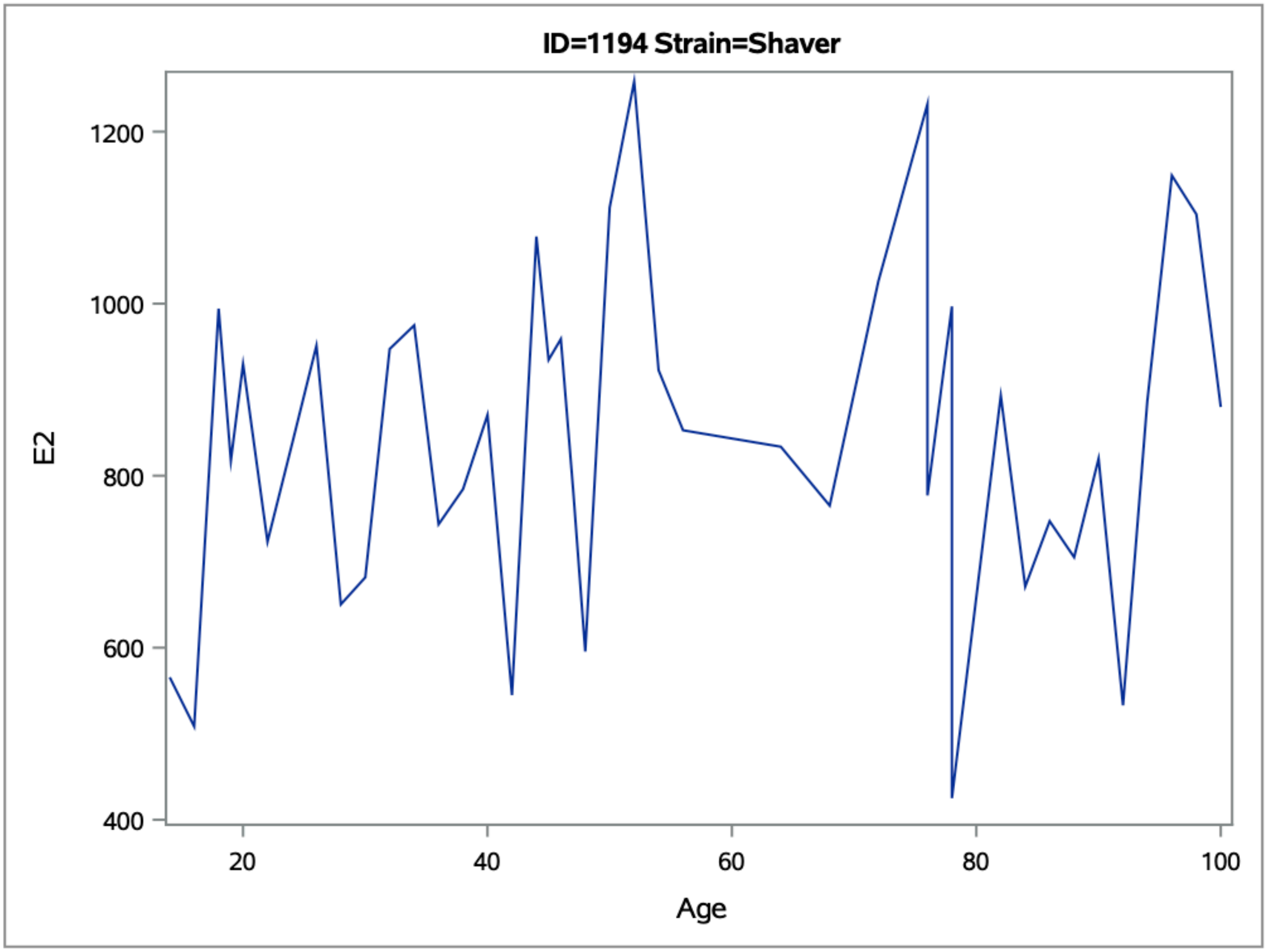
ID=1194 Strain=Shaver
E2
1200
1000
800
600
400
20
40
60
80
100
Age

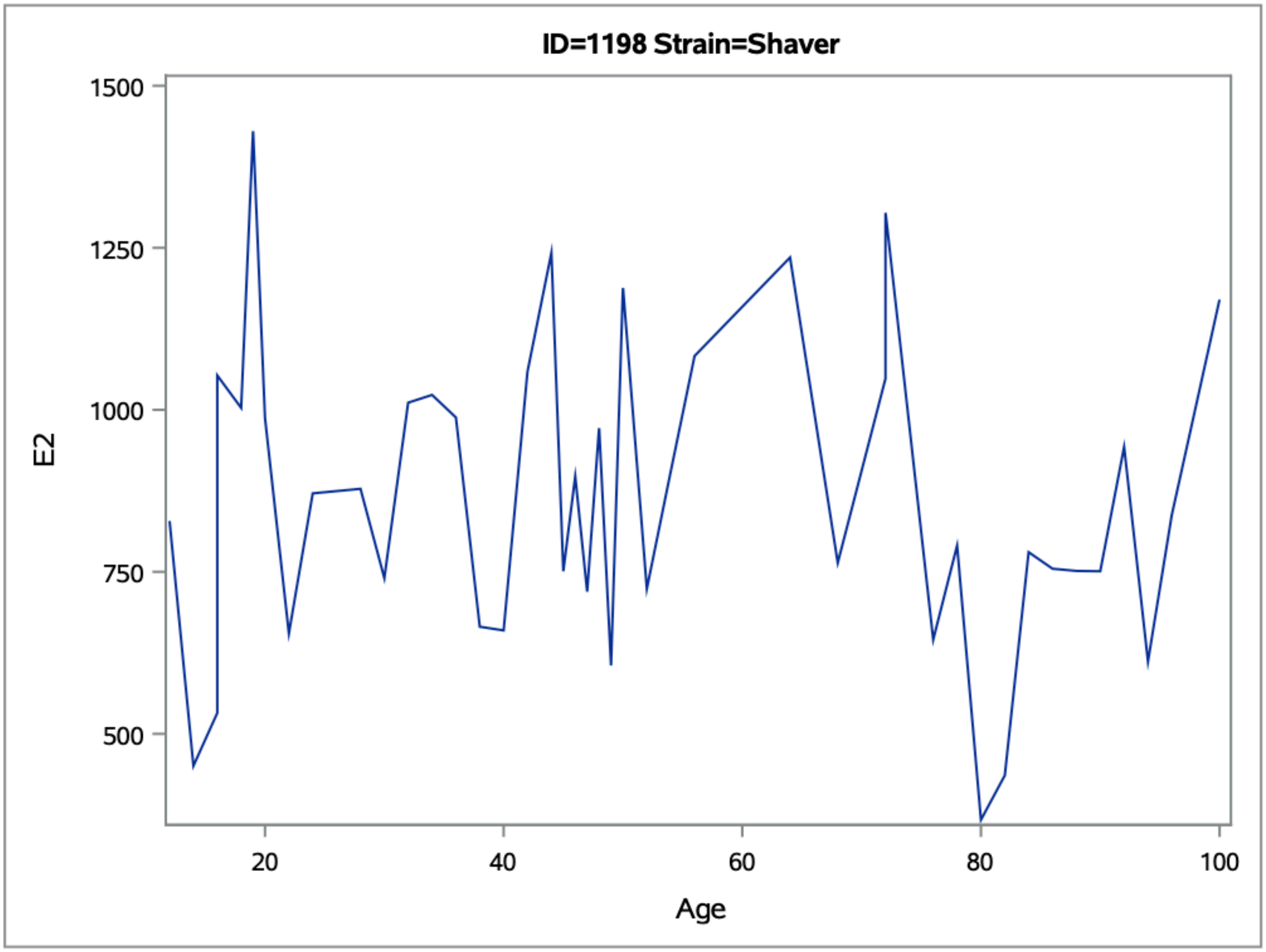

ID=1198 Strain=Shaver
1500
1250
1000
750
500
E2
20
40
60
80
100
Age

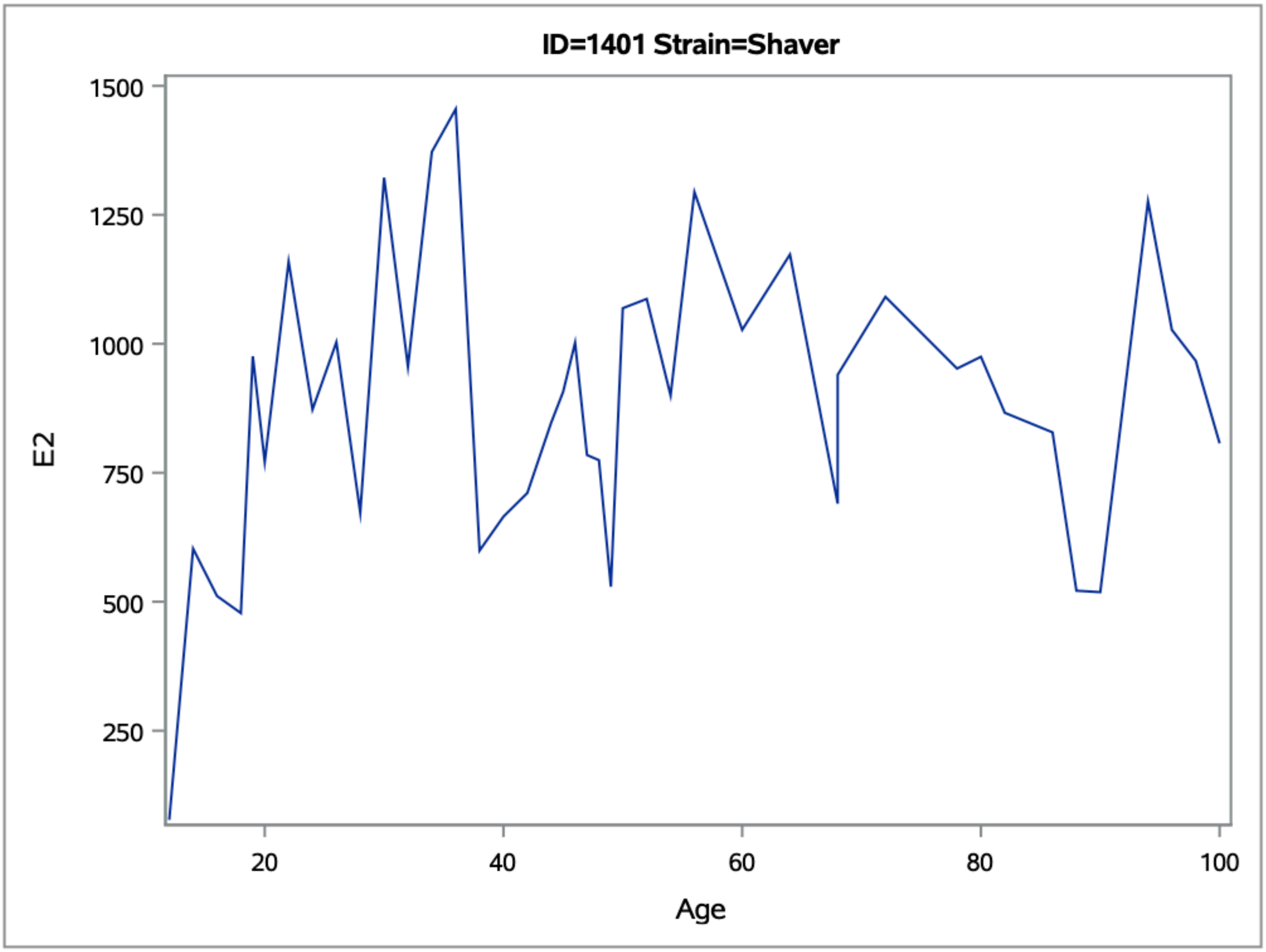

ID=1401 Strain=Shaver
1500
1250
1000
750
500
250
E2
20
40
60
80
100
Age

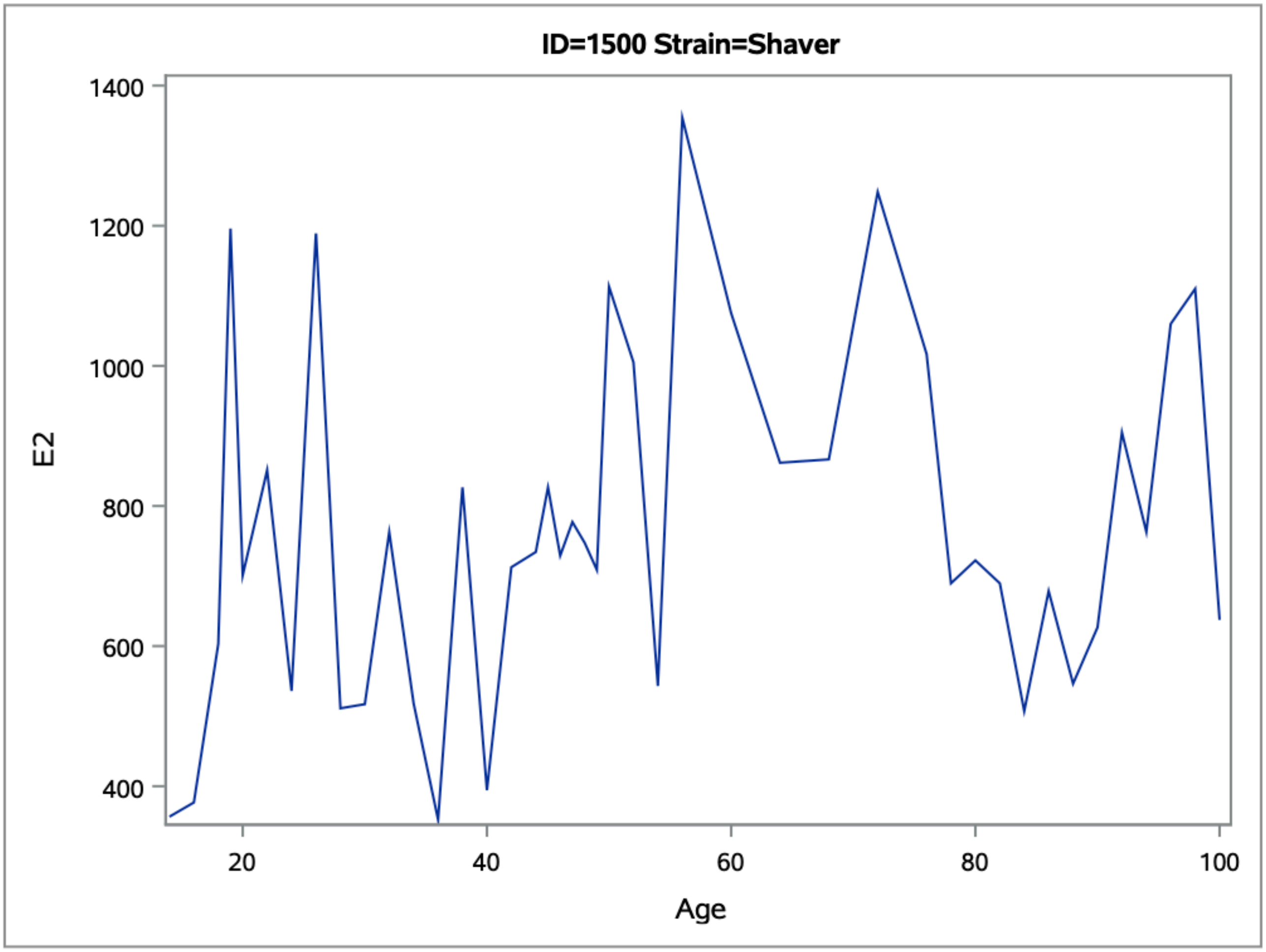
ID=1500 Strain=Shaver
1400
1200
1000
800
600
400
E2
20
40
60
80
100
Age
